# Supplementary material for: Global impact of micronutrients in modern human evolution
Source: Am J Hum Genet. 2025 Sep 10;112(10):2538–61. doi: 10.1016/j.ajhg.2025.08.005 (PMC12696500; doi:10.1016/j.ajhg.2025.08.005)
Supplement: Document S1. Figures S1–S38, Tables S1–S15, and Notes S1–S5 [file mmc1.pdf]

The American Journal of Human Genetics, Volume *112*

Supplemental information

Global impact of micronutrients in modern human evolution

Jasmin Rees, Sergi Castellano, and Aida M. Andrés

Supplemental Materials

Data

Data S1

Table 1: **All micronutrient-associated genes used in this study associated with the uptake, metabolism or regulation of 13 micronutrients.** When genes are associated with multiple micronutrients, their most supported association given in “Micronutrient” with secondary or tertiary associations given in “Other Associations”. Genes removed following the positive mask[2] indicated in the “Removed During Pruning” column. Gene regions as taken from ensemble[32] and suggested from the literature[33–51].

Data S2

Table 1: **Chi-squared test for enrichment of significant SNPs across all MA-genes according to the 5% empirical tail of F_{ST}.** “Total SNPs” = SNPs across all MA-genes in the given population-Yoruba pair; “Exp-Sig” = 5% of total SNPs; “Exp-NonSig” = 95% of total SNPs; “Obs-Sig” = SNPs in the 5% empirical tail of F_{ST} for the given population-Yoruba pair; “Obs-NonSig” = SNPs outside the 5% empirical tail of F_{ST} for the given population-Yoruba pair; “Enrichment” = percentage more observed significant SNPs than expected; “Chi-Squared” = significance of chi-squared test.

Table 2: **Chi-squared test for enrichment of significant SNPs across all MA-genes according to the 1% empirical tail of F_{ST}.** “Total SNPs” = SNPs across all MA-genes in the given population-Yoruba pair; “Exp-Sig” = 1% of total SNPs; “Exp-NonSig” = 99% of total SNPs; “Obs-Sig” = SNPs in the 1% empirical tail of F_{ST} for the given population-Yoruba pair; “Obs-NonSig” = SNPs outside the 1% empirical tail of F_{ST} for the given population-Yoruba pair; “Enrichment” = percentage more observed significant SNPs than expected; “Chi-Squared” = significance of chi-squared test.

Table 3: **Chi-squared test for enrichment of significant SNPs across all MA-genes according to the 5% empirical tail of $Relate$.** “Total SNPs” = SNPs across all MA-genes in the given population; “Exp-Sig” = 5% of total SNPs; “Exp-NonSig” = 95% of total SNPs; “Obs-Sig” = SNPs in the 5% empirical tail of $Relate$ for the given population; “Obs-NonSig” = SNPs outside the 5% empirical tail of $Relate$ for the given population; “Enrichment” = percentage more observed significant SNPs than expected; “Chi-Squared” = significance of chi-squared test.

Table 4: **Chi-squared test for enrichment of significant SNPs across all MA-genes according to the 1% empirical tail of $Relate$.** “Total SNPs” = SNPs across all MA-genes in the given population; “Exp-Sig” = 1% of total SNPs; “Exp-NonSig” = 99% of total SNPs; “Obs-Sig” = SNPs in the 1% empirical tail of $Relate$ for the given population;

“Obs-NonSig” = SNPs outside the 1% empirical tail of $Relate$ for the given population; “Enrichment” = percentage more observed significant SNPs than expected; “Chi-Squared” = significance of chi-squared test.

Table 5: **Chi-squared test for enrichment of significant SNPs across individual MA-gene sets according to the 5% empirical tail of $\boldsymbol{F_{ST}}$.** “Total SNPs” = SNPs across all MA-genes in a given MA-gene set in the given population-Yoruba pair; “Exp-Sig” = 5% of total SNPs; “Exp-NonSig” = 95% of total SNPs; “Obs-Sig” = SNPs in the 5% empirical tail of F_{ST} for the given population-Yoruba pair; “Obs-NonSig” = SNPs outside the 5% empirical tail of F_{ST} for the given population-Yoruba pair; “Enrichment” = percentage more observed significant SNPs than expected; “Chi-Squared” = significance of chi-squared test.

Table 5: **Chi-squared test for enrichment of significant SNPs across individual MA-gene sets according to the 5% empirical tail of $\boldsymbol{Relate}$**“Total SNPs” = SNPs across all MA-genes in a given MA-gene set in the given population; “Exp-Sig” = 5% of total SNPs; “Exp-NonSig” = 95% of total SNPs; “Obs-Sig” = SNPs in the 5% empirical tail of $Relate$for the given population; “Obs-NonSig” = SNPs outside the 5% empirical tail of $Relate$for the given population; “Enrichment” = percentage more observed significant SNPs than expected; “Chi-Squared” = significance of chi-squared test.

<u>Data S3</u>

Table 1: Proportion of MA-gene sets with strong signatures of positive selection according to $\boldsymbol{Relate}$. “Proportion” =Proportion of MA-genes within each MA-gene set (“Micronutrient”) with SNP(s) with signatures of positive selection in the 1% empirical tail of $Relate$ for the given population (“Population”) of interest.

Table 2: Proportion of MA-gene sets with strong signatures of positive selection according to $\boldsymbol{F_{ST}}$. “Proportion” =Proportion of MA-genes within each MA-gene set (“Micronutrient”) with SNP(s) with signatures of positive selection in the 1% empirical tail of F_{ST} for the given population (“Population”) of interest.

Table 3: Number of candidate SNPs identified in each MA-gene set according to $\boldsymbol{Relate}$. Number of candidate SNPs (“No. candidate SNPs”), those SNP(s) with signatures of positive selection in the 1% empirical tail of $Relate$, for each MA-gene set (“Micronutrient”) for the given population (“Population”) of interest.

Table 4: Number of candidate SNPs identified in each MA-gene set according to $\boldsymbol{F_{ST}}$. Number of candidate SNPs (“No. candidate SNPs”), those SNP(s) with signatures of positive selection in the 1% empirical tail of F_{ST} , for each MA-gene set (“Micronutrient”) for the given population (“Population”) of interest.

Data S4

Table 1: Results of the $CLUES2$ analysis of 19 candidate SNPs of iron- and calcium-associated genes. For each population ("Population"), gene ("Gene") and SNP of interest (positions in hg38 given by "Chromosome" and "Position"; see **Note S5** and **Table S5** for criteria in choosing these SNPs), the jointly calculated likelihood of selection ("-LOG10(p value)") and selection coefficient "SelectionMLE1" from each epoch "Epoch" (in generations) to present.

Note S1: Simulations

The forward-simulator SLiM[1] was used to simulate genomic segments of approximately 100,000 base pairs. Each segment was initiated with random nucleotides across its length and included exon, intron and non-coding regions. Variable recombination rates were also specified across this region, modeled according to the inferred distribution of recombination rates in the human genome (as calculated from chr15 of the 929 individuals of the HGDP dataset[2] with 100 different recombination rates given across this region). A gamma distribution of mean 1.311 and shape parameter 0.509 was used to draw recombination rates. The mutation rate was uniform throughout the 100 kb region, specified as per generation and following the Jukes-Cantor model.

We simulated the demographic history of four metapopulations: African, European, East Asian and American, as a combination of two pre-existing demographic models[3,4]. We integrated the inferred demographic history of the Puerto Rican population from the latter into the former model and use it as our proxy of an American population. Here, we note that we have not included deep structure in Africa[5,7,9,11] in our simulations, and emphasise that these simulations are 1) most informative for power in populations with demographic histories similar to those modelled here and 2) should be considered a broad overview of the power of pre-existing methods to identify selection on standing variation. Including deep structure in our simulations would likely improve the power of some methods (*e.g.,* leading to a higher number of older coalescence events that may be leveraged by Relate) and decrease the power of others (e.g., increasing differentiation between some populations and reducing the power of cross-population methods like F_{ST} to identify selected alleles from those with frequency increases under ancient drift) and we encourage future exploration of the effect of deep structure on inferring signatures of positive selection.

The onset of positive selection was set at one of four timepoints (1 kya, 5 kya, 10 kya and 40 kya) in only one of the four metapopulations. A single polymorphic allele segregating in the focal population is tagged given that it lies within the centre 10 kbp of the region and has a frequency of 0.1-0.15. The tagged mutation is then given a selection coefficient drawn from a uniform distribution between 0.001 and 0.005 and hence represents positive selection on standing variation. Each successful simulation run is a proxy for weak selection acting on a single genomic region, analogous to a gene region or haplotype, or can be grouped into “gene sets” to approximate polygenic selection.

An initial burn-in period was covered between 1.66 mya and 70 kya to allow the ancestral population to reach mutation-drift equilibrium. Beneficial, neutral or deleterious mutations were initiated in the exon regions, where the selection coefficients of deleterious mutations were drawn from a gamma distribution (mean: -0.03 and shape parameter: 0.2[6,8]) and beneficial mutations drawn from an exponential distribution (mean: 0.01, capped at 0.05). Neutral mutations appear in the exon, intron and non-coding regions.

To reduce CPU time, simulations were rescaled by a factor 5, reducing the number of simulated individuals. Mutation rate, recombination rate and selection coefficients were scaled up whilst effective population size scaled down, maintaining the necessary

population-genetic parameters[1]. The generation time was also downscaled by the same factor to account for the fact that genetic drift occurs faster in smaller populations. This reduced the CPU time by over a factor 20.

The output from the burn-in stage was then scaled up to 14,474 individuals via random mating. This represents the ancestral African population at 70 kya, which then undergoes population splits, expansions and migrations (as described in **Fig 1**) and the initiation of positive selection in a single segregating allele. For each scenario (the combination of one selection timepoint in one metapopulation), ~10,000 simulations were run on the requirement that the tagged mutation remains polymorphic in the focal population. For each run, VCF files of 50 individuals for each metapopulation were generated as output, alongside the position, selection coefficient and final frequency of the tagged mutation. The distribution of derived allele frequency (DAF) of selected alleles in the focal population are shown in Fig S1. A CSV files was also generated containing information on the inclusive upper bound position of each recombination rate, which was converted to a standard genetic map format, where recombination rate is given in cM/Mb. To simulate neutrality, a matched number of post-burn-in simulations were ran with no initiation of positive selection.

Note S2: Power analysis

Seven methods were applied to identify the genetic signatures of positive selection in the simulated genetic data. Four of these methods use haplotype structure to infer SNPs with evidence of positive selection but do so in subtly different ways. iHS and nSL both consider the length of haplotype homozygosity (where extended haplotype homozygosity is indicative of alleles rapidly rising in frequency, as expected under strong positive selection), with iHS measuring length as the recombination distance and nSL measuring length as the number of segregating sites[10,12]. nSL is an extension of iHS, a commonly used method to identify positive selection, and has been suggested to be more powerful when detecting selective sweeps on standing variation[12]. XPEHH and XPnSL are further extensions of these two methods (of iHS and nSL respectively) and compare the haplotype homozygosity between two populations to identify SNPs with unusually long haplotype length in one population[13,14].

The remaining methods evaluated include one which uses allele differentiation between populations to identify signatures of positive selection ($\mathrm{F_{ST}}$[15]) and two that consider genealogical-based evidence of positive selection (Relate and SDS[16,17]). Relate first infers local trees along the genome (where unique trees are separated by recombination breakpoints); before simultaneously estimating branch lengths, mutation rates and effective population sizes to re-infer the trees[16]. The inferred trees can then be used to evaluate the probability of a variant's trajectory under neutrality, leveraging the inferred history of the allele. SDS, however, simply uses the inferred tip branch lengths to identify trees with short terminal tips and, by extension, recent rapid allele frequency change[17].

All haplotype-based statistics were calculated using the $\mathrm{SELSCAN}$ programme[18] and normalized according to SNP frequency. For XPEHH and XPnSL, calculations were repeated for each combination of focal population with the three remaining populations.

$\mathrm{VCFTOOLS}$[19] was used to calculate per-site F_{ST} according to the Weir and Cockerham (1984) method[15], again for each combination of each focal population with the three remaining populations. The RELATE programme[16] was used to infer genealogies across simulated gene regions, re-infer branch lengths and calculate the $\log_{10}\mathrm{p-value}$ for positive selection. The RELATE programme was also used to directly calculate SDS on the inferred genealogies[16,17].

<u>Power of methods</u>

We categorised selected SNPs as having signatures of positive selection if they fall in the extreme 5% tail of the distribution of neutral simulations. These were built from each method's output values calculated on VCFs simulated from the neutral simulations (the same demographic scenario but with no onset of positive selection in any metapopulation). We caution that whilst the accuracy calculated for the African, European, East Asian and American populations are useful for observing patterns across methods and time, these populations should not be directly compared to each other. This is because we condition on the selected allele to persist to the end of the simulation. While this is a necessary condition widely used in comparable power analyses, it does result in differential biases across populations (since differences in demography result in differences in the probability of an allele to survive to the time of sampling). Therefore, the final set of simulated genomic regions is informative but not perfectly comparable across demographic histories and populations, even within this paper.

The highest true positive rate (TPR) of identification of SNPs under positive selection is observed when using F_{ST} for cross-population methods and Relate for single-population methods[15,16], with the haplotype-based methods and remaining tree-based method SDS displaying poor accuracy in comparison except in particular cases (**Fig S2-3**). This is likely due to the fact that we simulate selection on standing genetic variation. In cases where selection is recent or ongoing and on the same haplotype background (as would be expected under selection on *de novo* mutation), we would expect the power of haplotype-based methods to be substantially higher. We also observe minimal differences in the TPR of the haplotype-based methods that use recombination distance (iHS and XPEHH[10,13]) compared to those that use number of segregating sites as a proxy for distance (nSL and XPnSL[12,14]), perhaps because power is always quite low. For all methods, and as expected, the highest accuracy is observed for the oldest simulated selection (positive selection initiated at 40 kya) and for selection in the simulated population with the largest Ne (the African population; **Figs S2-3**).

The only exception to this pattern is for recent selection which, as expected, in some populations is best identified by SDS (10,000 years ago or earlier; **Fig S2**). Nevertheless, SDS has still low TPR at those timepoints, has much lower TPR than other statistics at older timepoints, and is limited in the small sample sizes available in this study, making it a less suitable statistic overall for our purposes. Further, 40,000 years represents a particularly interesting timeframe that encompasses major human migrations and the subsequent exposure to novel selective pressures.

Further, TPR of can be as high as 69.6% and 86.9% for Relate and F_{ST}, respectively, when selection is in the simulated African population and selection coefficients between

0.04-0.05% (**Figs S4-5**), with TPR sharply increasing with derived allele frequencies higher than 50% (**Figs S6-7**). It follows that, while our simulation design does not allow to confirm this, we expect considerably higher TPR than in **Figs S2-3** with selection coefficients over ~ 0.04.and/or frequency of the derived allele higher than 60%. In this simulation design, the false negative rate (FNR) is equal to 1-TPR and therefore follows the inverse patterns as described above, being as low as 30.4% and 13.1% for Relate and $\mathrm{F_{ST}}$, respectively, when selection is in the simulated African population and selection coefficients between 0.04-0.05%.

The false positive rate (FPR) is below 8.6% for all the tested methods, with minimal differences amongst individual methods (**Figs S8-9**). The FPR of the two most promising methods ($\mathrm{F_{ST}}$ and Relate[15,16]) are as follows: the FPR of $\mathrm{F_{ST}}$ is below 7.6% across all timepoints and populations (in comparison to FPR ~5% to other cross-population methods; **Fig S9**); the FPR of Relate is below 5% and lower than compared methods for all populations and timepoints except selection at 40kya in the simulated Asian and American populations (**Fig S8**; FPR climbs to 8.5% for selection at 40kya in America). We emphasise that whilst the empirical tails are enriched for targets of positive selection, especially in the case of $\mathrm{F_{ST}}$ (**Figs S2-3**), not all SNPs in this tail are true targets.

We also evaluate the TPR and FPR when using the raw output of Relate $(\log_{10}\mathrm{p}-\mathrm{value})$ to identify SNPs under positive selection for the same significance threshold (5%, or $\mathrm{p}-\mathrm{value}=0.05$). Given that the raw output of Relate is the transformed probability of a mutation rising to its observed contemporary frequency given it's inferred coalescence history, this method may have reduced power when applied to small sample sizes of high stochasticity and / or samples that poorly represent the full coalescence history (in comparison to using neutral distributions which incorporate more information on the expected distribution under neutrality, including increased stochasticity due to small sample sizes). We hence compared the use of the tail of the neutral distribution and the raw output of Relate to identify SNPs under positive selection for two sample sizes: 25 and 50 simulated individuals. For all simulated populations, using the neutral distribution to identify SNPs under positive selection leads to a higher TPR than using the $\log_{10}\mathrm{pvalue}$ when sample sizes are decreased to 25 individuals (**Fig S10**). With sample sizes of 50 individuals, the neutral distribution also displays a higher TPR in the simulated African population but performs poorly in the simulated European and East Asian populations (with no difference in the TPR in the simulated American population; **Fig S10**). This is likely because the neutral distribution better incorporates the high variance in small-Ne populations and small samples sizes than the raw Relate output can. The patterns of FPR are less clear, but we note that the difference in FPR between SNPs identified as under positive selection according to the neutral distribution versus the $\log_{10}\mathrm{pvalue}$ is < 2% for all sample sizes, tested timepoints and populations (**Fig S11**).

We conclude that when sample sizes are small (~ 25) in real data examples, the empirical distribution should be used to identify SNPs under positive selection with Relate. With larger sample sizes, the $\log_{10}\mathrm{pvalue}$ may have slightly higher power in very specific cases, for example in populations with demographic histories similar to the

simulated European and East Asian populations. We thus select significant SNPs based on the extreme 5% tail of the neutral distribution for Relate too.

Finally, we evaluate the TPR of the gene-set enrichment method SUMSTAT[20] to identify polygenic selection. This method extracts the most significant pvalue (in the direction of selection) of each gene within a gene set, summing across the gene set to give a final score (or **SUMSTAT value**). Hence, this method considers the signatures of positive selection on potentially small effect mutations across the entire gene set and has been shown to be more powerful than gene set enrichment analysis in identifying polygenic selection[21]. Gene sets with SUMSTAT summed values in the 5% tail of the neutral distribution (where this distribution is built from gene sets comprising gene regions simulated under neutrality, see above) are identified as those with signatures of positive selection.

We applied the SUMSTAT method to the two most promising methods (F_{ST} and Relate[15,16]). To explore the effect of gene set size and proportion of genes in the gene sets under selection, we built gene sets of various sizes (10, 20, 40, 60) and with varying proportions of genes under selection (20%, 40%, 60%, 80% and 100% gene regions under a selection) by random sampling simulated gene regions (**Fig. S12-13**). SUMSTAT with F_{ST} have high TPR for selection initiated at 40,000 years ago for gene sets of all sizes with 100% of genes under selection (TPR $> 82\%$ in the simulated African population, TPR $> 79\%$ when considering all simulated populations) and for large gene sets with proportions of genes under selection as low as 60% (e.g., TPR $> 84.9\%$ for gene sets of size 40 or larger when selection is at 40,000 years ago in the simulated African population, TPR $> 70.3\%$ when considering all simulated populations; **Fig S13**). SUMSTAT with Relate pvalues only has comparable TPRs for large gene sets with 80% or more genes under selection, and is more sensitive than SUMSTAT with F_{ST} to decreasing gene set size or proportion of genes under selection (**Fig S12**). Still, for selection initiated at 40,000 years ago, a TPR of $> 76.8\%$ can be achieved in the simulated African population if the gene sets are large (40 or 60 genes) and all genes are under selection (with TPR maintained $> 73\%$ when considering all simulated populations). If the proportion of genes under selection in these gene sets drops to 80%, TPR can vary between $54.5\% - 99.\%$, with a TPR $> 65.3\%$ for gene sets of size 60 when considering all simulated populations.

Note S3: Population Analysis

We use 929 full human genomes from the HGDP dataset (as published by[2]), which encompasses 54 populations across Africa, the Middle-east, Europe, East Asia, Central-South Asia, Oceania and the Americas and represents a significant proportion of human ethnic and cultural diversity. Since low sample sizes can significantly reduce the power to identify the genomic signatures of positive selection[22,23], we aim to merge populations with sample sizes below 20 with their geographically closest populations. In these cases, we acknowledge that the signatures of fine scale positive selection in response to extremely localised micronutrient soil levels may be lost, but we consider this a necessary step to maintain adequate power to identify positive selection that may be shared across these geographically close populations.

We carried out population analysis to verify that this criterion agreed in all cases with patterns of population differentiation. We calculated PCs for each metapopulation using plink[24], having thinned for linkage disequilibrium (pruning r^2 values above 0.2) and using windows of 50 kbp and window step size of 10 bp (see **Fig. S14-20**). This analysis confirmed that grouping by geography agrees with population differentiation, with two exceptions (see below), and we hence group according to this criterion.

When grouped, our final dataset comprised of 913 individuals from 40 populations, of which 10 are a result of merging (see **Table S16**). Two merged populations do not follow geography (Bantu-speaking population and the Xibo-Mongolian population), but instead reflect recent migrations[25–27]. Two populations were removed from our analysis (Columbian, n=7; Cambodian, n=9) since they do not group naturally geographically or genetically. Despite their small sample size, the Ju|'hoan population (n=6) was retained in our final dataset given their relatively distinct genetic variation.

Note S4: Adaptive Introgression

We briefly consider if adaptive archaic introgression has played a role in mediating genetic adaptation to micronutrients by determining which top-ranking SNPs fall in regions previously inferred as introgressed from Neandertal and Denisovan[28,29]. Whilst archaic introgression can result in genetic signatures falsely interpreted as those of local adaptation, the allele frequency of top-ranking SNPs in all non-African populations is higher than what would be expected under neutral archaic introgression (all top-ranking SNPs in non-African populations identified by $\mathrm{F_{ST}}$ have allele frequency > 10%; 99.47% of top-ranking SNPs in non-African populations identified by Relate have allele frequency > 10%). Hence, we propose that any archaic introgression within the MA-gene set would be adaptive in nature.

We suggest that two extreme candidate MA-genes have evidence of archaic introgression. First, the magnesium-associated *MECOM*: candidate SNPs (empirical p-value < 0.1%) of this gene in the Middle Eastern Mozabite and Central-South Asian Hazara populations fall in inferred regions of adaptive archaic introgression from Neanderthals[28,29]. Second, the zinc-associated *SLC30A9:* a previous study inferred adaptive alleles in East Asians to be introgressed from Denisovans[30] (which we do not identify with our limited analysis of archaic introgression using introgressed tracts from [28,29]).

Extreme candidate SNPs in these two genes (empirical p-value < $4.65\mathrm{e}^{-6}$) are identified using Relate in populations of the same geographic region (**Table 2**) as those with evidence of archaic introgression as referenced above, indicating a potential role of archaic introgression in mediating dietary micronutrient adaptation.

Note S5: Inferring Age of Positive Selection

We used CLUES2[31] to infer the timing of selection on four iron-associated and five calcium-associated genes to address the hypothesis that recent changes to the diet (*i.e.,* those

surrounding the Neolithic transition) drove putative iron and calcium-associated adaptation. Due to computational constraints, we only carry out this analysis on 19 candidate SNPs (those in the 0.1% empirical tail of either the $\mathrm{F_{ST}}$ or $Relate$) across the nine iron- and calcium-associated genes. The chosen candidate SNPs all have strong evidence of positive selection in one or more populations as identified by either $\mathrm{F_{ST}}$ or $Relate$ (see **Table S6**) and, in some cases, lie within regions of the respective MA-gene with a high density of signatures of positive selection (and we hence consider them representative of those regions). The candidate SNPs referenced here (see **Table S6**) also include the top candidate SNPs in HIF1A, FTMT and ATPB2 which bypass our most stringent threshold; $\mathrm{p-value} \leq 4.65\mathrm{e}^{-6}$.

For all 19 of the candidate SNPs, and for each population, we convert the tree sequences inferred by Relate[16] to marginal trees before directly using $\mathrm{CLUES2}$ to infer the evidence of positive selection at the four timepoints[31]. We note that this is only done for unfixed SNPs with $\mathrm{DAF} > 0.2$. Signatures of positive selection were identified in SNPs with $\mathrm{p} \leq 0.001$ and a cut-off of $\mathrm{p} \leq 1\mathrm{e}^{-10}$ was used to identify the SNPs with the strongest evidence of positive selection (**Fig 2**). Across all candidate iron- and calcium-associated genes, we isolate the calcium-associated *ATP2B4* and iron-associated *HIF1A* genes as those with strong evidence for having undergone recent positive selection (~14 kya) and as candidates for adaptation to the changes surrounding the emergence of the Neolithic diet (**Figs S36-44**).

Figures

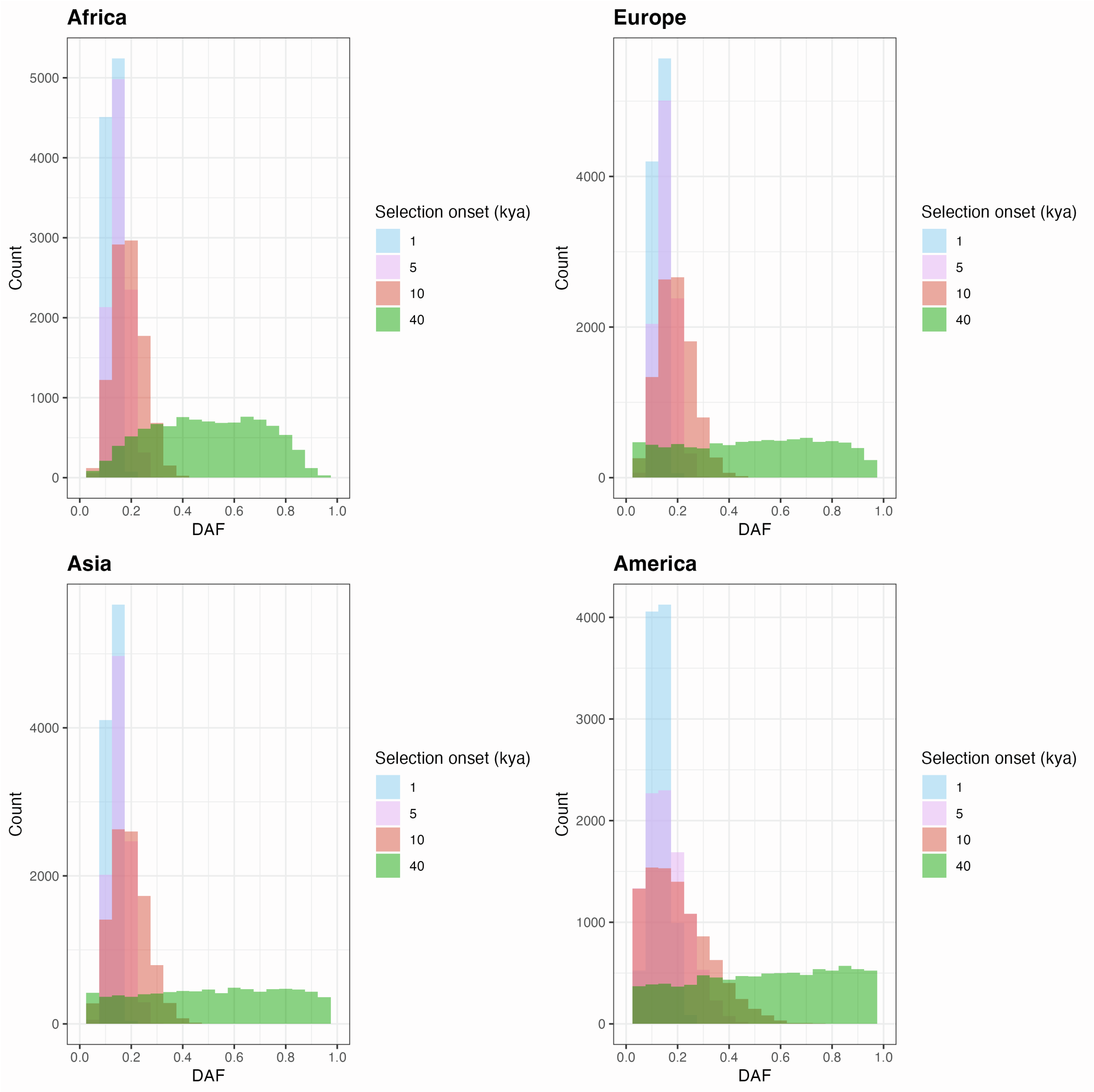

Figure S1: Distribution of derived allele frequency of selected alleles. Distribution of the derived allele frequency (DAF) of selected SNPs (y-axis) when the selection onset is 1 kya, 5 kya, 10 kya or 40 kya (indicated by legend) in four simulated populations (see **Fig. 1**).

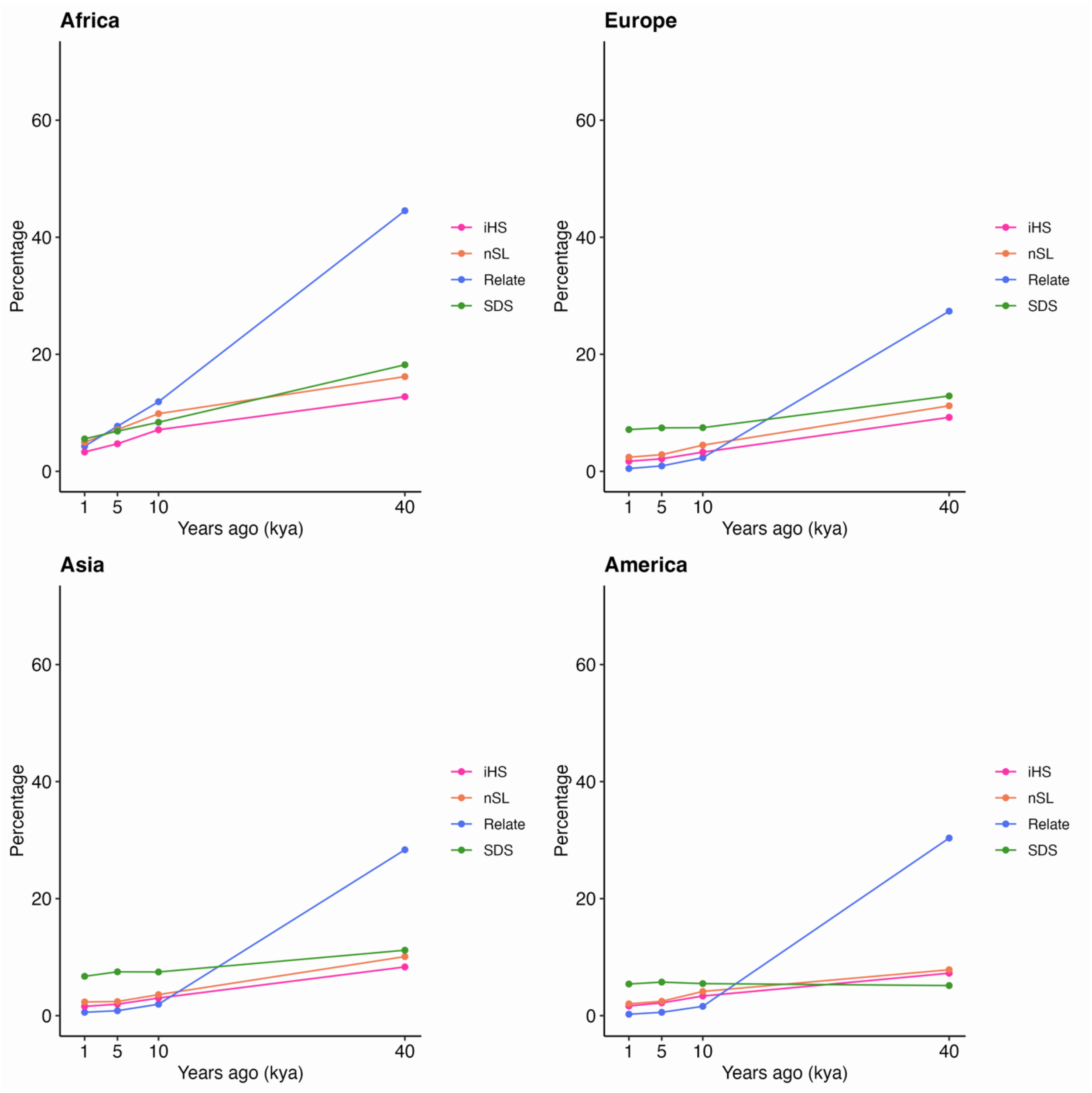

Figure S2: Percentage of selected SNPs identified as under positive selection. Percentage of selected SNPs identified as under positive selection (according to the tail of the neutral distribution; y-axis) at four different timepoints (x-axis) for methods used on focal populations. Shown for four simulated populations (see **Fig. 1**).

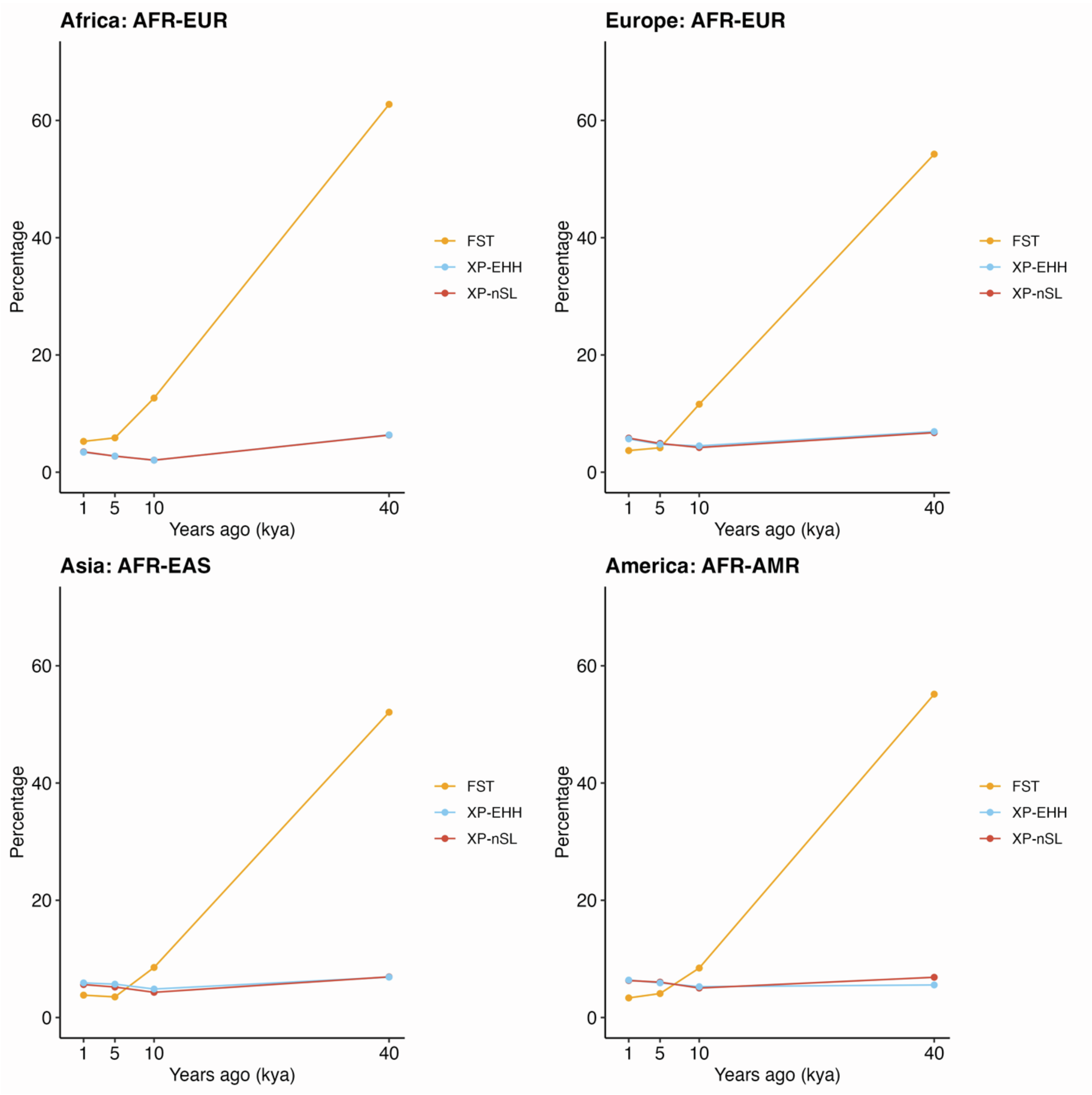

Figure S3: Percentage of selected SNPs identified as under positive selection for cross-population statistics. Percentage of selected SNPs identified as under positive selection (according to the tail of the neutral distribution; y-axis) at four different timepoints (x-axis) for cross-population methods. Shown for four simulated populations (see **Fig. 1**), with cross-population comparisons indicated.

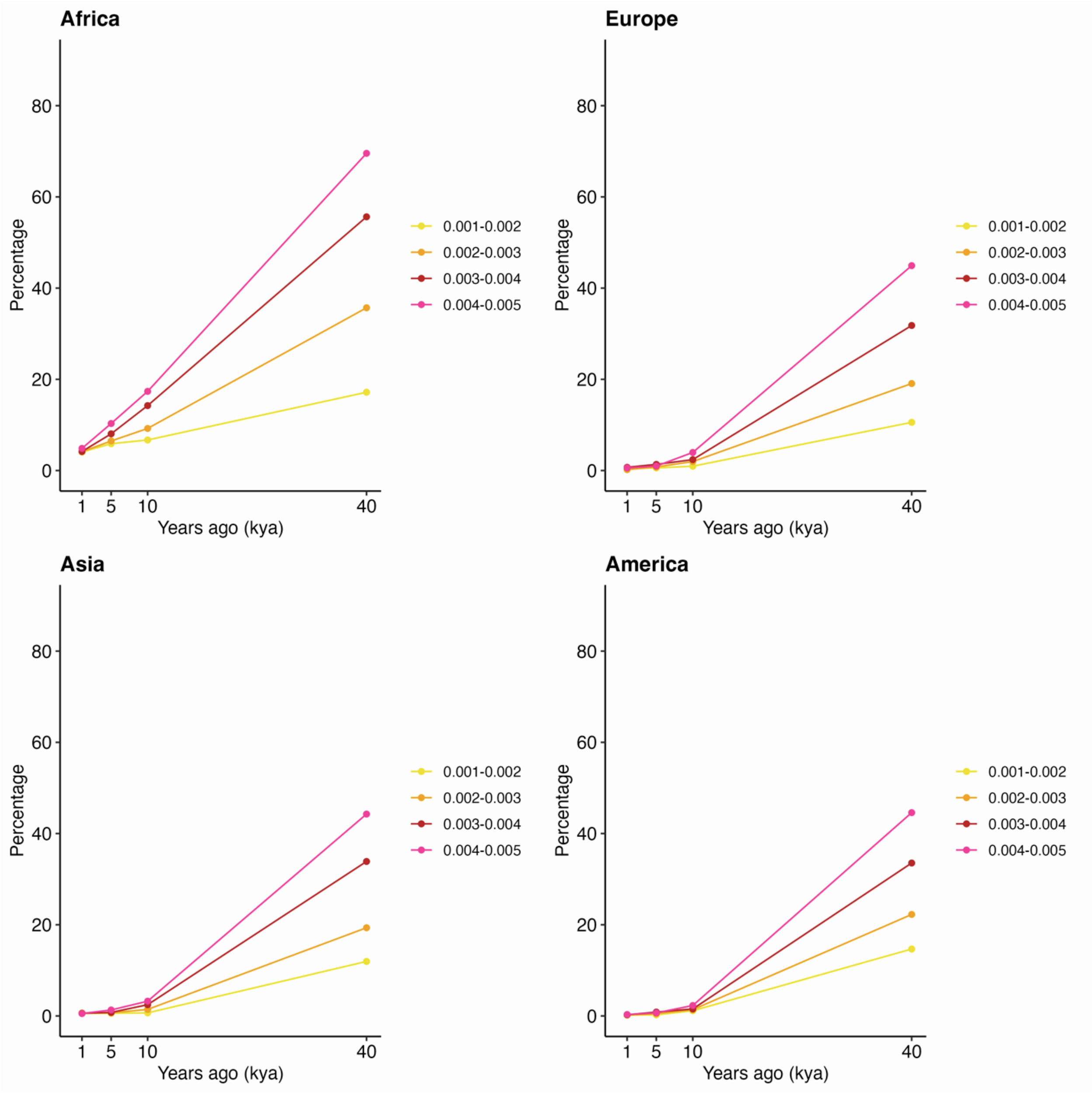

Figure S4: Percentage of selected SNPs identified as under positive selection by Relate by selection coefficient. Percentage of selected SNPs identified as under positive selection by Relate (according to the tail of the neutral distribution; y-axis) at four different timepoints (x-axis) as broken down by the simulated selection coefficient (see legend). Shown for four simulated populations (see **Fig. 1**).

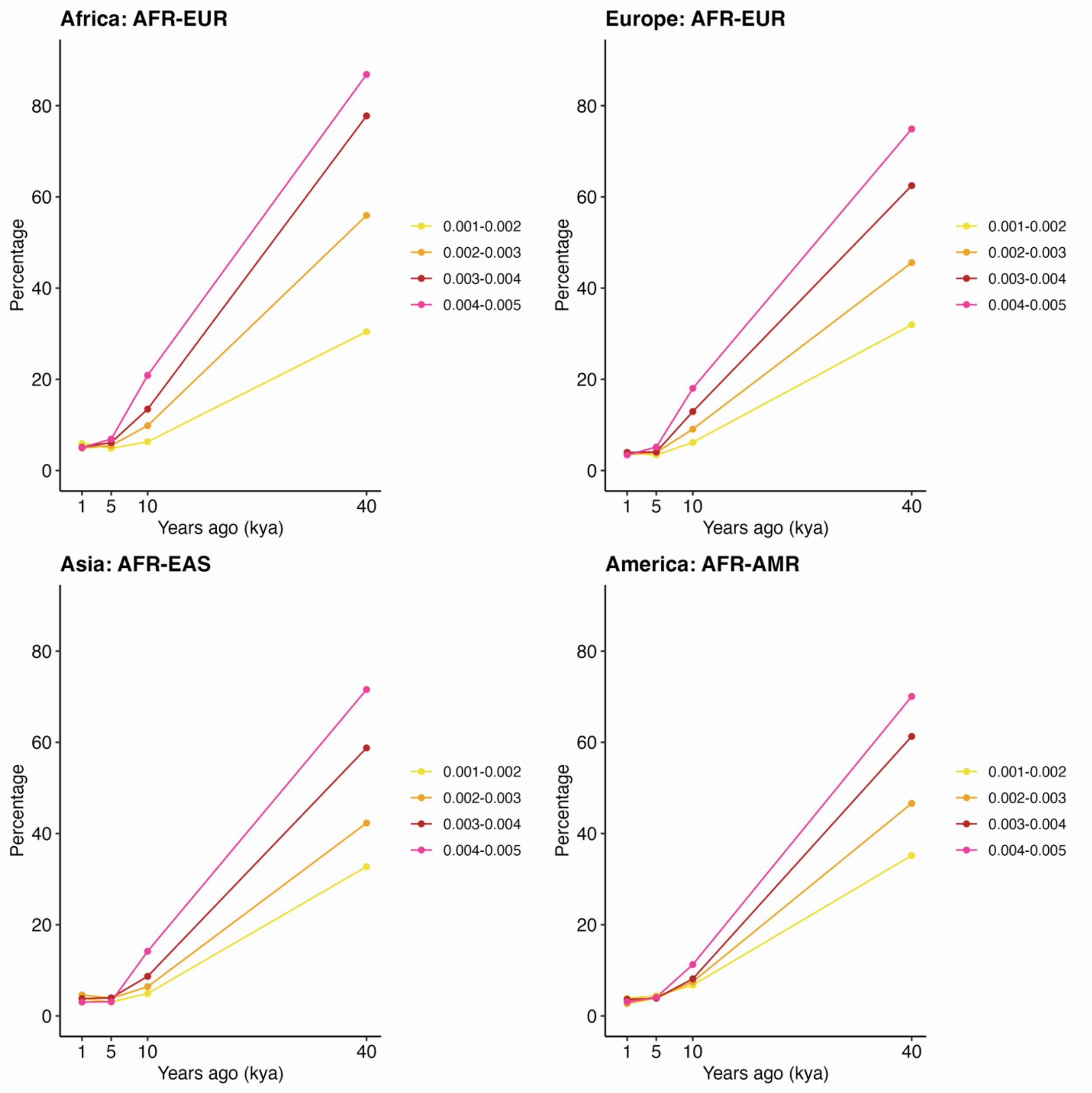

Figure S5: Percentage of selected SNPs identified as under positive selection by F_{ST} by selection coefficient. Percentage of selected SNPs identified as under positive selection by F_{ST} (according to the tail of the neutral distribution; y-axis) at four different timepoints (x-axis) as broken down by the simulated selection coefficient (see legend). Shown for four simulated populations (see **Fig. 1**), with cross-population comparisons indicated.

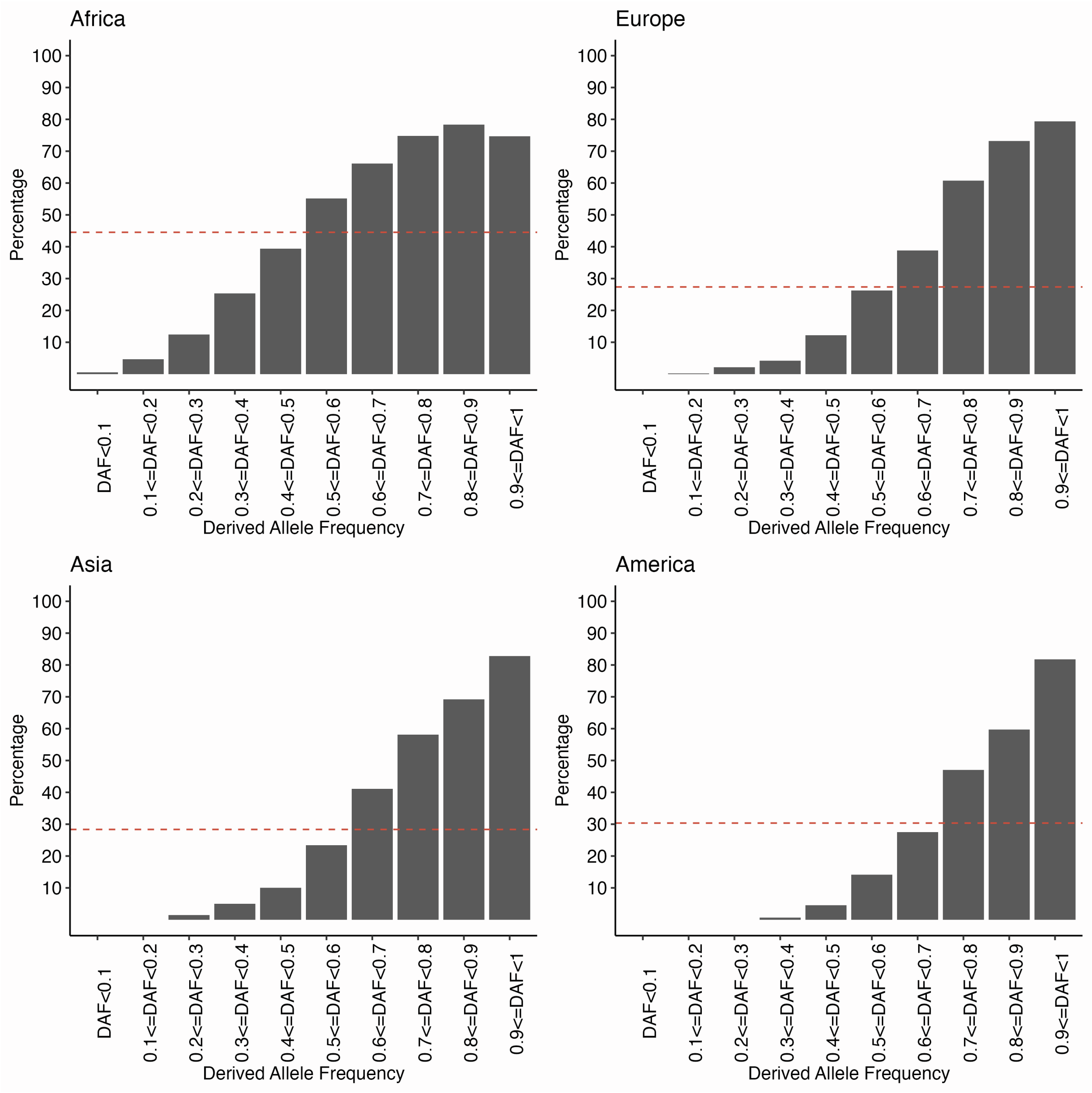

Figure S6: Percentage of selected SNPs identified as under positive selection by Relate by derived allele frequency. Percentage of selected SNPs identified as under positive selection by Relate (according to the tail of the neutral distribution; y-axis) as broken down by derived allele frequency (DAF; x-axis). Dashed line indicates the percentage of selected SNPs identified as under positive selection for all DAF values. Shown for positive selection initiated at 40 kya for four simulated populations (see **Fig. 1**).

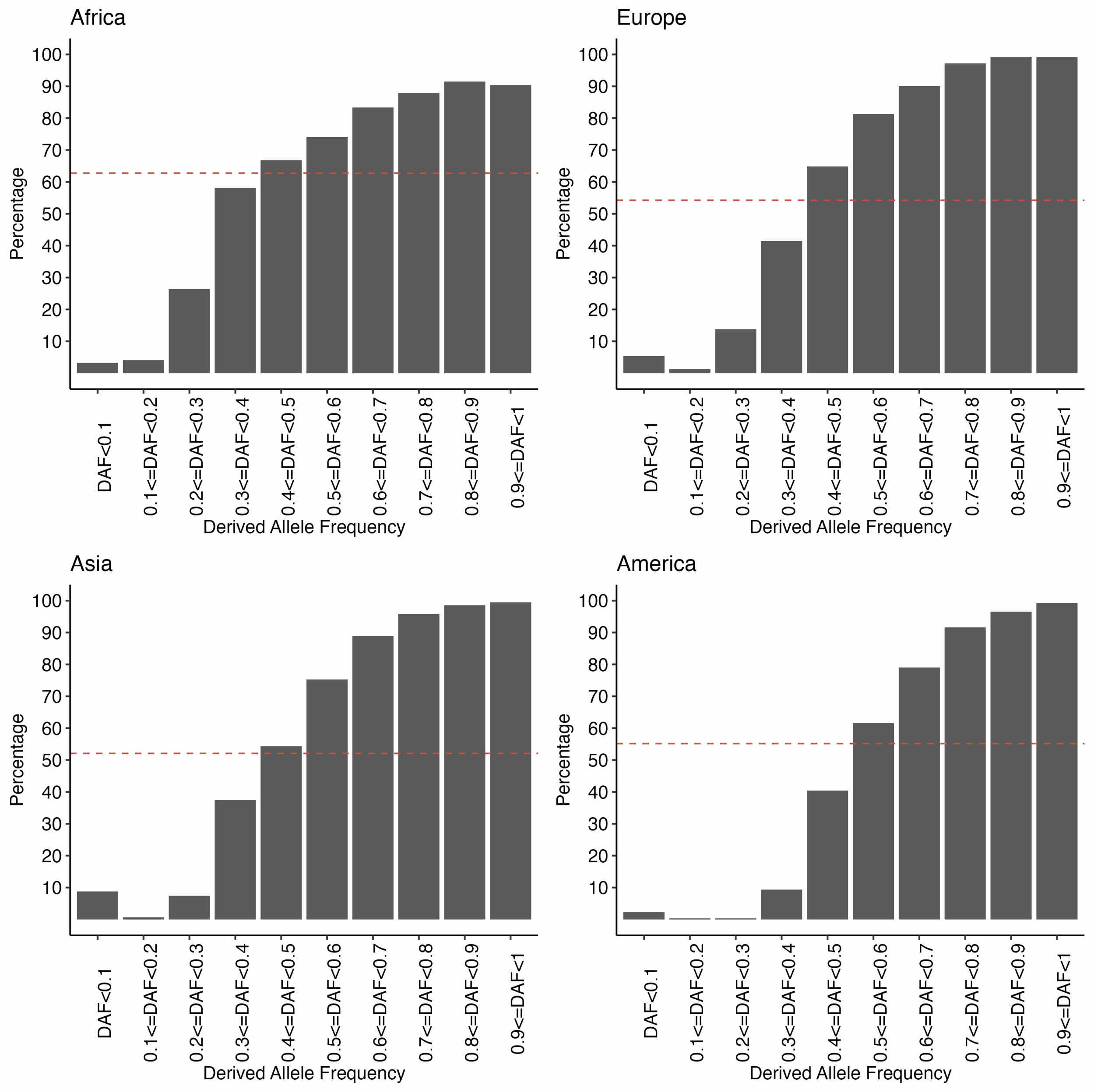

Figure S7: Percentage of selected SNPs identified as under positive selection by F_{ST} by derived allele frequency. Percentage of selected SNPs identified as under positive selection by F_{ST} (according to the tail of the neutral distribution; y-axis) as broken down by derived allele frequency (DAF; x-axis). Dashed line indicates the percentage of selected SNPs identified as under positive selection for all DAF values. Shown for positive selection initiated at 40 kya for four simulated populations (see **Fig. 1**), with cross-population comparisons indicated.

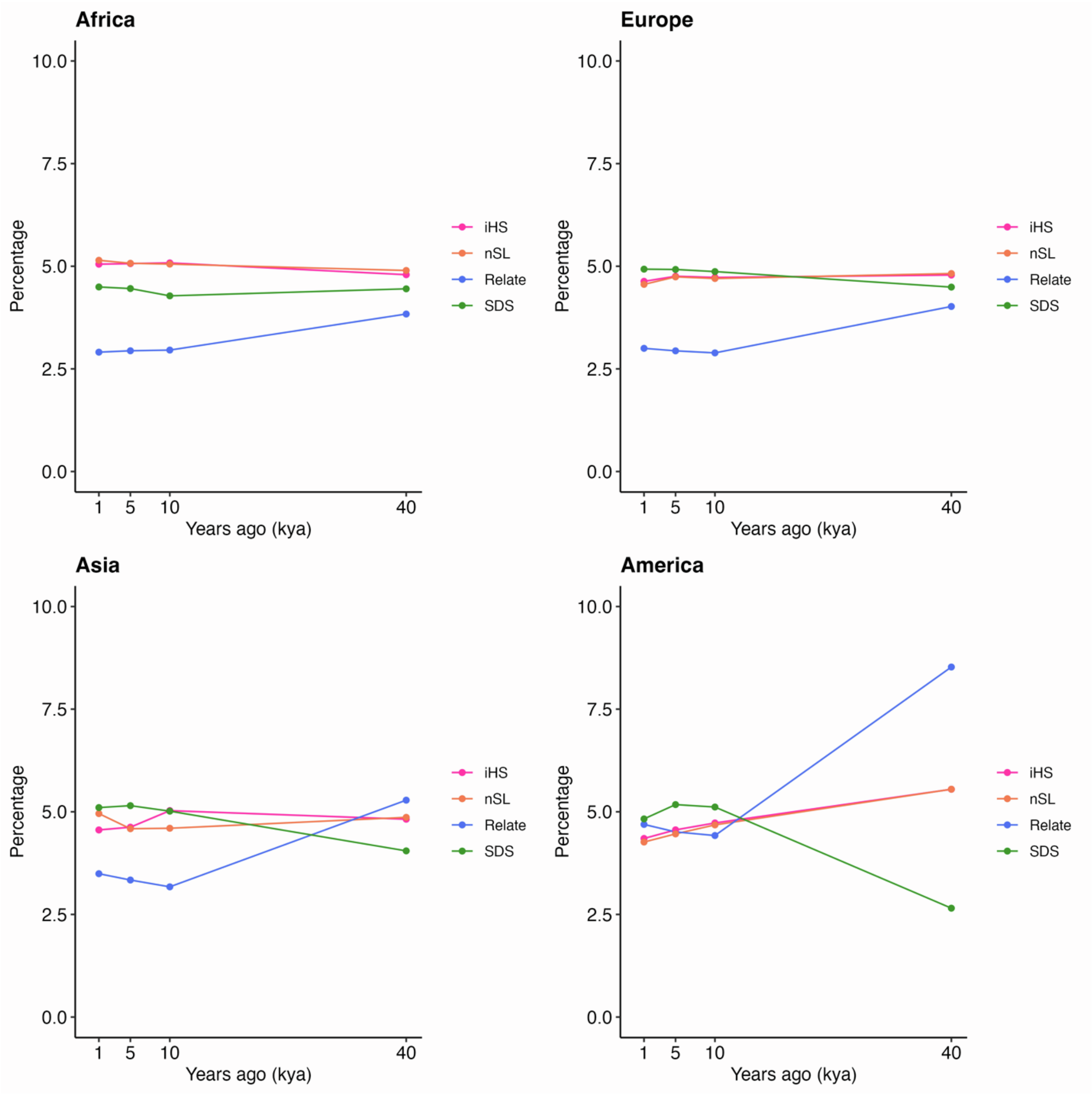

Figure S8: Percentage of non-selected SNPs identified as under positive selection. Percentage of non-selected SNPs identified as under positive selection (according to the tail of the neutral distribution; y-axis) at four different timepoints (x-axis) for methods used on focal populations. Shown for four simulated populations (see **Fig. 1**).

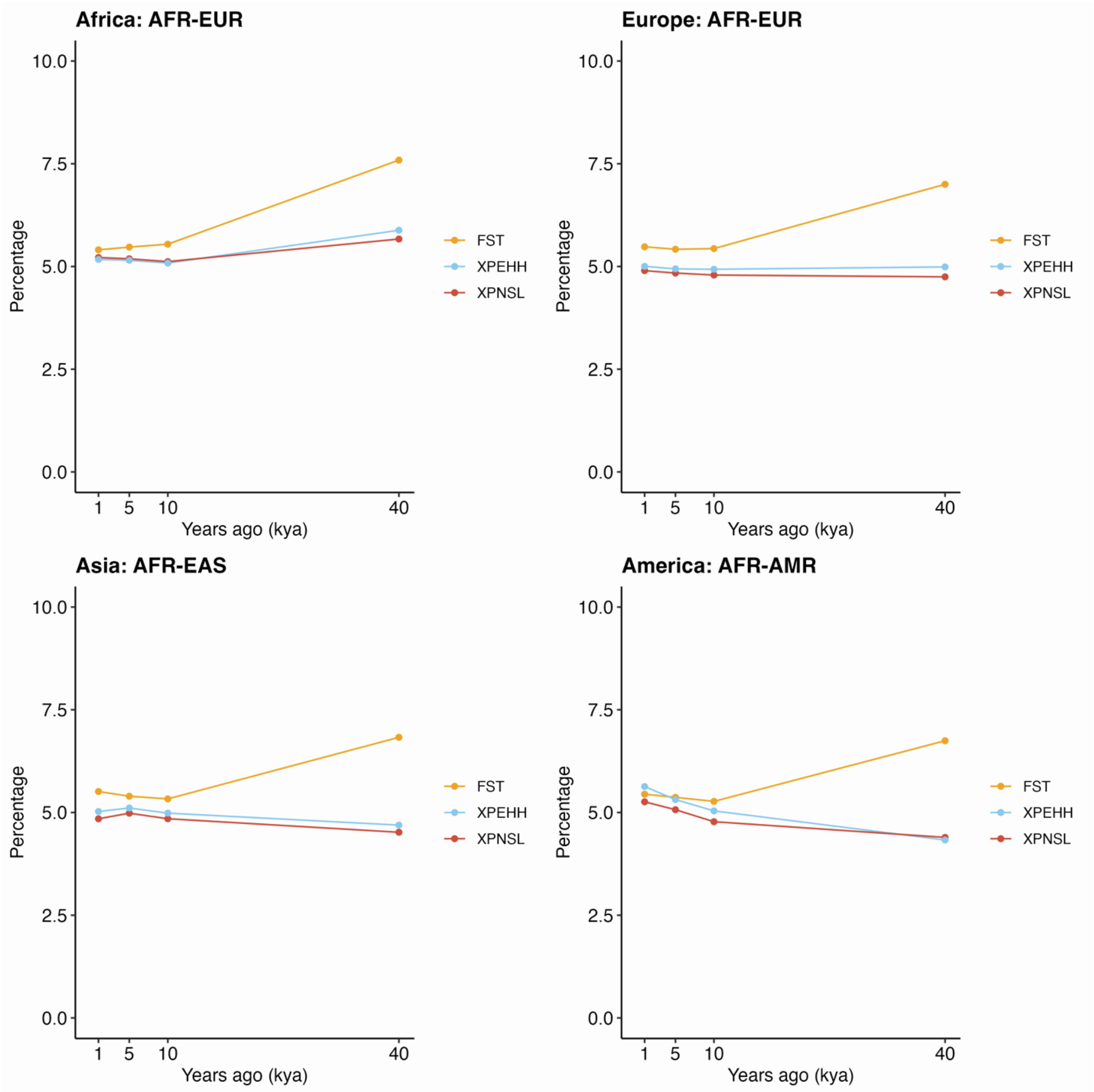

Figure S9: Percentage of non-selected SNPs identified as under positive selection for cross-population methods. Percentage of non-selected SNPs identified as under positive selection (according to the tail of the neutral distribution; y-axis) at four different timepoints (x-axis) for cross-population methods. Shown for four simulated populations (see **Fig. 1**), with cross-population comparisons indicated.

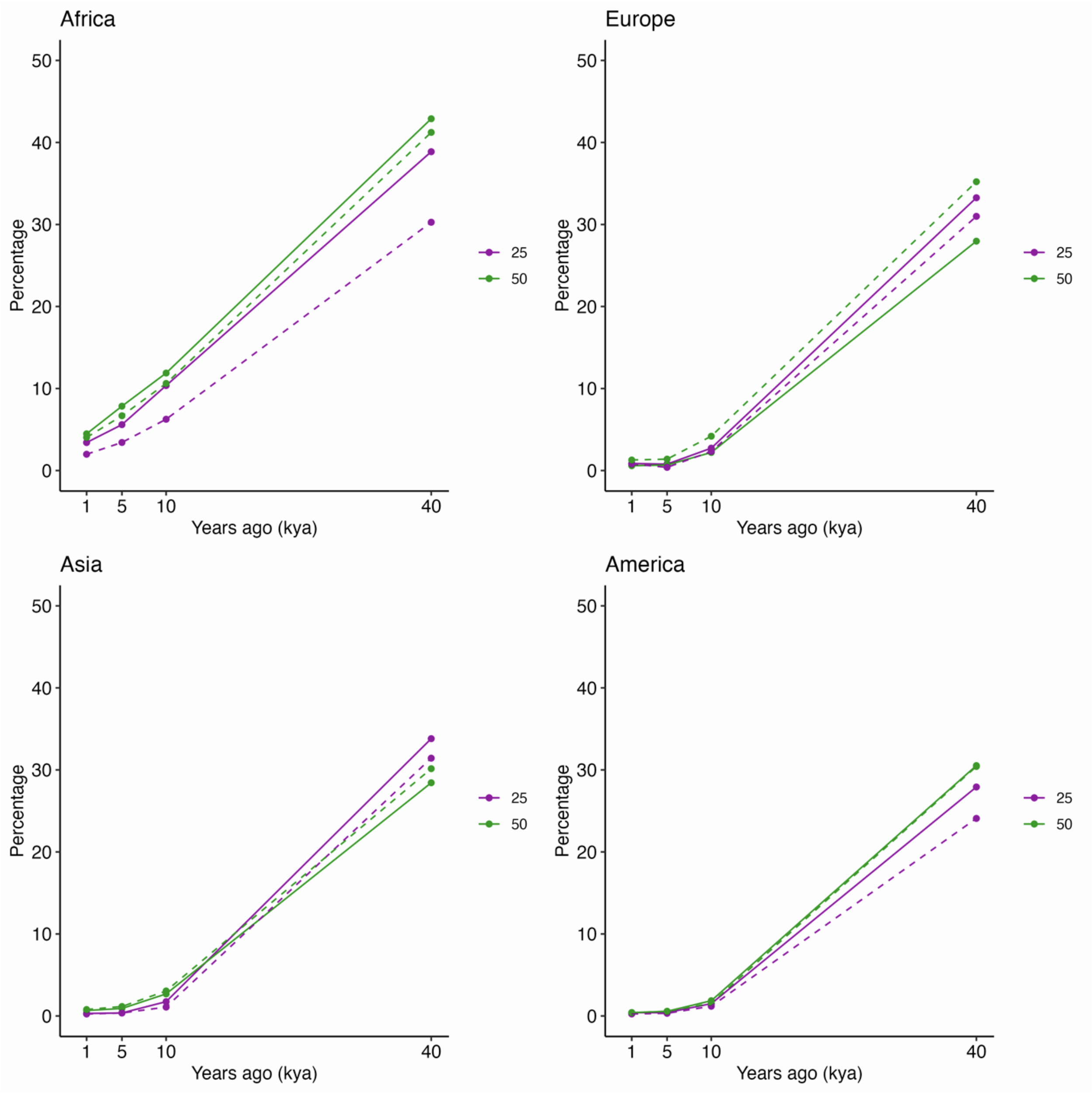

Figure S10: Percentage of selected SNPs identified as under positive selection by differing application of Relate. Percentage of selected SNPs identified as under positive selection (y-axis) by Relate according to either the tail of the neutral distribution (solid lines) or by the raw output of Relate ($-\log 10 \mathrm{p}-\text{value}$; dashed lines) at four different timepoints (x-axis) and for sample sizes of 50 (green) or 25 (purple) simulated individuals. Shown for four simulated populations (see **Fig. 1**)

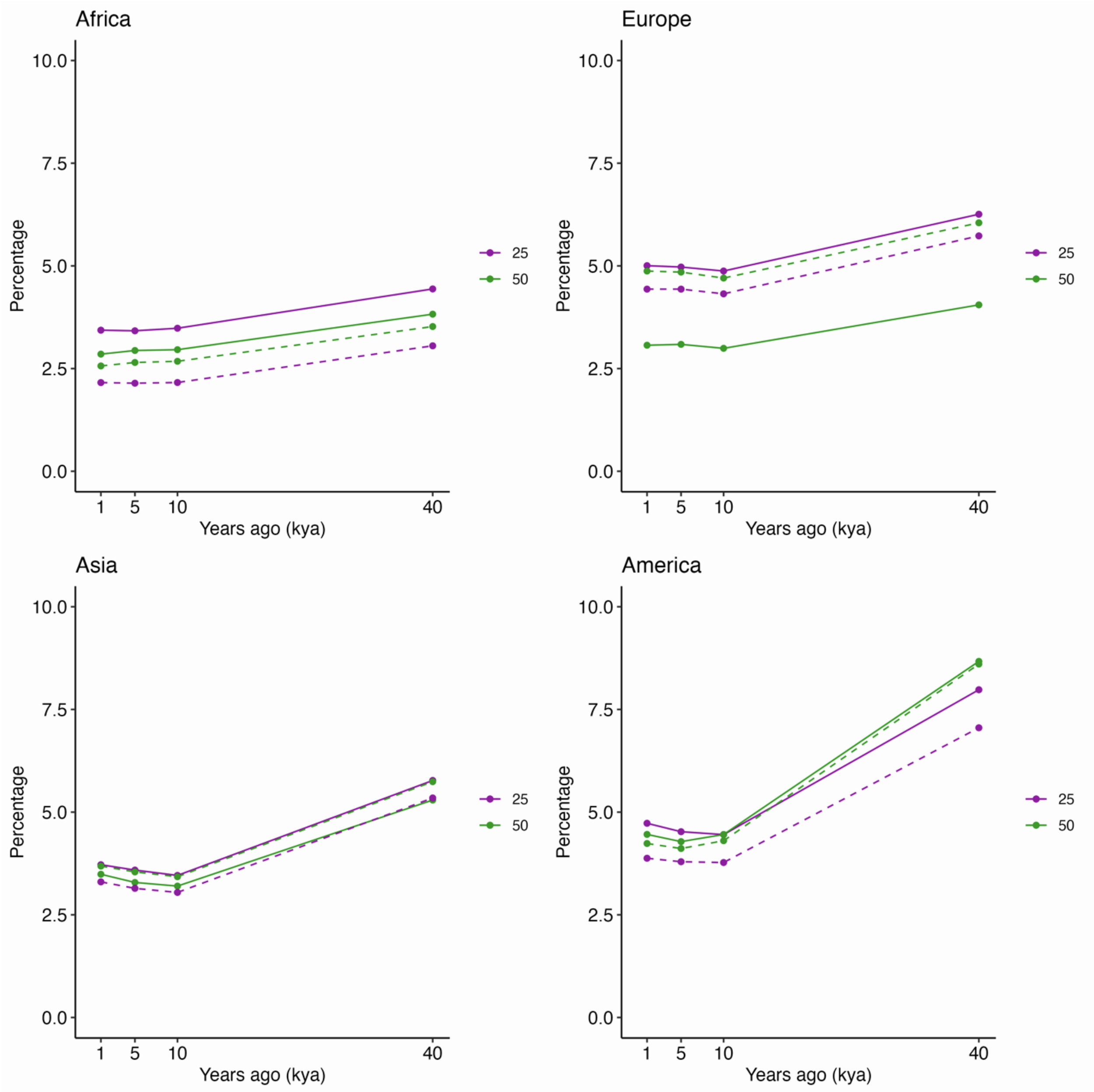

Figure S11: Percentage of non-selected SNPs identified as under positive selection by differing application of Relate. Percentage of non-selected SNPs identified as under positive selection (y-axis) by Relate according to either the tail of the neutral distribution (solid lines) or by the raw output of Relate ($-\log 10\mathrm{p} - \mathrm{value}$; dashed lines) at four different timepoints (x-axis) and for sample sizes of 50 (green) or 25 (purple) simulated individuals. Shown for four simulated populations (see **Fig. 1**)

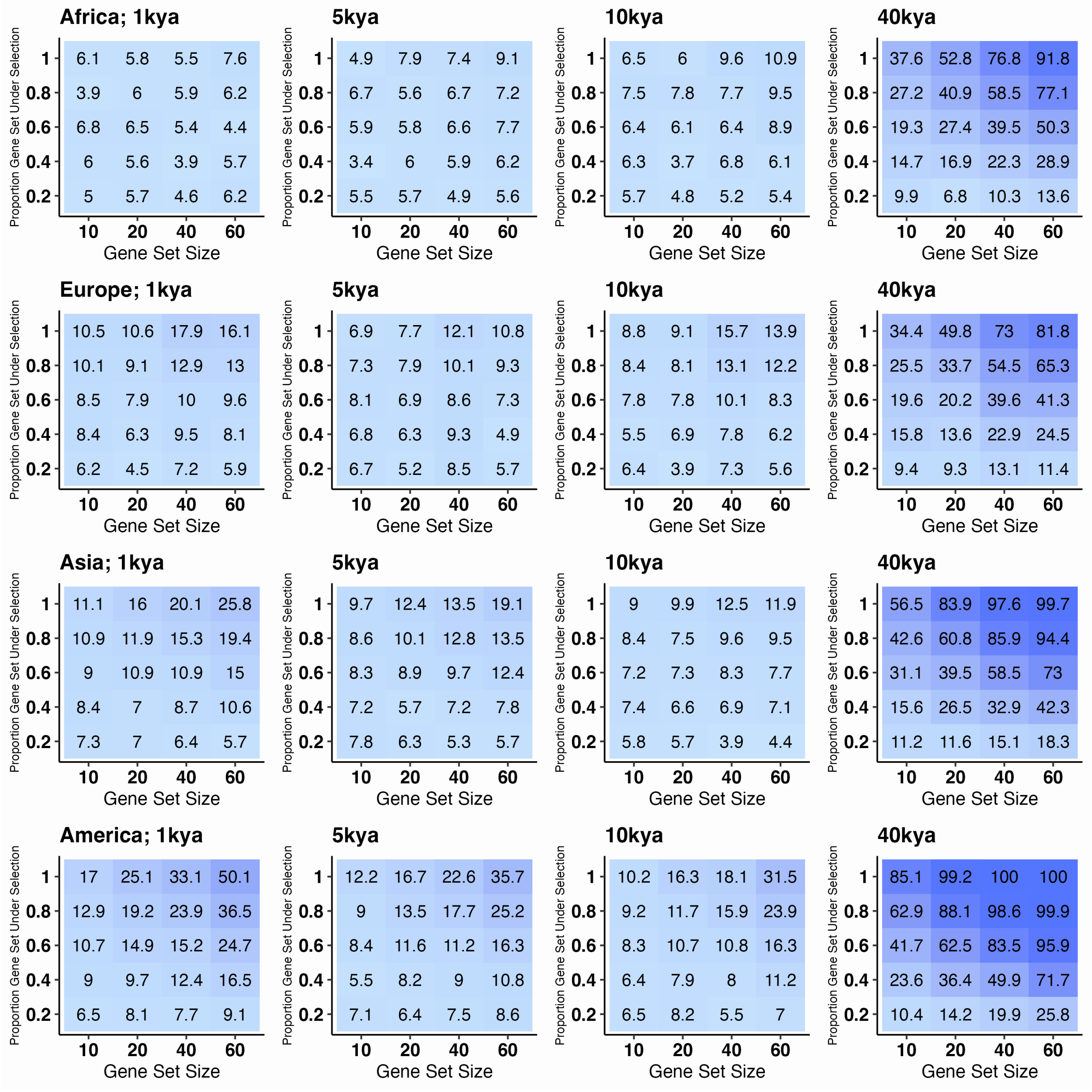

Figure S12: Percentage of selected gene sets identified as under positive selection by Relate. Percentage of selected gene sets identified as under positive selection by Relate (according to the tail of the neutral distribution; numbers inside matrix) for gene sets that vary by size (x-axis) and by proportion of gene regions under positive selection (y-axis). Shown for positive selection simulated in four simulated populations (rows; see **Fig. 1**) at four timepoints (columns).

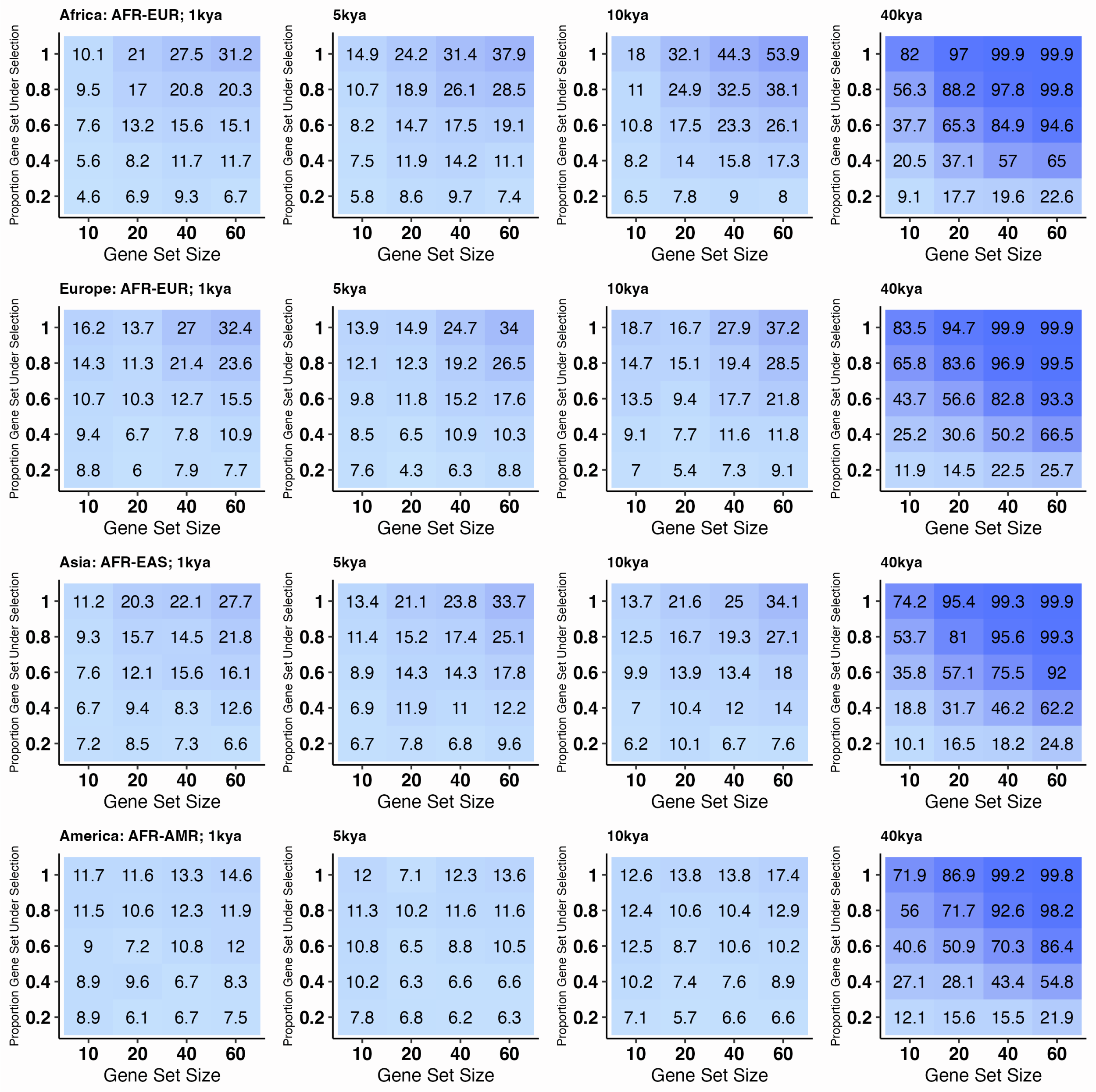

Figure S13: Percentage of selected gene sets identified as under positive selection by F_{ST}. Percentage of selected gene sets identified as under positive selection by F_{ST} (according to the tail of the neutral distribution; numbers inside matrix) for gene sets that vary by size (x-axis) and by proportion of gene regions under positive selection (y-axis). Shown for positive selection simulated in four simulated populations, with cross-population comparisons indicated (rows; see **Fig. 1**), at four timepoints (columns).

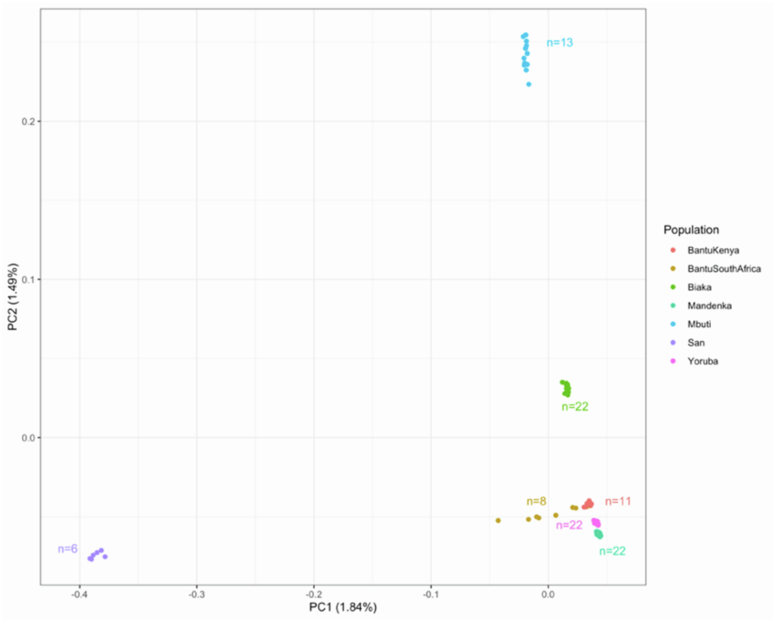

Figure S14: Principal component analysis of African individuals. Principal component analysis of African individuals from[2], showing PC1 and PC2.

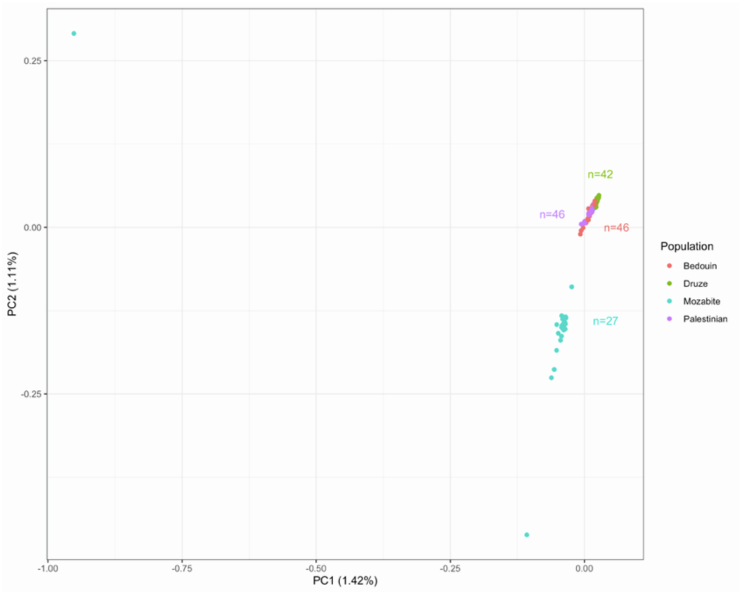

Figure S15: Principal component analysis of Middle-Eastern individuals. Principal component analysis of Middle-eastern individuals from[2], showing PC1 and PC2, having removed outlier individuals

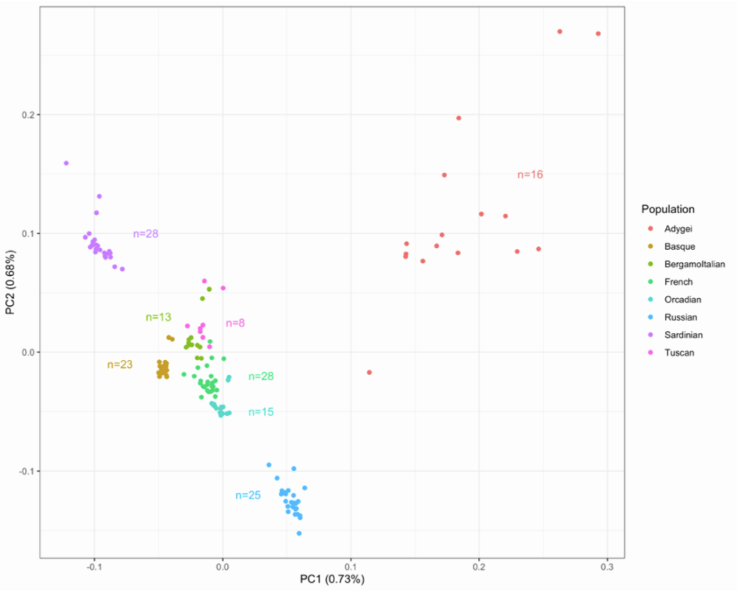

Figure S16: Principal component analysis of European individuals. Principal component analysis of European individuals from[2], showing PC1 and PC2, having removed outlier individuals.

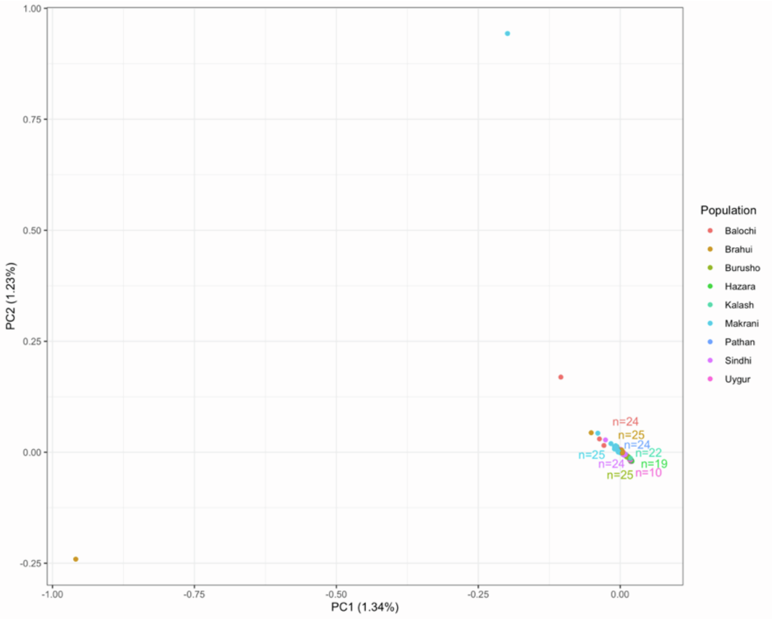

Figure S17: Principal component analysis of Central-South Asian individuals. Principal component analysis of Central-South Asian individuals from[2], showing PC1 and PC2, having removed outlier individuals

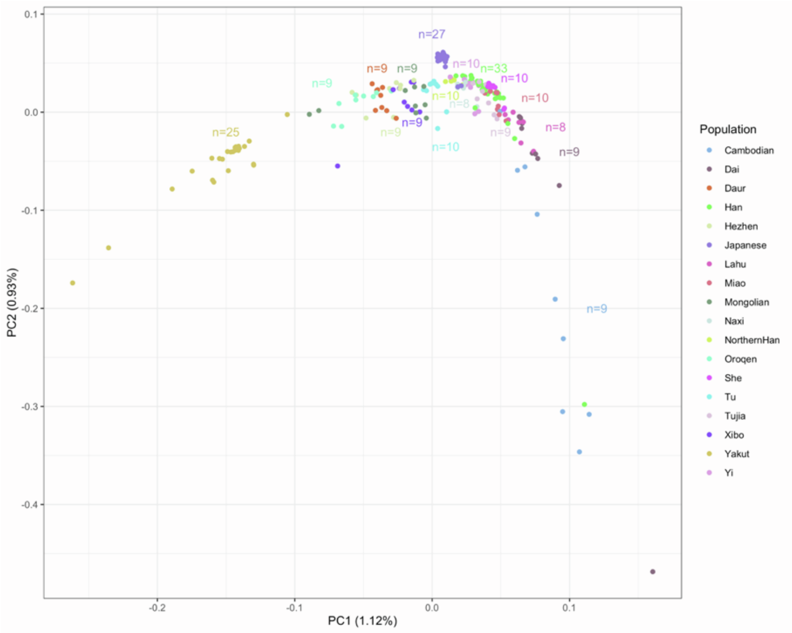

Figure S18: Principal component analysis of East Asian individuals. Principal component analysis of East Asian individuals from[2], showing PC1 and PC2

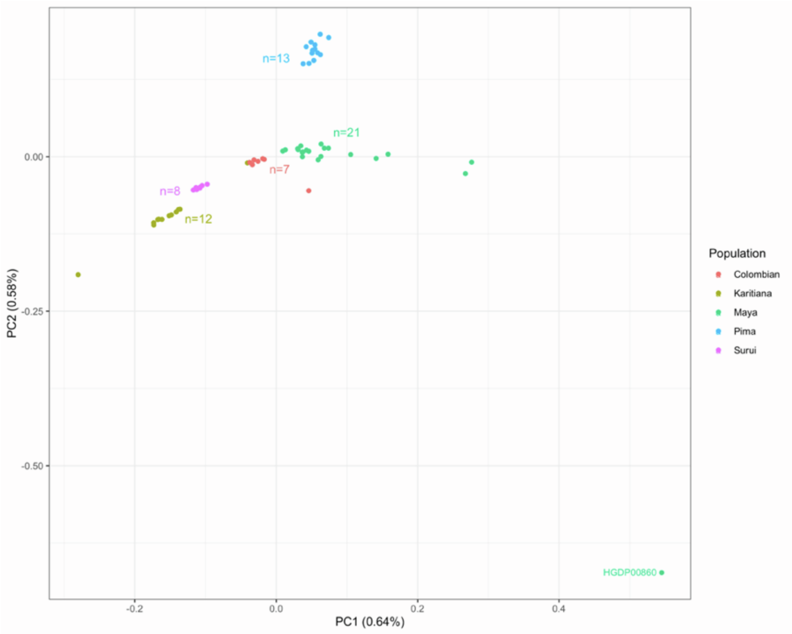

Figure S19: Principal component analysis of American individuals. Principal component analysis of American individuals from[2], showing PC1 and PC2, having removed outlier individuals

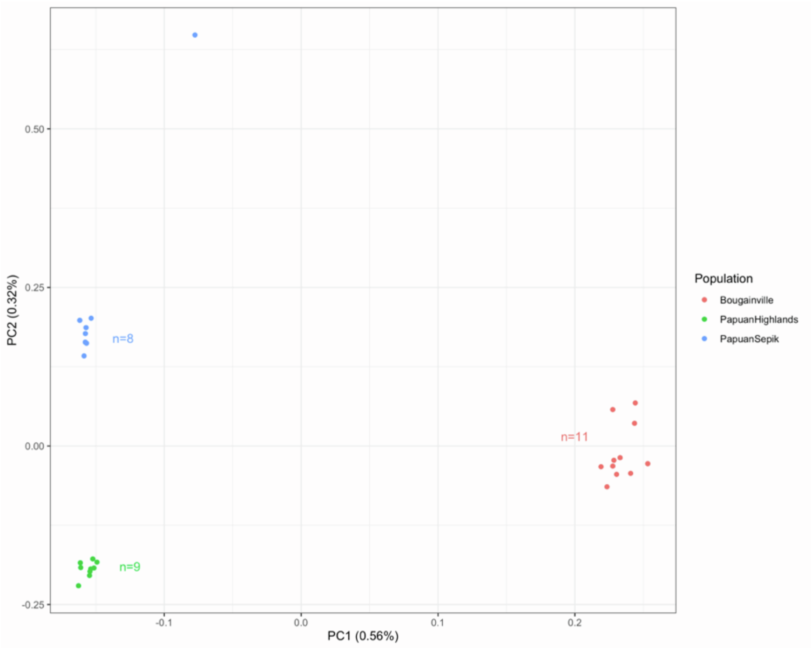

Figure S20: Principal component analysis of Oceanian individuals. Principal component analysis of Oceanic individuals from[2], showing PC1 and PC2

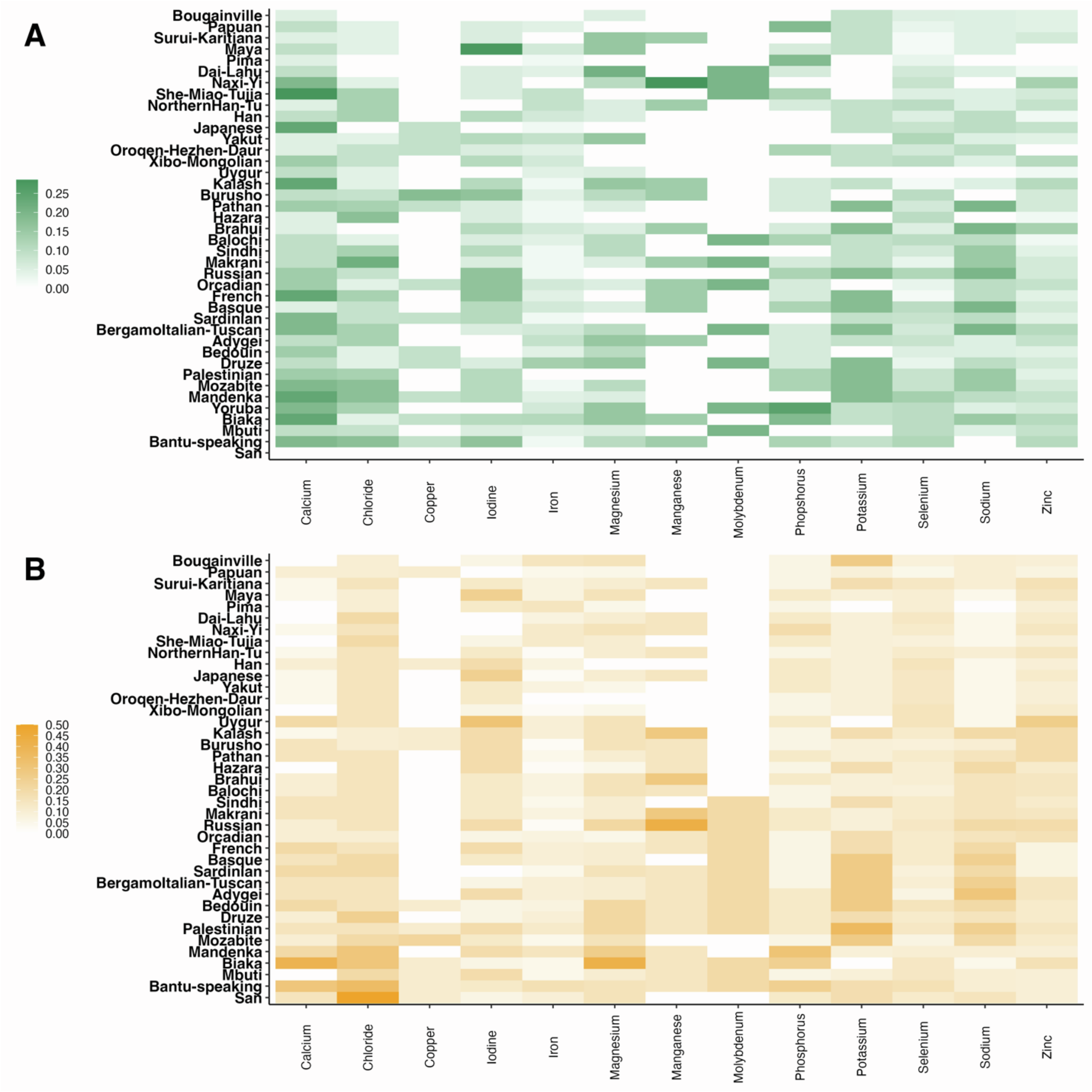

Fig. S21: Proportion of micronutrient gene sets with signatures of positive selection. A and B show the proportion of each micronutrient gene set (including overlap between sets) that have signatures in the 0.1% tail for Relate and F_{ST} selection values, respectively. Key shows the proportion of genes within a gene set with such signatures.

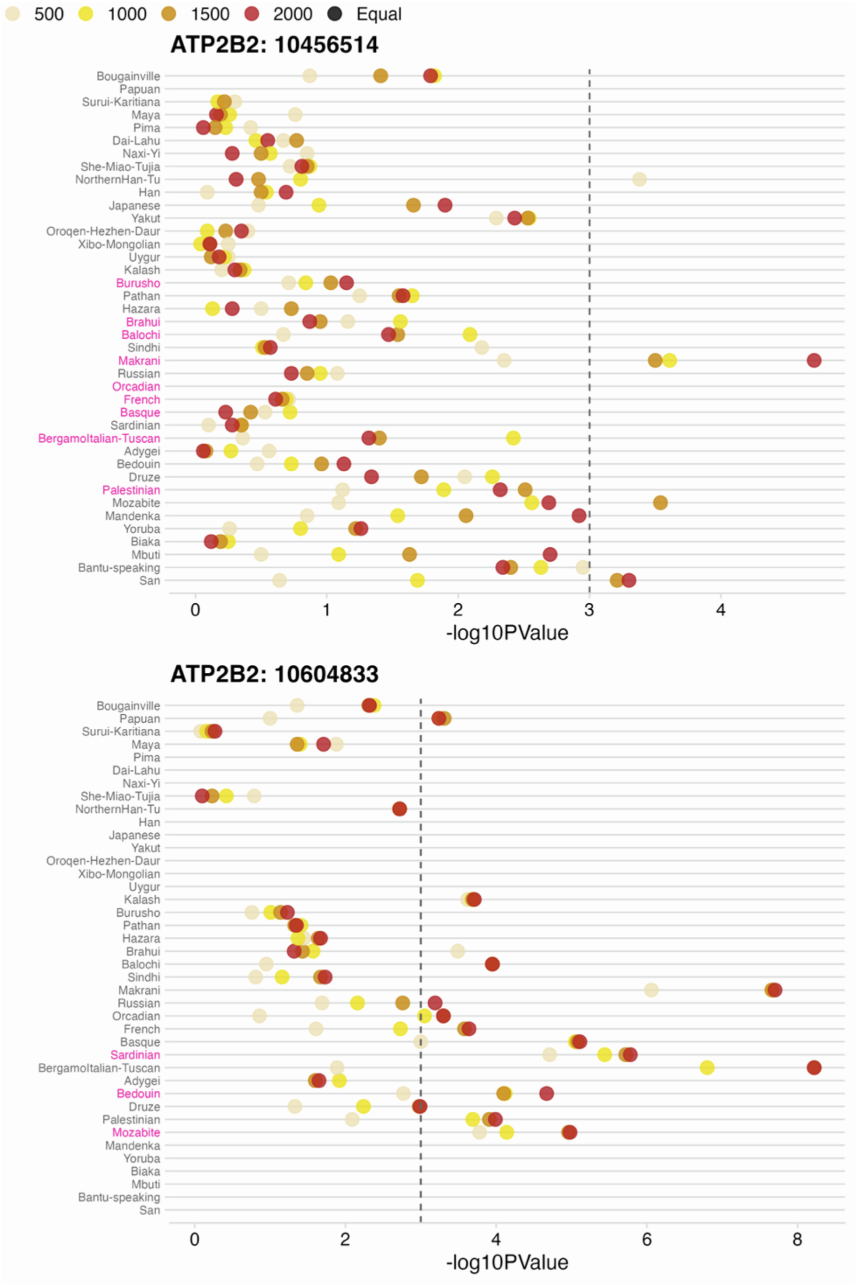

Fig. S22: Evidence for positive selection as inferred by CLUES2 for candidate SNPs in calcium-associated *ATP2B2*. Evidence for positive selection as inferred by CLUES2 for candidate SNPs (positions given) in calcium-associated *ATP2B2* (see **Supp. Note 5** and **Table S6**) across four generational timepoints (see top legend). Dashed vertical line indicates $p = 0.001$. Black points indicate that the evidence for selection is equal across all four tested timepoints. Populations in pink are those with signatures of positive selection ($p \leq 0.001$) identified by Relate or F_{ST}.

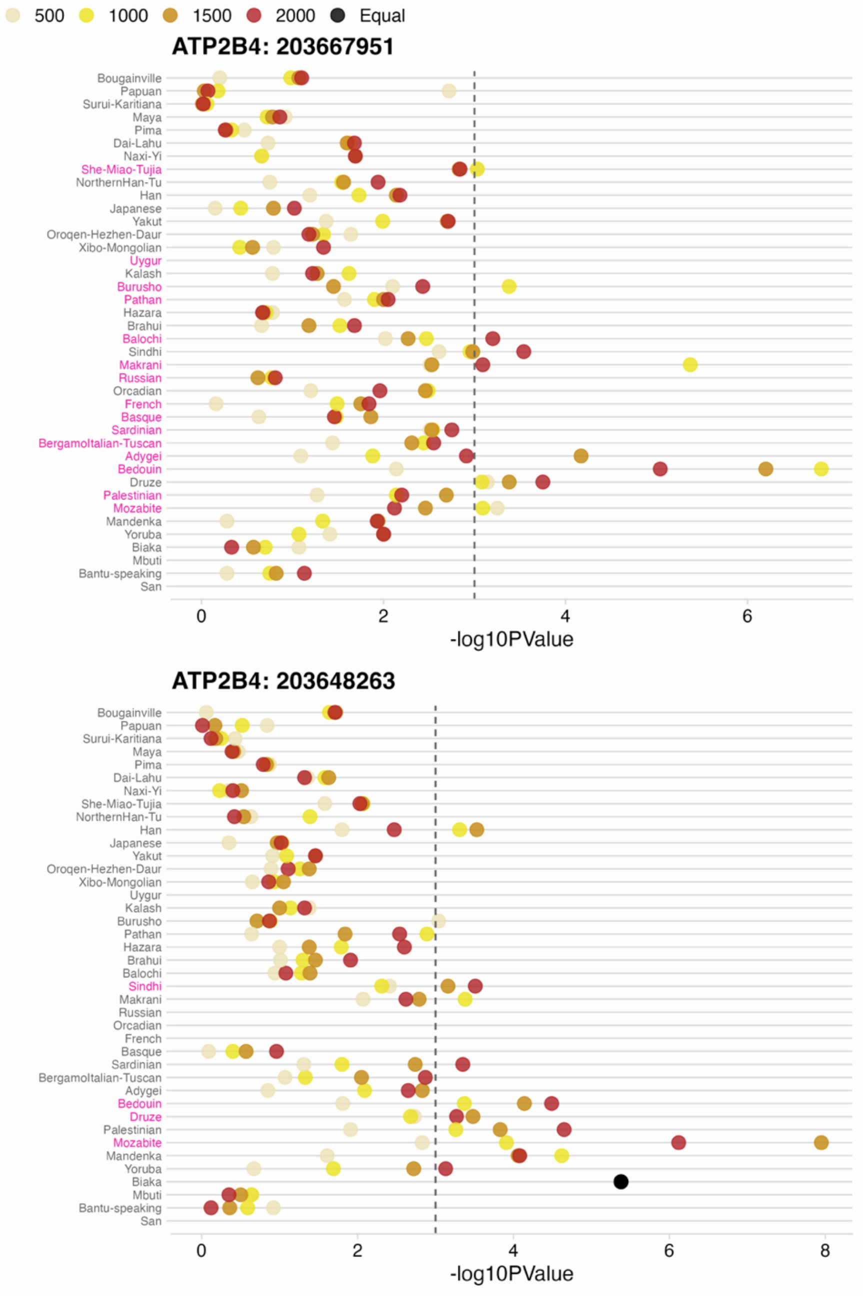

Fig. S23: Evidence for positive selection as inferred by $\mathrm{CLUES2}$ for candidate SNPs in calcium-associated *ATP2B4*. Evidence for positive selection as inferred by $\mathrm{CLUES2}$ for candidate SNPs (positions given) in calcium-associated *ATP2B4* (see **Supp. Note 5** and **Table S6**) across four generational timepoints (see top legend). Dashed vertical line indicates $p = 0.001$. Black points indicate that the evidence for selection is equal across all four tested timepoints. Populations in pink are those with signatures of positive selection ($p \leq 0.001$) identified by Relate or F_{ST}.

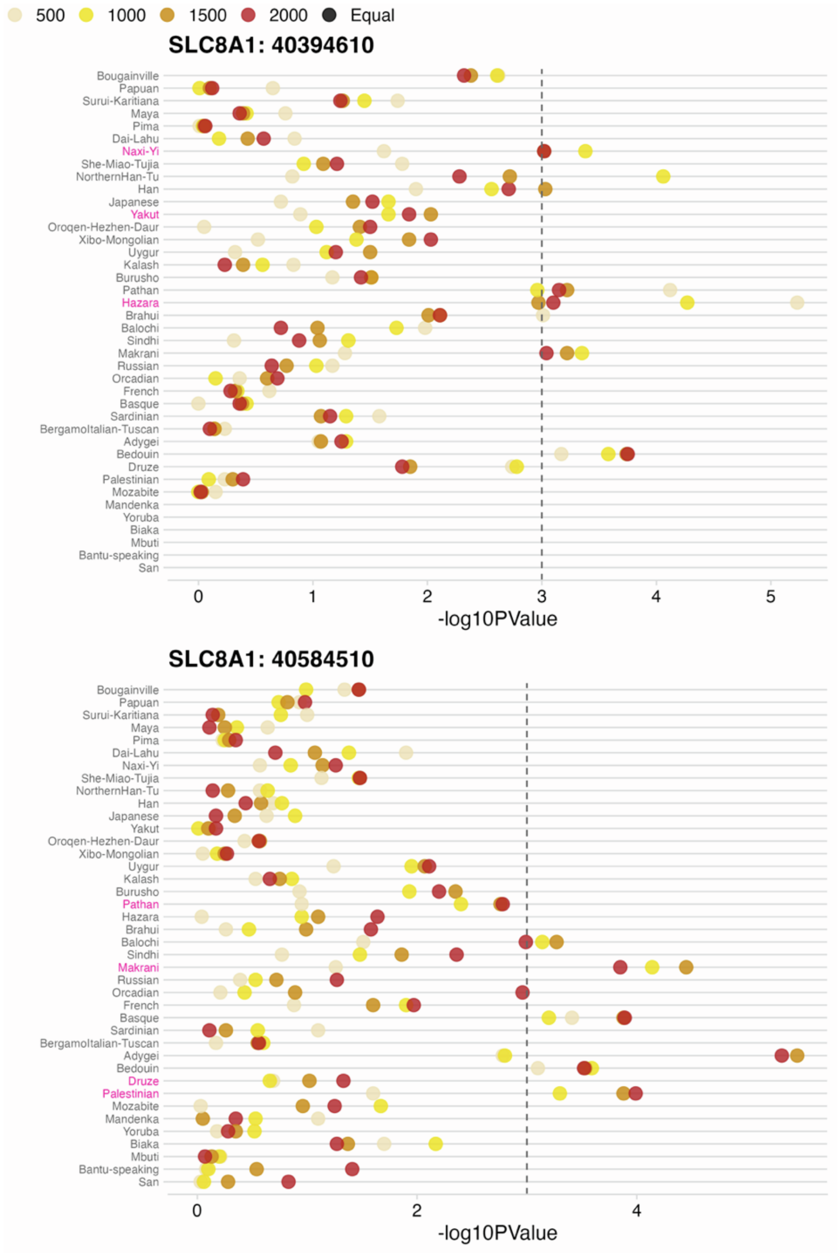

Fig. S24: Evidence for positive selection as inferred by CLUES2 for candidate SNPs in calcium-associated *SLC8A1.* Evidence for positive selection as inferred by CLUES2 for candidate SNPs (positions given) in calcium-associated *SLC8A1* (see **Supp. Note 5** and **Table S6**) across four generational timepoints (see top legend). Dashed vertical line indicates $p = 0.001$. Black points indicate that the evidence for selection is equal across all four tested timepoints. Populations in pink are those with signatures of positive selection ($p \leq 0.001$) identified by Relate or F_{ST}.

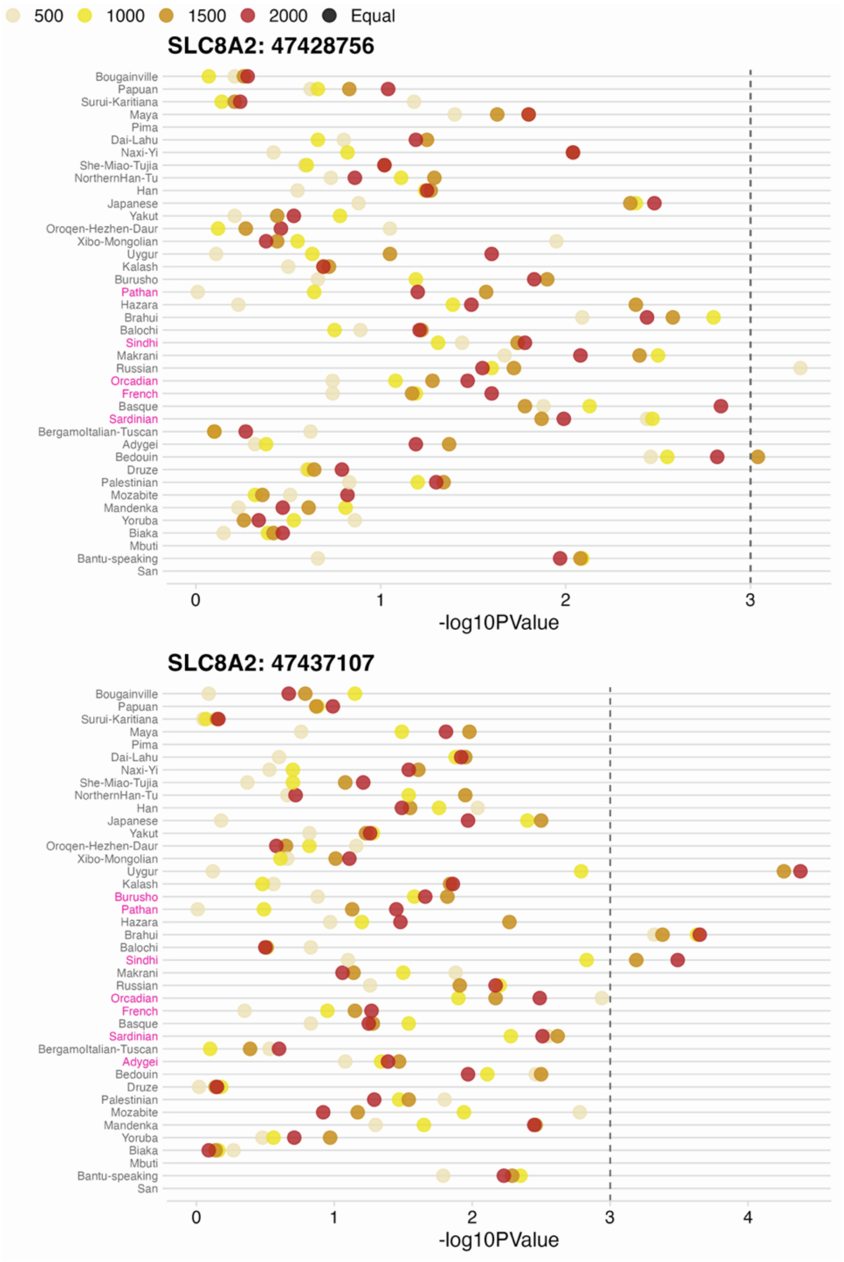

Fig. S25: Evidence for positive selection as inferred by CLUES2 for candidate SNPs in calcium-associated *SLC8A2* Evidence for positive selection as inferred by CLUES2 for candidate SNPs (positions given) in calcium-associated *SLC8A2* (see **Supp. Note 5** and **Table S6**) across four generational timepoints (see top legend). Dashed vertical line indicates $p = 0.001$. Black points indicate that the evidence for selection is equal across all four tested timepoints. Populations in pink are those with signatures of positive selection ($p \leq 0.001$) identified by Relate or F_{ST}.

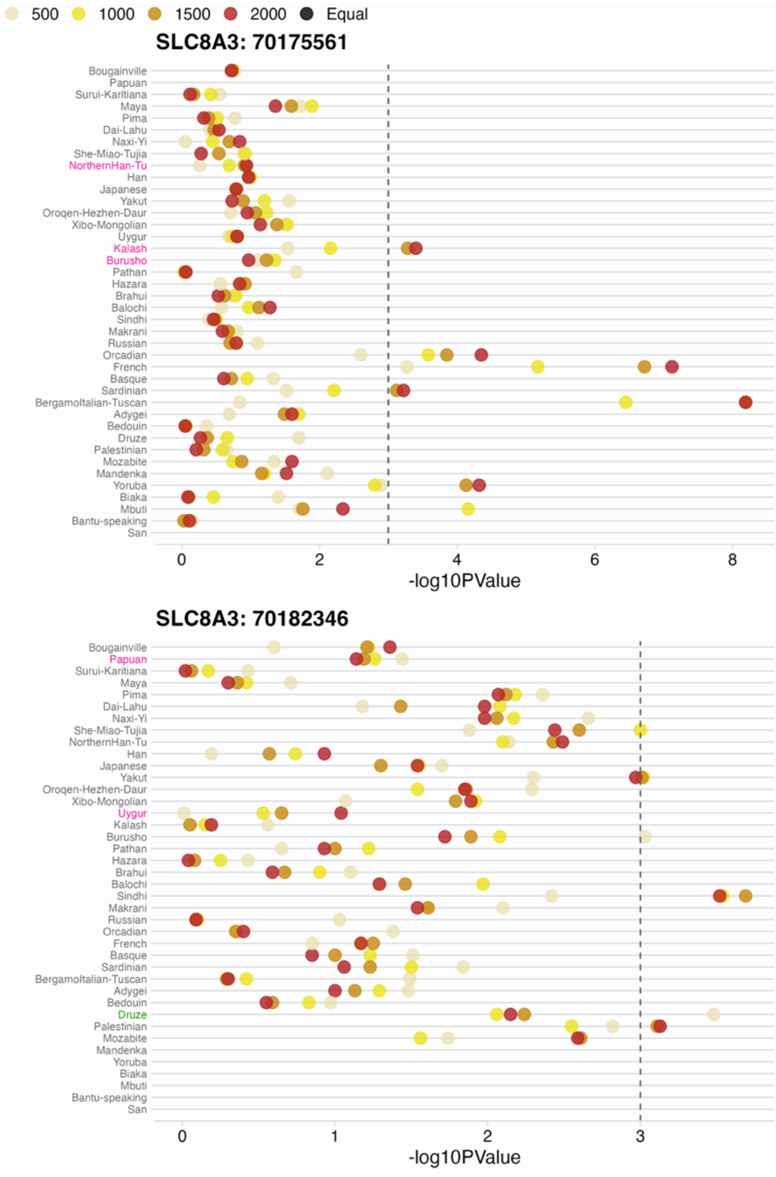

Fig. S26: Evidence for positive selection as inferred by CLUES2 for candidate SNPs in calcium-associated *SLC8A3*. Evidence for positive selection as inferred by CLUES2 for candidate SNPs (positions given) in calcium-associated *SLC8A3* (see **Supp. Note 5** and **Table S6**) across four generational timepoints (see top legend). Dashed vertical line indicates $p = 0.001$. Black points indicate that the evidence for selection is equal across all four tested timepoints. Populations in pink are those with signatures of positive selection ($p \leq 0.001$) identified by Relate or F_{ST}.

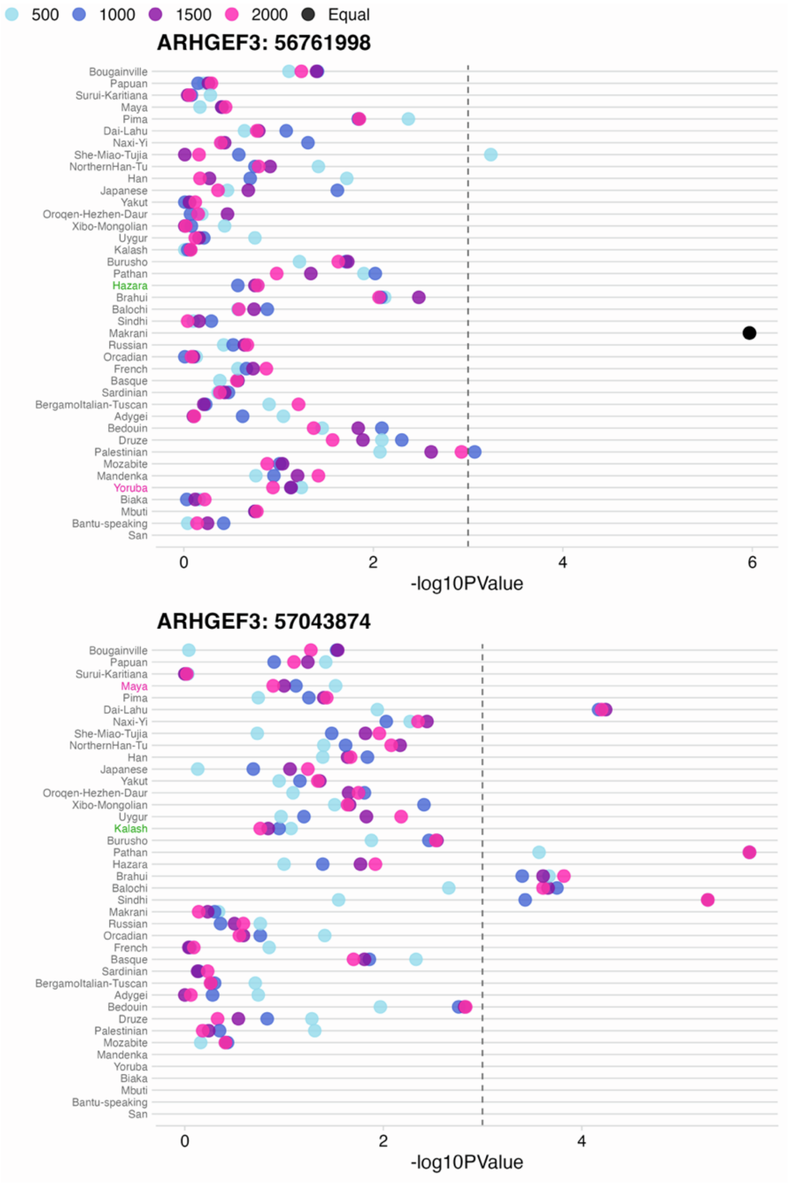

Fig. S27: Evidence for positive selection as inferred by CLUES2 for candidate SNPs in calcium-associated *ARHGEF3*. Evidence for positive selection as inferred by CLUES2 for candidate SNPs (positions given) in iron-associated *ARHGEF3* (see **Supp. Note 5** and **Table S6**) across four generational timepoints (see top legend). Dashed vertical line indicates $p = 0.001$. Black points indicate that the evidence for selection is equal across all four tested timepoints. Populations in pink are those with signatures of positive selection ($p \leq 0.001$) identified by Relate or F_{ST}.

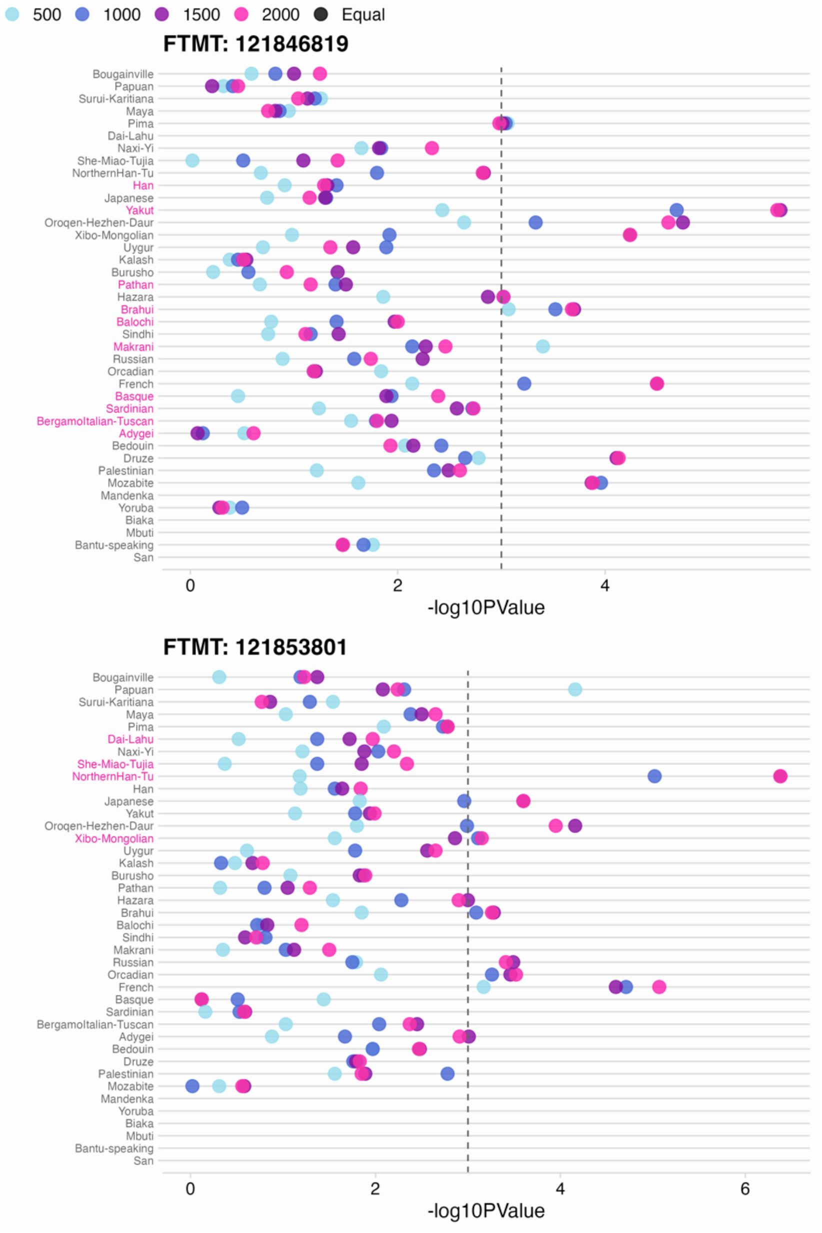

Fig. S28: Evidence for positive selection as inferred by CLUES2 for candidate SNPs in calcium-associated *FTMT*. Evidence for positive selection as inferred by CLUES2 for candidate SNPs (positions given) in iron-associated *FTMT* (see **Supp. Note 5** and **Table S6**) across four generational timepoints (see top legend). Dashed vertical line indicates $p = 0.001$. Black points indicate that the evidence for selection is equal across all four tested timepoints. Populations in pink are those with signatures of positive selection ($p \leq 0.001$) identified by Relate or F_{ST}.

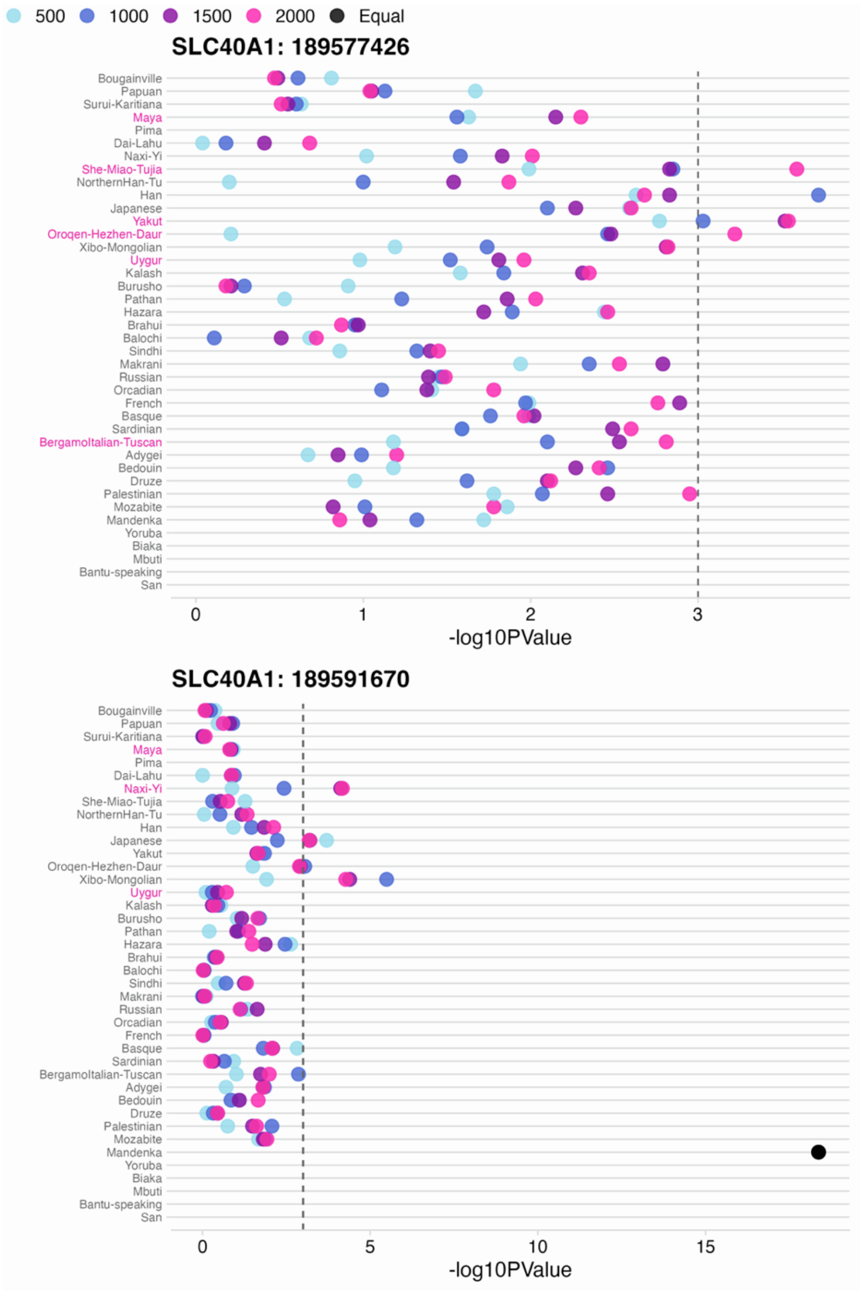

Fig. S29: Evidence for positive selection as inferred by CLUES2 for candidate SNPs in calcium-associated *SLC40A1*. Evidence for positive selection as inferred by CLUES2 for candidate SNPs (positions given) in iron-associated *SLC40A1* (see **Supp. Note 5** and **Table S6**) across four generational timepoints (see top legend). Dashed vertical line indicates $p = 0.001$. Black points indicate that the evidence for selection is equal across all four tested timepoints. Populations in pink are those with signatures of positive selection ($p \leq 0.001$) identified by Relate or F_{ST}.

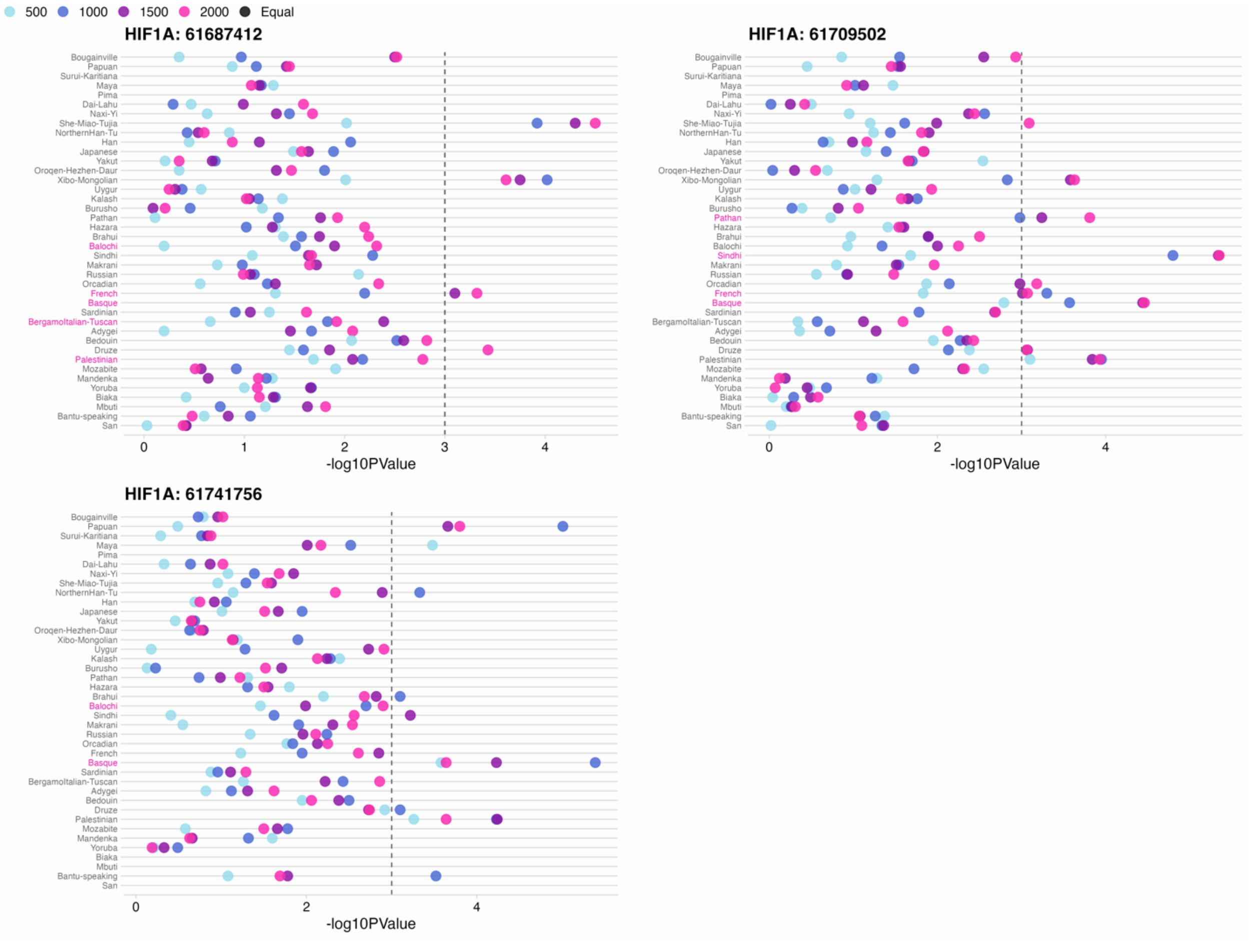

Fig. S30: Evidence for positive selection as inferred by $\mathrm{CLUES2}$ for candidate SNPs in calcium-associated *HIF1A*. Evidence for positive selection as inferred by $\mathrm{CLUES2}$ for candidate SNPs (positions given) in iron-associated *HIF1A* (see **Supp. Note 5** and **Table S6**) across four generational timepoints (see top legend). Dashed vertical line indicates $\mathrm{p} = 0.001$. Black points indicate that the evidence for selection is equal across all four tested timepoints. Populations in pink are those with signatures of positive selection ($\mathrm{p} \leq 0.001$) identified by Relate or $\mathrm{F_{ST}}$.

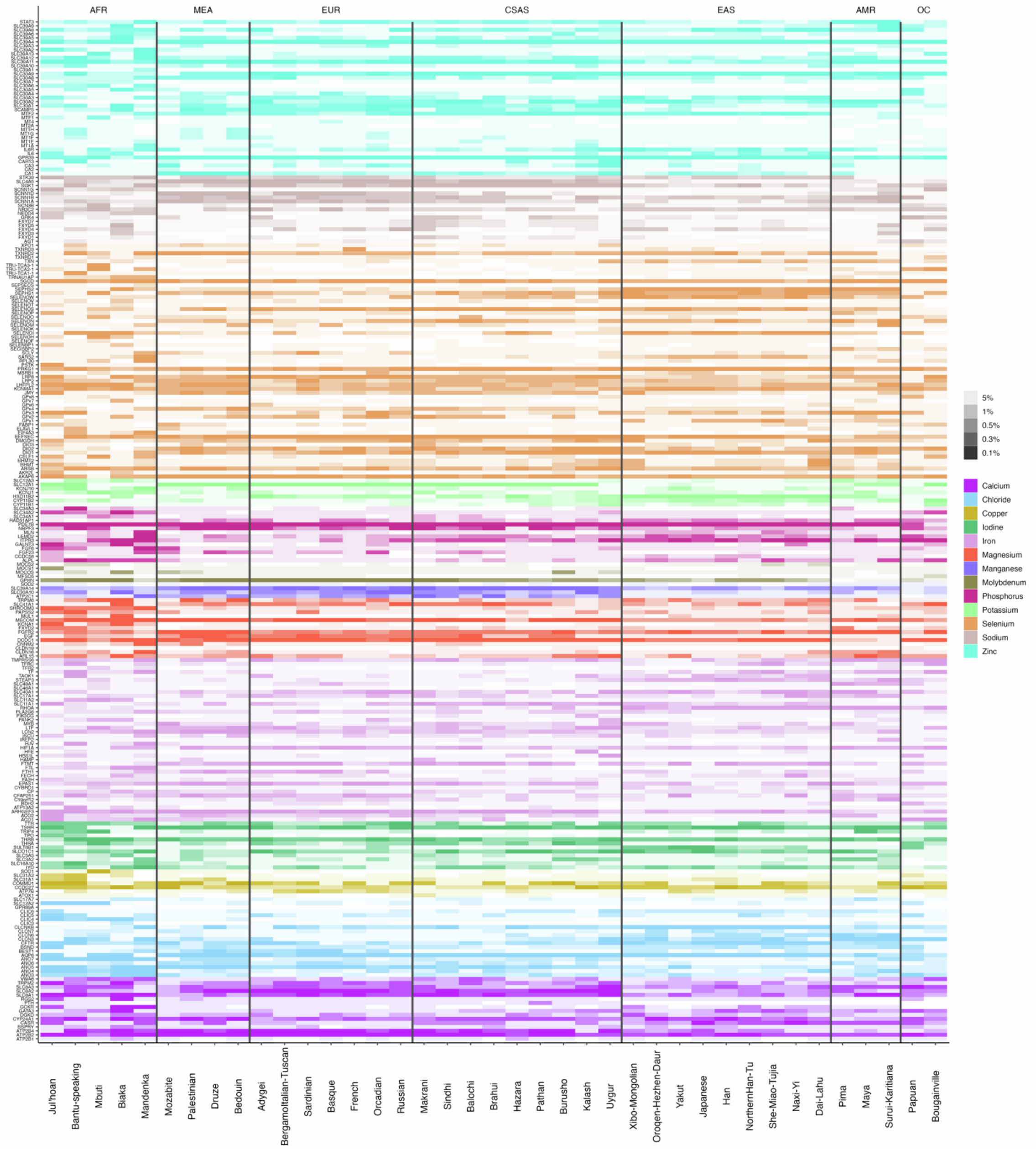

Figure S31: Signatures of positive selection as inferred by F_{ST} across all autosomal MA-genes. Signatures of positive selection as inferred by F_{ST} across all autosomal MA-genes (Y-axis, coloured by micronutrient) and all populations (X-axis, grouped by metapopulation). Darker blocks reflect lower empirical p-values (from lightest to darkest: below 5%, 1%, 0.5%, 0.3%, 0.1%, see left legend).

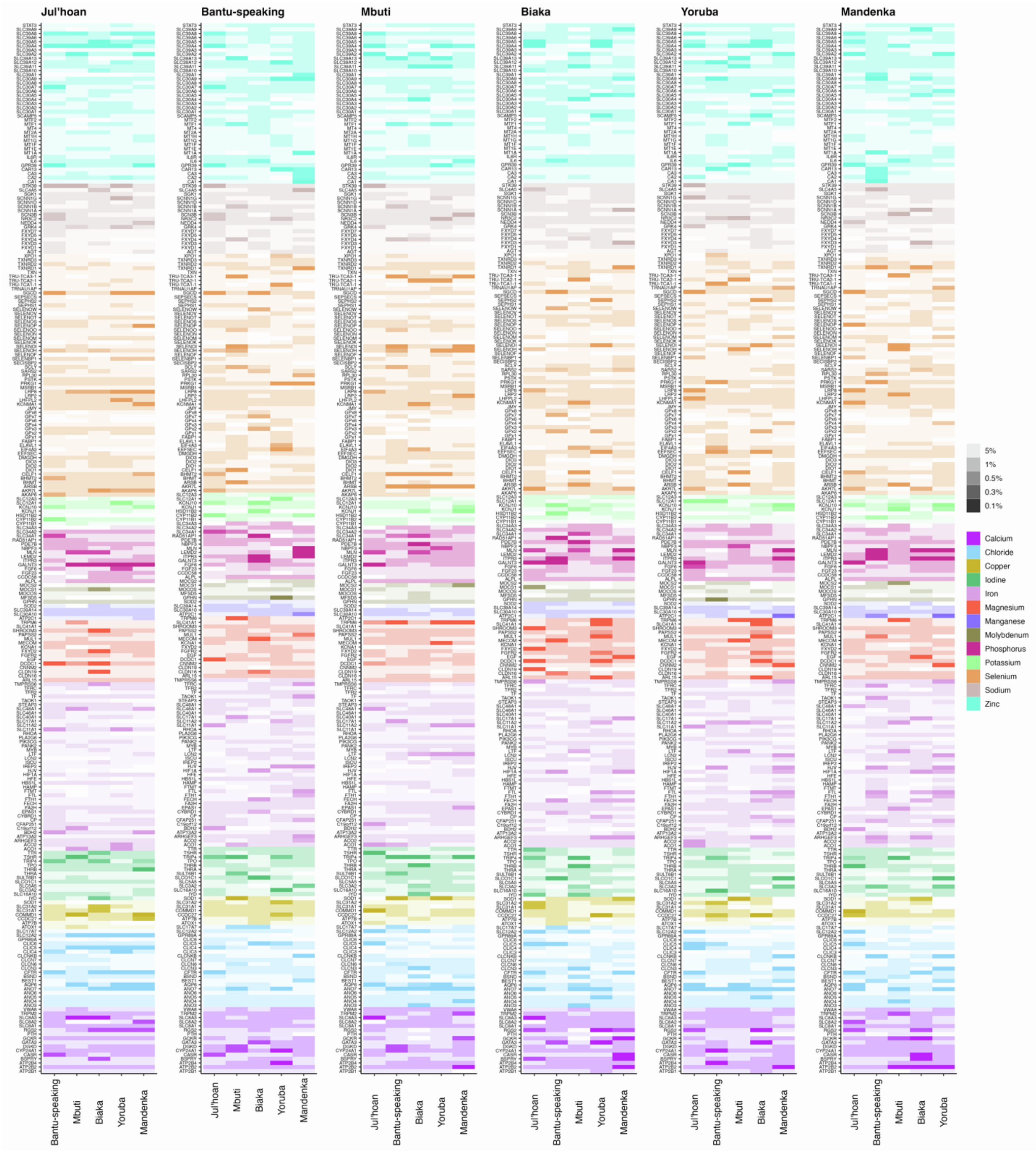

Figure S32: Signatures of positive selection as inferred by F_{ST} across all autosomal MA-genes for all within-Africa pairs. Signatures of positive selection as inferred by F_{ST} across all autosomal MA-genes (Y-axis, coloured by micronutrient) for all within-Africa pairs (each labelled panel represents one population vs. all other African populations on the X-axis). Darker blocks reflect lower empirical p-values (from lightest to darkest: below 5%, 1%, 0.5%, 0.3%, 0.1%, see left legend).

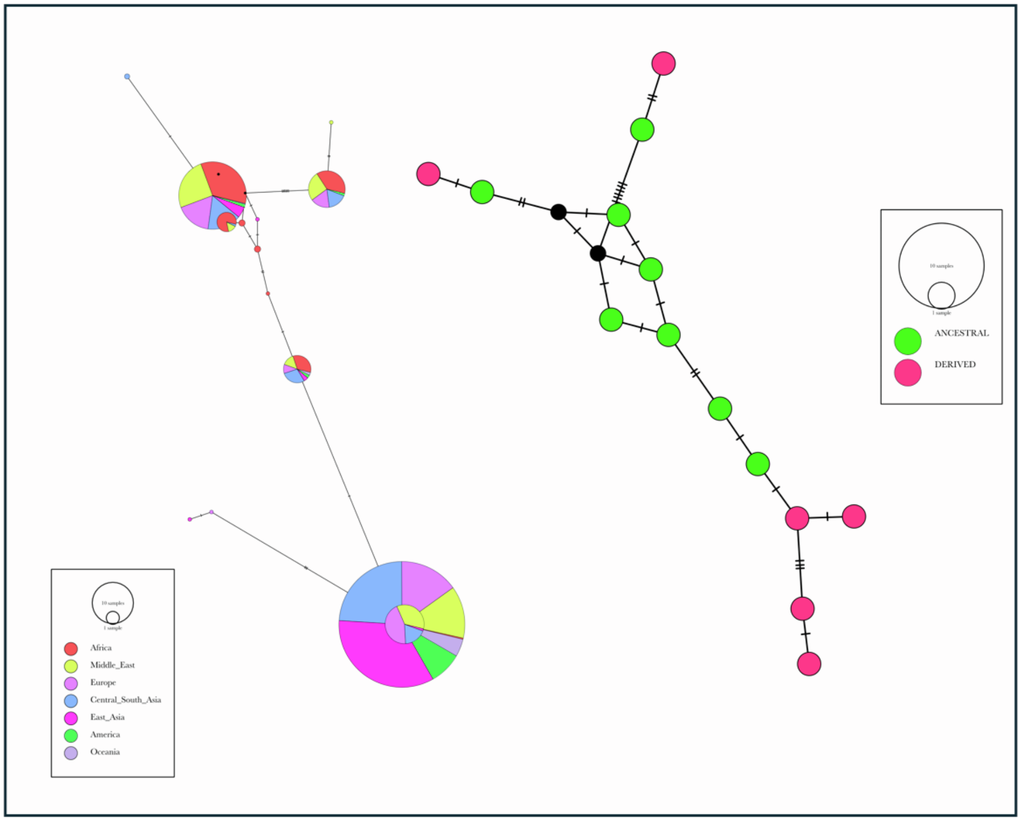

Figure S33: : Haplotype network for region surrounding chr4: 41981527. Haplotype network built from the 10kb region surrounding the chr4: 41981527 SNP (position given) of SLC30A9. Left: Red = Africa; Yellow = Middle-East; Purple = Europe; Blue = Central-South Asia; Pink = East Asia; Green= America; Lilac = Oceania. Right: Green= Ancestral, Pink = Derived.

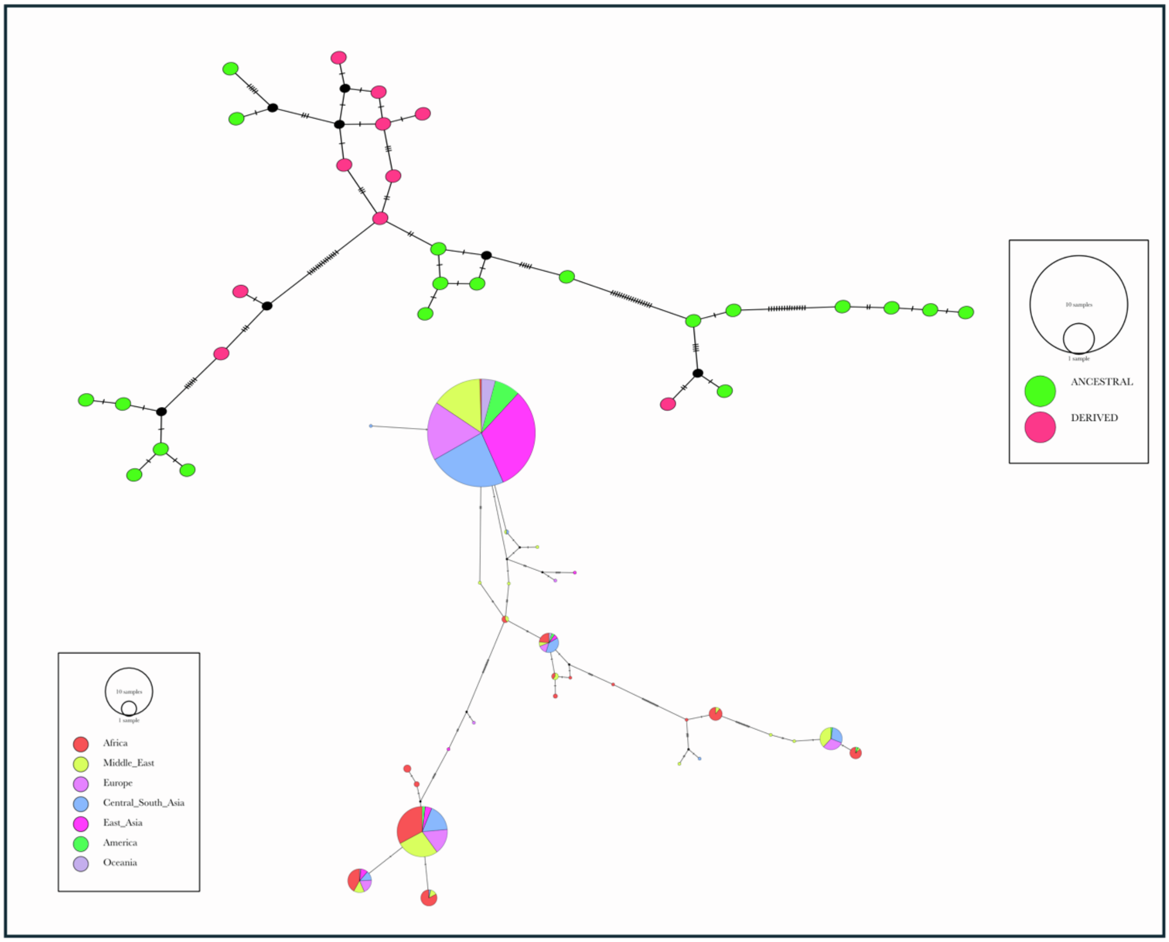

Figure S34: **Haplotype network for region surrounding chr4: 42001116.** Haplotype network built from the 10kb region surrounding the chr4: 42001116 SNP (position given) of SLC30A9. Top: Green= Ancestral, Pink = Derived. Bottom: Red = Africa; Yellow = Middle-East; Purple = Europe; Blue = Central-South Asia; Pink = East Asia; Green= America; Lilac = Oceania.

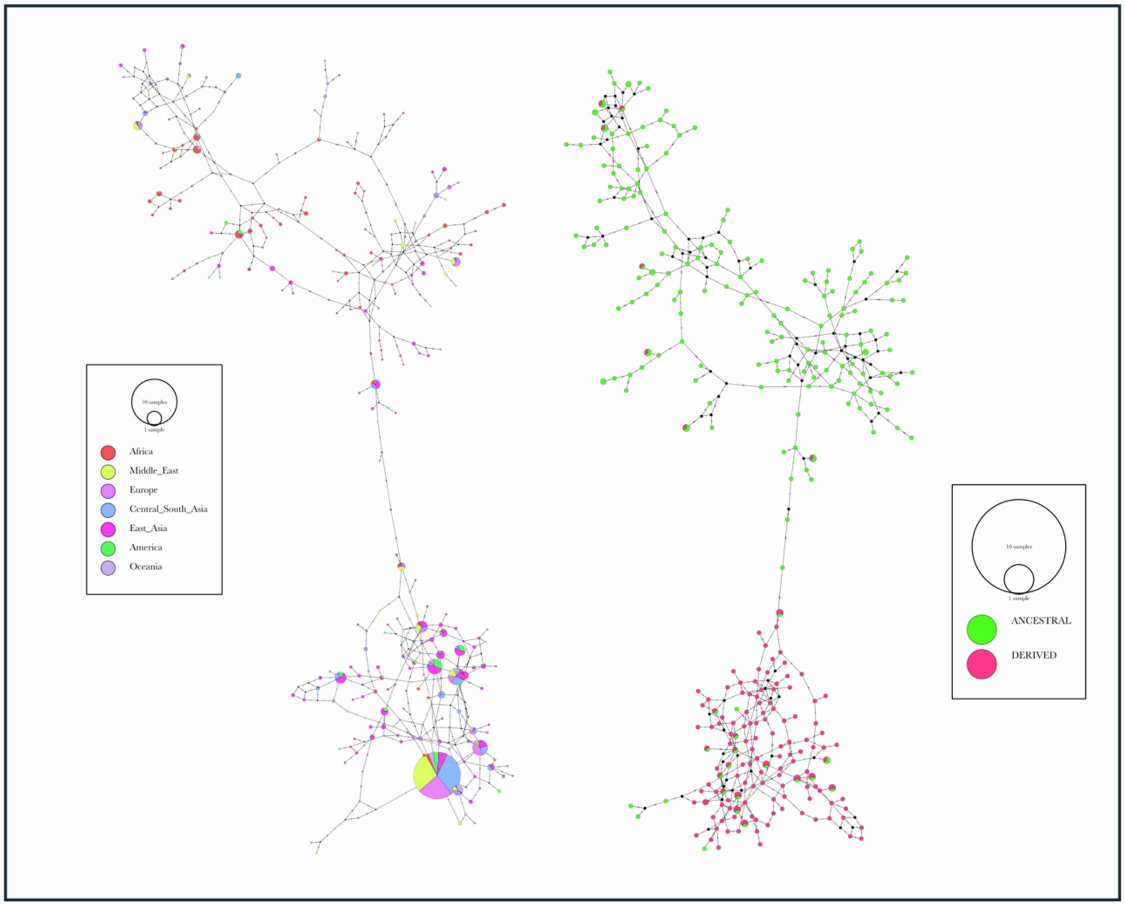

Figure S35: Haplotype network for region surrounding chr17: 73012306.
Haplotype network built from the 10kb region surrounding the chr17: 73012306 (position given) SNP of SLC39A11. Left: Red = Africa; Yellow = Middle-East; Purple = Europe; Blue = Central-South Asia; Pink = East Asia; Green= America; Lilac = Oceania. Right: Green= Ancestral, Pink = Derived.

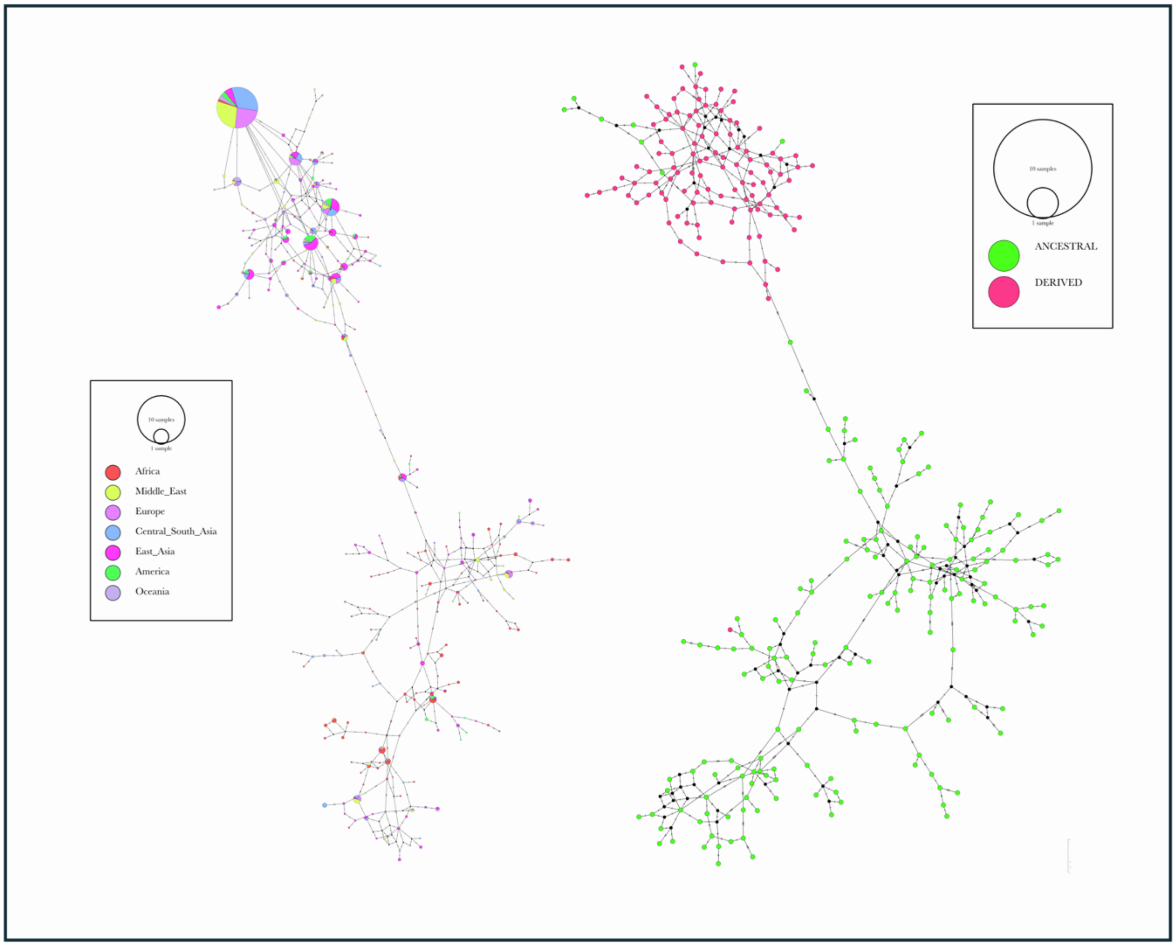

Figure S36: **Haplotype network for region surrounding chr17: 73010373.** Haplotype network built from the 10kb region surrounding the chr17: 73010373 SNP (position given) of SLC39A11. Left: Red = Africa; Yellow = Middle-East; Purple = Europe; Blue = Central-South Asia; Pink = East Asia; Green= America; Lilac = Oceania. Right: Green= Ancestral, Pink = Derived.

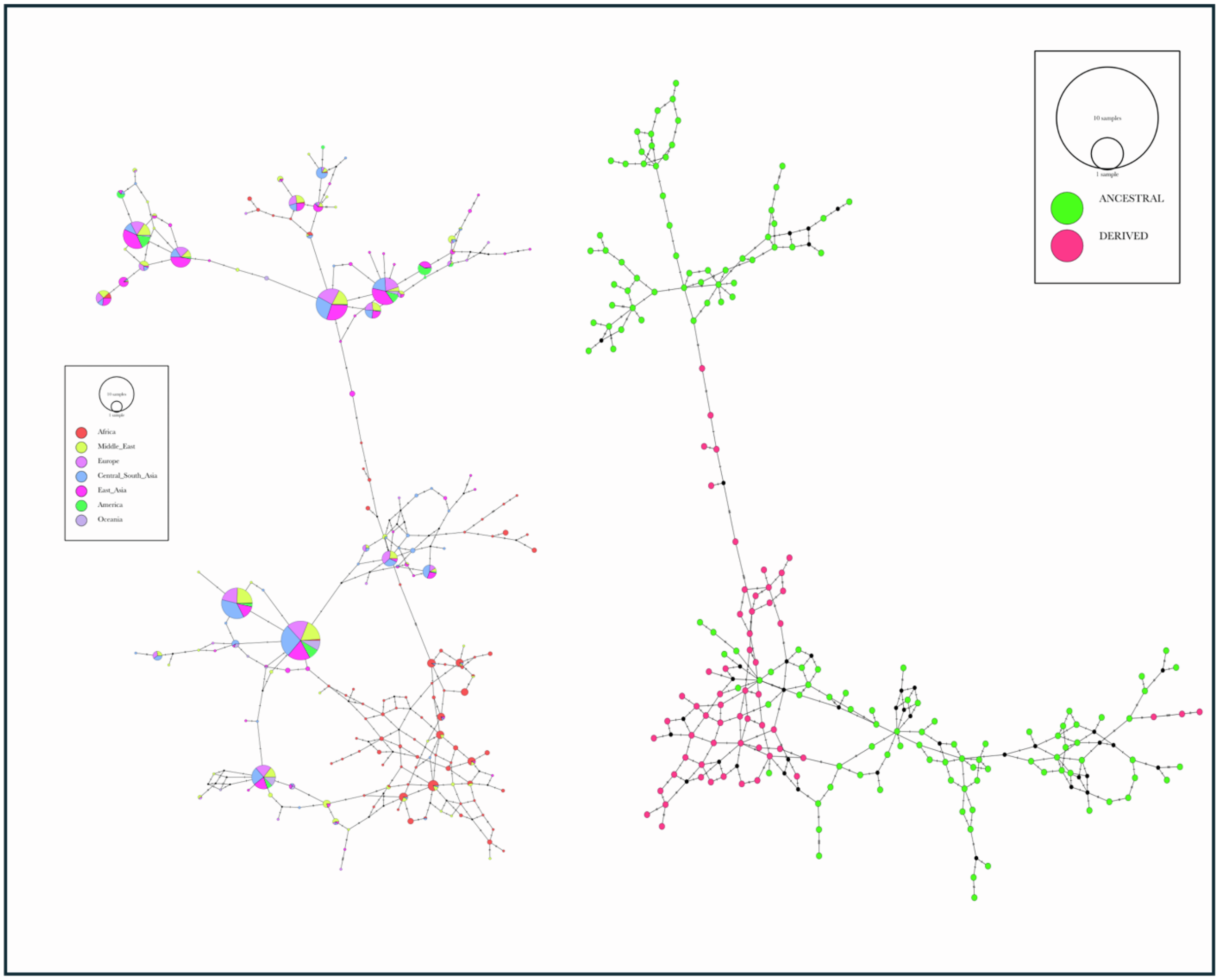

Figure S37: **Haplotype network for region surrounding chr2: 132638916.** Haplotype network built from the 10kb region surrounding the chr2: 132638916 (position given) SNP of GPR39. Left: Red = Africa; Yellow = Middle-East; Purple = Europe; Blue = Central-South Asia; Pink = East Asia; Green= America; Lilac = Oceania. Right: Green= Ancestral, Pink = Derived.

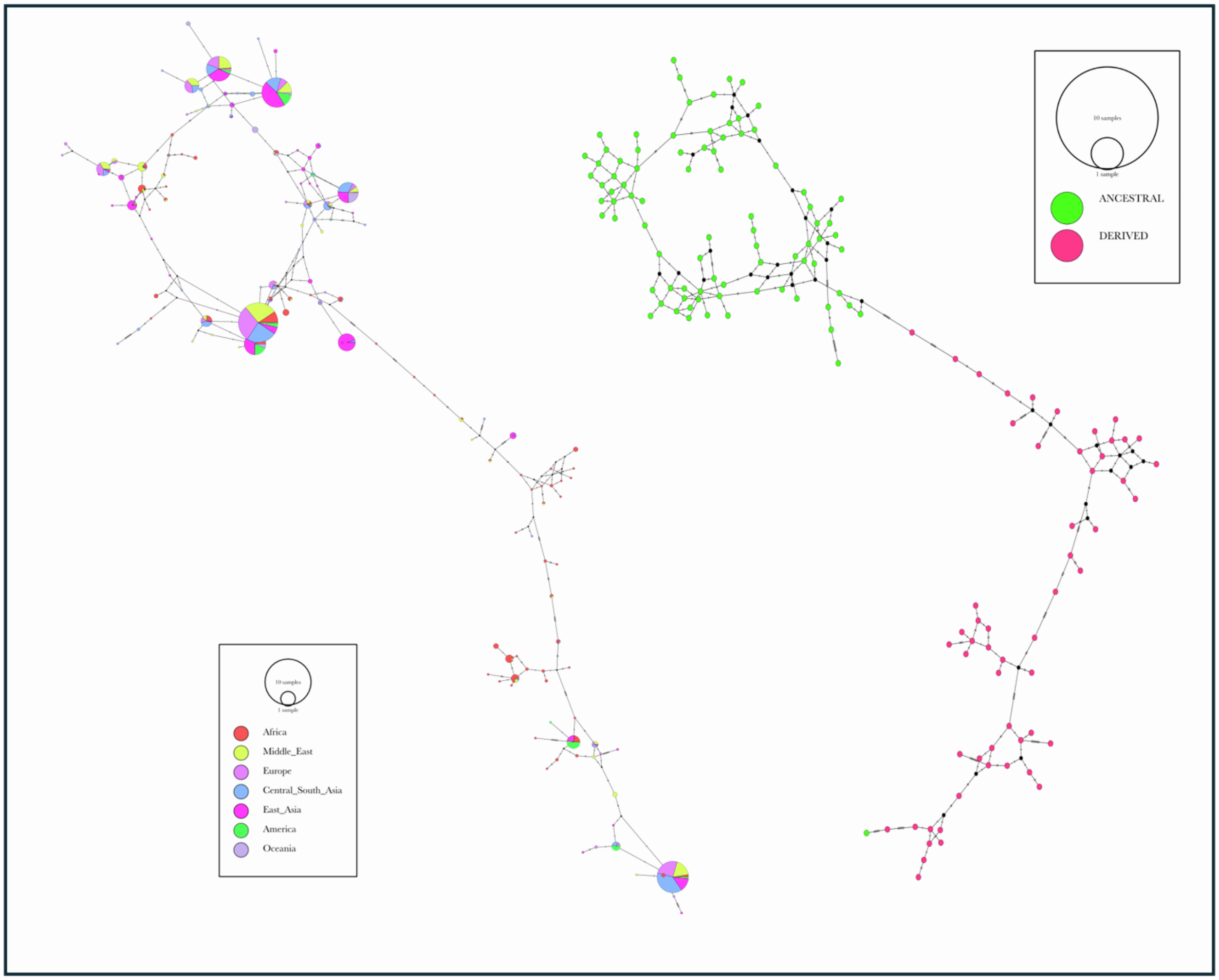

Figure S38: **Haplotype network for region surrounding chr2: 132602934**.Haplotype network built from the 10kb region surrounding the chr2: 132602934 (position given) SNP of GPR39. Left: Red = Africa; Yellow = Middle-East; Purple = Europe; Blue = Central-South Asia; Pink = East Asia; Green= America; Lilac = Oceania. Right: Green= Ancestral, Pink = Derived.

Tables

	Micronutrient	Number of Associated Genes<br>Total Set	Post-mask	Cut-down
**Trace Minerals**	Selenium	61	59	59
	Copper	11	11	9
	Iron	44	44	44
	Zinc	46	45	42
	Iodine	18	18	14
	Manganese	7	7	4
	Molybdenum	5	5	5
**Macrominerals**	Calcium	23	21	17
	Phosphorus	16	16	14
	Magnesium	19	19	15
	Sodium	20	20	17
	Chloride	25	23	22
	Potassium	11	11	7

Table S1: The total number of genes used in this study associated with the uptake, metabolism or regulation of 13 micronutrients. Number of genes for each MA-gene set given as a total (“Total Set”), the number of genes following the removal of any masked gene regions (“Post-mask”) and when cutting down gene sets to remove any overlap, assigning each gene to its most supported associated micronutrient set (“Cut-down”).

	Number of SNPs	Mean	Difference to Background Mean	Median	Difference to Background Median	Standard Deviation	Difference to Background Standard Deviation	Significance
**Selenium**	17614	0.337	-0.008	0.227	0.000	0.282	-0.008	0.057
**Copper**	**1409**	**0.315**	**-0.030**	**0.182**	**-0.045**	**0.279**	**-0.011**	**0.0008543**
**Iron**	6476	0.343	-0.002	0.227	0.000	0.292	0.002	0.6131
**Magnesium**	**7862**	**0.356**	**0.011**	**0.227**	**0.000**	**0.293**	**0.003**	**0.03418**
**Zinc**	7755	0.356	0.011	0.227	0.000	0.297	0.007	0.1861
**Sodium**	**5016**	**0.327**	**-0.018**	**0.205**	**-0.023**	**0.284**	**-0.006**	**0.001019**
**Calcium**	6978	0.344	-0.001	0.205	-0.023	0.293	0.003	0.6302
**Iodine**	4035	0.351	0.006	0.250	0.023	0.290	0.000	0.2436
**Chloride**	8514	0.337	-0.008	0.227	0.000	0.281	-0.009	0.9225
**Potassium**	1682	0.342	-0.003	0.250	0.023	0.276	-0.014	0.1838
**Phosphorus**	2662	0.344	-0.001	0.205	-0.023	0.294	0.004	0.8334
**Manganese**	2152	0.348	0.003	0.205	-0.023	0.289	-0.001	0.08396
**Molybdenum**	**1390**	**0.427**	**0.082**	**0.273**	**0.045**	**0.305**	**0.015**	**2.20E-16**

Table S2: Details on the allele frequency distribution of all micronutrient-associated gene sets. The number of SNPs over all genes in a given gene set (calculated from the Yoruba population), mean, median and standard deviation of the allele frequency distribution of all micronutrient-associated gene sets, the difference to that of the background (allele frequency distribution of chr1 of the Yoruba population; mean= 0.345, median= 0.227, standard deviation= 0.290), and the significance calculated when comparing these distributions (unpaired Wilcoxon test). Excluding the molybdenum gene set, the differences to the background mean are negligible.

Method	Micronutrient	Population	Significance	
$Relate$	Phosphorus	Pima	0.000013	**< 0.0001**
	**Sodium**	**Adygei**	**0.000029**	
	**Potassium**	**French**	**0.000322**	**< 0.001**
	Iodine	Maya	0.000325	
	**Sodium**	**Brahui**	**0.00115**	**<0.01**
	**Potassium**	**BergamoItalian_Tuscan**	**0.002963**	
	Sodium	Bougainville	0.003455	
	Potassium	Bougainville	0.003722	
	**Sodium**	**Russian**	**0.004935**	
	Sodium	Pathan	0.004951	
	Sodium	Ju|’hoan	0.0057	
	**Sodium**	**Orcadian**	**0.005823**	
	**Sodium**	**French**	**0.006133**	
	Iodine	Mozabite	0.006333	
	Calcium	Mozabite	0.007348	
	Iodine	Russian	0.009037	
	Sodium	NorthernHan_Tu	0.010827	**<0.05**
	**Sodium**	**BergamoItalian_Tuscan**	**0.01141**	
	**Sodium**	**Basque**	**0.011611**	
	Potassium	NorthernHan_Tu	0.014566	
	Sodium	Dai_Lahu	0.01592	
	**Potassium**	**Russian**	**0.017106**	
	Potassium	Druze	0.018052	
	Calcium	Sardinian	0.018387	
	Calcium	Pima	0.018616	
	**Sodium**	**Sindhi**	**0.021039**	
	Potassium	Xibo_Mongolian	0.021087	
	**Selenium**	**Xibo_Mongolian**	**0.02171**	
	Magnesium	Surui_Karitiana	0.023716	
	Potassium	Mandenka	0.02482	
	**Potassium**	**Sindhi**	**0.027062**	
	**Potassium**	**Palestinian**	**0.033025**	
	**Potassium**	**Mozabite**	**0.033461**	
	Manganese	Naxi_Yi	0.034193	
	Zinc	Naxi_Yi	0.03529	
	**Potassium**	**Sardinian**	**0.035508**	
	Phosphorus	Yoruba	0.036495	
	Iodine	Orcadian	0.036859	
	Copper	Sardinian	0.037657	
	Calcium	Japanese	0.038349	
	**Potassium**	**Kalash**	**0.038729**	
	Phosphorus	PapuanHighlands_PapuanSepik	0.04091	
	Calcium	Maya	0.042026	
	**Potassium**	**Pathan**	**0.044689**	
	Calcium	Pathan	0.044822	
	Potassium	Yoruba	0.045052	
	Phosphorus	Pathan	0.046086	
	Calcium	Orcadian	0.047252	
F_{ST}	Potassium	BantuSouthAfrica_BantuKenya	0.000043	**< 0.0001**
	Sodium	Makrani	0.00048	**< 0.001**
	Calcium	Mandenka	0.000912	
	Calcium	Biaka	0.001264	**< 0.01**
	Potassium	Orcadian	0.001556	
	Potassium	Surui_Karitiana	0.001698	
	**Potassium**	**Russian**	**0.002343**	

Zinc	Kalash	0.004891	
**Potassium**	**Palestinian**	**0.005466**	
Phosphorus	Mandenka	0.006715	
Sodium	Surui_Karitiana	0.0068	
**Potassium**	**Mozabite**	**0.008791**	
**Potassium**	**French**	**0.0088**	
**Potassium**	**Kalash**	**0.009572**	
**Selenium**	**Xibo_Mongolian**	**0.00993**	
Potassium	Pima	0.012051	**<0.05**
**Sodium**	**French**	**0.013281**	
Zinc	Uygur	0.016652	
**Sodium**	**Orcadian**	**0.016658**	
**Sodium**	**Russian**	**0.017819**	
Potassium	Basque	0.018501	
Potassium	Bedouin	0.01917	
**Sodium**	**BergamoItalian_Tuscan**	**0.020302**	
Potassium	Adygei	0.021671	
Iron	Mandenka	0.022027	
Potassium	Makrani	0.022717	
Potassium	Brahui	0.022756	
**Sodium**	**Sindhi**	**0.024379**	
**Sodium**	**Basque**	**0.024715**	
Selenium	Japanese	0.025141	
**Potassium**	**Sardinian**	**0.026595**	
**Sodium**	**Brahui**	**0.026743**	
Magnesium	Biaka	0.027088	
**Sodium**	**Adygei**	**0.028808**	
**Potassium**	**BergamoItalian_Tuscan**	**0.029975**	
Selenium	Pima	0.031199	
Selenium	Surui_Karitiana	0.032171	
**Potassium**	**Sindhi**	**0.032643**	
Selenium	Han	0.033762	
Selenium	She_Miao_Tujia	0.037054	
Selenium	Oroqen_Hezhen_Daur	0.038023	
Potassium	Balochi	0.038248	
Potassium	Dai_Lahu	0.042516	
Potassium	Burusho	0.042705	
Sodium	Kalash	0.043516	
**Potassium**	**Pathan**	**0.046447**	
Sodium	Biaka	0.047979	
Potassium	Ju|'hoan	0.048742	
Potassium	Maya	0.048774	

Table S3: Micronutrient-associated gene sets with significantly different summed selection values. According to the gene set method $\mathrm{SUMSTAT}$ integrating Relate or $\mathrm{F_{ST}}$ selection values. Bold rows are for MA-gene set and population pairs with significantly different summed selection results according to both integrating Relate and $\mathrm{F_{ST}}$ selection values. Partitioned by significance.

Method	Micronutrient	Population	Significance	
Relate	Phosphorus	Pima	0.005012	**< 0.01**
	**Selenium**	**Xibo_Mongolian**	**0.02171**	**< 0.05**
F_{ST}	**Selenium**	**Xibo_Mongolian**	**0.00993**	**< 0.01**
	Iron	Mandenka	0.022027	
	Selenium	Japanese	0.025141	
	Selenium	Pima	0.031199	
	Selenium	Surui_Karitiana	0.032171	
	Selenium	Han	0.033762	
	Selenium	She_Miao_Tujia	0.037054	
	Selenium	Oroqen_Hezhen_Daur	0.038023	**< 0.05**

Table S4: Micronutrient-associated gene sets, as cut down to remove overlap, with significantly different summed selection values. According to the gene set method $\mathrm{SUMSTAT}$ integrating Relate or $\mathrm{F_{ST}}$ selection values. Bold rows are for MA-gene set and population pairs with significantly different summed selection results according to both integrating Relate and $\mathrm{F_{ST}}$ selection values. Partitioned by significance.

Micronutrient	Gene	Candidate SNP Position	rsID	Ancestral/Derived	Consequence	CADD Score	No. Populations		Strongest Evidence	
							Relate	Fst	Relate	Fst
Calcium	*ATP2B2*	chr3:10456514	rs638107	G/C	Intron variant	4.955	3	9	4.97e-5 (Makrani)	0.0003 (Basque)
		chr3:10604833	rs11720808	C/A	Intron variant	0.053	3	1	2.10e-7 (Sardinian)	0.00052 (Mozabite)
	ATP2B4	chr1:203648263	rs7540131	T/A	Intron variant	15.17	4	0	0.00054 (Bedouin)	0.0049 (Biaka)
		chr1:203667951	rs12045866	A/G	Intron variant	5.235	3	14	0.00057 (Bedouin)	0.00019 (Russian)
	SLC8A1	chr2:40394610	rs2192773	A/T	Intron variant	0.567	3	0	0.000452 (Yakut)	0.0014 (Uygur)
		chr2:40584510	rs10172760	T/C	Intron variant	3.341	4	0	2.40e-5 (Makrani)	0.013 (Makrani)
	SLC8A2	chr19:47428756	rs2280692	T/C	3 prime UTR variant	4.661	0	5	0.0087 (French)	0.00013 (Sardinian)
		chr19:47437107	rs4802363	C/T	Intron variant	0.477	0	7	0.0033 (Brahui)	0.00033 (Sardinian)
	SLC8A3	chr14:70182346	rs66672884	T/G	Intron variant	3.026	0	2	0.0052 (NorthernHan-Tu)	0.00072 (Uygur)
		chr14:70175561	rs715305	A/G	Intron variant	0.878	2	0	3.90e-5 (NorthernHan-Tu)	
Iron	*ARHGEF3*	chr3:56761998	rs9825091	C/T	Intron variant	9.002	0	14		2.89e-5 (Balochi)
		chr3:57043874	rs55952750	G/A	Intron variant	3.634	1	0	0.00029 (Maya)	0.0043 (Pima)
	HIF1A	chr14:61687412	rs4899055	G/C	Intron variant	0.192	0	5	0.0033 (Balochi)	0.00024 (Basque)
		chr14:61709502	rs12232182	C/T	Intron variant	13.75	4		2.28e-5 (Sindhi)	0.0018 (Surui-Karitiana)
		chr14:61741756	rs4902081	C/G	Intron variant	6.7	1	3	2.43e-6 (Basque)	0.00031 (Pima)
	FTMT	chr5:121846819	rs6595327	A/C	Intron variant	0.277	2	11	3.37e-6 (Yakut)	4.85e-5 (Dai-Lahu)
		chr5:121853801	rs1560550	A/G	Intron variant	4.003	3	1	0.00022 (She-Miao-Tujia)	0.000203 (Dai-Lahu)
	SLC40A1	chr2:189577426	rs10188785	T/G	Intron variant	0.855	3	4	1.62e-5 (Uygur)	0.00031 (Pima)
		chr2:189591670	rs2352270	G/A	Intergenic variant	0.784	0	1	0.0012 (Naxi-Yi)	0.0001 (Pima)

Table S5: The candidate SNPs in CALCIUM- and IRON-associated genes chosen for analysis in $\mathbf{CLUES2}$. The candidate SNPs (positions given) in CALCIUM- and IRON-associated genes chosen for analysis in $\mathrm{CLUES2}$ (see **Note S1**) alongside their inferred genomic consequences (as inferred from dbSNP[52]) and CADD score[53]. “No. Populations” gives the number of populations where this SNP falls into the 0.1% empirical tail of Relate or $\mathrm{F_{ST}}$. “Strongest Evidence” gives the most extreme pvalue over our dataset and the corresponding population.

Population	Top-ranking MA-Gene	Micronutrient	Significance
Ju|'hoan	*PRKG1*	selenium	0.0011774
	AKAP6	selenium	0.0011774
	SGCD	selenium	0.0011774
	SELENOP	selenium	0.0011774
	TXNRD3	selenium	0.0011774
	PSTK	selenium	0.0011774
Mbuti	*SGK1*	selenium	6.77e-5
	SGCD	selenium	8.72e-5
	EEFSEC	selenium	0.00020261
Biaka	*SKG1*	selenium	4.81e-5
	SGCD	selenium	8.77e-5
Mandenka	*TRPM2*	calcium	2.11e-5
	SLC8A1	calcium	7.52e-5
Druze	*FGFR2*	magnesium	6.67E-05
	SHROOM3	magnesium	0.00021632
Bedouin	*EPAS1*	iron	0.00013935
	HBS1L	iron	0.0002138
Adygei	*C19orf12*	iron	2.01E-05
	CFAP251	iron	9.28E-05
	ARHGEF3	iron	0.00015855
Sardinian	*ATP2B2*	calcium	2.10E-07
	SLC8A1	calcium	0.00012589
	SECISBP2	selenium, iodine	3.27E-05
	VWA8	selenium	0.0002822
Basque	*HIF1A*	iron	2.43E-06
	ARHGEF3	iron	2.47E-05
	TXNRD3	selenium	4.79E-05
	EEFSEC	selenium	5.59E-05
French	*SCNN1D*	sodium, potassium	1.87E-06
	ANO3	chloride	4.56E-05
	SLC12A1	sodium, chloride, potassium	0.00022405
Russian	*SLC4A5*	sodium	3.83E-06
	SCNN1D	sodium, potassium	6.81E-06
	SLC30A1	zinc	1.32E-05
	KCNMA1	calcium, potassium	0.000137
	SLC39A11	zinc	0.00015104
Makrani	*SLC8A1*	calcium	2.40E-05
	ATP2B2	calcium	4.97E-05
	SLC39A11	zinc	1.40E-06
	SLC39A12	zinc	2.40E-05
	ATP2B2	calcium	4.97E-05
Sindhi	*HIF1A*	iron	2.28E-05
	HSD11B2	iron	9.68E-05
	SLC39A11	zinc	0.00015917
	SLC30A9	zinc	0.00031178
Brahui	*GPx2*	selenium	0.00010148
	SGK1	selenium	0.00010148
Hazara	*PRKG1*	selenium	5.56E-05
	ARSB	selenium	0.00017433
	SELENOP	selenium	0.00017433
	TXNRD1	selenium	0.00017433
	LRP2	selenium	0.00034417
Pathan	*ANO3*	chloride	1.76E-05
	SLC12A1	sodium, chloride, potassium	5.81E-05
Kalash	*SLC39A10*	zinc, magnesium, manganese	1.21E-05
	SLC12A3	calcium, magnesium, potassium	2.21E-05
	MECOM	magnesium	0.00014809
Uygur	*ATP2B2*	calcium	5.23E-05
	SLC8A1	calcium	0.0002744
	SLC40A1	iron	1.62E-05
	PLA2G6	iron	7.10E-05
Xibo-Mongolian	*SLC8A3*	calcium	6.74E-05
	KCNMA1	calcium, potassium	0.00016803
	PRKG1	selenium	0.0002743

	SELENOP	selenium	0.0002743
Oroqen-Hezhen-Daur	*SLC40A1*	iron	0.00023563
	ARHGEF3	iron	0.00023563
	PRKG1	selenium	0.00023563
	ARSB	selenium	0.00023563
	AKAP6	selenium	0.00023563
Yakut	*AKAP6*	selenium	7.62E-05
	GPx7	selenium	0.00013104
Japenese	*LHFPL2*	selenium	0.00015814
	ATP2B2	selenium	0.0002387
Han	*TRIP4*	iodine	0.00013754
	SLCO1C1	iodine	0.00013754
NorthernHan-Tu	*SLC8A3*	calcium	3.90E-05
	KCNMA1	calcium, potassium	3.90E-05
She-Miao-Tujia	*MLN*	phosphorus	4.27E-06
	ITPR3	phosphorus	1.93E-05
Naxi-Yi	*PRKG1*	selenium	2.17E-05
	SELENOI	selenium	0.00037186
	SLC39A11	zinc	8.43E-05
	SLC39A8	zinc, magnesium, manganese	0.00016511
	IL6	zinc	0.00037186
	SLC30A10	zinc, magnesium, manganese	0.00037186
Dai-Lahu	*SLC8A3*	calcium	4.43E-05
	SLC8A1	calcium	7.39E-05
Pima	*ITPR3*	phosphorus	8.09E-05
	MLN	phosphorus	0.00053942
	LEMD2	phosphorus	0.00053942
Maya	*SLC8A1*	calcium	4.17E-05
	ATP2B1	calcium	0.00015384
	TRPM6	magnesium	5.25E-05
	ARL15	magnesium	0.00015794
	TSHR	iodine	0.00027492
	THRA	iodine	0.00027492
Surui-Karitiana	*CLDN16*	magnesium	0.00012466
	SLC39A8	zinc, magnesium, manganese	0.00020085
	MECOM	magnesium	0.00041299
	SLC39A10	zinc	0.00014355
	SLC39A11	zinc	0.00041299
Papuan	*SLC8A1*	calcium	1.26E-05
	ATP2B2	calcium	1.97E-05
	HIF1A	iron	0.00027674
	HBS1L	iron	0.00027674
Bougainville	*SLC39A11*	zinc	0.000375
	SLC30A6	zinc	0.000375

Table S6: Populations with top-ranking MA-genes that fall within the same MA-gene set according to Relate. "Population" = populations where two or more of the top-ranking MA-genes ("Top-ranking MA-gene"; those with signatures of positive selection ranking within the top-five strongest signatures of positive selection according to Relate for that population) fall within the same micronutrient gene set ("Micronutrient"). Significance according to Relate given in "Significance" column.

Population	Gene	Micronutrient	Significance
Ju|'hoan	*LRP8*	selenium	2.15E-05
	LHFPL2	selenium	3.08E-05
Bantu-speaking	*SLC12A1*	sodium, chloride, potassium	5.06E-06
	KCNJ10	calcium, potassium	1.38E-05
	LHFPL2	selenium	4.99E-06
	PRKG1	selenium	2.11E-05
	EEFSEC	selenium	2.53E-05
Mbuti	*TRU-TCA2-1*	selenium	6.63E-05
	LHFPL2	selenium	7.44E-05
Biaka	*SLC8A1*	calcium	3.05E-05
	ATP2B4	calcium	8.39E-05
	LHFPL2	selenium	8.33E-05
	EEFSEC	selenium	0.00010422
Mandenka	*ATP2B2*	calcium	7.75E-08
	SLC8A1	calcium	3.00E-05
	FTL	iron	1.99E-05
	HJV	iron	2.91E-05
Mozabite	*EEFSEC*	calcium	1.29E-05
	ATP2B4	calcium	8.32E-05
Palestinian	*SLC39A4*	zinc	2.85E-05
	GPR39	zinc	3.72E-05
Druze	*SLC39A4*	zinc	1.02E-05
	GPR39	zinc	3.23E-05
BergamoItalian-Tuscan	*SLC39A4*	zinc	8.84E-06
	GPR39	zinc	8.94E-05
Sardinian	*SLC39A4*	zinc	1.33E-05
	GPR39	zinc	5.57E-05
Basque	*SLC39A4*	zinc	1.41E-05
	GPR39	zinc	0.00013401
French	*SLC12A1*	sodium, chloride, potassium	7.65E-07
	AQP6	chloride	3.41E-05
	SLC39A4	zinc	5.58E-06
	GPR39	zinc	5.35E-05
Orcadian	*SLC12A1*	sodium, chloride, potassium	4.80E-06
	SCNN1A	sodium, potassium	9.97E-05
	SLC39A4	zinc	3.19E-05
	SLC30A10	zinc, magnesium, manganese	9.97E-05
Russian	*SLC12A1*	sodium, chloride, potassium	1.40E-06
	SLC4A5	sodium	1.89E-05
Makrani	*SGCD*	selenium	1.18E-05
	SGK1	selenium	3.59E-05
Sindhi	*SLC39A4*	zinc	1.03E-05
	SLC39A11	zinc	2.99E-05
	GPR39	zinc	5.83E-05
Brahui	*SLC39A4*	zinc	7.98E-06
	GPR39	zinc	2.63E-05
Hazara	*SLC39A4*	zinc	7.24E-06
	SLC30A9	zinc	2.23E-05
	GPR39	zinc	9.24E-05
	MTF2	zinc	9.77E-05
Pathan	*SLC39A4*	zinc	5.47E-06
	GPR39	zinc	3.47E-05
	SLC30A9	zinc	6.08E-05
Burusho	*SLC39A4*	zinc	8.76E-06
	SLC30A9	zinc	5.38E-05
	GPR39	zinc	5.78E-05
Kalash	*SLC39A4*	zinc	1.72E-05
	SLC39A11	zinc	9.98E-05
	GPR39	zinc	0.00013799
Uygur	*SLC39A4*	zinc	5.66E-05
	CA3	zinc	0.00019682
Xibo-Mongolian	*PRKG1*	selenium	1.00E-05
	SEPHS2	selenium	2.66E-05
	SLC30A9	zinc	2.66E-05
	SLC39A4	zinc	5.49E-05
	HSD11B2	iron	6.97E-05
Oroqen-Hezhen-Daur	*SLC30A9*	zinc	1.51E-05

	SLC39A4	zinc	3.99E-05
Yakut	*SLC30A9*	zinc	1.71E-05
	SLC39A4	zinc	3.57E-05
	PRKG1	selenium	0.00010185
	DIO1	selenium, iodine	0.0002481
Japanese	*SLC39A4*	zinc	6.69E-05
	SLC30A9	zinc	0.00014582
Han	*SLC30A9*	zinc	3.55E-06
	SLC39A4	zinc	4.34E-05
NorthernHan-Tu	*SLC39A4*	zinc	5.98E-05
	SLC30A9	zinc	7.01E-05
	PRKG1	selenium	0.00011442
	SEPHS2	selenium	0.00020983
She-Miao-Tujia	*SLC30A9*	zinc	2.05E-05
	SLC39A4	zinc	4.75E-05
Naxi-Yi	*SLC39A4*	zinc	9.03E-05
	SLC30A9	zinc	0.00010709
	SEPHS2	selenium	0.00010969
	PRKG1	selenium	0.0001864
Dai-Lahu	*SLC30A9*	zinc	2.12E-05
	SLC39A4	zinc	7.82E-05
Pima	*GPx3*	selenium	0.00010471
	SELENON	selenium	0.00010471
	RHOA	iron	0.00010471
	SLC40A1	iron	0.00010471
	STAT3	zinc	0.00010471
	SLC39A11	zinc	0.00010471
	SLC30A2	zinc	0.00010471
Surui-Karitiana	*GPx3*	selenium	0.00012002
	SGCD	selenium	0.00012002
Papuan	*ACO1*	chloride	5.36E-05
	CLCN3	chloride	0.00025195
	SLC39A11	zinc	9.64E-05
	SLC30A9	zinc	0.00025195
Bougainville	*TFRC*	iron	3.80E-05
	TMPRSS6	iron	8.23E-05
	DCDC1	magnesium	3.80E-05
	SLC41A1	magnesium	8.23E-05

Table S7: Populations with top-ranking MA-genes that fall within the same MA-gene set according to $\mathbf{F_{ST}}$. "Population" = populations where two or more of the top-ranking MA-genes ("Top-ranking MA-gene"; those with signatures of positive selection ranking within the top-five strongest signatures of positive selection according to F_{ST} for that population) fall within the same micronutrient gene set ("Micronutrient"). Significance according to F_{ST} given in "Significance" column.

Gene	Focal SNP	No. Populations $Relate$	F_{ST}
SLC30A9	chr4:41981527	3	25*
	chr4:42001116	8*	21
SLC39A11	chr17:73012306	11	20*
	chr17:73010373	15*	6
GPR39	chr2:132638916	1	30*
	chr2:132598713	3*	2

Table S8: **Candidate SNPs of ZINC-associated genes with geographically widespread signatures of positive selection**. Candidate SNPs of ZINC-associated genes with geographically widespread signatures of positive selection (those identified as candidate SNPs in ten or more populations) that are identified as candidate SNPs in the highest number of populations according to either Relate and $\mathrm{F_{ST}}$ ["No. Populations" gives the number of populations where this SNP is identified as a candidate SNP for either Relate and $\mathrm{F_{ST}}$]. Asteriks indicate that this is the most commonly identified candidate SNP in this gene according to the column's respective method.

Gene	0.1% tail	
	No.	Populations
PRKG1	24	Bantu-speaking, Biaka, Yoruba, Mandenka, Palestinian, Bedouin, Adygei, Sardinian, Basque, Sindhi, Balochi, Hazara, Pathan, Burusho, Kalash, Xibo-Mongolian, Oroqen-Hezhen-Daur, Yakut, Japanese, Han, NorthernHan-Tu, She-Miao-Tujia, Naxi-Yi, Dai-Lahu
SGCD	21	Bantu-speaking, Mbuti, Biaka, Mozabite, Palestinian, Druze, Bedouin, BergamoItalian-Tuscan, Sardinian, Basque, Sindhi, Hazara, Burusho, Oroqen-Hezhen-Daur, Yakut, Han, NorthernHan-Tu, She-Miao-Tujia, Naxi-Yi, Dai-Lahu, Bougainville
AKAP6	20	Bantu-speaking, Biaka, Yoruba, Mandenka, Mozabite, Palestinian, Druze, Bedouin, Adygei, BergamoItalian-Tuscan, Sardinian, Basque, Burusho, Xibo-Mongolian, Oroqen-Hezhen-Daur, Yakut, NorthernHan-Tu, Naxi-Yi, Surui-Karitiana, Papuan
KCNMA1	16	Mbuti, Biaka, Yoruba, Mandenka, Adygei, BergamoItalian-Tuscan, Russian, Sindhi, Xibo-Mongolian, Yakut, Japanese, Han, NorthernHan-Tu, She-Miao-Tujia, Naxi-Yi, Dai-Lahu
EEFSEC	5	Bantu-speaking, Mbuti, Adygei, Basque, Balochi
ARSB	5	Hazara, Burusho, Xibo-Mongolian, Oroqen-Hezhen-Daur, Japanese
SELENOP	5	Bantu-speaking, Mandenka, Hazara, Pathan, Xibo-Mongolian
LRP8	5	Bantu-speaking, Yoruba, Mandenka, Balochi, Xibo-Mongolian
SELENOS	5	Biaka, Yoruba, Palestinian, Balochi, NorthernHan-Tu
LHFPL2	4	Biaka, Mandenka, Japanese, Maya
SCLY	4	Bantu-speaking, Mozabite, Russian
GPx2	4	BergamoItalian-Tuscan, Makrani, Balochi, Brahui
TXNRD3	3	Mozabite, Adygei, Basque
SECISBP2	3	Mandenka, Sardinian, Russian
AKR7L	1	Burusho

TXNRD2	1	Dai-Lahu
SELENOM	1	Yoruba

Table S9: **The populations with signatures of positive selection for all selenium-associated genes.** The number ("No.") and name of the populations ("Populations") with signatures of positive selection, as identified by the 0.1% tail of the empirical background distribution of Relate, for all selenium-associated genes with p-values $< 10^{-5}$ in at least one population.

Gene	Population	$Relate$ Significance	F_{ST} Significance
PRKG1	Bantu-speaking		2.11e-5
	Palestinian	6.83e-5	
	Hazara	5.56e-5	
	Han	6.54e-5	
	Naxi-Yi	2.17e-5	
	She-Miao-Tujia		2.05e-5
	Xibo-Mongolian		1e-5
SGCD	Biaka	8.77e-5	
	Mbuti	8.72e-5	
	Palestinian	9.50e-5	
	BergamoItalian-Tuscan		1.07e-5
	Makrani		1.18e-5
	Papuan		5.36e-5
AKAP6	Mozabite	4.16e-5	
	Adygei	4.62e-5	
	Yakut	7.62e-5	
	Surui-Karitiana	3.96e-5	
EEFSEC	Bantu-speaking		2.53e-5
	Bedouin		8.33e-5
	Mozabite		1.29e-5
	Basque	5.59e-5	
LHFPL2	Bantu-speaking		4.99e-6
	Biaka		8.33e-5
	Mbuti		7.44e-5
	Ju|’hoan		3.08e-5
LRP8	Bantu-speaking	8.8e-5	
	Mandenka	1.04e-5	
	Ju|’hoan	2.15e-5	
SELENOS	Russian		4.01e-5
	Brahui		8.04e-5
	Hazara		1.86e-5
KCNMA1	NorthernHan-Tu	3.9e-5	
	Yakut	1.46e-5	
SLCY	Mozabite	9.09e-5	
TXNDR3	Basque	4.79e-5	
SECISBP2	Sardinian	3.27e-5	
AKR7L	Burusho	9.83e-6	
GPx2	Makrani	9.61e-6	
TXNRD2	Mandenka		9.72e-5
TRU-TCA2-1	Mbuti		6.63e-5
ARSB	Orcadian		7.92e-5
SELENOM	Yoruba	5.87e-6	
SELENOP	Pathan	4.09e-5	
SELENOW	Japanese		6.91e-5
SEPHS2	Xibo-Mongolian		2.66e-5

Table S10: **Selenium-associated genes with p-values $< 10^{-5}$.** Selenium-associated genes with p-values $< 10^{-5}$, as calculated from the empirical distribution of either Relate or F_{ST}. P-values less than 4.65×10^{-6} (see **Section 3.4.5**) highlighted in bold.

Overlapping Genes				Overlap (bp)	Nature of Overlap
GPx1	(selenium)	RHOA	(iron)	787	$\pm 10kbp$
LHFPL2	(selenium)	ARSB	(selenium)	7188	$\pm 10kbp$
LEMD2	(phosphorus)	MLN	(phosphorus)	5543	$\pm 10kbp$
MT1F	(zinc)	MT1G	(zinc)	6032	$\pm 10kbp$
MT1G	(zinc)	MT1H	(zinc)	1749	$\pm 10kbp$
FXYD1	(sodium)	FXYD7	(sodium)	141	$\pm 10kbp$
FXYD7	(sodium)	FXYD5	(sodium)	428	$\pm 10kbp$
DMGDH	(selenium)	BHMT2	(selenium)	166271	$ensemble$
GPx5	(selenium)	GPx6	(selenium)	2334	$ensemble$
CA1	(zinc)	CA3	(zinc)	5578	$ensemble$
BEST1	(chloride)	FT1H	(zinc)	5797	$ensemble$

Table S11: **All overlapping micronutrient-associated genes**. All micronutrient-associated genes which are less than 10kbp (“Nature of Overlap” = “$\pm 10\mathrm{kbp}$”) or have overlapping gene regions as given by $\mathrm{ensemble}$ (“Nature of Overlap” = “$\mathrm{ensemble}$”)

Micronutrient	Gene	SNPs	CDF
Selenium	*SELENOO*	1083	0.99776
Iron	*EPAS1*	2829	0.97643
Zinc	*MT1A*	584	0.97112
Zinc	*MT1F*	631	0.98755
Sodium, Potassium	*SCNN1D*	807	0.95443
Calcium	*SLC8A1*	13155	0.98523
Chloride	*CLCN7*	1388	0.99038

Table S12: **Micronutrient-associated genes enriched for SNP-density** . Micronutrient genes enriched for SNP-density (over 95% quantile of the cumulative density function drawn from the distribution formed from generated neutral gene regions).

Metapopulation	Group name	Populations	Sample Size
**Africa**	Mbuti	Mbuti	13
	Biaka	Biaka	22
	Ju|'hoan	Ju|'hoan	6
	Bantu-speaking	Bantu(Kenya), Bantu(SouthAfrica)	19
	Yoruba	Yoruba	22
	Mandenka	Mandenka	22
**Middle-East**	Mozabite	Mozabite	27
	Palestinian	Palestinian	46
	Druze	Druze	42
	Bedouin	Bedouin	46
**Europe**	BergamoItalian-Tuscan	Bergamo_Italian, Tuscan	21
	Russian	Russian	25
	Adygei	Adygei	16
	Orcadian	Orcadian	15
	French	French	28
	Basque	Basque	23
	Sardinian	Sardinian	28
	Russian	Russian	25
**East-Asia**	Xibo-Mongolian	Mongolian, Xibo	18
	NorthernHan-Tu	NorthernHan, Tu	20
	Naxi-Yi	Naxi, Yi	18
	She-Miao-Tujia	She, Miao, Tujia	29
	Oroqen-Hezhen-Daur	Oroqen, Hezhen, Daur	27
	Dai-Lahu	Dai, Lahu	17
	Han	Han	33
	Japanese	Japanese	27
	Yakut	Yakut	25
**Central-South Asia**	Hazara	Hazara	19
	Uygur	Uygur	10
	Makrani	Makrani	25
	Sindhi	Sindhi	24
	Balochi	Balochi	24
	Brahui	Brahui	25
	Burusho	Barusho	25
	Kalash	Kalash	22
	Pathan	Pathan	24

**Oceania**	Papuan	Papuan (Sepik), Papuan(Highlands)	17
	Bougainville	Bougainville	11
**Americas**	Pima	Pima	13
	Maya	Maya	21
	Surui-Karitiana	Surui, Karitiana	20

Table S13: **Populations used in this study.** Populations used in this study, as defined by[2] .Note, Ju|’hoan refers to the population previously labelled San by the Human Diversity Genome Panel (HGDP).

Population	Correlation	Rsquared	Pvalue
**Ju|’hoan**	0.07659437	0.0058667	2.55E-40
**BantuSouthAfrica_BantuKenya**	0.03474197	0.001207	1.79E-16
**Mbuti**	0.14264497	0.02034759	1.30E-213
**Biaka**	0.07205673	0.00519217	6.80E-75
**Mandenka**	0.01919085	0.00036829	3.08E-06
**Mozabite**	0.16813781	0.02827032	0
**Palestinian**	0.21530503	0.04635626	0
**Druze**	0.21907057	0.04799192	0
**Bedouin**	0.1928931	0.03720775	0
**Adygei**	0.1649932	0.02722276	9.53E-243
**BergamoItalian_Tuscan**	0.20588759	0.0423897	0
**Sardinian**	0.21234659	0.04509107	0
**Basque**	0.21153356	0.04474645	0
**French**	0.22424484	0.05028575	0
**Orcadian**	0.1769971	0.03132797	8.95E-276
**Russian**	0.17270263	0.0298262	1.18E-302
**Makrani**	0.1804534	0.03256343	0
**Sindhi**	0.18332147	0.03360676	0
**Balochi**	0.18597233	0.03458571	0
**Brahui**	0.19563697	0.03827382	0
**Hazara**	0.17675899	0.03124374	7.84E-301
**Pathan**	0.18677213	0.03488383	0
**Burusho**	0.17608256	0.03100507	1.3723108091009e-317
**Kalash**	0.2503178	0.062659	0
**Uygur**	0.17682151	0.03126585	2.48E-239
**Xibo_Mongolian**	0.23409674	0.05480128	0
**Oroqen_Hezhen_Daur**	0.23733334	0.05632712	0
**Yakut**	0.26052163	0.06787152	0
**Japanese**	0.22845015	0.05218947	0
**Han**	0.25153117	0.06326793	0

**NorthernHan_Tu**	0.21461105	0.0460579	0
**She_Miao_Tujia**	0.24431667	0.05969064	0
**Naxi_Yi**	0.21039097	0.04426436	0
**Dai_Lahu**	0.23418859	0.0548443	0
**Pima**	0.28077905	0.07883687	0
**Maya**	0.32331967	0.10453561	0
**Surui_Karitiana**	0.28452489	0.08095441	0
**PapuanHighlands_PapuanSepik**	0.24935343	0.06217713	0
**Bougainville**	0.19203861	0.03687883	7.31E-265

Table S14: Correlation between empirical p-values of MA-genes calculated by $\mathbf{F_{ST}}$ and $\boldsymbol{Relate}$. Correlation between empirical p-values of MA-genes calculated by F_{ST} (as calculated between Yoruba and each test population) and $Relate$ for all populations. Populations with positive correlations with p-value < 0.05 are given in italics. Pvalue=0 indicates pvalue below 1e-318.

Population	Correlation	Rsquared	Pvalue
**Ju|'hoan**	*0.07840859*	*0.00614791*	*0.0513838*
**BantuSouthAfrica_BantuKenya**	0.1399856	0.01959597	3.54E-08
**Mbuti**	0.11269175	0.01269943	2.51E-05
**Biaka**	0.19952424	0.03980992	6.53E-14
**Mandenka**	*0.00263365*	*6.94E-06*	*0.91958327*
**Mozabite**	0.22675485	0.05141776	2.23E-33
**Palestinian**	0.13053672	0.01703984	5.81E-12
**Druze**	0.30606577	0.09367626	4.21E-60
**Bedouin**	0.14237412	0.02027039	3.89E-15
**Adygei**	0.1376565	0.01894931	5.63E-12
**BergamoItalian_Tuscan**	0.26151534	0.06839027	6.39E-46
**Sardinian**	0.14279613	0.02039073	2.62E-14
**Basque**	0.23054803	0.0531524	1.19E-32
**French**	0.24662259	0.0608227	2.51E-37
**Orcadian**	0.20221591	0.04089127	3.75E-23
**Russian**	0.24381588	0.05944618	6.08E-38
**Makrani**	0.21591664	0.04662	2.19E-32
**Sindhi**	0.1176469	0.01384079	6.78E-10
**Balochi**	0.16826001	0.02831143	3.86E-19
**Brahui**	0.10323381	0.01065722	4.19E-08
**Hazara**	0.19161027	0.0367145	4.35E-20
**Pathan**	0.14826994	0.02198397	1.40E-14
**Burusho**	0.17353564	0.03011462	1.05E-19
**Kalash**	0.19137435	0.03662414	6.93E-21
**Uygur**	0.15219298	0.0231627	2.90E-12
**Xibo_Mongolian**	0.19156455	0.03669697	2.81E-21
**Oroqen_Hezhen_Daur**	0.25448203	0.0647611	4.29E-34
**Yakut**	0.24049998	0.05784024	3.47E-34
**Japanese**	0.20340443	0.04137336	2.21E-21
**Han**	0.25333765	0.06417997	3.36E-34
**NorthernHan_Tu**	0.16345559	0.02671773	8.62E-15
**She_Miao_Tujia**	0.27300488	0.07453166	4.03E-40
**Naxi_Yi**	0.27932745	0.07802383	3.93E-40
**Dai_Lahu**	0.25160567	0.06330541	3.83E-35
**Pima**	0.18042089	0.0325517	1.91E-13
**Maya**	0.20890365	0.04364074	3.93E-25
**Surui_Karitiana**	0.29465741	0.08682299	2.79E-35
**PapuanHighlands_PapuanSepik**	0.24589531	0.0604645	1.01E-32
**Bougainville**	0.25884529	0.06700088	2.14E-63

Table S15: Correlation between empirical p-values of MA-genes calculated by F_{ST} and $Relate$, pruned by F_{ST} p-value. Correlation between empirical p-values of MA-

genes calculated by $\mathrm{F_{ST}}$ (as calculated between Yoruba and each test population) and $Relate$ for all populations. To allow a better comparison between methods, we prune SNPs to only include those in the 5% tail of the empirical $\mathrm{F_{ST}}$ distribution (selection over the lifetime of a mutation - as captured by $Relate$ – does not necessitate the signatures of differential selection that are identified by $\mathrm{F_{ST}}$) with lower frequency in Yoruba (indicating selection in the test population). Populations with positive correlations with p-value < 0.05 are given in italics.

Supplemental References

1. Haller, B.C., and Messer, P.W. (2019). SLiM 3: Forward Genetic Simulations Beyond the Wright-Fisher Model. Mol Biol Evol. https://doi.org/10.1093/molbev/msy228.
2. Bergström, A., McCarthy, S.A., Hui, R., Almarri, M.A., Ayub, Q., Danecek, P., Chen, Y., Felkel, S., Hallast, P., Kamm, J., et al. (2020). Insights into human genetic variation and population history from 929 diverse genomes. Science (1979) *367*. https://doi.org/10.1126/SCIENCE.AAY5012/SUPPL_FILE/AAY5012-BERGSTROM-SM.PDF.
3. Gravel, S., Henn, B.M., Gutenkunst, R.N., Indap, A.R., Marth, G.T., Clark, A.G., Yu, F., Gibbs, R.A., and Bustamante, C.D. (2011). Demographic history and rare allele sharing among human populations. Proc Natl Acad Sci U S A. https://doi.org/10.1073/pnas.1019276108.

4. Gravel, S., Zakharia, F., Moreno-Estrada, A., Byrnes, J.K., Muzzio, M., Rodriguez-Flores, J.L., Kenny, E.E., Gignoux, C.R., Maples, B.K., Guiblet, W., et al. (2013). Reconstructing Native American Migrations from Whole-Genome and Whole-Exome Data. PLoS Genet. https://doi.org/10.1371/journal.pgen.1004023.
5. Schlebusch, C.M., Sjödin, P., Breton, G., Günther, T., Naidoo, T., Hollfelder, N., Sjöstrand, A.E., Xu, J., Gattepaille, L.M., Vicente, M., et al. (2020). Khoe-San Genomes Reveal Unique Variation and Confirm the Deepest Population Divergence in Homo sapiens. Mol Biol Evol *37*, 2944–2954. https://doi.org/10.1093/MOLBEV/MSAA140.
6. Boyko, A.R., Williamson, S.H., Indap, A.R., Degenhardt, J.D., Hernandez, R.D., Lohmueller, K.E., Adams, M.D., Schmidt, S., Sninsky, J.J., Sunyaev, S.R., et al. (2008). Assessing the evolutionary impact of amino acid mutations in the human genome. PLoS Genet. https://doi.org/10.1371/journal.pgen.1000083.
7. Ragsdale, A.P., Weaver, T.D., Atkinson, E.G., Hoal, E.G., Möller, M., Henn, B.M., and Gravel, S. (2023). A weakly structured stem for human origins in Africa. Nature 2023 617:7962 *617*, 755–763. https://doi.org/10.1038/s41586-023-06055-y.
8. Kim, B.Y., Huber, C.D., and Lohmueller, K.E. (2017). Inference of the distribution of selection coefficients for new nonsynonymous mutations using large samples. Genetics. https://doi.org/10.1534/genetics.116.197145.
9. Lipson, M., Sawchuk, E.A., Thompson, J.C., Oppenheimer, J., Tryon, C.A., Ranhorn, K.L., de Luna, K.M., Sirak, K.A., Olalde, I., Ambrose, S.H., et al. (2022). Ancient DNA and deep population structure in sub-Saharan African foragers. Nature 2022 603:7900 *603*, 290–296. https://doi.org/10.1038/s41586-022-04430-9.
10. Voight, B.F., Kudaravalli, S., Wen, X., and Pritchard, J.K. (2006). A Map of Recent Positive Selection in the Human Genome. https://doi.org/10.1371/journal.pbio.0040072.
11. Schlebusch, C.M., Malmström, H., Günther, T., Sjödin, P., Coutinho, A., Edlund, H., Munters, A.R., Vicente, M., Steyn, M., Soodyall, H., et al. (2017). Southern African ancient genomes estimate modern human divergence to 350,000 to 260,000 years ago. Science (1979) *358*, 652–655. https://doi.org/10.1126/SCIENCE.AAO6266/SUPPL_FILE/AAO6266_SCHLEBUSCH_SM.PDF.
12. Ferrer-Admetlla, A., Liang, M., Korneliussen, T., and Nielsen, R. (2014). On Detecting Incomplete Soft or Hard Selective Sweeps Using Haplotype Structure. Mol Biol Evol. https://doi.org/10.1093/molbev/msu077.
13. Sabeti, P.C., Varilly, P., Fry, B., Lohmueller, J., Hostetter, E., Cotsapas, C., Xie, X., Byrne, E.H., Mccarroll, S.A., Gaudet, R., et al. (2007). Genome-wide detection and characterization of positive selection in human populations. *449*. https://doi.org/10.1038/nature06250.
14. Szpiech, Z.A., Novak, T.E., Bailey, N.P., and Stevison, L.S. (2020). High-altitude adaptation in rhesus macaques. Preprint at bioRxiv, https://doi.org/10.1101/2020.05.19.104380 https://doi.org/10.1101/2020.05.19.104380.

15. Weir, B.S., and Cockerham, C.C. (1984). Estimating F-Statistics for the Analysis of Population Structure. Evolution (N Y) *38*, 1358. https://doi.org/10.2307/2408641.
16. Speidel, L., Forest, M., Shi, S., and Myers, S.R. (2019). A method for genome-wide genealogy estimation for thousands of samples. Nat Genet *51*, 1321–1329. https://doi.org/10.1038/s41588-019-0484-x.
17. Field, Y., Boyle, E.A., Telis, N., Gao, Z., Gaulton, K.J., Golan, D., Yengo, L., Rocheleau, G., Froguel, P., McCarthy, M.I., et al. (2016). Detection of human adaptation during the past 2000 years. Science (1979) *354*, 760–764. https://doi.org/10.1126/science.aag0776.
18. Szpiech, Z.A., and Hernandez, R.D. (2014). Selscan: An efficient multithreaded program to perform EHH-based scans for positive selection. Mol Biol Evol. https://doi.org/10.1093/molbev/msu211.
19. Danecek, P., Auton, A., Abecasis, G., Albers, C.A., Banks, E., DePristo, M.A., Handsaker, R.E., Lunter, G., Marth, G.T., Sherry, S.T., et al. (2011). The variant call format and VCFtools. Bioinformatics. https://doi.org/10.1093/bioinformatics/btr330.
20. Daub, J.T., Hofer, T., Cutivet, E., Dupanloup, I., Quintana-Murci, L., Robinson-Rechavi, M., and Excoffier, L. (2013). Evidence for Polygenic Adaptation to Pathogens in the Human Genome. Mol Biol Evol *30*, 1544–1558. https://doi.org/10.1093/molbev/mst080.
21. Tintle, N.L., Borchers, B., Brown, M., and Bekmetjev, A. (2009). Comparing gene set analysis methods on single-nucleotide polymorphism data from Genetic Analysis Workshop 16. BMC Proceedings 2009 3:7 *3*, 1–5. https://doi.org/10.1186/1753-6561-3-S7-S96.
22. Serdar, C.C., Cihan, M., Yücel, D., and Serdar, M.A. (2021). Sample size, power and effect size revisited: simplified and practical approaches in pre-clinical, clinical and laboratory studies. Biochem Med (Zagreb) *31*, 1–27. https://doi.org/10.11613/BM.2021.010502.
23. Subramanian, S. (2016). The effects of sample size on population genomic analyses - implications for the tests of neutrality. BMC Genomics *17*, 1–13. https://doi.org/10.1186/S12864-016-2441-8/FIGURES/7.
24. Purcell, S., Neale, B., Todd-Brown, K., Thomas, L., Ferreira, M.A.R., Bender, D., Maller, J., Sklar, P., De Bakker, P.I.W., Daly, M.J., et al. (2007). PLINK: A tool set for whole-genome association and population-based linkage analyses. Am J Hum Genet. https://doi.org/10.1086/519795.
25. Hou, X., Zhang, X., Li, X., Huang, T., Li, W., Zhang, H., Huang, H., and Wen, Y. (2022). Genomic insights into the genetic structure and population history of Mongolians in Liaoning Province. Front Genet *13*, 947758. https://doi.org/10.3389/FGENE.2022.947758/BIBTEX.
26. Patin, E., Lopez, M., Grollemund, R., Verdu, P., Harmant, C., Quach, H., Laval, G., Perry, G.H., Barreiro, L.B., Froment, A., et al. (2017). Dispersals and genetic adaptation of Bantu-speaking populations in Africa and North America. Science *356*, 543–546. https://doi.org/10.1126/SCIENCE.AAL1988.
27. Bai, H., Guo, X., Zhang, D., Narisu, N., Bu, J., Jirimutu, J., Liang, F., Zhao, X., Xing, Y., Wang, D., et al. (2014). The genome of a Mongolian individual reveals the genetic

imprints of Mongolians on modern human populations. Genome Biol Evol *6*, 3122–3136. https://doi.org/10.1093/GBE/EVU242.
28. Racimo, F., Marnetto, D., and Huerta-Sánchez, E. (2017). Signatures of Archaic Adaptive Introgression in Present-Day Human Populations. Mol Biol Evol *34*, 296–317. https://doi.org/10.1093/MOLBEV/MSW216.
29. Skov, L., Coll Macià, M., Sveinbjörnsson, G., Mafessoni, F., Lucotte, E.A., Einarsdóttir, M.S., Jonsson, H., Halldorsson, B., Gudbjartsson, D.F., Helgason, A., et al. (2020). The nature of Neanderthal introgression revealed by 27,566 Icelandic genomes. Nature 2020 582:7810 *582*, 78–83. https://doi.org/10.1038/s41586-020-2225-9.
30. Roca-Umbert, A., Garcia-Calleja, J., Vogel-González, M., Fierro-Villegas, A., Ill-Raga, G., Herrera-Fernández, V., Bosnjak, A., Muntané, G., Gutiérrez, E., Campelo, F., et al. (2023). Human genetic adaptation related to cellular zinc homeostasis. PLoS Genet *19*, e1010950. https://doi.org/10.1371/JOURNAL.PGEN.1010950.
31. Vaughn, A.H., and Nielsen, R. (2024). Fast and Accurate Estimation of Selection Coefficients and Allele Histories from Ancient and Modern DNA. Mol Biol Evol *41*. https://doi.org/10.1093/MOLBEV/MSAE156.
32. Yates, B., Braschi, B., Gray, K.A., Seal, R.L., Tweedie, S., and Bruford, E.A. (2017). Genenames.org: The HGNC and VGNC resources in 2017. Nucleic Acids Res. https://doi.org/10.1093/nar/gkw1033.
33. White, L., Romagné, F., Müller, E., Erlebach, E., Weihmann, A., Parra, G., Andrés, A.M., and Castellano, S. (2015). Genetic adaptation to levels of dietary selenium in recent human history. Mol Biol Evol *32*, 1507–1518. https://doi.org/10.1093/molbev/msv043.
34. Engelken, J., Espadas, G., Mancuso, F.M., Bonet, N., Scherr, A.L., Jímenez-Álvarez, V., Codina-Solà, M., Medina-Stacey, D., Spataro, N., Stoneking, M., et al. (2016). Signatures of evolutionary adaptation in quantitative trait loci influencing trace element homeostasis in liver. Mol Biol Evol *33*, 738–754. https://doi.org/10.1093/molbev/msv267.
35. Wishart, D.S., Tzur, D., Knox, C., Eisner, R., Guo, A.C., Young, N., Cheng, D., Jewell, K., Arndt, D., Sawhney, S., et al. (2007). HMDB: The human metabolome database. Nucleic Acids Res. https://doi.org/10.1093/nar/gkl923.
36. Dib, M.J., Elliott, R., and Ahmadi, K.R. (2019). A critical evaluation of results from genome-wide association studies of micronutrient status and their utility in the practice of precision nutrition. British Journal of Nutrition. https://doi.org/10.1017/S0007114519001119.
37. Kovacs, G., Montalbetti, N., Franz, M.C., Graeter, S., Simonin, A., and Hediger, M.A. (2013). Human TRPV5 and TRPV6: Key players in cadmium and zinc toxicity. Cell Calcium *54*, 276–286. https://doi.org/10.1016/j.ceca.2013.07.003.
38. López Herráez, D., Bauchet, M., Tang, K., Theunert, C., Pugach, I., Li, J., Nandineni, M.R., Gross, A., Scholz, M., and Stoneking, M. (2009). Genetic Variation and Recent Positive Selection in Worldwide Human Populations: Evidence from Nearly 1 Million SNPs. PLoS One *4*, e7888. https://doi.org/10.1371/journal.pone.0007888.

39. Hughes, D.A., Tang, K., Strotmann, R., Schöneberg, T., Prenen, J., Nilius, B., and Stoneking, M. (2008). Parallel selection on TRPV6 in human populations. PLoS One *3*. https://doi.org/10.1371/journal.pone.0001686.
40. Muckenthaler, M.U., Galy, B., and Hentze, M.W. (2008). Systemic iron homeostasis and the iron-responsive element/iron-regulatory protein (IRE/IRP) regulatory network. Annu Rev Nutr *28*, 197–213. https://doi.org/10.1146/ANNUREV.NUTR.28.061807.155521.
41. Khanal, R.C., and Nemere, I. (2008). Regulation of intestinal calcium transport. Annu Rev Nutr *28*, 179–196. https://doi.org/10.1146/ANNUREV.NUTR.010308.161202.
42. Stauber, T., and Jentsch, T.J. (2013). Chloride in vesicular trafficking and function. Annu Rev Physiol *75*, 453–477. https://doi.org/10.1146/ANNUREV-PHYSIOL-030212-183702.
43. Chang, A.R., and Anderson, C. (2017). Dietary Phosphorus Intake and the Kidney. Annu Rev Nutr *37*, 321–346. https://doi.org/10.1146/ANNUREV-NUTR-071816-064607.
44. Jain, G., Ong, S., and Warnock, D.G. (2013). Genetic Disorders of Potassium Homeostasis. Semin Nephrol *33*, 300–309. https://doi.org/10.1016/J.SEMNEPHROL.2013.04.010.
45. Reiss, J., and Hahnewald, R. (2011). Molybdenum cofactor deficiency: Mutations in GPHN, MOCS1, and MOCS2. Hum Mutat *32*, 10–18. https://doi.org/10.1002/HUMU.21390.
46. Horning, K.J., Caito, S.W., Tipps, K.G., Bowman, A.B., and Aschner, M. (2015). Manganese Is Essential for Neuronal Health. Annu Rev Nutr *35*, 71. https://doi.org/10.1146/ANNUREV-NUTR-071714-034419.
47. Bateman, A., Martin, M.J., Orchard, S., Magrane, M., Ahmad, S., Alpi, E., Bowler-Barnett, E.H., Britto, R., Bye-A-Jee, H., Cukura, A., et al. (2023). UniProt: the Universal Protein Knowledgebase in 2023. Nucleic Acids Res *51*, D523–D531. https://doi.org/10.1093/NAR/GKAC1052.
48. Freitas, S.R.S. (2018). Molecular Genetics of Salt-Sensitivity and Hypertension: Role of Renal Epithelial Sodium Channel Genes. Am J Hypertens *31*, 172–174. https://doi.org/10.1093/AJH/HPX184.
49. Rossier, B.C., Pradervand, S., Schild, L., and Hummler, E. (2002). Epithelial sodium channel and the control of sodium balance: Interaction between genetic and environmental factors. Annu Rev Physiol *64*, 877–897. https://doi.org/10.1146/ANNUREV.PHYSIOL.64.082101.143243/CITE/REFWORKS.
50. Houillier, P. (2014). Mechanisms and regulation of renal magnesium transport. Annu Rev Physiol *76*, 411–430. https://doi.org/10.1146/ANNUREV-PHYSIOL-021113-170336/CITE/REFWORKS.
51. Fishilevich, S., Nudel, R., Rappaport, N., Hadar, R., Plaschkes, I., Stein, T.I., Rosen, N., Kohn, A., Twik, M., Safran, M., et al. (2017). GeneHancer: genome-wide integration of enhancers and target genes in GeneCards. Database *2017*, 1–17. https://doi.org/10.1093/DATABASE/BAX028.

52. Phan, L., Zhang, H., Wang, Q., Villamarin, R., Hefferon, T., Ramanathan, A., and Kattman, B. (2025). The evolution of dbSNP: 25 years of impact in genomic research. Nucleic Acids Res *53*, D925–D931. https://doi.org/10.1093/NAR/GKAE977.
53. Rentzsch, P., Witten, D., Cooper, G.M., Shendure, J., and Kircher, M. (2019). CADD: predicting the deleteriousness of variants throughout the human genome. Nucleic Acids Res *47*, D886–D894. https://doi.org/10.1093/NAR/GKY1016.
