# Supplementary material for: Global impact of micronutrients in modern human evolution
Source: Am J Hum Genet. 2025 Sep 10;112(10):2538–61. doi: 10.1016/j.ajhg.2025.08.005 (PMC12696500; doi:10.1016/j.ajhg.2025.08.005)
Supplement: Document S2. Article plus supplemental information [file mmc6.pdf]

ARTICLE

Global impact of micronutrients in modern human evolution

Authors

Jasmin Rees, Sergi Castellano, Aida M. Andrés

Correspondence

jasmin.rees@pennmedicine.upenn.edu (J.R.),
a.andres@ucl.ac.uk (A.M.A.)

In this study, Rees et al. evaluate the role of dietary micronutrients in driving local genetic adaptation and population differentiation in humans. The authors find evidence that micronutrient-associated positive selection has shaped human genetic diversity and highlight the potential health risks of changing levels of dietary micronutrients across the globe.

Rees et al., 2025, The American Journal of Human Genetics *112*, 2538–2561
October 2, 2025

© 2025 The Authors. Published by Elsevier Inc. on behalf of American Society of Human Genetics.

https://doi.org/10.1016/j.ajhg.2025.08.005

CellPress

ARTICLE

Global impact of micronutrients in modern human evolution

Jasmin Rees,[1,2,4,*] Sergi Castellano,[1,3] and Aida M. Andrés[2,*]

Summary

Micronutrients are essential components of the human diet, but dietary levels above or below their narrow, recommended range are harmful. Deficiencies increase the risk of stunted growth and metabolic, infectious, and respiratory disorders, and have likely been pervasive in human history, as local soils poor in micronutrients are widespread. Deficiencies are also common today, affecting approximately 2 billion people. Limited evidence exists for selenium, zinc, iodine, and iron deficiencies driving local adaptation in a few human populations, but the broader potential role of micronutrients in shaping modern human evolution remains unclear. Here, we investigate signatures of positive selection in 276 genes associated with 13 micronutrients and evaluate whether human adaptation across global populations has been driven by micronutrients. We identify known and previously undescribed instances of rapid local adaptation in micronutrient-associated genes in particular populations, including previously undescribed individual signatures of adaptation across most of the world. Further, we identify signatures of oligogenic-positive selection in multiple populations at different geographic and temporal scales, with some recapitulating known associations of geology and micronutrient deficiencies. We conclude that micronutrient deficiencies have likely shaped worldwide human evolution more directly than previously appreciated and, given the ongoing depletion of soil quality from over-farming and climate change, caution that some populations may be at higher risk of suffering from micronutrient-driven disorders going forward.

Introduction

The composition of the human diet varies widely across populations, due to both environmentally induced factors and recently introduced cultural practices, most notably the Neolithic revolution.[1–4] In some cases, components of the diet can act as selective pressures, driving local genetic adaptations to mitigate novel dietary stressors. Selective pressures include culturally introduced practices, such as milk drinking in adulthood,[5–8] but also challenges posed by local environments, such as nutritionally poor diets,[9] high fatty acid content of local foods,[10,11] or the presence of toxic[12–14] or deficient[13–19] levels of chemicals in the diet via their accumulation in local plants and animals. Micronutrients are particularly important chemical elements of the human diet and directly dependent on local geology, and they play a central role in a multitude of physiological processes (e.g., metabolism, the maintenance of tissue function, immunity, and healthy growth or development[20–26]).

Micronutrient is a broad term that includes vitamins and minerals that are essential in very small and specific amounts. They cannot be synthesized within the body (with the exception of vitamin D[24]) and must instead be absorbed from the diet. However, global soils are highly variable in their levels or bioavailability of micronutrients, even among proximal locations.[27–29] Soil geology has therefore likely had a tremendous effect on the micronutrient composition of local human diets throughout much of our species' evolution. In more recent history, human cultural practices, such as agriculture, overfarming, or food practices, may have also disturbed the levels of dietary micronutrients.[4,27] Potential differences in dietary micronutrients across human populations are of great importance because even slight deviations from their narrow, required range in the diet can result in micronutrient deficiency or toxicity.[20,21,23,24,26,30]

Micronutrient deficiencies impair mental and physical development and are particularly dangerous in early development.[20,21,26] The most common deficiencies, those in zinc, iron, iodine, folate, and vitamin A, are associated with increases in birth defects, vision loss, and poor cognitive function and development.[31–35] Micronutrient deficiency is also pernicious outside key periods of development, increasing the risk of various metabolic, infectious, and respiratory diseases, as well as directly causing anemia, goiter, Kashin-Beck disease, and Keshan disease.[29,36,37] Diseases induced or exacerbated by micronutrient deficiencies have been insidious across recorded history and likely throughout human evolutionary history, and they also remain a dominant public health concern today.[20,23,26,38,39] Across the globe, 178 million children under the age of 5 years are estimated to have stunted growth due to micronutrient deficiency.[21] Excess levels of dietary micronutrients are relatively rare, and typically result in gastrointestinal stress, nausea, vomiting, and diarrhea.[30] Most cases of toxicity stem from poor industrial practices poisoning local soils or as a result of excess

[1]Great Ormond Street Institute of Child Health, University College London, London, UK; [2]UCL Genetics Institute, Department of Genetics, Evolution and Environment, University College London, London, UK; [3]UCL Genomics, University College London, London, UK
[4]Present address: Department of Genetics, University of Pennsylvania, Philadelphia, PA 19104, USA
*Correspondence: jasmin.rees@pennmedicine.upenn.edu (J.R.), a.andres@ucl.ac.uk (A.M.A.)
https://doi.org/10.1016/j.ajhg.2025.08.005.
© 2025 The Authors. Published by Elsevier Inc. on behalf of American Society of Human Genetics.
This is an open access article under the CC BY license (http://creativecommons.org/licenses/by/4.0/).

supplementation[30,40–44] and were likely uncommon throughout most of human history.

Indeed, the limited existing evidence only supports a role of soil-induced micronutrient deficiency as a selective pressure in humans. Selenium-deficient soil in East Asia has been linked to signatures of positive selection in selenium-associated genes such *DIO2* (MIM:601413), *SELENOS* (MIM: 607918), *GPX1* (MIM: 138320), *CELF1* (MIM: 601074), and *SEPHS2* (MIM: 606218).[15] Iodine-deficient rainforest soil has been suggested to drive signatures of positive selection in iodine-associated *TRIP4* (MIM: 604501) and *IYD* (MIM: 612025) in the African rainforest hunter-gatherer Biaka population.[16] Additionally, the rainforest hunter-gatherer Efe populations in Central Africa have lower incidence of goiter, an enlargement of the thyroid gland caused by iodine deficiency, than the neighboring Bantu-speaking populations occupying the same iodine-deficient soil of the Ituri forest (42.9% vs. 9.1%[45]), suggesting the presence of an adaptive response to iodine deficiency in the rainforest hunter-gatherer populations. Finally, low levels of zinc in soil and crops in East Asia are correlated with the frequency of a haplotype of the zinc-transporter *SLC30A9* (MIM: 604604) that has signatures of positive selection in some East Asian populations.[19]

However, the overall relevance of micronutrients as selective forces in human evolution remains unknown. First, it is unclear if only micronutrient deficiencies have acted as effective selective pressures in human evolutionary history or if toxic levels of micronutrients, induced by geology or human cultural practices, has also driven genetic adaptation. Second, it is unknown if all micronutrients have acted as effective selective pressures in human evolutionary history or if this is the case for only a few key minerals or trace metals. Finally, it is unclear whether putative genetic adaptation is geographically and temporally widespread or restricted to a few populations under extreme dietary levels of a particular micronutrient. Given the critical role of micronutrients in human health and their variation in dietary levels across human populations, understanding their influence in genetic variation and population differentiation is important to understand not only human evolution but also contemporary health disparities.

Here, we evaluate the role of dietary micronutrients as a selective force in modern human evolution by studying the patterns of genetic variation in gene sets associated with the uptake, metabolism, or regulation of 13 micronutrients in 40 geographically diverse modern human populations. By analyzing about ~300 genes known to play a functional role in the metabolism and function of these micronutrients, with measures of allele-frequency differentiation and genealogical inferences, we identify genomic signatures of positive selection both at the level of individual genes and of the set of genes related to each micronutrient. We find widespread individual signatures of positive selection in multiple genes associated with different micronutrients at different time points in human evolutionary history, suggesting that micronutrients have been a powerful selective force in modern humans.

Material and methods

Simulation design

We first aim to assess, using simulations, which methods are able to identify even subtle signatures of positive selection in the genome. The forward-simulator SLiM[46] was used to simulate 100-kbp gene regions undergoing positive selection on standing genetic variation in one of four focal populations: African, European, East Asian, and American (see Note S1). These simulations feature a simplified demographic model, for example by not including deep structure in Africa[47–50] (see Note S1), and are therefore most informative for power in populations with demographic histories similar to those modeled here.

To approximate selection on standing variation, at one of four time points (1, 5, 10, and 40 kya), a single polymorphic allele segregating in the focal population (at frequency between 0.1 and 0.15) was tagged and given a selection coefficient drawn from $U(0,1)$. A burn-in period was first simulated between 1.66 mya and 70 kya (where population-genetic parameters were scaled to reduce CPU time[46]) before the forward simulation from 70 kya-present covering all population splits, expansions, and migrations as described in Figure 1A, as well as the onset of selection in one time point in one population (Note S1). For each scenario of positive selection (16 combinations of one selection time point in one metapopulation), ~10,000 simulations where the selected allele remained polymorphic in the focal population were completed. A matched number of simulations were run under neutrality (Note S1). For each successful simulation (those where the selected allele was not lost), VCF files of 50 individuals for each metapopulation were generated.

Assessing accuracy of methods

We tested the accuracy of seven methods to identify signatures of weak positive selection in the simulated genetic data: iHS, nSL, XPEHH, XPnSL, F_{ST}, tSDS, and Relate (see Note S2[53–59]). For each method, we identified SNPs that fall in the extreme 5% tail of neutral distributions as those with signatures of positive selection (where neutral distributions are built from neutral simulations; Note S2; Figures S2–S9). To evaluate accuracy, we calculated true-positive rate (TPR; percentage of truly selected SNPs that fall in the extreme 5% tails of the neutral distribution), false-negative rate (FNR; percentage of truly selected SNPs that fall outside the extreme 5% tails of the neutral distribution, or 1-TPR), and false-positive rate (FPR; the percentage of non-selected SNPs that fall in the extreme 5% tails of the neutral distribution; discussed in Note S2). As an additional exploration of the recent Relate method,[56] we also compared the accuracy of using the raw output ($-\log 10\ p$ value) to using the tails of the neutral distribution to identify SNPs with signatures of positive selection (Note S2, Figures S10 and S11).

To approximate polygenic selection, we combined simulated gene regions into gene sets. We then evaluated the accuracy of the gene set enrichment method SUMSTAT[60] to identify polygenic selection. The SUMSTAT method is as follows: for each gene set, the *p*value of the SNP with the strongest evidence of

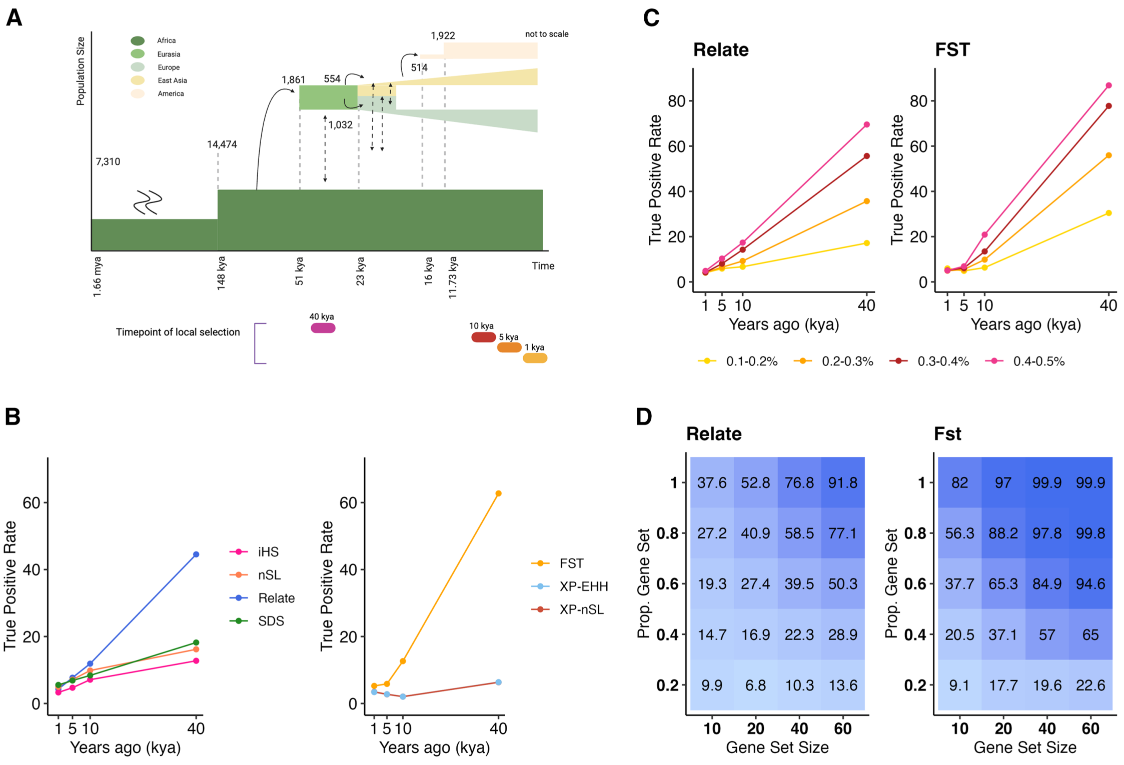

Figure 1. Overview of simulation framework and power analysis

(A) Schematic overview of the simulation design, including demographic model (top; where dashed lines correspond to time points of population splits and changes of effective population size, based on models from the literature[51,52]) and the variable time points of positive selection (bottom).

(B) Percentage of selected SNPs identified as under positive selection (according to the tail of the neutral distribution; *y* axis) at four different time points (*x* axis) for different methods tested on individual populations (left) and between population pairs (right). Both plots represent the simulated African population (other simulated populations for methods tested on individual populations and between population pairs are given in Figures S2 and S3, respectively).

(C) Percentage of selected SNPs identified as under positive selection (according to the tail of the neutral distribution; *y* axis) at four time points (*x* axis) for Relate (left) and F_{ST} (right) by simulated selection coefficient (see legend), for the African population (other populations shown in Figures S4 and S5).

(D) Percentage of selected gene sets identified as under positive selection (according to the tail of the neutral distribution; numbers inside matrix) for gene sets of different size (*x* axis) and different proportion of gene regions under positive selection (*y* axis) for Relate (left) and F_{ST} (right). Shown for positive selection at 40 kya in the African population; all other time points of selection and simulated populations shown in Figures S12 and S13. Created in BioRender. Rees, J. (2025) https://BioRender.com/o76t447.

positive selection (as calculated from the neutral distribution) in each gene region is extracted and summed to give a final score (or SUMSTAT value). SUMSTAT values are compared to the neutral distribution (as generated from applying the SUMSTAT method to 1,000 random gene sets simulated under neutrality), and the gene sets with SUMSTAT values in the extreme 5% tail are identified as those with signatures of positive selection. For our two most powerful methods (F_{ST} and Relate), we evaluated the accuracy of the SUMSTAT method on gene sets of various sizes (10, 20, 40, and 60) and gene sets with varying proportions of true selected gene regions (20%, 40%, 60%, 80%, and 100% gene regions within a gene set under a selection) to consider a range of selection scenarios (Figures S12 and S13).

Micronutrient-associated gene sets

Micronutrient-associated (MA) gene sets were generated from relevant datasets (e.g., Human Metabolome Database[61]) and literature searches for each of the 13 micronutrients. The literature used includes clinical studies, functional biochemical studies, and studies identifying signatures of natural selection (see Data S1). Signatures of natural selection have only been identified in genes associated with selenium, zinc, iron, calcium, and iodine as identified from either genome-wide selection scans or studies of positive selection on gene sets functionally associated with their respective micronutrient.[15,16,18,19,62–64] All such genes are considered because of their functional associations and make up only a small proportion of the total MA genes (17 of the 276 MA genes, 6.2%). Hence, the ascertainment bias from the literature search in this regard is minor.

In total, we identified 276 genes associated with 13 micronutrients, of which 269 remained after filtering out segments of the genome according to an accessibility mask ([65]; see below). The resulting MA genes are largely randomly distributed along the human genome; there are only 11 pairs of MA genes within 10 kbp of each other (Table S11), and, in these few cases, signatures of positive selection cannot be strictly assigned to either of the genes in the pair. Further, some genes are associated with multiple micronutrients; for analyses where this is undesirable (see results), we generated non-overlapping gene sets, where no

overlap exists among sets and each gene is assigned to only the micronutrient for which we consider its most important functional association (Data S1).

We also generated a database of background genomic regions matched to each MA gene. For each MA gene, we sampled 1,500 regions from the human genome, each beginning at the starting genomic coordinate of a random human gene and then matched in exact length to the respective MA gene. Here, we are agnostic to the function of candidate SNPs and match the background-gene regions to the full length of the MA gene. To account for the SNP density of genes within each set, only the 1,000 gene regions with the SNP densities closest to each associated MA gene were retained. The SNP densities were from Yoruba individuals[65] because matching SNP densities to those of the target populations could result in background genes with unusual evolutionary histories if MA genes are under selection. This results in a random set of gene regions (referred to as background-gene regions) that were used to represent the random genomic background. Seven MA genes have SNP densities significantly above those of their respective background-gene regions, but these do not cluster by micronutrient (Table S12). Background-gene regions were then grouped into gene sets of sizes matched to those of their respective MA-gene sets. These are referred to as background-gene sets.

The population-genetic dataset

Whole-genome sequence data were collected from the HGDP dataset.[65] Here, we use the assigned populations as proxies for genetic ancestry groups. Small population samples sizes can generate noisy allele frequencies, so 22 populations with small sample sizes (sample size 12 or below) were merged with populations that were genetically very similar and either geographically close or separated by very recent migrations (see principal-component analysis [PCA] and admixture analyses; Figures S14–S20 and Note S3). Three populations had sample sizes below 12 but did not group naturally by geography or genetics. Of these, only the African Ju|'hoan (previously referred to as San by the HGDP; $n = 6$[65]) were retained in our dataset given their unique genetic history (Note S3). The final dataset comprises 913 individuals from 40 populations, over eight major geographic areas (Africa, the Middle East, Europe, East Asia, Central-South Asia, Oceania, and the Americas) and represents a significant proportion of human geographic and cultural diversity (Table S13).

Identifying the genomic signatures of positive selection

We used two methods to identify the genetic signatures of positive selection in single loci (F_{ST} and Relate[56,59]). These methods have high power to identify strong positive selection or selection on *de novo* mutation but also perform considerably better than additional considered methods (see "accessing accuracy of methods") when considering weaker selection and/or selection on standing variation. Importantly, the signatures of positive selection identified by F_{ST} and Relate are related but subtly different. SNPs with extreme F_{ST} values are the most highly differentiated between populations; a key property of F_{ST}, therefore, is that it can only identify signatures of positive selection that have arisen following the split of the two populations used in each pairwise calculation. Relate, on the other hand, identifies sites that have risen to an unusual frequency, given their age and the number of lineages present when they first arose, over the entire inferred history of the locus in each population. In reality, this is up to the time of the common ancestor of all populations used in the genealogical inference. Therefore, it is not expected that these statistics will necessarily identify the same SNPs as having signatures of positive selection and, in theory, using both allows us to identify adaptation that has occurred differentially between populations and within the specific inferred history of an individual population.

Before calculating per-SNP F_{ST}, we filtered the VCF files to remove indels, retain only biallelic sites, and retain only the regions given in the accessibility mask.[65] This mask is based upon the 1000 Genomes Project's strict mask (which removes regions of low coverage and mapping quality[66]) while also removing regions of excess heterozygosity and regions of the GRCh38 genome build that have patch scaffolds or alternative loci. Over our entire dataset, this conservative filtering results in 45,819,591 SNPs. Per-SNP F_{ST} was then calculated for all autosomal SNPs with the Weir and Cockerham method in VCFTOOLS.[59,67] We calculated pairwise F_{ST} for all populations vs. the African Yoruba to capture allelic differences (1) of all populations against the same population and (2) between African and non-African populations. The analysis with F_{ST} thus most explicitly focuses on adaptation following the out-of-Africa migration,[68] but we note that elevated F_{ST} shared over many non-African populations can also indicate adaptation in the Yoruba population. To recognize that diverse environments[69–74] may also drive adaptations within Africa, we also calculated F_{ST} for all African population pairs, which are presented in Figure S32.

To leverage as much information as possible, we used all 929 individuals in the HGDP dataset[65] to infer the genealogical trees with Relate.[56] We filtered the VCF dataset to retain only biallelic sites and those regions retained in the accessibility mask.[65] We also removed SNPs with more than 10% missing data, as recommended for phasing.[75] Phasing was performed with SHAPEIT 2 (0.3-Mb window size and 200 conditioning states[75]), resulting in 47,299,072 SNPs that were used for tree inference. Before running Relate, we followed the advised pre-processing step[56] that determines the ancestral state of variants (using the ancestral human genome from Ensembl[76]), adjusts the distances between SNPs (necessary when using a mask to remove regions), and generates an additional annotation file (detailing the upstream and downstream alleles and the number of carriers of the derived allele in each population). Genealogical trees were then inferred along the genome with Relate. We then restricted our analysis to the 913 individuals representing our 40 populations. For each of the 40 populations in our dataset, we extracted and re-inferred their genealogical trees, simultaneously estimating population size changes, branch lengths, and average mutation rate. We used the DetectSelection module in Relate to calculate the probability under neutrality of each autosomal SNP reaching its observed frequency today, given its inferred genealogical history. This latter step was also done for each of the 40 populations individually.

Signatures of positive selection in MA genes

F_{ST} values and Relate probabilities were extracted for all SNPs spanning 10 kbp up- and downstream of each MA-gene coordinates. For each population (or population pair), SNPs that fall in the tails of the F_{ST} and Relate empirical distributions (built from all SNPs along the genome) were identified as those with signatures of positive selection. This empirical approach frees us from requiring inferred demographic models that do not exist

for these populations, and the SNPs identified will be enriched for true targets of positive selection—even if not every SNP is a true target. Further, while the raw output of Relate can be used to identify candidate SNPs, using the tails of the empirical distribution increases accuracy in populations of smaller sample sizes (similar in size to those used here; Figure S10). For this case, the tail of the empirical distribution of Relate identifies SNPs that have an unusually fast spread compared to all other SNPs within this population's inferred history.

When considering signatures at the SNP level, we label the SNPs in the 0.1% tail of either the F_{ST} or Relate empirical distribution as having individual signatures of positive selection. We refer to these as candidate SNPs. When considering signatures across entire MA-gene sets, we label the SNPs in the 5% tail of the empirical distribution as significant SNPs, potentially contributing to polygenic adaptation in that gene set.

For all MA-gene SNPs, the empirical p values of F_{ST} and Relate have a weak correlation (r varies between 0.02 and 0.032) that is nevertheless significant ($p$value<2 − 16), likely due to the very large number of datapoints (Table S14). When only considering significant SNPs identified by F_{ST} and with a lower frequency in the Yoruba population, this weak, positive correlation becomes insignificant in the African Mandenka and Ju|'hoan populations (Table S15). These two methods thus identify slightly related, but not fully overlapping, sets of significant and candidate SNPs.

We first evaluated whether there is an excess of SNPs with signatures of positive selection across each micronutrient by comparing, using a chi-squared test, the observed number of candidate and significant SNPs across entire MA-gene sets to the expected number of SNPs above each significance threshold (5% or 0.1% of the total number of SNPs, respectively). This was repeated for each MA-gene set separately, testing for an enrichment of SNPs at the 5% significance level only.

To explicitly investigate the signatures of polygenic adaptation in individual micronutrients, we used the gene set enrichment method SUMSTAT.[60,77] For each population and MA-gene set combination, and separately for F_{ST} and Relate, the $p$value of the top-ranking SNP of each gene in the set (as calculated from the empirical background distribution) was extracted. In the case of SUMSTAT values calculated from F_{ST}, we only consider F_{ST} calculated between Yoruba and each test population. These $p$values were summed across each MA-gene set to generate a summed MA-gene set value, or SUMSTAT value, for each micronutrient. The SUMSTAT values for each micronutrient were then compared to summed values calculated for each population from 1,000 background-gene sets (see section "micronutrient-associated gene sets"). Micronutrients with SUMSTAT values that fall in the 5% tail of this background distribution were identified as candidates for polygenic adaptation.

We isolate the MA genes with the most extreme evidence of positive selection (passing our stringent Bonferroni threshold; $p \leq 4.65e^{-6}$) as candidates for driving monogenic adaptation (see section "positive selection on individual MA genes"). To explore whether top-ranking SNPs may be adaptively introgressed from archaic humans, we determined which SNPs fall in regions previously inferred as introgressed from Neanderthal and Denisovan (see Note S4[78,79]).

Inferring time of positive selection

We use CLUES2[80] to estimate the strength and likelihood of selection from generations 500, 1,000, 1,500, and 2,000 (corresponding to time points beginning at 14, 28, 42, and 56 kya; Data S4). This method leverages inferred local trees to jointly estimate the timing and strength of selection, using a hidden Markov model that treats inferred local trees as the observed state and the allele-frequency trajectory as the hidden state.[80,81] Signatures of positive selection were identified in SNPs with $p \leq 0.001$; a cutoff of $p \leq 1e^{-10}$ was used to identify the SNPs with the strongest evidence of positive selection (Figures S22–S30).

Building haplotype networks

Haplotype networks were built for 10-kbp regions surrounding candidate SNPs (see Table S8) in three zinc-associated genes with geographically widespread signatures of positive selection (identified in more than 10 populations) at the more stringent threshold of $p < 0.0001$. The candidate SNPs chosen for this analysis were those with signatures of positive selection identified in the highest number of populations (Table S8). These regions were extracted from the phased data (see section "identifying the genomic signatures of positive selection") and used to build a median joining tree network in POPART.[82]

Results

Simulating positive selection in human populations and power results

We first evaluate the power of different methods to identify diverse signatures of positive selection (Figure 1). Many current methods to identify the genomic signatures of positive selection have good power for strong selection on *de novo* mutations[53,83–86] but have considerably lower power when selection is weak, on standing variation or polygenic. Identifying the subtler signatures of positive selection is important, however, given that selection on standing variation likely plays a significant role in local adaptation in modern humans[83,87–89] and that many complex traits, such as micronutrient uptake or metabolism, are polygenic in nature.[15,60,90–92]

For this reason, we designed simulations to test the power of commonly used methods to identify the signatures of positive selection on standing genetic variation at both the monogenic and polygenic level at four time points (1, 5, 10, and 40 kya) in four simulated populations (African, European, East Asian, and American) and two strengths of selection (0.001 and 0.005) (Figure 1, section "material and methods"; Note S1), as well as neutral simulations under the same demographic model. To assess power, we simulated the process of identifying SNPs with signatures of positive selection by identifying selected SNPs that fall in the extreme 5% tail of the neutral distribution (Note S2). This allows us to calculate the TPR (the percentage of selected SNPs with evidence of positive selection).

We evaluated methods that identify candidate SNPs as those with unusual allele-frequency differentiation between populations (F_{ST}[59]), extended haplotype homozygosity (iHS, nSL, XPEHH, and XPnSL[53–55,58]), and unusual local inferred genealogies, such as those with short terminal tips (SDS[57]) or indicative of unusually rapid allele-frequency increase (Relate[56]) (Note S2).

For all methods, and as expected, the TPR is highest for the oldest simulated selection (positive selection initiated 40 kya; Figures S2 and S3). The haplotype-based methods and SDS have relatively low power in our simulations at all time points (TPR $\leq$ 19; see Note S2; Figures S2 and S3), likely because we model selection on standing variation. In contrast, the allele-differentiation method (F_{ST}[59]) and the tree-recording method (Relate[56]) have appreciable power when selection starts 40 kya (TPR $\geq$ 51.6% and $\geq$ 27.4% for F_{ST} and Relate, respectively; Figures 1B, S2, and S3), and they retain power higher than or equal to the other methods for younger selection (Figure 1C). Also as expected, TRP improves with stronger selection: TPR is as high as 69.6% and 86.9% for Relate and F_{ST}, respectively, when selection in the simulated African population has selection coefficients between 0.04% and 0.05% (Figure 1C; all other simulated populations and time points shown in Figures S4 and S5).

Finally, we simulated polygenic selection by grouping simulated gene regions into gene sets of variable size (Figures 1A and 1E) and using the gene-set method SUMSTAT,[60,77] which sums the strongest evidence of positive selection for each gene within a gene set and compares this sum to that of a comparable neutral distribution (see section "material and methods"). We run SUMSTAT with the two best-powered methods: F_{ST} and Relate. SUMSTAT performs notably better when based on F_{ST} than Relate for positive selection initiated at 40 kya (Figures 1D, S12, and S13), but both perform well when gene sets are large and all genes in the set are selected (where F_{ST} and Relate have TPR of $\geq$ 99.9% and $\geq$ 76.8%, respectively, for gene sets of size 40 or larger). Power drops for gene sets that are smaller or contain neutral genes (Figure 1D) and, naturally, for more recent selection (e.g., TPR $\leq$ 53.9% for large gene sets containing only selected genes with selection at 10 kya or earlier; Figures S12 and S13). Still, power remains appreciable even when many genes in a set evolve neutrally; for example, for selection starting 40 kya, F_{ST} maintains a TPR of $\geq$ 84.9 when gene sets contain 40 genes or more and only 60% of genes are under selection (Figure 1D).

While these power estimates are specific to our particular model and should not be considered universally accurate, they allow us to select the best methods for this study. Following this analysis, we selected Relate[56] and F_{ST}[59] to identify signatures of positive selection in MA genes in genomic data. These methods have the highest power to identify selection on standing variation and are, in our view, most suited to identifying the varied signatures that accompany positive selection. The combination of these two methods can also be informative; Relate calculates the probability of selection since the onset of mutation, which typically predates the split of populations, and as a consequence is more likely to detect selection on *de novo* mutations that are typically old enough to be shared among populations. In contrast to Relate, F_{ST} identifies only local adaptation post-dating the split of the population pair and has equal power for selection on standing variation. Using both methods not only maximizes power to identify local adaptation but can also allow us to make a finer-scale inference of the evolutionary history of the alleles.[89,90]

Gene and population datasets

We investigate the evolutionary history of gene sets associated with 13 micronutrients, selected based on their importance in public health and our knowledge of the genetic basis of their biology[15,20–26,63,93–104]: calcium, chloride, copper, iodine, iron, magnesium, manganese, molybdenum, phosphorus, potassium, selenium, sodium, and zinc. This includes all trace metals and macrominerals (Table S1), with the exception of fluoride and sulfur, which were omitted due to limited knowledge of their functionally associated genes in humans. For similar reasons, this study does not investigate the role of the 13 essential vitamins in human adaptation.

We manually curated gene sets associated with the uptake, regulation, and metabolism of these micronutrients (Table S1, section "material and methods"). After filtering for genome accessibility[65] ("material and methods"), a total of 269 MA genes (henceforth referred to as MA genes; Data S1) are included across the 13 MA-gene sets. Random sets of gene-containing genomic regions approximately matched in length and SNP density to the genes in each MA-gene set, and therefore representative of comparable genomic regions, were generated to serve as proxies of random genomic backgrounds. These are henceforth referred to as background-gene sets.

Patterns of genetic variation in MA genes were analyzed in the global HGDP dataset,[65] which contains 929 individuals sequenced to an average 35$\times$ coverage from geographically and culturally diverse populations over seven major continental regions (Africa, Middle East, Europe, Central-South Asia, East Asia, the Americas, and Oceania). Of the populations with the smallest sample size, two were discarded and 22 were merged with populations of very high genetic similarity, generating a final dataset of 913 individuals in 40 populations, with an average sample size of 23 individuals each (see section "material and methods"; Note S3; Figures S14–S20). We note that merging populations may reduce sensitivity but will not generate false positives in signatures of local adaptation.

Our 269 MA genes contain 477,029 SNPs (MA-SNPs) across the 40 populations. The distribution of allele frequencies of SNPs across each MA-gene set in Yoruba (which we use as our background population) fits expectations based on chromosome 1 for all micronutrients except for molybdenum (Table S2). The molybdenum-associated gene set is the smallest MA-gene set in our study ($n = 5$) with its SNPs displaying unusually high allele frequencies (mean allele-frequency difference between these SNPs and chromosome 1 = 0.082; $p = 2.2 \times 10^{-16}$), largely due to the SNPs in the *GPHN* (MIM: 603930) and *MOCS2* (MIM: 603708), indicating possible

selection in these genes in the Yoruba population and otherwise; see section "positive selection in individual micronutrients"). Still, higher background allele frequencies can bias inferences of local adaptation, so we interpret signatures of positive selection in the molybdenum-associated genes with extreme caution—while confirming that this is not an issue for any of the other MA sets.

MA positive selection

To investigate whether positive selection has shaped the evolution of MA genes in humans, we identify signatures of positive selection in each MA-gene SNP in each population using the tails of the empirical distribution of Relate[56] and F_{ST}[59] (see section "material and methods"). We calculate Relate probabilities for positive selection across the entire genome for each of the 40 populations in our dataset and calculate F_{ST} values per SNP between each worldwide population and Yoruba as well as between all population pairs within Africa.[56,59] SNPs in the extreme 0.1% tail of the empirical distribution (empirical p value <0.1%) of each population (Relate) or population pair (F_{ST}) are considered candidate SNPs and their respective MA genes as candidate MA genes, while those in the 5% empirical tails, the significant SNPs, are considered only for polygenic selection.

First, we consider the evidence that micronutrients, as a group, can be considered an important selective driver in modern humans. For each population, we assess whether MA genes are enriched with signatures of positive selection by evaluating whether the number of SNPs in the 5% and 0.1% tail (significant and candidate SNPs, respectively) across all MA genes is significantly higher than neutral expectations (the same percentage of all SNPs across all MA genes).

When considering the number of significant SNPs identified by F_{ST}, the majority of populations (25 out of 40) have significantly more SNPs compared to neutral expectations, with an average enrichment of 8.5% and up to 14.8% more SNPs than expected by chance (chi-squared p values <0.05; Data S2). When only considering candidate SNPs (empirical p value <0.1%), 21 populations show an excess of SNPs in MA genes compared to neutral expectations (with an average enrichment of 56.6% and up to 106% more SNPs than expected by chance; chi-squared; all p values <0.05; Data S2). This unusually high genetic differentiation of SNPs in these genes suggests that, as a group, micronutrients may have been an important selective force driving genetic adaptation in modern humans.

Intriguingly, the picture is slightly different for Relate, with no populations showing more significant SNPs than expected under neutrality and only nine populations showing more candidate SNPs than expected under neutrality (although with an average enrichment of 45.7% and up to 82.9% more SNPs than expected by chance; chi-squared; p value <0.05; Data S2). As mentioned above, differences between F_{ST} and Relate may drive this discrepancy and point to the nature of the selective events. While the results of Relate show very strong evidence of positive selection in a small number of genes, the many more SNPs with F_{ST} signatures suggest micronutrient-related genetic adaptation may largely be local and/or on previously neutral standing variation.

Positive selection in individual micronutrients

To establish whether the patterns above are due to widespread adaptation across all micronutrients or driven by only a few micronutrients, we repeat the analysis for each individual MA-gene set (Figure 2). As expected, for the majority of populations, most micronutrients show no excess of significant SNPs in their respective gene sets, indicating that most micronutrients have not driven widespread, global adaptation. On the contrary, and in line with expectations of long-term purifying selection, 137 of the 507 possible micronutrient and population combinations have a statistically significant deficit of significant SNPs (those in the 5% empirical tail) in their respective MA-gene set according to both Relate and F_{ST}. Nevertheless, all micronutrients show an excess of SNPs in their respective gene set in at least one population, and sometimes in many (Figure 2). Positive selection may therefore have been driven by several different micronutrients, albeit at local geographic scales and specific time points.

Of the 507 possible micronutrient and population combinations, 45 have a statistically significant excess of significant SNPs (those in the 5% empirical tail) in their respective MA-gene set according to both Relate and F_{ST} (chi-squared; p value <0.05; Data S2). These represent the most reliable excess of significant SNPs, strongly suggesting the presence of selective pressures associated with the relevant micronutrient in the given population. Notable examples include the excess of significant SNPs identified in genes associated with selenium in three East Asian populations, in agreement the proposed link between low dietary selenium across East Asia[15] and evidence of polygenic or oligogenic adaptation (where oligogenic selection is selection that acts on multiple genes but fewer than considered in classic models of polygenic adaptation). This includes[15] the Yakut, Xibo-Mongolian, and Japanese, which show an excess of 17%, 22%, and 8.6% of significant SNPs when compared with random expectations (see section "materials and methods") with Relate, and an excess of 40%, 38% and 20% with F_{ST} (Data S2). Further, with F_{ST} all East Asian populations have a significant excess of significant SNPs of at least 18%. However, the most striking example is the set of iodine-associated genes of the American Maya, which shows the highest significant excess of significant SNPs over both methods: an excess of 79% and 50% with Relate and F_{ST}, respectively.

The genes associated with molybdenum show an excess of Relate and F_{ST} significant SNPs in many

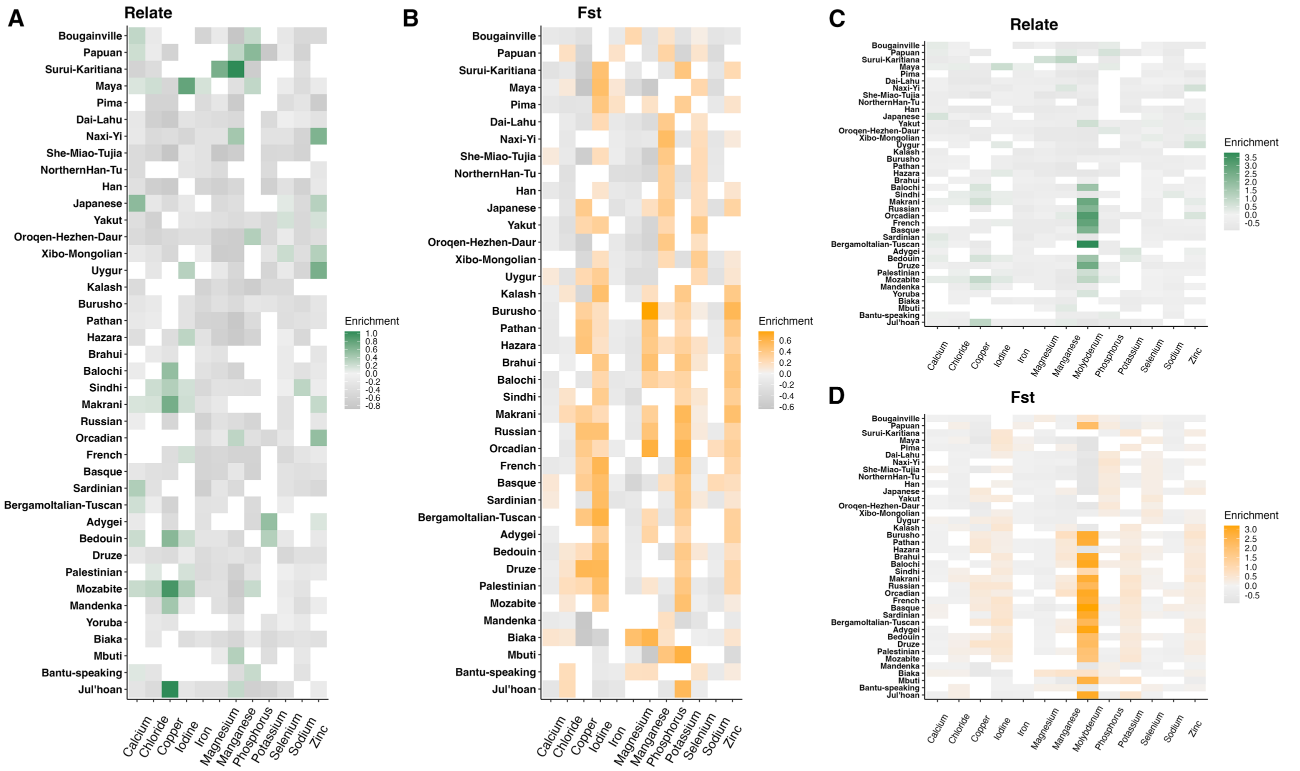

Figure 2. Populations showing a significant excess or deficit of significant SNPs for sets of genes associated with each micronutrient

The excess of significant SNPs for Relate and F_{ST} in each population and micronutrient gene set are shown. Significance is calculated by a chi − squared test (comparing the number of SNPs observed in the 5% tail to the expected 5% of total SNPs); gray shows a significant deficit (fewer SNPs in the tail than expected), and green and orange show a significant excess (more SNPs in the tail than expected, for Relate and F_{ST}, respectively). (A and B) All MA-gene sets excluding molybdenum for Relate and F_{ST}, respectively; (C and D) including the molybdenum gene set for Relate and F_{ST}, respectively.

populations (Figure 2), but this appears to be driven by the high number of significant SNPs in two genes, *GPHN* and *MOCS2*, in this very small gene set ($n = 5$). The SNPs in these two genes have high allele frequencies in Yoruba (see section "gene and population datasets"), where elevated F_{ST} may represent selection in this West African population. Still, there is an excess of signatures of positive selection inferred by Relate in the molybdenum dataset in many non-African populations, making this a complex but intriguing result worthy of follow-up work.

Given that many MA-gene sets are enriched in significant SNPs according to both Relate and F_{ST} (Figure 2), positive selection signatures appear to be present in several SNPs in the same gene set. This is consistent with oligogenic adaptation (signatures in some genes in each gene set, perhaps boosted by linkage disequilibrium in individual genes under selection) but can also be due to highly polygenic adaptation (signatures in many genes in each gene set) or strong monogenic adaptation (selective sweeps with strong linkage disequilibrium in individual genes), or every scenario in between. To understand the role of adaptation to micronutrients in shaping human evolution and differentiation, we must turn to determining which model best explains the observed signatures of positive selection.

Polygenic positive selection

Polygenic positive selection is perhaps the most likely possible mechanism of MA adaptation given (1) the large number of genes associated with micronutrient uptake, metabolism, and regulation[15,22,23]; and (2) the importance of polygenic adaptation in mediating complex trait adaptation.[84,91,105] We therefore test the presence of signatures of polygenic adaptation using the gene set method SUMSTAT[60,77] for each micronutrient and population combination (see section "material and methods"). In brief, this method tests for evidence of polygenic adaptation by combining the strongest evidence of positive selection for each gene within a functional gene set and comparing it with random expectations. We classify micronutrients as having evidence of polygenic selection if the SUMSTAT value falls in the 5% tail of an empirical distribution built from background-gene sets that represent the random genomic background (see section "material and methods").

All micronutrients, with the exception of molybdenum, have SUMSTAT values within the 5% tail of the

background distribution (built from either Relate or F_{ST} summed values; Table S3) in one or more populations. Of these, seven micronutrients (phosphorus, sodium, potassium, iodine, calcium, zinc, and selenium) have SUMSTAT values in the 1% tail of the background distribution in one or more populations (see Table 1). The SUMSTAT values of potassium and phosphorus remain significant even up to the 0.01% tail (Table 1). Nevertheless, none of them reach the stringent Bonferroni correction threshold $p \leq 9.62e^{-5}$.

If we remove overlapping genes (so that no genes are present in more than one MA-gene set; see Table S4 for details), only the SUMSTAT values of phosphorus, selenium, and iron fall in the 5% tail of the neutral distribution of either Relate or F_{ST} SUMSTAT values in one or more populations. Of these, only the SUMSTAT values of phosphorus in the American Pima population and selenium in the East Asian Xibo-Mongolian population fall in the 1% tail of the background distribution (Table 1). The limited signatures of polygenic selection inferred from SUMSTAT are thus largely driven by a small number of genes that are functionally associated with multiple micronutrients (Table 1). Perhaps unsurprisingly, likely targets of positive selection include genes relevant to the biology of multiple micronutrients.

Thus, there is limited evidence of highly polygenic adaptation. In agreement with this, for any individual population, candidate SNPs are found in less than 30% of all genes associated with any micronutrient for Relate and 50% or less for F_{ST} (Figure S21; Data S3). Still, all MA-gene sets, with the exclusion of those associated with potassium and molybdenum, contain candidate SNPs in three or more genes identified by either Relate or F_{ST}. Six MA-gene sets (those associated with calcium, chloride, iodine, iron, selenium, and zinc) contain candidate SNPs in five or more genes identified by Relate or F_{ST}. In these gene sets, candidate SNPs are identified in as many as seven or 11 genes (as inferred by Relate or F_{ST}, respectively). As such, it does not appear that the observed signatures of positive selection are solely driven by singular genes with strong linkage disequilibrium (LD). Instead, we propose that positive selection acting on multiple genes is common across micronutrient categories, compatible with the oligogenic model of local adaptation.

Positive selection on individual MA genes

Although MA adaptation at the gene set level may be largely mediated by oligogenic adaptation, some adaptations may still be monogenic in nature, as in other cases of dietary adaptation.[7,9,12,106] To explore such cases, we isolate the MA genes with extreme individual evidence of positive selection as inferred by Relate or F_{ST} ($p \leq 4.65e^{-6}$, falling below the threshold for Bonferroni multiple-testing correction; Table 2) and present these 15 MA genes as "extreme candidates" for mediating individual micronutrient adaptation. We do not consider genetic adaptation in these genes exclusive to the populations listed in Table 2 (many of these genes are candidate genes in numerous other populations; see "a global view of MA adaptation"), but they remain the strongest candidate populations.

Of the 15 extreme candidate MA genes, 10 genes have extreme candidate SNPs in up to three populations, and two genes have extreme candidate SNPs in seven or more populations across the Middle East, Europe, and Central-South Asia (the phosphorus-associated *PDE7B* [MIM: 604645] and the sodium, chloride, and potassium-associated *SLC12A1* [MIM: 600839]; Table 2). In these latter cases, signatures of selection are therefore both strong and geographically widespread.

Not surprisingly, a good number of extreme candidate MA genes belong to MA-gene sets with limited evidence of adaptation across multiple genes (Data S3). This includes the magnesium-associated genes of *MECOM* (MIM: 165215) and *MLN* (MIM: 158270) in Central-South Asian populations, which are thus likely mediators of monogenic, rather than oligogenic, adaptation. Conversely, other belong to MA-gene sets with evidence of oligogenic adaptation (e.g., the zinc-associated genes in Eurasian populations; see "zinc: candidate for widespread adaptation") and they may not be mediating monogenic adaptation per se, and instead may act as the major drivers of adaptation in that gene set.

Timing of positive selection on iron- and calcium-associated genes

Of the 15 MA genes with extreme candidate SNPs, three are associated with either iron or calcium (Table 2) and could represent rapid micronutrient adaptation with cultural selective drivers. The dietary changes during the Neolithic revolution, such as the transition from nutrient-rich animal products to cereals and staple crops, resulted in drastic reductions of iron and calcium in the diet,[1,3,4] which may have driven rapid and recent genetic adaptation in the affected populations.

To establish whether positive selection on iron- and calcium-associated genes co-occurred with the recent dietary changes of the Neolithic revolution, or if selection was older and more likely driven by the migration into novel soil environments, we use CLUES2 to infer the likelihood of selection at different time points across 19 candidate SNPs of iron- and calcium-associated genes (including those extreme candidate SNPs; see Note S5 and Table S5 for criteria in choosing these SNPs). The inference of CLUES2[80] was run at four informative time points: just after the out-of-Africa migration (2,000 generations; 56 kya), first migrations to Eurasia (1,500 generations; 42 kya), approximate time of major migrations within Eurasia (1,000 generations; 28 kya), and the approximate beginning of the Neolithic transition (500 generations; 14 kya).

CLUES2 identifies signatures of positive selection ($p \leq 0.001$) in three or more populations for each of

Table 1. The SUMSTAT values of all micronutrients and populations within the 1% tail of the empirical distribution of either Relate or F_{ST}

Population	All genes		Non-overlapping genes		Genes removed
	Relate significance	F_{ST} significance	Relate significance	F_{ST} significance	
**Phosphorus**					
Pima	*0.000013*	0.556044	*0.005012*	0.972050	*CASR* (MIM: 601199; calcium)
Mandenka	0.818281	*0.006715*	0.984136	0.068766	
Sodium					
Adygei	*0.000029*	0.028808	0.056801	0.875837	*SLC5A5* (MIM: 601843; iodine); *HSD11B2* (MIM: 614232] potassium); *SLC12A1* (MIM: 600839; potassium, chloride)
Makrani	0.176685	*0.000480*	0.918623	0.312685	
Brahui	*0.001150*	0.026743	0.588617	0.867201	
Bougainville	*0.003455*	0.146640	0.343165	0.949697	
Russian	*0.004935*	0.017819	0.336204	0.809297	
Pathan	*0.004951*	0.228682	0.818310	0.981016	
Ju|'hoan	*0.005700*	0.263232	0.421653	0.989004	
Orcadian	*0.005823*	0.016658	0.512330	0.859558	
French	*0.006133*	0.013281	0.619952	0.806131	
Surui-Karitiana	0.620187	*0.006800*	0.941481	0.766029	
Potassium					
Bantu-speaking	0.222493	*0.000043*	0.999448	0.163764	*SCNN1A* (MIM: 600228; sodium); *SCNN1B* (MIM: 600760; sodium); *SCNN1D* (MIM: 601328; sodium); *SCNN1G* (MIM: 600761; sodium) *PTH* (MIM: 168450; potassium)
French	*0.000322*	*0.008800*	0.993678	0.997444	
Orcadian	0.443830	*0.001556*	1.000000	0.992561	
Surui-Karitiana	0.418921	*0.001698*	0.999134	0.999696	
Russian	0.017106	*0.002343*	0.999301	0.990527	
Bergamo Italian-Italian	*0.002963*	0.029975	0.998941	0.999006	
Bougainville	*0.003722*	0.079932	0.850007	0.999604	
Palestinian	0.033025	*0.005466*	0.994873	0.997447	
Mozabite	0.033461	*0.008791*	0.997262	0.994528	
Kalash	0.038729	*0.009572*	0.988635	0.992443	
Iodine					
Maya	*0.000325*	0.101934	0.352666	0.999365	*DIO1* (MIM: 147892; selenium); *DIO2* (MIM: 601413; selenium); *DIO3* (MIM: 601038; selenium)
Mozabite	*0.006333*	0.717484	0.721672	0.999662	
Russian	*0.009037*	0.438001	0.738631	0.999544	
Calcium					
Mandenka	0.104698	*0.000912*	0.945878	0.289385	*KCNJ10* (MIM: 602208; potassium); *SLC12A1* (MIM: 600839; potassium, chloride); *SLC34A1* (MIM: 182309; phosphorus); *SLC34A3* (MIM: 609826; phosphorus)
Biaka	0.184204	*0.001264*	0.991495	0.406607	
Mozabite	*0.007348*	0.687845	0.613761	0.994571	
Zinc					
Kalash	0.257221	*0.004891*	0.877790	0.361498	*SLC11A1* (MIM: 600266; iron); SLC30A10 (MIM: 611146; manganese, magnesium); *SLC39A14* (MIM: 608736; manganese)
Selenium					
Xibo-Mongolian	0.021710	*0.009930*	0.021710	*0.009930*	–

Clustering of populations by Relate SUMSTAT p values for each full micronutrient gene set are shown in Figures S22–S34. The p values calculated for the equivalent gene sets with no overlapping genes are given to the right. The "Genes Removed" column lists the genes removed from the non-overlapping gene sets (with their MIM numbers and other micronutrient associations given in brackets). Italics indicate values within the 1% tail of the empirical distribution.

Table 2. **Genes, their micronutrient associations, and populations with *p* values below the multiple-testing threshold of 4.65e^{-6}**

Gene	MIM numbers	Micronutrient	Population	Relate significance	F_{ST} significance
ATP2B2	108733	calcium	Mandenka	0.000187	*7.75e−8*
			Sardinian	*2.10e−7*	0.000969
THRB	190160	iodine	Palestinian	*3.23e−6*	0.00159
FTMT	608847	iron	Yakut	*3.37e−6*	0.000827
HIF1A	603348	iron	Basque	*2.43e−6*	0.000249
MECOM	165215	magnesium	Brahui	*1.26e−6*	0.000225
FXYD2	601814	magnesium	Uygur	*2.80e−6*	0.00923
GALNT3	601756	phosphorus	Ju|'hoan	0.0849	*3.5e−6*
PDE7B	604645	phosphorus	Druze	0.000586	*8.16e−7*
			Bergamo Italian-Tuscan	0.000678	*2.92e−6*
			Sardinian	0.00366	*7.02e−7*
			Basque	0.00522	*2.00e−6*
			French	0.000500	*4.28e−6*
			Orcadian	0.00194	*2.24e−6*
			Brahui	0.000618	*1.53e−6*
MLN	158270	phosphorus	She-Miao-Tujia	*4.27e−6*	0.00460
SCNN1D	601328	sodium, potassium	French	*1.87e−6*	0.000684
SLC12A1	600839	sodium, chloride, potassium	Palestinian	0.000137	*6.37e−7*
			Druze	3.61e−05	*2.97e−7*
			Bedouin	0.00571	*1.25e−6*
			Adygei	0.00481	*2.01e−6*
			Bergamo Italian-Tuscan	0.0128	*1.58e−6*
			Basque	0.000855	*2.00e−6*
			French	0.000224	*7.65e−7*
			Russian	0.000519	*1.40e−6*
			Brahui	0.00106	*1.07e−6*
			Balochi	0.0129	*1.76e−6*
			Kalash	0.0504	*2.56e−6*
SLC4A5	606757	sodium	Russian	*3.83e−6*	0.000160
SLC30A9	604604	zinc	Han	0.00228	*3.55e−6*
SLC39A11	616508	zinc	Makrani	*1.40e−6*	0.0003304
SLC39A4	607059	zinc	Makrani	0.0934	*3.95e−6*

Given alongside their associated micronutrient and accompanied by the *p* value calculated by the other method to identify selection. Italics indicate values below the multiple-testing threshold of 4.65e^{-6}.

the 19 candidate SNPs in the calcium- and iron-associated genes (Figures 3 and S22–S30; Data S3). For most calcium- and iron-associated genes, strong evidence for positive selection is identified at time points surrounding major migrations, often in clusters within particular regions in Eurasia (Figure 3), in support of positive selection coinciding with the colonization of novel Eurasian environments. There are a few exceptions where the strongest evidence for positive selection is at the most recent time point in certain populations, but, overall, SNPs in these genes often have very similar evidence for positive selection across different time points (Figures 3 and S22–S30), so we are largely unable to distinguish among the tested time points when positive selection was most likely. Positive selection on calcium- and iron-associated genes is thus unlikely to have been exclusively driven by the recent dietary changes of the Neolithic, although recent positive selection may have shaped the evolution of a small number of genes in particular

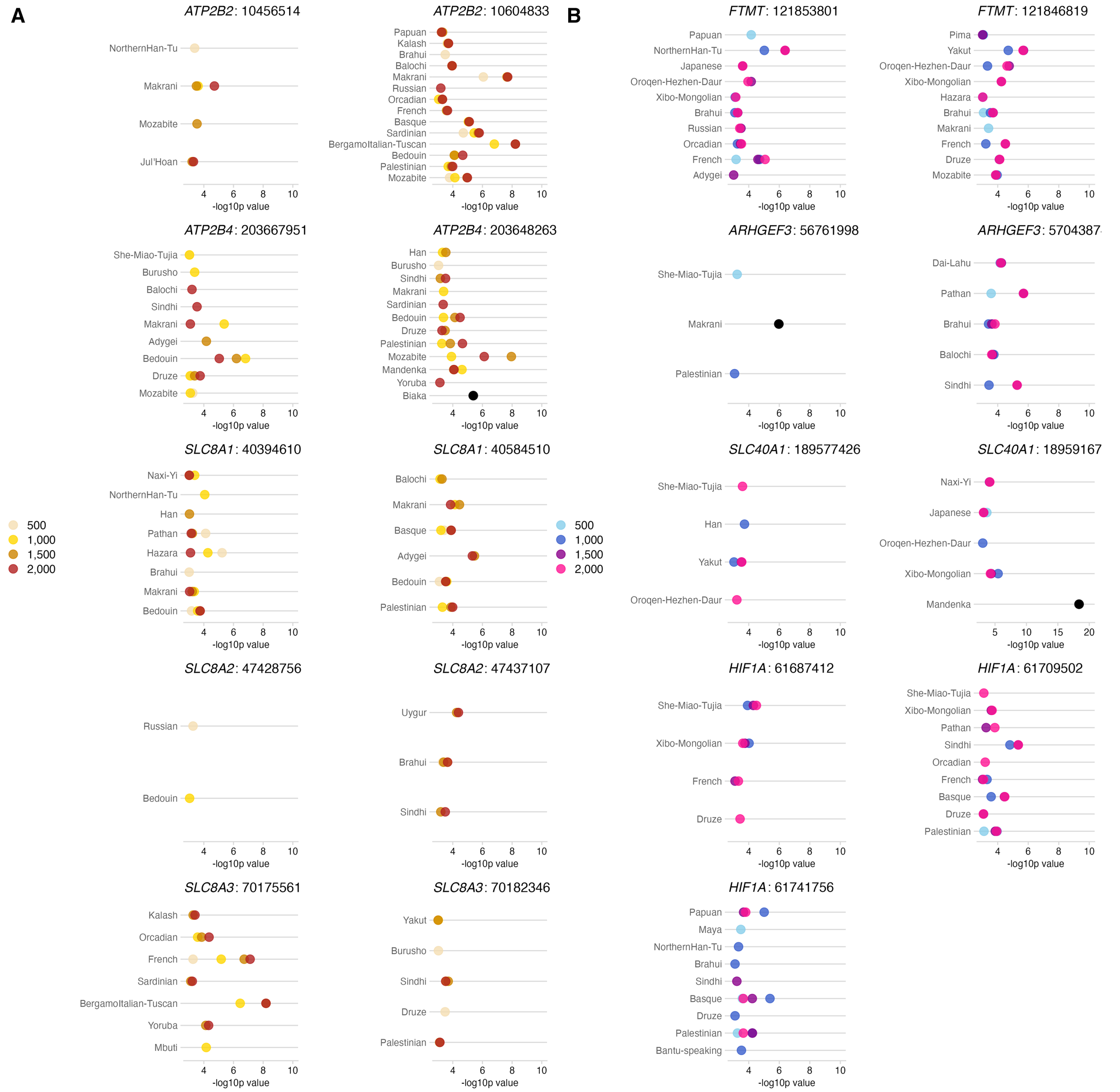

Figure 3. The populations with the strongest evidence for positive selection

The populations with the strongest evidence for positive selection as inferred by CLUES2 ($-\log 10$ p value > 3[80]) for candidate SNPs (where positions are given according to genome build hg38) in (A) calcium-associated genes *ATP2B2*, *ATP2B4*, *SLC8A1*, *SLC8A2*, and *SLC8A3* and (B) iron-associated genes *FTMT*, *ARHGEF3*, *SLC40A1*, and *FTMT*.

Points colored by the generations for which the evidence of positive selection is observed (see legends on the right); black points indicate that the evidence for selection is equal across all four tested time points.

geographic areas (e.g., Central-South Asia for the calcium-associated *SLC8A1* [MIM: 182305]).

A global view of MA adaptation

Stringent significance thresholds are necessary to robustly identify signatures of monogenic and oligogenic positive selection but allow weaker cases of positive selection to remain hidden. As such, they can result in a limited picture of the geographic distribution of putative adaptations.

To provide a more comprehensive picture of MA-mediated genetic adaptation, we summarize the distribution of signatures of positive selection identified by Relate across all MA genes and populations in Figure 4 (see Figure S31 and S32 for signatures of positive selection identified by F_{ST}). This largely recapitulates previous conclusions; there is high heterogeneity in the evidence of positive selection across micronutrients (both at the level of MA genes and entire MA-gene sets), with some having very strong evidence of having evolved under positive selection. There

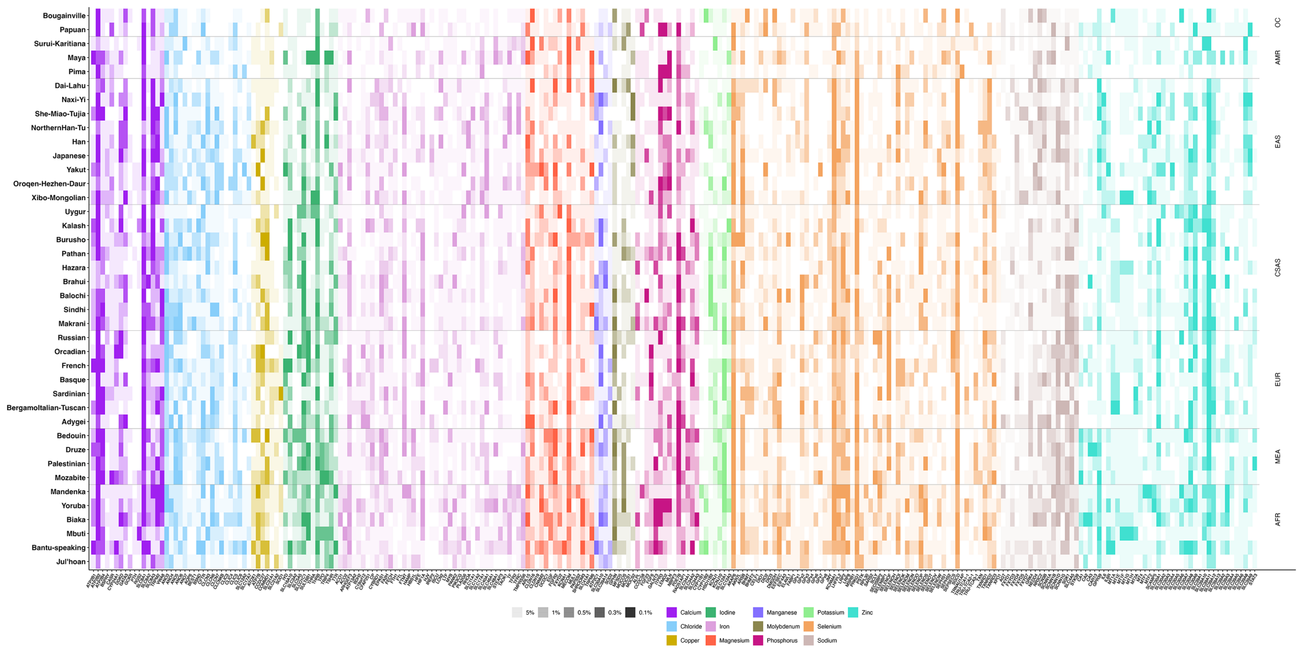

Figure 4. Signatures of positive selection as inferred by Relate across all autosomal MA genes
y axis, colored by micronutrient, and in all populations, *x* axis is grouped by metapopulation. Darker blocks reflect lower empirical *p* values (from lightest to darkest: below 5%, 1%, 0.5%, 0.3%, and 0.1%, see left legend).

is also substantial geographic heterogeneity, with some signatures of positive selection clustering in individual populations, while others are present across continental regions or even most non-African populations. Figure 4 suggests that (1) virtually all micronutrients may have acted as selective drivers in particular human populations; (2) many putative instances of adaptation are likely geographically widespread, to a larger extent than previously appreciated; and, most importantly, (3) MA adaptation has likely had a global impact on the patterns of genetic diversity of our species.

However, if we consider each micronutrient as a selective driver, which populations have the strongest evidence of positive selection? This is a challenging question to answer, especially under an oligogenic model, so we develop criteria that consider the number of candidate SNPs, candidate genes, and strength of signatures of positive selection. Here, we focus on criteria across entire gene sets: while genetic drift in populations with extreme demographic histories (e.g., recent bottlenecks or ancient structure[47,49,65,69,73]) may drive strong allele-frequency change in individual SNPs, such processes are unlikely to result in signatures at the oligogenic level.

While avoiding explicitly comparing populations, we highlight those that, for each micronutrient, have (1) a significant excess of significant SNPs within the associated gene set (Figure 5A, Data S2); (2) a substantial proportion of genes with signatures of positive selection (those with more than 20%; Figure 5B and Data S3) within the associated gene set; or (3) two or more MA genes with the strongest signatures of positive selection (top-ranking MA genes, defined as those MA genes falling within the five strongest signatures of positive selection when considering all MA genes for that population) within the associated gene set (Figure 5C; Tables S6 and S7). These criteria are *ad hoc*, but they allow us to generate a list of populations with the strongest evidence of oligogenic adaptation associated with particular micronutrients (Figure 5D). Additional populations may have weaker evidence of adaptation for that micronutrient (see Figure 4), but these criteria allow us to highlight and prioritize the most interesting signatures of positive selection among all the populations in this study.

With the exception of selenium-associated adaptation in East Asians,[15] zinc-associated adaptation across non-African populations,[18,19] and putative iron-associated adaptation in Europeans,[62] the cases presented in Figure 5D represent previously undescribed cases of MA adaptation. This includes the iodine-associated gene set in the American Maya, which fulfills all three of the selection criteria outlined in Figure 5. Importantly, evidence for oligogenic adaptation is identified in all MA-gene sets (bar molybdenum, our smallest gene set of $n = 5$) and across all major global regions (excluding Oceania, of which only two populations are included; Figure 5D). Both Figures 4 and 5 suggest not only that the diversity and prevalence of MA adaptation among human populations is much higher than previously appreciated but also that it may underlie many cases of human local adaptation.

Zinc: Candidate for widespread adaptation

Among the micronutrients referenced in Figure 5, zinc is particularly interesting because of the geographically

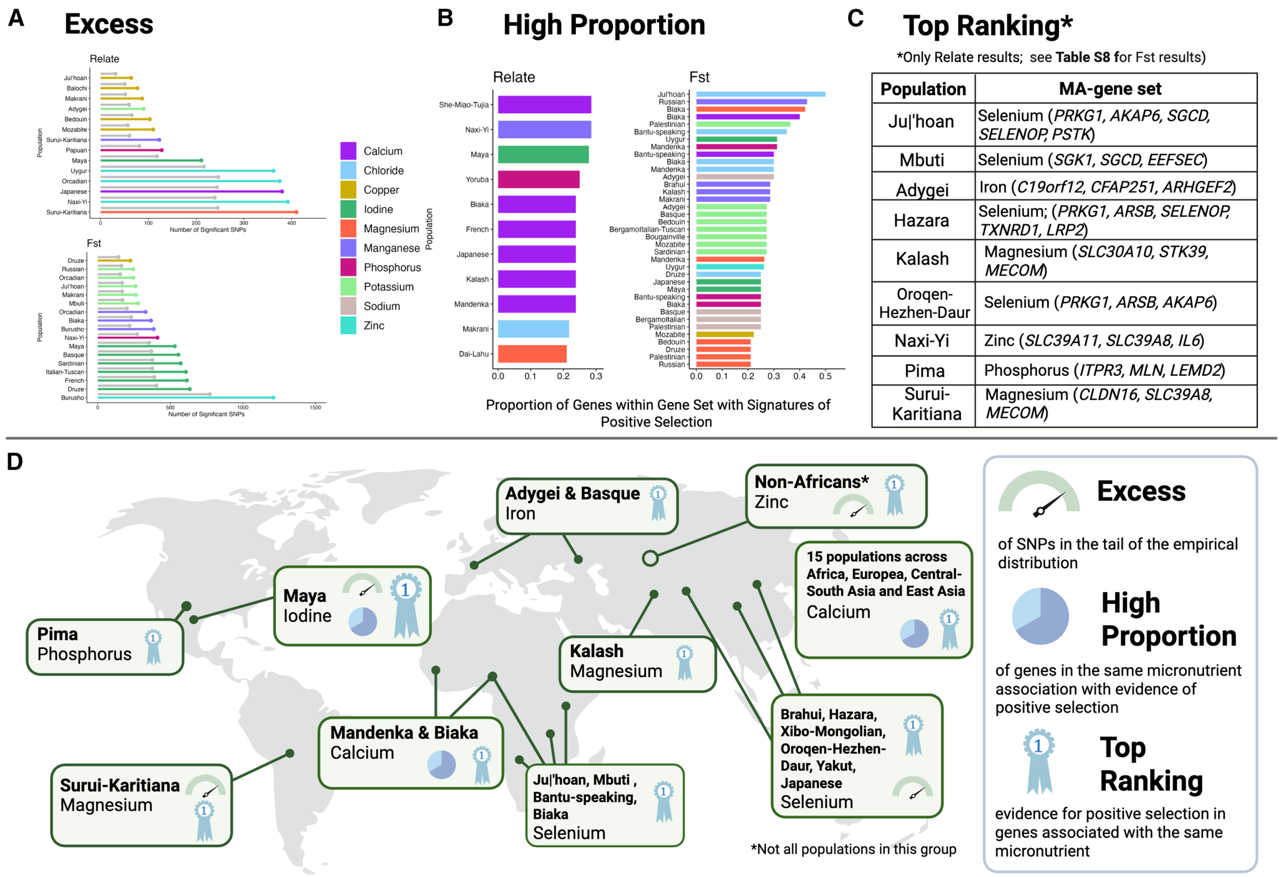

Figure 5. Overview of some of the populations with the strongest evidence for oligogenic adaptation for different micronutrients

(A) Combinations of MA-gene sets and populations with over 50% excess of significant SNPs (5% empirical tail) of Relate (top) or F_{ST} (bottom) when compared to random expectations (5% of all SNPs).
(B) Combinations of MA-gene sets and populations with over 20% of genes showing signatures of positive selection (at least one SNPs with $p < 0.001$) according to either Relate (left) or F_{ST} (right).
(C) Populations where three or more of the top-ranking MA genes are associated with the same micronutrient, according to Relate. Populations with two or more of the top-ranking MA genes associated with the same micronutrient, according to either Relate or F_{ST}, are given in Tables S6 and S7.
(D) Schematic representation of the populations with the strongest evidence for oligogenic selection, with icons reflecting populations with criteria as “Excess” (A), “High Proportion” (B) and “Top-ranking” (C). Created in BioRender. Rees, J. (2025) https://BioRender.com/u42r662

widespread nature of its signatures. Of the 40 populations included in this study, 28 populations have a significant excess of SNPs in the 5% empirical tail of the zinc-associated gene set (Figure 2), and 25 populations have two or more top-ranking MA genes associated with zinc (Figure 5; Tables S6 and S7). Signatures are present both with F_{ST} and Relate (Data S2; Tables S6 and S7), and thus the widespread signatures with F_{ST} are unlikely to reflect adaptation solely within Africa. We hence evaluate whether the geographically widespread signatures are consistent with shared selection on an ancestral non-African population.

Of the 46 zinc-associated genes, 36 (78%) have candidate SNPs in at least one population ($p \leq 0.001$, according to either Relate or F_{ST}), of which 16 (34%) remain at the more stringent threshold of $p < 0.0001$. Seven genes have candidate SNPs in 10 or more populations ($p \leq 0.001$ according to either Relate or F_{ST}), which we consider “geographically widespread” signatures, of which four genes (*SLC39A4* [MIM: 607059], *SLC30A9* and *SLC39A11* [MIM: 616508], and *GPR39* [MIM: 602886]) remain at the more stringent threshold of $p < 0.0001$ (Figures 6A and 6B). The signatures of positive selection in *SLC39A4* are only identified by F_{ST}, and we caution that this may be a West African-specific signature of local adaptation (see Figures 6B, S32, and S33). In contrast, all three of the remaining genes have candidate SNPs identified by Relate in five or more non-African populations and are thus strong candidates to have mediated adaptation in the ancestors of all analyzed non-Africans: *SLC30A9*, *SLC39A11*, and *GPR39*. The strongest candidate SNP is in *SLC39A11* in the Central-South Asian Makrani ($p = 1.40e-6$; see Table 2) and its derived allele is in nearly identical haplotypes in nearly all

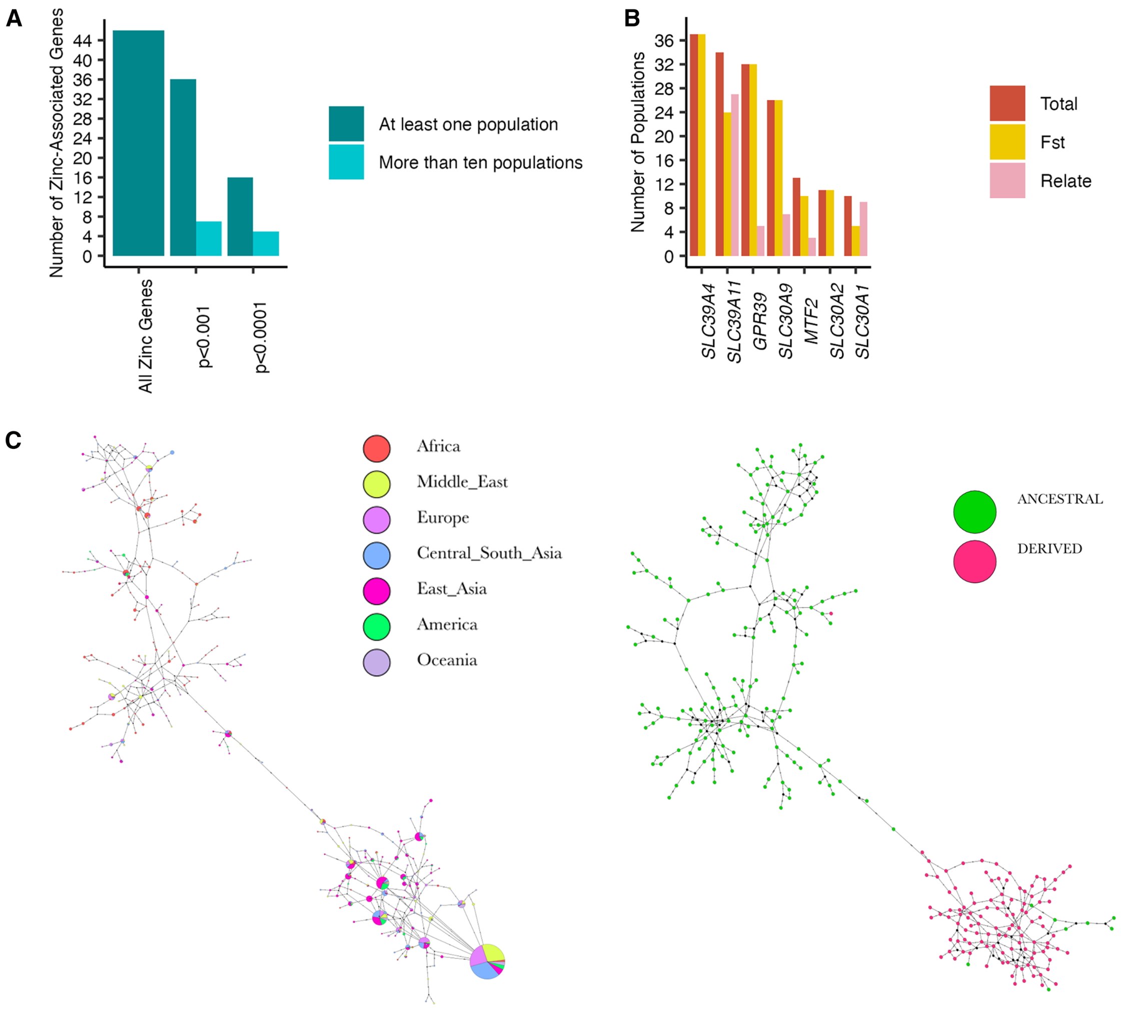

Figure 6. Signatures of positive selection across zinc-associated genes
(A) Number of zinc-associated genes with significant SNPs at the $p < 0.001$ and $p < 0.0001$ thresholds for at least one population (dark blue) or at least 10 populations (light blue).
(B) Number of populations with candidate SNPs identified in the seven genes with geographically widespread signatures ($p < 0.0001$) by Fst and Relate.
(C) Haplotype networks of *SLC39A11* built from the 10-kb locus around the SNP with the strongest evidence of positive selection (in the Central-South Asian Makrani; $p = 1.40e-6$; hg38 position: 73010373) colored by an individual's population (left) and whether the focal allele is ancestral or derived (right).

non-African individuals (some of which are at high frequency within continental groups; Figure 6C; see section "materials and methods"); conversely, the candidate SNPs in *SLC30A9* and *GPR39* are found on multiple, divergent haplotypes in non-African individuals (Table S8; Figures S33–S38). This suggest that positive selection on both new mutations and standing genetic variation may have mediated zinc-associated adaptation in an ancestral non-African population.

Discussion

For the majority of our history as a species, the diet of populations has been dictated by local food availability, soil quality, and cultural practices.[7,9,27,38,107,108] Variation among these factors has driven genetic adaptation among populations across the world,[7,12,15,18,19,106,109–111] with some adaptations as key considerations in modern public healthcare.[5,9,12,15] Here, we go beyond such individual cases to investigate the global impact of micronutrients in human evolution, considering their essentiality in human health and variability across the diets of populations. We identify evidence of positive selection across all MA gene sets, including evidence for one or more gene sets in over half of the tested populations, suggesting that, as a group, micronutrients are a more important selective force in recent human evolution. This global signature is driven by particular MA-gene sets, suggesting that some micronutrients played a particularly important role in human local adaptation. It is these adaptations that have the most important implications when considering the role of evolutionary history in the population-level risk of MA disorders today.[85,112]

Oligogenic local adaptation

A model of oligogenic adaptation is best suited to explain the signatures of adaptation identified in MA gene sets. It remains possible that adaptation was highly polygenic, because, if the signatures of positive selection in individual genes are very weak, the power of even the most powerful methods will remain limited. As shown here, F_{ST} and Relate have higher power than haplotype-based methods to identify weak positive selection and selection on standing variation, but power is still low for recent selection and for the lowest tested selection coefficients. Nevertheless, and in consideration of methodological limitations, our results suggest that only a few genes mediate adaptation for most micronutrients, while purifying selection has likely driven the evolution of the rest of MA genes. Our gene sets include genes involved in uptake, metabolism, and function of the micronutrient, and it is likely that only some of these aspects would evolve upon a particular dietary pressure. A clear example of this is the zinc-associated set, where evidence of positive selection clusters heavily on zinc transporters.

Importantly, we observe evidence of MA adaptation across populations in all major regions of the globe. By extension, human populations may, on average, differ in their micronutrient metabolism, uptake, or regulation, and those with strongest evidence of MA adaptation should be prioritized in future genomic medicine studies. Due to space limitations we are only able to discuss only the most interesting cases: iodine, which has particularly strong evidence of positive selection and potential phenotypic consequence in the American Maya population; selenium, where adaptation coincides with both soil geology and public health across East Asia; and zinc, where adaptation appears to be widely shared outside of Africa and thus contributes to genetic differences between people of African and out-of-African descent.

Iodine

In the Maya population of Central America, the iodine-associated gene set has both a significant excess of signatures of positive selection (Figure 2; Table 1) and a high proportion of iodine-associated genes identified as candidate genes (>20%; Figure 5). High rates of goiter, a swelling of the thyroid gland caused by extreme iodine deficiency, is common among contemporary populations across Central America,[113,114] indicating iodine deficiency as a pervasive environmental pressure in this region (albeit perhaps exacerbated by socioeconomic inequalities). Given that contemporary populations in this locality likely have substantial recent European and African ancestry, this is not an evaluation of the response to iodine deficiency in the Maya, and it remains possible that this population has evolved genetic adaptations to mediate the risks of dietary iodine deficiency.

Interestingly, four iodine-associated candidate genes in the Maya are also candidates in the Mbuti population of Central Africa, three of which are part of the thyroid hormone pathway: *THRA* (MIM: 190120), *THRB* (MIM: 190160), and *TRIP4*[115] (the latter identified as a target of positive selection[16]). Rainforest environments, such as those where the Mbuti live, often have iodine-deficient soils,[35,45,116] and it has been previously suggested that this may drive adaptations in local populations.[16] Thyroid receptors also play an important role in regulating growth, development, and metabolism,[25,117,118] and it is feasible that adaptations in these genes, driven by low dietary iodine, underlie the distinctive short stature of Maya and Mbuti (height < 160 cm[113,119]).

Signatures of positive selection in an additional iodine-dependent thyroid receptor (*IYD*) have previously been identified in the Biaka (and recovered here, with SNPs within the 0.3% and 0.5% empirical tail of Fst and Relate, respectively), another short-statured (height < 160 cm[113]) population living on the iodine-deficient soils of an African rainforest environment.[16] Incidentally, the short-statured Efe population (another rainforest hunter-gatherer population living in similar rainforest environments in Africa, like the Biaka and Mbuti populations) exhibit a much lower rate of goiter than their neighboring, and much taller, Bantu-speaking populations.[45] Our results provide additional support for a link between genetic variation of iodine-associated genes and short stature. While there is still significant debate about the adaptive significance of short stature in rainforest hunter-gatherers (with hypothesized advantages including thermoregulation, mobility, and earlier reproduction[69,113,120,121]),we suggest that, in some populations, short stature may be an evolutionary consequence to adaptations mediating the risks of low dietary iodine.

Selenium

A selenium-deficient soil belt stretches across China,[108,122] generating selenium deficiency in the diets of local populations. In the regions with the most severe selenium deficiency, there are endemic levels of the cardiomyopathy Keshan disease and the bone disease Kashin-Beck.[36,37] We identify evidence of oligogenic adaptation in multiple selenium-associated genes in many East Asian populations (Figures 2 and 4; Table 1), in agreement with previous evidence of polygenic or oligogenic adaptation in selenium metabolism genes in East Asian populations.[15] We also find evidence of selenium-associated oligogenic adaptation in several African populations. While there lacks a comprehensive understanding of geospatial variation in soil levels of selenium across Africa, there is evidence for considerable variation in selenium status within and between African countries,[28,29] which may have driven local adaptation.

Focusing on Asia, where the strongest link between soil and adaptation exists, four selenium-associated genes have frequently co-occurring signatures of positive selection in the same populations: *SGCD* (MIM: 601411), *AKAP6* (MIM: 604691), *PRKG1* (MIM: 176894), and *KCNMA1* (MIM: 600150; Figures 4, S32, and S33; Table S9). While this is simply a qualitative observation, all these genes have intron SNPs associated with selenium

regulation, and epistatic interactions exist between SNPs of *AKAP6* and *SGCD* and between SNPs of *AKAP6* and *KCNMA1*.[123] It is thus likely that mutations in these genes interact to regulate selenium levels and that this is the driver of their evolution in humans. In African populations, the strongest signatures of selection in selenium-associated genes are in *LRP8* (MIM: 602600) and *LHFPL2* (MIM: 609718). Most interestingly, *LRP8*, a receptor of the selenoprotein P (*SELENOP* [MIM: 601484]), determines the hierarchy of selenium supply to various organs under deficiency[124] and increases mRNA concentrations following deficiency induced by *SELENOP* knockout.[125] Given that both African and East Asian populations exhibit evidence of selenium-associated oligogenic selection but the genes with the strongest signatures differ (Table S10), it is possible that such populations have adapted to changes in dietary selenium, possibly its deficiency, through changes in different genes. Still, without a more comprehensive understanding of the levels of selenium in soil across Africa, this remains speculative.

Zinc

For zinc, the evidence of genetic adaptation is both remarkably strong and geographically widespread (Figures 2, 4, and 5; Table 2). Signatures of positive selection have been previously identified in multiple zinc-transporter genes,[18,19,64] with much of the literature highlighting *SLC30A9* and *SLC39A4* as those with the strongest evidence of selection.[126] We provide additional support for these hypotheses here, identifying signatures of positive selection at our most stringent threshold in the Makrani of Central-South Asia (*SLC39A4*) and the Han of East Asia (*SLC30A9*). Further, seven zinc-associated genes (five of them zinc transporters) have strong signatures of positive selection ($p < 0.001$) in at least 10 populations: *SLC30A1* (MIM: 609521), *SLC30A2* (MIM: 609617), *SLC30A9*, *SLC39A4*, *SLC39A11*, *MTF2* ([MIM: 609882), and *GPR39*. However, we note that candidate SNPs of *SLC39A4* are identified only by F_{ST} and may be a West African-specific signal of natural selection. It has been previously proposed that the near fixation of the Val372 allele of *SLC39A4* in West Africa is due to increased pathogen stress driving lower zinc uptake in this region,[64] and it appears to be an interesting exception to the global pattern.

Still, by and large, zinc adaptation appears to be mediated by a network of genes (such as *SLC39A11*, *SLC30A8* [MIM: 611145], *SLC30A10* [MIM: 611146], and *SLC30A1*; Figure 6) across many non-African populations, likely as a result of positive selection on an ancestral population. The haplotypes containing top candidate SNPs of *SLC39A11* provide particularly strong support of this hypothesis; they are identical or near identical in nearly all non-African individuals and support a selection event on an allele segregating at low frequency in an ancestral non-African population. A likely candidate is the ancestral population that migrated out of Africa to the Arabian Peninsula. Given the severely iron- and zinc-deficient soils of the Middle East, especially Iran,[127] and detailed history of zinc-deficiency disorders in this region,[39,128] we propose that significant selective pressure may have driven adaptations to regulate zinc levels in an ancestral population inhabiting these zinc-deficient environments. Zinc-deficient soils exist elsewhere outside of Africa, such as South Asia, where populations exhibiting the strongest signatures of positive selection on zinc-transporter genes live; e.g., the Makrani in modern-day Pakistan, where zinc deficiency is prevalent (22.1%) and up to 96.1% of grain samples are zinc-deficient.[129,130] We consider it unlikely that selection events were entirely independent from those observed elsewhere, and instead suggest that either (1) additional adaptations in zinc-transporter genes may subsequently have occurred in populations living on similarly zinc-deficient soil, or (2) differences in power across populations of our tests may result in missed signatures in some populations.

Monogenic local adaptation

We identify 15 MA genes with evidence of having undergone particularly rapid increases in allele frequency, consistent with the action of positive selection. This adds to other cases of adaptation to diet previously reported in humans.[7,9,11,12] These genes represent the strongest candidates of monogenic adaptation in our study, but other candidates for monogenic adaptation may be identified at less stringent thresholds or in currently unsampled populations.

The functions of these 15 genes are diverse and not always exclusively related to their assigned micronutrient.[23,24] Nevertheless, two magnesium-associated genes have strong signatures of positive selection coinciding with soil geology and biological function. The top candidate SNPs in *FXYD2* (MIM: 601814) and *MECOM* are both identified in Central-South Asian populations (Uygur and Brahui, respectively). Adaptation in these genes may not be exclusive to these populations, but soils across Central-South Asian have particularly high levels of magnesium[131,132] and both genes have been linked to hypomagnesemia (HOMG2 [MIM:154020]), either directly (*FXYD2*[132]) or indirectly (*MECOM*, associated with osteoporotic fractures that can be caused by hypomagnesemia[133,134]). We speculate that selection in these populations might reduce magnesium intake, which would represent an entirely undescribed case of adaptation to mediate micronutrient toxicity. Interestingly, top-ranking SNPs in *MECOM* fall within a region of the genome likely introgressed from an archaic human closely related to the Vindija Neanderthal[79,135] (see Note S4). This is thus another example of potential adaptive archaic introgression mediating adaptation to local environment.[10,126,135–137]

Genes associated with nine other micronutrients also have strong signatures of monogenic positive selection. This includes the calcium-associated gene *ATP2B2* (MIM: 108733) and the iron-associated genes *FTMT*

(MIM: 608847) and *HIF1A* (MIM: 603348), which might have mediated adaptation to drastic dietary changes surrounding the Neolithic transition (Figure 3). However, the evidence of positive selection in other iron- and calcium-associated genes (with the exception of calcium-associated *ATP2B4*) does not appear to be strongest at similarly recent time points (Figure 3), and, instead, it appears that the onset of positive selection may largely coincide with the colonization of Eurasian environments, supporting the role of geology in driving MA adaptation.

The way ahead

Here, we show that, beyond the few previously known cases, micronutrients have likely had a significant role in shaping the evolution of genetic variation in humans. For some micronutrients, there is strong evidence that genetic adaptations may have resulted in population-level differences in their metabolism, uptake, or regulation. Given that dietary levels of micronutrients outside a very narrow range has a range of important health consequences,[20,26,34,36,37,116] these adaptations have the potential to cause or exacerbate health disparities across populations. Indeed, micronutrient deficiencies are already considered a global health issue, affecting an estimated 2 billion people worldwide, with the majority of these individuals in sub-Saharan Africa and South-Central Asia.[20,21] We see it as a matter of global health to understand how varying micronutrient levels, especially common deficiencies, may affect people of different genetic backgrounds.

Further, global soil micronutrient levels are rapidly changing as a result of climate change, rising CO_2 levels, and over-farming.[13,27,138] This, alongside increased migration and mobility of global populations, means that many populations will likely encounter micronutrient levels for which they lack adaptations or even have adaptations to regulate in the opposite and now deleterious direction (the "evolutionary mismatch" scenario[112]). For many micronutrients and populations, this study represents a first step in understanding the relationship between genetic variation and MA pressures (such as dietary micronutrient levels), but additional work, including individuals of genetic ancestry groups not represented by populations used here, is required to address this accelerating issue and health connotations in contemporary populations.

First, comprehensive characterization of soil environments across the globe may strengthen or dispute associations between genetic signatures and proposed environmental drivers. High-resolution environmental data are needed for phylogenetic regression models,[139–142] such as phylogenetic generalized least-squares analysis,[142] that can further aid the identification of targets of positive selection driven by soil geology. Currently available variables (e.g., geographic coordinates) are not informative enough of soil geology, which can show large differences even among neighboring locations.[14,27–29,38,107,122,132,134] Generating maps of soil micronutrient levels to co-analyze with genomes remains a promising avenue of future work. Inferences of ancestral soil environments should also be considered, given that over-farming depletes soils of their natural nutrients[27] and may lead to false associations. This is especially true for phosphorus and chloride-associated adaptation; recent agricultural practices heavily shape phosphorus and salinity levels of soils,[13,143] and contemporary soil environments are therefore relatively uninformative in identifying ancestral selective pressures.

Second, functional analysis of candidate genes will more clearly elucidate their role, if any, in their associated micronutrient uptake, regulation, or metabolism. This may inform on the adaptive direction of MA genes (i.e., if adaptive variants have likely responded to deficient or toxic levels of micronutrients). Further studies should be done to identify candidate genes in micronutrients unexplored in this study; vitamin deficiency is also an important health concern,[20,32,144] and there remains a gap in our understanding of genetic adaptation to mediate vitamin uptake or regulation.

Finally, analysis of large biobank datasets and public-health data may reveal relative differences in risk of MA pathologies according to genetic variation, linking local adaptation in response to dietary micronutrients to contemporary health consequences. We highlight the importance of the latter; subtly different genetically encoded responses to dietary micronutrients may contribute to already-existing health inequalities between populations, which may be exacerbated by present and future decreases in soil or diet quality.

Data and code availability

- All data generated for this article are available in the supplemental information or from the corresponding author on request.
- Scripts are available at https://github.com/jas-rees/micronutrients-2025/.

Acknowledgments

J.R. and S.C. are funded by NIHR GOSH BRC. The views expressed are those of the authors and do not necessarily reflect those of the funding body, including those of the NHS, the NIHR, or the Department of Health. A.M.A. is supported by UCL's Wellcome Trust ISSF3 award no. 204841/Z/16/Z.

Declaration of interests

The authors declare no competing interests.

Supplemental information

Supplemental information can be found online at https://doi.org/10.1016/j.ajhg.2025.08.005.

Received: February 20, 2025
Accepted: August 4, 2025
Published: September 10, 2025

References

1. Bocquet-Appel, J.P., and Bar-Yosef, O. (2008). The neolithic demographic transition and its consequences https://doi.org/10.1007/978-1-4020-8539-0.
2. Wells, J.C.K., and Stock, J.T. (2020). Life History Transitions at the Origins of Agriculture: A Model for Understanding How Niche Construction Impacts Human Growth, Demography and Health. Front. Endocrinol. *11*, 325. https://doi.org/10.3389/FENDO.2020.00325.
3. Evershed, R.P., Davey Smith, G., Roffet-Salque, M., Timpson, A., Diekmann, Y., Lyon, M.S., Cramp, L.J.E., Casanova, E., Smyth, J., Whelton, H.L., et al. (2022). Dairying, diseases and the evolution of lactase persistence in Europe. Nature *608*, 336–345. https://doi.org/10.1038/s41586-022-05010-7.
4. Diamond, J. (2002). Evolution, consequences and future of plant and animal domestication. Nature *418*, 700–707. https://doi.org/10.1038/nature01019.
5. Ségurel, L., and Bon, C. (2017). On the Evolution of Lactase Persistence in Humans. Annu. Rev. Genom. Hum. Genet *8*, 297–319. https://doi.org/10.1146/annurev-genom-091416.
6. Macholdt, E., Lede, V., Barbieri, C., Mpoloka, S.W., Chen, H., Slatkin, M., Pakendorf, B., and Stoneking, M. (2014). Tracing pastoralist migrations to southern Africa with lactase persistence alleles. Curr. Biol. *24*, 875–879. https://doi.org/10.1016/j.cub.2014.03.027.
7. Tishkoff, S.A., Reed, F.A., Ranciaro, A., Voight, B.F., Babbitt, C.C., Silverman, J.S., Powell, K., Mortensen, H.M., Hirbo, J. B., Osman, M., et al. (2007). Convergent adaptation of human lactase persistence in Africa and Europe. Nat. Genet. *39*, 31–40. https://doi.org/10.1038/ng1946.
8. Bersaglieri, T., Sabeti, P.C., Patterson, N., Vanderploeg, T., Schaffner, S.F., Drake, J.A., Rhodes, M., Reich, D.E., and Hirschhorn, J.N. (2004). Genetic signatures of strong recent positive selection at the lactase gene. Am. J. Hum. Genet. *74*, 1111–1120. https://doi.org/10.1086/421051.
9. Minster, R.L., Hawley, N.L., Su, C.-T., Sun, G., Kershaw, E.E., Cheng, H., Buhule, O.D., Lin, J., Reupena, M.S., Viali, S., et al. (2016). A thrifty variant in CREBRF strongly influences body mass index in Samoans. Nat. Genet. *48*, 1049–1054. https://doi.org/10.1038/ng.3620.
10. Racimo, F., Gokhman, D., Fumagalli, M., Ko, A., Hansen, T., Moltke, I., Albrechtsen, A., Carmel, L., Huerta-Sánchez, E., and Nielsen, R. (2017). Archaic Adaptive Introgression in TBX15/WARS2. Mol. Biol. Evol. *34*, 509–524. https://doi.org/10.1093/molbev/msw283.
11. Fumagalli, M., Moltke, I., Grarup, N., Racimo, F., Bjerregaard, P., Jørgensen, M.E., Korneliussen, T.S., Gerbault, P., Skotte, L., Linneberg, A., et al. (2015). Greenlandic Inuit show genetic signatures of diet and climate adaptation. Science *349*, 1343–1347. https://doi.org/10.1126/science.aab2319.
12. Schlebusch, C.M., Gattepaille, L.M., Engström, K., Vahter, M., Jakobsson, M., and Broberg, K. (2015). Human adaptation to arsenic-rich environments. Mol. Biol. Evol. *32*, 1544–1555. https://doi.org/10.1093/molbev/msv046.
13. Hassani, A., Azapagic, A., and Shokri, N. (2021). Global predictions of primary soil salinization under changing climate in the 21st century. Nat. Commun. *12*, 6663. https://doi.org/10.1038/s41467-021-26907-3.
14. Nell, J.P., and van Huyssteen, C.W. (2018). Prediction of primary salinity, sodicity and alkalinity in South African soils. S. Afr. J. Plant Soil *35*, 173–178. https://doi.org/10.1080/02571862.2017.1345015.
15. White, L., Romagné, F., Müller, E., Erlebach, E., Weihmann, A., Parra, G., Andrés, A.M., and Castellano, S. (2015). Genetic adaptation to levels of dietary selenium in recent human history. Mol. Biol. Evol. *32*, 1507–1518. https://doi.org/10.1093/molbev/msv043.
16. López Herráez, D., Bauchet, M., Tang, K., Theunert, C., Pugach, I., Li, J., Nandineni, M.R., Gross, A., Scholz, M., and Stoneking, M. (2009). Genetic Variation and Recent Positive Selection in Worldwide Human Populations: Evidence from Nearly 1 Million SNPs. PLoS One *4*, e7888. https://doi.org/10.1371/journal.pone.0007888.
17. Roca-Umbert, A., Garcia-Calleja, J., Vogel-González, M., Fierro-Villegas, A., Bosnjak, A., Ill-Raga, G., Herrera-Fernández, V., Muntané, G., Campelo, F., Vicente, R., et al. (2022). Adaptive archaic introgression related to cellular zinc homeostasis in humans. Preprint at bioRxiv. https://doi.org/10.1101/2022.06.29.498106.
18. Roca-Umbert, A., Caro-Consuegra, R., Londono-Correa, D., Rodriguez-Lozano, G.F., Vicente, R., and Bosch, E. (2022). Understanding signatures of positive natural selection in human zinc transporter genes. Sci. Rep. *12*, 4320. https://doi.org/10.1038/S41598-022-08439-Y.
19. Zhang, C., Li, J., Tian, L., Lu, D., Yuan, K., Yuan, Y., and Xu, S. (2015). Differential natural selection of human zinc transporter genes between African and non-African populations. Sci. Rep. *5*, 9658. https://doi.org/10.1038/srep09658.
20. Bailey, R.L., West, K.P., and Black, R.E. (2015). The epidemiology of global micronutrient deficiencies. Ann. Nutr. Metab. *66*, 22–33. https://doi.org/10.1159/000371618.
21. Bhutta, Z.A., and Salam, R.A. (2012). Global nutrition epidemiology and trends. Ann. Nutr. Metab. *61*, 19–27. https://doi.org/10.1159/000345167.
22. Kambe, T., Tsuji, T., Hashimoto, A., and Itsumura, N. (2015). The Physiological, Biochemical, and Molecular Roles of Zinc Transporters in Zinc Homeostasis and Metabolism. Physiol. Rev. *95*, 749–784. https://doi.org/10.1152/PHYSREV.00035.2014.
23. Monteiro, J.P., Kussmann, M., and Kaput, J. (2015). The genomics of micronutrient requirements. Genes Nutr. *10*, 466. https://doi.org/10.1007/S12263-015-0466-2.
24. Shenkin, A. (2006). Micronutrients in health and disease. Postgrad. Med. J. *82*, 559–567. https://doi.org/10.1136/PGMJ.2006.047670.
25. Triggiani, V., Tafaro, E., Giagulli, V.A., Sabbà, C., Resta, F., Licchelli, B., and Guastamacchia, E. (2009). Role of iodine, selenium and other micronutrients in thyroid function and disorders. Endocr., Metab. Immune Disord.: Drug Targets *9*, 277–294. https://doi.org/10.2174/187153009789044392.
26. Tulchinsky, T.H. (2010). Micronutrient deficiency conditions: Global health issues. Public Health Rev. *32*, 243–255. https://doi.org/10.1007/BF03391600/METRICS.
27. Dhaliwal, S.S., Naresh, R.K., Mandal, A., Singh, R., and Dhaliwal, M.K. (2019). Dynamics and transformations of micronutrients in agricultural soils as influenced by organic matter build-up: A review. Environmen. Sustain. Indic. *1–2*, 100007. https://doi.org/10.1016/J.INDIC.2019.100007.

28. Hurst, R., Siyame, E.W.P., Young, S.D., Chilimba, A.D.C., Joy, E.J.M., Black, C.R., Ander, E.L., Watts, M.J., Chilima, B., Gondwe, J., et al. (2013). Soil-type influences human selenium status and underlies widespread selenium deficiency risks in Malawi. Sci. Rep. *3*, 1425. https://doi.org/10.1038/srep01425.
29. Ibrahim, S.A.Z., Kerkadi, A., and Agouni, A. (2019). Selenium and Health: An Update on the Situation in the Middle East and North Africa. Nutrients *11*, 1457. https://doi.org/10.3390/NU11071457.
30. Pike, V., and Zlotkin, S. (2019). Excess micronutrient intake: defining toxic effects and upper limits in vulnerable populations. Ann. N. Y. Acad. Sci. *1446*, 21–43. https://doi.org/10.1111/NYAS.13993.
31. Wald, N.J. (2022). Folic acid and neural tube defects: Discovery, debate and the need for policy change. J. Med. Screen *29*, 138–146. https://doi.org/10.1177/09691413221102321.
32. Xu, Y., Shan, Y., Lin, X., Miao, Q., Lou, L., Wang, Y., and Ye, J. (2021). Global patterns in vision loss burden due to vitamin A deficiency from 1990 to 2017. Public Health Nutr. *24*, 5786–5794. https://doi.org/10.1017/S1368980021001324.
33. Stevens, G.A., Paciorek, C.J., Flores-Urrutia, M.C., Borghi, E., Namaste, S., Wirth, J.P., Suchdev, P.S., Ezzati, M., Rohner, F., Flaxman, S.R., and Rogers, L.M. (2022). National, regional, and global estimates of anaemia by severity in women and children for 2000–19: a pooled analysis of population-representative data. Lancet. Glob. Health *10*, e627–e639. https://doi.org/10.1016/S2214-109X(22)00084-5.
34. Khan, S.T., Malik, A., Alwarthan, A., and Shaik, M.R. (2022). The enormity of the zinc deficiency problem and available solutions; an overview. Arab. J. Chem. *15*, 103668. https://doi.org/10.1016/J.ARABJC.2021.103668.
35. Gebremichael, G., Demena, M., Egata, G., and Gebremichael, B. (2020). Prevalence of Goiter and Associated Factors Among Adolescents in Gazgibla District, Northeast Ethiopia. Glob. Adv. Health Med. *9*, 2164956120923624. https://doi.org/10.1177/2164956120923624.
36. Xu, J., Wang, J., and Zhao, H. (2023). The Prevalence of Kashin-Beck Disease in China: a Systematic Review and Meta-analysis. Biol. Trace Elem. Res. *201*, 3175–3184. https://doi.org/10.1007/S12011-022-03417-X.
37. Shi, Y., Yang, W., Tang, X., Yan, Q., Cai, X., and Wu, F. (2021). Keshan Disease: A Potentially Fatal Endemic Cardiomyopathy in Remote Mountains of China. Front. Pediatr. *9*, 576916. https://doi.org/10.3389/FPED.2021.576916.
38. De Groote, H., Tessema, M., Gameda, S., and Gunaratna, N. S. (2021). Soil zinc, serum zinc, and the potential for agronomic biofortification to reduce human zinc deficiency in Ethiopia. Sci. Rep. *11*, 8770. https://doi.org/10.1038/s41598-021-88304-6.
39. Halsted, J.A., Ronaghy, H.A., Abadi, P., Haghshenass, M., Amirhakemi, G.H., Barakat, R.M., and Reinhold, J.G. (1972). Zinc deficiency in man. The Shiraz experiment. Am. J. Med. *53*, 277–284. https://doi.org/10.1016/0002-9343(72)90169-6.
40. Kaur, H., and Garg, N. (2021). Zinc toxicity in plants: a review. Planta *253*, 129. https://doi.org/10.1007/S00425-021-03642-Z/METRICS.
41. Becker, M., and Asch, F. (2005). Iron toxicity in rice—conditions and management concepts. Z. Pflanzenernähr. Bodenk. *168*, 558–573. https://doi.org/10.1002/JPLN.200520504.
42. Fraga, C.G. (2005). Relevance, essentiality and toxicity of trace elements in human health. Mol. Aspects Med. *26*, 235–244. https://doi.org/10.1016/J.MAM.2005.07.013.
43. Muneer, S., Siddiqui, I., Majid, H., Zehra, N., Jafri, L., and Khan, A.H. (2022). Practices of vitamin D supplementation leading to vitamin D toxicity: Experience from a Low-Middle Income Country. Ann. Med. Surg. *73*, 103227. https://doi.org/10.1016/J.AMSU.2021.103227.
44. Taylor, P.N., and Davies, J.S. (2018). A review of the growing risk of vitamin D toxicity from inappropriate practice. Br. J. Clin. Pharmacol. *84*, 1121–1127. https://doi.org/10.1111/BCP.13573.
45. Dormitzer, P.R., Ellison, P.T., and Bode, H.H. (1989). Anomalously low endemic goiter prevalence among Efe pygmies. Am. J. Phys. Anthropol. *78*, 527–531. https://doi.org/10.1002/ajpa.1330780408.
46. Haller, B.C., and Messer, P.W. (2019). SLiM 3: Forward Genetic Simulations Beyond the Wright-Fisher Model. Mol. Biol. Evol. *36*, 632–637. https://doi.org/10.1093/molbev/msy228.
47. Lipson, M., Sawchuk, E.A., Thompson, J.C., Oppenheimer, J., Tryon, C.A., Ranhorn, K.L., de Luna, K.M., Sirak, K.A., Olalde, I., Ambrose, S.H., et al. (2022). Ancient DNA and deep population structure in sub-Saharan African foragers. Nature *603*, 290–296. https://doi.org/10.1038/s41586-022-04430-9.
48. Schlebusch, C.M., Sjödin, P., Breton, G., Günther, T., Naidoo, T., Hollfelder, N., Sjöstrand, A.E., Xu, J., Gattepaille, L.M., Vicente, M., et al. (2020). Khoe-San Genomes Reveal Unique Variation and Confirm the Deepest Population Divergence in Homo sapiens. Mol. Biol. Evol. *37*, 2944–2954. https://doi.org/10.1093/MOLBEV/MSAA140.
49. Ragsdale, A.P., Weaver, T.D., Atkinson, E.G., Hoal, E.G., Möller, M., Henn, B.M., and Gravel, S. (2023). A weakly structured stem for human origins in Africa. Nature *617*, 755–763. https://doi.org/10.1038/s41586-023-06055-y.
50. Schlebusch, C.M., Malmström, H., Günther, T., Sjödin, P., Coutinho, A., Edlund, H., Munters, A.R., Vicente, M., Steyn, M., Soodyall, H., et al. (2017). Southern African ancient genomes estimate modern human divergence to 350,000 to 260,000 years ago. Science (1979) *358*, 652–655. https://doi.org/10.1126/SCIENCE.AAO6266/SUPPL_FILE/AAO6266_SCHLEBUSCH_SM.PDF.
51. Gravel, S., Zakharia, F., Moreno-Estrada, A., Byrnes, J.K., Muzzio, M., Rodriguez-Flores, J.L., Kenny, E.E., Gignoux, C.R., Maples, B.K., Guiblet, W., et al. (2013). Reconstructing Native American Migrations from Whole-Genome and Whole-Exome Data. PLoS Genet. *9*, e1004023. https://doi.org/10.1371/journal.pgen.1004023.
52. Gravel, S., Henn, B.M., Gutenkunst, R.N., Indap, A.R., Marth, G.T., Clark, A.G., Yu, F., Gibbs, R.A., 1000 Genomes Project, and Bustamante, C.D. (2011). Demographic history and rare allele sharing among human populations. Proc. Natl. Acad. Sci. USA *108*, 11983–11988. https://doi.org/10.1073/pnas.1019276108.
53. Voight, B.F., Kudaravalli, S., Wen, X., and Pritchard, J.K. (2006). A Map of Recent Positive Selection in the Human Genome. PLoS Biol. *4*, e72. https://doi.org/10.1371/journal.pbio.0040072.
54. Sabeti, P.C., Varilly, P., Fry, B., Lohmueller, J., Hostetter, E., Cotsapas, C., Xie, X., Byrne, E.H., Mccarroll, S.A., Gaudet, R., et al. (2007). Genome-wide detection and characterization of positive selection in human populations. Nature *449*, 913–918. https://doi.org/10.1038/nature06250.

55. Szpiech, Z.A., Novak, T.E., Bailey, N.P., and Stevison, L.S. (2020). High-altitude adaptation in rhesus macaques. Preprint at bioRxiv. https://doi.org/10.1101/2020.05.19.104380.
56. Speidel, L., Forest, M., Shi, S., and Myers, S.R. (2019). A method for genome-wide genealogy estimation for thousands of samples. Nat. Genet. *51*, 1321–1329. https://doi.org/10.1038/s41588-019-0484-x.
57. Field, Y., Boyle, E.A., Telis, N., Gao, Z., Gaulton, K.J., Golan, D., Yengo, L., Rocheleau, G., Froguel, P., McCarthy, M.I., and Pritchard, J.K. (2016). Detection of human adaptation during the past 2000 years. Science *354*, 760–764. https://doi.org/10.1126/science.aag0776.
58. Ferrer-Admetlla, A., Liang, M., Korneliussen, T., and Nielsen, R. (2014). On Detecting Incomplete Soft or Hard Selective Sweeps Using Haplotype Structure. Mol. Biol. Evol. *31*, 1275–1291. https://doi.org/10.1093/molbev/msu077.
59. Weir, B.S., and Cockerham, C.C. (1984). Estimating F-Statistics for the Analysis of Population Structure. Evolution *38*, 1358–1370. https://doi.org/10.2307/2408641.
60. Daub, J.T., Hofer, T., Cutivet, E., Dupanloup, I., Quintana-Murci, L., Robinson-Rechavi, M., and Excoffier, L. (2013). Evidence for Polygenic Adaptation to Pathogens in the Human Genome. Mol. Biol. Evol. *30*, 1544–1558. https://doi.org/10.1093/molbev/mst080.
61. Wishart, D.S., Tzur, D., Knox, C., Eisner, R., Guo, A.C., Young, N., Cheng, D., Jewell, K., Arndt, D., Sawhney, S., et al. (2007). HMDB: the Human Metabolome Database. Nucleic Acids Res. *35*, D521–D526. https://doi.org/10.1093/NAR/GKL923.
62. Ye, K., Cao, C., Lin, X., O'Brien, K.O., and Gu, Z. (2015). Natural selection on HFE in Asian populations contributes to enhanced non-heme iron absorption. BMC Genet. *16*, 61. https://doi.org/10.1186/s12863-015-0223-y.
63. Engelken, J., Espadas, G., Mancuso, F.M., Bonet, N., Scherr, A.L., Jímenez-Álvarez, V., Codina-Solà, M., Medina-Stacey, D., Spataro, N., Stoneking, M., et al. (2016). Signatures of evolutionary adaptation in quantitative trait loci influencing trace element homeostasis in liver. Mol. Biol. Evol. *33*, 738–754. https://doi.org/10.1093/molbev/msv267.
64. Engelken, J., Carnero-Montoro, E., Pybus, M., Andrews, G. K., Lalueza-Fox, C., Comas, D., Sekler, I., de la Rasilla, M., Rosas, A., Stoneking, M., et al. (2014). Extreme Population Differences in the Human Zinc Transporter ZIP4 (SLC39A4) Are Explained by Positive Selection in Sub-Saharan Africa. PLoS Genet. *10*, e1004128. https://doi.org/10.1371/journal.pgen.1004128.
65. Bergström, A., McCarthy, S.A., Hui, R., Almarri, M.A., Ayub, Q., Danecek, P., Chen, Y., Felkel, S., Hallast, P., Kamm, J., et al. (2020). Insights into human genetic variation and population history from 929 diverse genomes. Science *367*, eaay5012. https://doi.org/10.1126/science.aay5012.
66. Auton, A., and Salcedo, T. (2015). In The 1000 genomes project. Assessing Rare Variation in Complex Traits: Design and Analysis of Genetic Studies, E. Zeggini and A. Morris, eds. (Springer). https://doi.org/10.1007/978-1-4939-2824-8_6.
67. Danecek, P., Auton, A., Abecasis, G., Albers, C.A., Banks, E., DePristo, M.A., Handsaker, R.E., Lunter, G., Marth, G.T., Sherry, S.T., et al. (2011). The variant call format and VCFtools. Bioinformatics *27*, 2156–2158. https://doi.org/10.1093/bioinformatics/btr330.
68. Nielsen, R., Akey, J.M., Jakobsson, M., Pritchard, J.K., Tishkoff, S., and Willerslev, E. (2017). Tracing the peopling of the world through genomics. Nature *541*, 302–310. https://doi.org/10.1038/NATURE21347.
69. Lachance, J., Vernot, B., Elbers, C.C., Ferwerda, B., Froment, A., Bodo, J.M., Lema, G., Fu, W., Nyambo, T.B., Rebbeck, T. R., et al. (2012). Evolutionary history and adaptation from high-coverage whole-genome sequences of diverse African hunter-gatherers. Cell *150*, 457–469. https://doi.org/10.1016/J.CELL.2012.07.009.
70. Scheinfeldt, L.B., Soi, S., Thompson, S., Ranciaro, A., Woldemeskel, D., Beggs, W., Lambert, C., Jarvis, J.P., Abate, D., Belay, G., and Tishkoff, S.A. (2012). Genetic adaptation to high altitude in the Ethiopian highlands. Genome Biol. *13*, R1. https://doi.org/10.1186/gb-2012-13-1-r1.
71. Lachance, J., Vernot, B., Elbers, C.C., Ferwerda, B., Froment, A., Bodo, J.M., Lema, G., Fu, W., Nyambo, T.B., Rebbeck, T. R., et al. (2012). Evolutionary history and adaptation from high-coverage whole-genome sequences of diverse African hunter-gatherers. Cell *150*, 457–469. https://doi.org/10.1016/j.cell.2012.07.009.
72. Fan, S., Spence, J.P., Feng, Y., Hansen, M.E.B., Terhorst, J., Beltrame, M.H., Ranciaro, A., Hirbo, J., Beggs, W., Thomas, N., et al. (2023). Whole-genome sequencing reveals a complex African population demographic history and signatures of local adaptation. Cell *186*, 923–939.e14. https://doi.org/10.1016/J.CELL.2023.01.042.
73. Schlebusch, C.M., Skoglund, P., Sjödin, P., Gattepaille, L.M., Hernandez, D., Jay, F., Li, S., De Jongh, M., Singleton, A., Blum, M.G.B., et al. (2012). Genomic variation in seven Khoe-San groups reveals adaptation and complex African history. Science *338*, 374–379. https://doi.org/10.1126/SCIENCE.1227721.
74. Patin, E., Lopez, M., Grollemund, R., Verdu, P., Harmant, C., Quach, H., Laval, G., Perry, G.H., Barreiro, L.B., Froment, A., et al. (2017). Dispersals and genetic adaptation of Bantu-speaking populations in Africa and North America. Science *356*, 543–546. https://doi.org/10.1126/SCIENCE.AAL1988.
75. Delaneau, O., Howie, B., Cox, A.J., Zagury, J.F., and Marchini, J. (2013). Haplotype estimation using sequencing reads. Am. J. Hum. Genet. *93*, 687–696. https://doi.org/10.1016/J.AJHG.2013.09.002.
76. Dyer, S.C., Austine-Orimoloye, O., Azov, A.G., Barba, M., Barnes, I., Barrera-Enriquez, V.P., Becker, A., Bennett, R., Beracochea, M., Berry, A., et al. (2025). Ensembl 2025. Nucleic Acids Res. *53*, D948–D957. https://doi.org/10.1093/NAR/GKAE1071.
77. Tintle, N.L., Borchers, B., Brown, M., and Bekmetjev, A. (2009). Comparing gene set analysis methods on single-nucleotide polymorphism data from Genetic Analysis Workshop 16. BMC Proc. *3*, S96. https://doi.org/10.1186/1753-6561-3-S7-S96.
78. Racimo, F., Marnetto, D., and Huerta-Sánchez, E. (2017). Signatures of Archaic Adaptive Introgression in Present-Day Human Populations. Mol. Biol. Evol. *34*, 296–317. https://doi.org/10.1093/MOLBEV/MSW216.
79. Skov, L., Coll Macià, M., Sveinbjörnsson, G., Mafessoni, F., Lucotte, E.A., Einarsdóttir, M.S., Jonsson, H., Halldorsson, B., Gudbjartsson, D.F., Helgason, A., et al. (2020). The nature of Neanderthal introgression revealed by 27,566 Icelandic genomes. Nature *582*, 78–83. https://doi.org/10.1038/s41586-020-2225-9.
80. Vaughn, A.H., and Nielsen, R. (2024). Fast and Accurate Estimation of Selection Coefficients and Allele Histories from

Ancient and Modern DNA. Mol. Biol. Evol. *41*, msae156. https://doi.org/10.1093/MOLBEV/MSAE156.

81. Stern, A.J., Wilton, P.R., and Nielsen, R. (2019). An approximate full-likelihood method for inferring selection and allele frequency trajectories from DNA sequence data. PLoS Genet. *15*, e1008384. https://doi.org/10.1371/journal.pgen.1008384.
82. Leigh, J.W., and Bryant, D. (2015). popart: full-feature software for haplotype network construction. Methods Ecol. Evol. *6*, 1110–1116. https://doi.org/10.1111/2041-210X.12410.
83. Hermisson, J., and Pennings, P.S. (2017). Soft sweeps and beyond: understanding the patterns and probabilities of selection footprints under rapid adaptation. Methods Ecol. Evol. *8*, 700–716. https://doi.org/10.1111/2041-210X.12808.
84. Pritchard, J.K., Pickrell, J.K., and Coop, G. (2010). The Genetics of Human Adaptation: Hard Sweeps, Soft Sweeps, and Polygenic Adaptation. Curr. Biol. *20*, R208–R215. https://doi.org/10.1016/J.CUB.2009.11.055.
85. Rees, J.S., Castellano, S., and Andrés, A.M. (2020). The Genomics of Human Local Adaptation. Trends Genet. *36*, 415–428. https://doi.org/10.1016/j.tig.2020.03.006.
86. Sabeti, P.C., Varilly, P., Fry, B., Lohmueller, J., Hostetter, E., Cotsapas, C., Xie, X., Byrne, E.H., McCarroll, S.A., Gaudet, R., et al. (2007). Genome-wide detection and characterization of positive selection in human populations. Nature *449*, 913–918. https://doi.org/10.1038/nature06250.
87. Schrider, D.R., and Kern, A.D. (2016). S/HIC: Robust Identification of Soft and Hard Sweeps Using Machine Learning. PLoS Genet. *12*, e1005928. https://doi.org/10.1371/journal.pgen.1005928.
88. Schrider, D.R., and Kern, A.D. (2017). Soft sweeps are the dominant mode of adaptation in the human genome. Mol. Biol. Evol. *34*, 1863–1877. https://doi.org/10.1093/molbev/msx154.
89. Pritchard, J.K., and Di Rienzo, A. (2010). Adaptation - Not by sweeps alone. Nat. Rev. Genet. *11*, 665–667. https://doi.org/10.1038/nrg2880.
90. Berg, J.J., Zhang, X., and Coop, G. (2017). Polygenic Adaptation has Impacted Multiple Anthropometric Traits. Preprint at bioRxiv. https://doi.org/10.1101/167551.
91. Berg, J.J., Zhang, X., and Coop, G. (2019). Polygenic adaptation has impacted multiple anthropometric traits. Preprint at bioRxiv. https://doi.org/10.1101/167551.
92. Daub, J.T., Dupanloup, I., Robinson-Rechavi, M., and Excoffier, L. (2015). Inference of evolutionary forces acting on human biological pathways. Genome Biol. Evol. *7*, 1546–1558. https://doi.org/10.1093/gbe/evv083.
93. Dib, M.J., Elliott, R., and Ahmadi, K.R. (2019). A critical evaluation of results from genome-wide association studies of micronutrient status and their utility in the practice of precision nutrition. Br. J. Nutr. *122*, 121–130. https://doi.org/10.1017/S0007114519001119.
94. Kovacs, G., Montalbetti, N., Franz, M.C., Graeter, S., Simonin, A., and Hediger, M.A. (2013). Human TRPV5 and TRPV6: Key players in cadmium and zinc toxicity. Cell Calcium *54*, 276–286. https://doi.org/10.1016/j.ceca.2013.07.003.
95. Muckenthaler, M.U., Galy, B., and Hentze, M.W. (2008). Systemic iron homeostasis and the iron-responsive element/iron-regulatory protein (IRE/IRP) regulatory network. Annu. Rev. Nutr. *28*, 197–213. https://doi.org/10.1146/ANNUREV.NUTR.28.061807.155521.
96. Khanal, R.C., and Nemere, I. (2008). Regulation of intestinal calcium transport. Annu. Rev. Nutr. *28*, 179–196. https://doi.org/10.1146/ANNUREV.NUTR.010308.161202.
97. Stauber, T., and Jentsch, T.J. (2013). Chloride in vesicular trafficking and function. Annu. Rev. Physiol. *75*, 453–477. https://doi.org/10.1146/ANNUREV-PHYSIOL-030212-183702.
98. Chang, A.R., and Anderson, C. (2017). Dietary Phosphorus Intake and the Kidney. Annu. Rev. Nutr. *37*, 321–346. https://doi.org/10.1146/ANNUREV-NUTR-071816-064607.
99. Jain, G., Ong, S., and Warnock, D.G. (2013). Genetic Disorders of Potassium Homeostasis. Semin. Nephrol. *33*, 300–309. https://doi.org/10.1016/J.SEMNEPHROL.2013.04.010.
100. Reiss, J., and Hahnewald, R. (2011). Molybdenum cofactor deficiency: Mutations in GPHN, MOCS1, and MOCS2. Hum. Mutat. *32*, 10–18. https://doi.org/10.1002/HUMU.21390.
101. Horning, K.J., Caito, S.W., Tipps, K.G., Bowman, A.B., and Aschner, M. (2015). Manganese Is Essential for Neuronal Health. Annu. Rev. Nutr. *35*, 71–108. https://doi.org/10.1146/ANNUREV-NUTR-071714-034419.
102. Freitas, S.R.S. (2018). Molecular Genetics of Salt-Sensitivity and Hypertension: Role of Renal Epithelial Sodium Channel Genes. Am. J. Hypertens. *31*, 172–174. https://doi.org/10.1093/AJH/HPX184.
103. Rossier, B.C., Pradervand, S., Schild, L., and Hummler, E. (2002). Epithelial sodium channel and the control of sodium balance: Interaction between genetic and environmental factors. Annu. Rev. Physiol. *64*, 877–897. https://doi.org/10.1146/ANNUREV.PHYSIOL.64.082101.143243/CITE/REFWORKS.
104. Houillier, P. (2014). Mechanisms and regulation of renal magnesium transport. Annu. Rev. Physiol. *76*, 411–430. https://doi.org/10.1146/ANNUREV-PHYSIOL-021113-170336/CITE/REFWORKS.
105. Polimanti, R., Yang, B.Z., Zhao, H., and Gelernter, J. (2016). Evidence of Polygenic Adaptation in the Systems Genetics of Anthropometric Traits. PLoS One *11*, e0160654. https://doi.org/10.1371/journal.pone.0160654.
106. Fumagalli, M., Moltke, I., Grarup, N., Racimo, F., Bjerregaard, P., Jørgensen, M.E., Korneliussen, T.S., Gerbault, P., Skotte, L., Linneberg, A., et al. (2015). Greenlandic Inuit show genetic signatures of diet and climate adaptation. Science *349*, 1343–1347. https://doi.org/10.1126/science.aac5017.
107. Hengl, T., Leenaars, J.G.B., Shepherd, K.D., Walsh, M.G., Heuvelink, G.B.M., Mamo, T., Tilahun, H., Berkhout, E., Cooper, M., Fegraus, E., et al. (2017). Soil nutrient maps of Sub-Saharan Africa: assessment of soil nutrient content at 250 m spatial resolution using machine learning. Nutr. Cycl. Agroecosyst. *109*, 77–102. https://doi.org/10.1007/s10705-017-9870-x.
108. Xia, Y., Hill, K.E., Byrne, D.W., Xu, J., and Burk, R.F. (2005). Effectiveness of selenium supplements in a low-selenium area of China. Am. J. Clin. Nutr. *81*, 829–834. https://doi.org/10.1093/AJCN/81.4.829.
109. Osier, M.V., Pakstis, A.J., Soodyall, H., Comas, D., Goldman, D., Odunsi, A., Okonofua, F., Parnas, J., Schulz, L.O., Bertranpetit, J., et al. (2002). A global perspective on genetic variation at the ADH genes reveals unusual patterns of linkage disequilibrium and diversity. Am. J. Hum. Genet. *71*, 84–99. https://doi.org/10.1086/341290.
110. Han, Y., Gu, S., Oota, H., Osier, M.V., Pakstis, A.J., Speed, W. C., Kidd, J.R., and Kidd, K.K. (2007). Evidence of positive

selection on a class I ADH locus. Am. J. Hum. Genet. *80*, 441–456. https://doi.org/10.1086/512485.

111. Perry, G.H., Dominy, N.J., Claw, K.G., Lee, A.S., Fiegler, H., Redon, R., Werner, J., Villanea, F.A., Mountain, J.L., Misra, R., et al. (2007). Diet and the evolution of human amylase gene copy number variation. Nat. Genet. *39*, 1256–1260. https://doi.org/10.1038/ng2123.
112. Manus, M.B. (2018). Evolutionary mismatch. Evol. Med. Public Health *2018*, 190–191. https://doi.org/10.1093/EMPH/EOY023.
113. Perry, G.H., and Dominy, N.J. (2009). Evolution of the human pygmy phenotype. Trends Ecol. Evol. *24*, 218–225. https://doi.org/10.1016/J.TREE.2008.11.008.
114. Kelly, F.C., and Snedden, W.W. (1958). Prevalence and geographical distribution of endemic goitre. Bull. World Health Organ. *18*, 5–173.
115. Singh, B.K., and Yen, P.M. (2017). A clinician's guide to understanding resistance to thyroid hormone due to receptor mutations in the TRα and TRβ isoforms. Clin. Diabetes Endocrinol. *3*, 8–11. https://doi.org/10.1186/S40842-017-0046-Z.
116. Biban, B.G., and Lichiardopol, C. (2017). Iodine Deficiency, Still a Global Problem? Curr. Health Sci. J. *43*, 103–111. https://doi.org/10.12865/CHSJ.43.02.01.
117. Xu, J., Ke, Z., Xia, J., He, F., and Bao, B. (2016). Change of body height is regulated by thyroid hormone during metamorphosis in flatfishes and zebrafish. Gen. Comp. Endocrinol. *236*, 9–16. https://doi.org/10.1016/J.YGCEN.2016.06.028.
118. Rose, S.R. (1995). Isolated central hypothyroidism in short stature. Pediatr. Res. *38*, 967–973. https://doi.org/10.1203/00006450-199512000-00023.
119. Vázquez-Vázquez, A., Azcorra, H., Falfán, I., Argáeź, J., Kantun, D., and Dickinson, F. (2013). Effects of maya ancestry and environmental variables on knee height and body proportionality in growing individuals in merida, yucatan. Am. J. Hum. Biol. *25*, 586–593. https://doi.org/10.1002/AJHB.22417.
120. Lopez, M., Choin, J., Sikora, M., Siddle, K., Harmant, C., Costa, H.A., Silvert, M., Mouguiama-Daouda, P., Hombert, J.M., Froment, A., et al. (2019). Genomic Evidence for Local Adaptation of Hunter-Gatherers to the African Rainforest. Curr. Biol. *29*, 2926–2935.e4. https://doi.org/10.1016/J.CUB.2019.07.013.
121. Migliano, A.B., Vinicius, L., and Lahr, M.M. (2007). Life history trade-offs explain the evolution of human pygmies. Proc. Natl. Acad. Sci. USA *104*, 20216–20219. https://doi.org/10.1073/PNAS.0708024105/SUPPL_FILE/IMAGE1811.GIF.
122. Liu, Y., Tian, X., Liu, R., Liu, S., and Zuza, A.V. (2021). Key driving factors of selenium-enriched soil in the low-Se geological belt: A case study in Red Beds of Sichuan Basin, China. Catena *196*, 104926. https://doi.org/10.1016/J.CATENA.2020.104926.
123. Savas, S., Briollais, L., Ibrahim-Zada, I., Jarjanazi, H., Choi, Y.H., Musquera, M., Fleshner, N., Venkateswaran, V., and Ozcelik, H. (2010). A whole-genome SNP association study of NCI60 cell line panel indicates a role of Ca2+ signaling in selenium resistance. PLoS One *5*, e12601. https://doi.org/10.1371/JOURNAL.PONE.0012601.
124. Sarangi, G.K., Romagné, F., and Castellano, S. (2018). Distinct Patterns of Selection in Selenium-Dependent Genes between Land and Aquatic Vertebrates. Mol. Biol. Evol. *35*, 1744–1756. https://doi.org/10.1093/MOLBEV/MSY070.
125. Pietschmann, N., Rijntjes, E., Hoeg, A., Stoedter, M., Schweizer, U., Seemann, P., and Schomburg, L. (2014). Selenoprotein P is the essential selenium transporter for bones. Metallomics *6*, 1043–1049. https://doi.org/10.1039/C4MT00003J.
126. Roca-Umbert, A., Garcia-Calleja, J., Vogel-González, M., Fierro-Villegas, A., Ill-Raga, G., Herrera-Fernández, V., Bosnjak, A., Muntané, G., Gutiérrez, E., Campelo, F., et al. (2023). Human genetic adaptation related to cellular zinc homeostasis. PLoS Genet. *19*, e1010950. https://doi.org/10.1371/JOURNAL.PGEN.1010950.
127. Ryan, J., Rashid, A., Torrent, J., Yau, S.K., Ibrikci, H., Sommer, R., and Erenoglu, E.B. (2013). Micronutrient Constraints to Crop Production in the Middle East–West Asia Region: Significance, Research, and Management. Adv. Agron. *122*, 1–84. https://doi.org/10.1016/B978-0-12-417187-9.00001-2.
128. Prasad, A.S. (2013). Discovery of Human Zinc Deficiency: Its Impact on Human Health and Disease. Adv. Nutr. *4*, 176–190. https://doi.org/10.3945/AN.112.003210.
129. Ishfaq, M., Wakeel, A., Shahzad, M.N., Kiran, A., and Li, X. (2021). Severity of zinc and iron malnutrition linked to low intake through a staple crop: a case study in east-central Pakistan. Environ. Geochem. Health *43*, 4219–4233. https://doi.org/10.1007/S10653-021-00912-3.
130. Rehman, A., Farooq, M., Ullah, A., Nadeem, F., Im, S.Y., Park, S.K., and Lee, D.J. (2020). Agronomic Biofortification of Zinc in Pakistan: Status, Benefits, and Constraints. Front. Sustain. Food Syst. *4*, 591722. https://doi.org/10.3389/FSUFS.2020.591722/BIBTEX.
131. Vyshpolsky, F., Qadir, M., Karimov, A., Mukhamedjanov, K., Bekbaev, U., Paroda, R., Aw-Hassan, A., and Karajeh, F. (2008). Enhancing the productivity of high-magnesium soil and water resources in Central Asia through the application of phosphogypsum. Land Degrad. Dev. *19*, 45–56. https://doi.org/10.1002/LDR.814.
132. Karimov, A., Qadir, M., Noble, A., Vyshpolsky, F., and Anzelm, K. (2009). Development of Magnesium-Dominant Soils Under Irrigated Agriculture in Southern Kazakhstan. Pedosphere *19*, 331–343. https://doi.org/10.1016/S1002-0160(09)60124-7.
133. Hwang, J.Y., Lee, S.H., Go, M.J., Kim, B.J., Kou, I., Ikegawa, S., Guo, Y., Deng, H.W., Raychaudhuri, S., Kim, Y.J., et al. (2013). Meta-analysis identifies a MECOM gene as a novel predisposing factor of osteoporotic fracture. J. Med. Genet. *50*, 212–219. https://doi.org/10.1136/JMEDGENET-2012-101156.
134. Castiglioni, S., Cazzaniga, A., Albisetti, W., and Maier, J.A. M. (2013). Magnesium and Osteoporosis: Current State of Knowledge and Future Research Directions. Nutrients *5*, 3022–3033. https://doi.org/10.3390/NU5083022.
135. Racimo, F., Sankararaman, S., Nielsen, R., and Huerta-Sánchez, E. (2015). Evidence for archaic adaptive introgression in humans. Nat. Rev. Genet. *16*, 359–371. https://doi.org/10.1038/nrg3936.
136. Huerta-Sánchez, E., Jin, X., Bianba, Z., Bianba, Z., Peter, B. M., Vinckenbosch, N., Liang, Y., Yi, X., He, M., Somel, M., et al. (2014). Altitude adaptation in Tibetans caused by introgression of Denisovan-like DNA. Nature *512*, 194–197. https://doi.org/10.1038/nature13408.
137. Deschamps, M., Laval, G., Fagny, M., Itan, Y., Abel, L., Casanova, J.L., Patin, E., and Quintana-Murci, L. (2016). Genomic Signatures of Selective Pressures and Introgression

from Archaic Hominins at Human Innate Immunity Genes. Am. J. Hum. Genet. *98*, 5–21. https://doi.org/10.1016/j.ajhg.2015.11.014.

138. Shahid, S.A., Zaman, M., and Heng, L. (2018). Soil Salinity: Historical Perspectives and a World Overview of the Problem. In Guideline for Salinity Assessment, Mitigation and Adaptation Using Nuclear and Related Techniques, pp. 43–53. https://doi.org/10.1007/978-3-319-96190-3_2.
139. Grafen, A., and Vickerman, K. (1989). The phylogenetic regression. Philos. Trans. R. Soc. Lond. B Biol. Sci. *326*, 119–157. https://doi.org/10.1098/RSTB.1989.0106.
140. Freckleton, R.P., Harvey, P.H., and Pagel, M. (2002). Phylogenetic analysis and comparative data: A test and review of evidence. Am. Nat. *160*, 712–726. https://doi.org/10.1086/343873/ASSET/IMAGES/LARGE/FG3.JPEG.
141. Günther, T., and Coop, G. (2013). Robust identification of local adaptation from allele frequencies. Genetics *195*, 205–220. https://doi.org/10.1534/GENETICS.113.152462/-/DC1/GENETICS.113.152462-6.PDF.
142. Key, F.M., Abdul-Aziz, M.A., Mundry, R., Peter, B.M., Sekar, A., D'Amato, M., Dennis, M.Y., Schmidt, J.M., and Andrés, A.M. (2018). Human local adaptation of the TRPM8 cold receptor along a latitudinal cline. PLoS Genet. *14*, e1007298. https://doi.org/10.1371/journal.pgen.1007298.
143. Alewell, C., Ringeval, B., Ballabio, C., Robinson, D.A., Panagos, P., and Borrelli, P. (2020). Global phosphorus shortage will be aggravated by soil erosion. Nat. Commun. *11*, 4546. https://doi.org/10.1038/s41467-020-18326-7.
144. Stevelink, R., Pangilinan, F., Jansen, F.E., Braun, K.P.J., A.M., International League Against Epilepsy Consortium on Complex Epilepsies, Molloy, A.M., Brody, L.C., and Koeleman, B. P.C. (2019). Assessing the genetic association between vitamin B6 metabolism and genetic generalized epilepsy. Mol. Genet. Metab. Rep. *21*, 100518. https://doi.org/10.1016/j.ymgmr.2019.100518.

The American Journal of Human Genetics, Volume *112*

Supplemental information

Global impact of micronutrients in modern human evolution

Jasmin Rees, Sergi Castellano, and Aida M. Andrés

Supplemental Materials

Data

Data S1

Table 1: **All micronutrient-associated genes used in this study associated with the uptake, metabolism or regulation of 13 micronutrients.** When genes are associated with multiple micronutrients, their most supported association given in “Micronutrient” with secondary or tertiary associations given in “Other Associations”. Genes removed following the positive mask[2] indicated in the “Removed During Pruning” column. Gene regions as taken from ensemble[32] and suggested from the literature[33–51].

Data S2

Table 1: **Chi-squared test for enrichment of significant SNPs across all MA-genes according to the 5% empirical tail of F_{ST}.** “Total SNPs” = SNPs across all MA-genes in the given population-Yoruba pair; “Exp-Sig” = 5% of total SNPs; “Exp-NonSig” = 95% of total SNPs; “Obs-Sig” = SNPs in the 5% empirical tail of F_{ST} for the given population-Yoruba pair; “Obs-NonSig” = SNPs outside the 5% empirical tail of F_{ST} for the given population-Yoruba pair; “Enrichment” = percentage more observed significant SNPs than expected; “Chi-Squared” = significance of chi-squared test.

Table 2: **Chi-squared test for enrichment of significant SNPs across all MA-genes according to the 1% empirical tail of F_{ST}.** “Total SNPs” = SNPs across all MA-genes in the given population-Yoruba pair; “Exp-Sig” = 1% of total SNPs; “Exp-NonSig” = 99% of total SNPs; “Obs-Sig” = SNPs in the 1% empirical tail of F_{ST} for the given population-Yoruba pair; “Obs-NonSig” = SNPs outside the 1% empirical tail of F_{ST} for the given population-Yoruba pair; “Enrichment” = percentage more observed significant SNPs than expected; “Chi-Squared” = significance of chi-squared test.

Table 3: **Chi-squared test for enrichment of significant SNPs across all MA-genes according to the 5% empirical tail of $Relate$.** “Total SNPs” = SNPs across all MA-genes in the given population; “Exp-Sig” = 5% of total SNPs; “Exp-NonSig” = 95% of total SNPs; “Obs-Sig” = SNPs in the 5% empirical tail of $Relate$ for the given population; “Obs-NonSig” = SNPs outside the 5% empirical tail of $Relate$ for the given population; “Enrichment” = percentage more observed significant SNPs than expected; “Chi-Squared” = significance of chi-squared test.

Table 4: **Chi-squared test for enrichment of significant SNPs across all MA-genes according to the 1% empirical tail of $Relate$.** “Total SNPs” = SNPs across all MA-genes in the given population; “Exp-Sig” = 1% of total SNPs; “Exp-NonSig” = 99% of total SNPs; “Obs-Sig” = SNPs in the 1% empirical tail of $Relate$ for the given population;

“Obs-NonSig” = SNPs outside the 1% empirical tail of $Relate$ for the given population; “Enrichment” = percentage more observed significant SNPs than expected; “Chi-Squared” = significance of chi-squared test.

Table 5: **Chi-squared test for enrichment of significant SNPs across individual MA-gene sets according to the 5% empirical tail of $\boldsymbol{F_{ST}}$.** “Total SNPs” = SNPs across all MA-genes in a given MA-gene set in the given population-Yoruba pair; “Exp-Sig” = 5% of total SNPs; “Exp-NonSig” = 95% of total SNPs; “Obs-Sig” = SNPs in the 5% empirical tail of F_{ST} for the given population-Yoruba pair; “Obs-NonSig” = SNPs outside the 5% empirical tail of F_{ST} for the given population-Yoruba pair; “Enrichment” = percentage more observed significant SNPs than expected; “Chi-Squared” = significance of chi-squared test.

Table 5: **Chi-squared test for enrichment of significant SNPs across individual MA-gene sets according to the 5% empirical tail of $\boldsymbol{Relate}$**“Total SNPs” = SNPs across all MA-genes in a given MA-gene set in the given population; “Exp-Sig” = 5% of total SNPs; “Exp-NonSig” = 95% of total SNPs; “Obs-Sig” = SNPs in the 5% empirical tail of $Relate$for the given population; “Obs-NonSig” = SNPs outside the 5% empirical tail of $Relate$for the given population; “Enrichment” = percentage more observed significant SNPs than expected; “Chi-Squared” = significance of chi-squared test.

<u>Data S3</u>
Table 1: Proportion of MA-gene sets with strong signatures of positive selection according to $\boldsymbol{Relate}$. “Proportion” =Proportion of MA-genes within each MA-gene set (“Micronutrient”) with SNP(s) with signatures of positive selection in the 1% empirical tail of $Relate$ for the given population (“Population”) of interest.

Table 2: Proportion of MA-gene sets with strong signatures of positive selection according to $\boldsymbol{F_{ST}}$. “Proportion” =Proportion of MA-genes within each MA-gene set (“Micronutrient”) with SNP(s) with signatures of positive selection in the 1% empirical tail of F_{ST} for the given population (“Population”) of interest.

Table 3: Number of candidate SNPs identified in each MA-gene set according to $\boldsymbol{Relate}$. Number of candidate SNPs (“No. candidate SNPs”), those SNP(s) with signatures of positive selection in the 1% empirical tail of $Relate$, for each MA-gene set (“Micronutrient”) for the given population (“Population”) of interest.

Table 4: Number of candidate SNPs identified in each MA-gene set according to $\boldsymbol{F_{ST}}$. Number of candidate SNPs (“No. candidate SNPs”), those SNP(s) with signatures of positive selection in the 1% empirical tail of F_{ST} , for each MA-gene set (“Micronutrient”) for the given population (“Population”) of interest.

Data S4

Table 1: Results of the $CLUES2$ analysis of 19 candidate SNPs of iron- and calcium-associated genes. For each population (“Population”), gene (“Gene”) and SNP of interest (positions in hg38 given by “Chromosome” and “Position”; see **Note S5** and **Table S5** for criteria in choosing these SNPs), the jointly calculated likelihood of selection (“-LOG10(p value)”) and selection coefficient “SelectionMLE1” from each epoch “Epoch” (in generations) to present.

Note S1: Simulations

The forward-simulator SLiM[1] was used to simulate genomic segments of approximately 100,000 base pairs. Each segment was initiated with random nucleotides across its length and included exon, intron and non-coding regions. Variable recombination rates were also specified across this region, modeled according to the inferred distribution of recombination rates in the human genome (as calculated from chr15 of the 929 individuals of the HGDP dataset[2] with 100 different recombination rates given across this region). A gamma distribution of mean 1.311 and shape parameter 0.509 was used to draw recombination rates. The mutation rate was uniform throughout the 100 kb region, specified as per generation and following the Jukes-Cantor model.

We simulated the demographic history of four metapopulations: African, European, East Asian and American, as a combination of two pre-existing demographic models[3,4]. We integrated the inferred demographic history of the Puerto Rican population from the latter into the former model and use it as our proxy of an American population. Here, we note that we have not included deep structure in Africa[5,7,9,11] in our simulations, and emphasise that these simulations are 1) most informative for power in populations with demographic histories similar to those modelled here and 2) should be considered a broad overview of the power of pre-existing methods to identify selection on standing variation. Including deep structure in our simulations would likely improve the power of some methods (*e.g.,* leading to a higher number of older coalescence events that may be leveraged by Relate) and decrease the power of others (e.g., increasing differentiation between some populations and reducing the power of cross-population methods like F_{ST} to identify selected alleles from those with frequency increases under ancient drift) and we encourage future exploration of the effect of deep structure on inferring signatures of positive selection.

The onset of positive selection was set at one of four timepoints (1 kya, 5 kya, 10 kya and 40 kya) in only one of the four metapopulations. A single polymorphic allele segregating in the focal population is tagged given that it lies within the centre 10 kbp of the region and has a frequency of 0.1-0.15. The tagged mutation is then given a selection coefficient drawn from a uniform distribution between 0.001 and 0.005 and hence represents positive selection on standing variation. Each successful simulation run is a proxy for weak selection acting on a single genomic region, analogous to a gene region or haplotype, or can be grouped into “gene sets” to approximate polygenic selection.

An initial burn-in period was covered between 1.66 mya and 70 kya to allow the ancestral population to reach mutation-drift equilibrium. Beneficial, neutral or deleterious mutations were initiated in the exon regions, where the selection coefficients of deleterious mutations were drawn from a gamma distribution (mean: -0.03 and shape parameter: 0.2[6,8]) and beneficial mutations drawn from an exponential distribution (mean: 0.01, capped at 0.05). Neutral mutations appear in the exon, intron and non-coding regions.

To reduce CPU time, simulations were rescaled by a factor 5, reducing the number of simulated individuals. Mutation rate, recombination rate and selection coefficients were scaled up whilst effective population size scaled down, maintaining the necessary

population-genetic parameters[1]. The generation time was also downscaled by the same factor to account for the fact that genetic drift occurs faster in smaller populations. This reduced the CPU time by over a factor 20.

The output from the burn-in stage was then scaled up to 14,474 individuals via random mating. This represents the ancestral African population at 70 kya, which then undergoes population splits, expansions and migrations (as described in **Fig 1**) and the initiation of positive selection in a single segregating allele. For each scenario (the combination of one selection timepoint in one metapopulation), ~10,000 simulations were run on the requirement that the tagged mutation remains polymorphic in the focal population. For each run, VCF files of 50 individuals for each metapopulation were generated as output, alongside the position, selection coefficient and final frequency of the tagged mutation. The distribution of derived allele frequency (DAF) of selected alleles in the focal population are shown in Fig S1. A CSV files was also generated containing information on the inclusive upper bound position of each recombination rate, which was converted to a standard genetic map format, where recombination rate is given in cM/Mb. To simulate neutrality, a matched number of post-burn-in simulations were ran with no initiation of positive selection.

Note S2: Power analysis

Seven methods were applied to identify the genetic signatures of positive selection in the simulated genetic data. Four of these methods use haplotype structure to infer SNPs with evidence of positive selection but do so in subtly different ways. iHS and nSL both consider the length of haplotype homozygosity (where extended haplotype homozygosity is indicative of alleles rapidly rising in frequency, as expected under strong positive selection), with iHS measuring length as the recombination distance and nSL measuring length as the number of segregating sites[10,12]. nSL is an extension of iHS, a commonly used method to identify positive selection, and has been suggested to be more powerful when detecting selective sweeps on standing variation[12]. XPEHH and XPnSL are further extensions of these two methods (of iHS and nSL respectively) and compare the haplotype homozygosity between two populations to identify SNPs with unusually long haplotype length in one population[13,14].

The remaining methods evaluated include one which uses allele differentiation between populations to identify signatures of positive selection ($\mathrm{F_{ST}}$[15]) and two that consider genealogical-based evidence of positive selection (Relate and SDS[16,17]). Relate first infers local trees along the genome (where unique trees are separated by recombination breakpoints); before simultaneously estimating branch lengths, mutation rates and effective population sizes to re-infer the trees[16]. The inferred trees can then be used to evaluate the probability of a variant's trajectory under neutrality, leveraging the inferred history of the allele. SDS, however, simply uses the inferred tip branch lengths to identify trees with short terminal tips and, by extension, recent rapid allele frequency change[17].

All haplotype-based statistics were calculated using the $\mathrm{SELSCAN}$ programme[18] and normalized according to SNP frequency. For XPEHH and XPnSL, calculations were repeated for each combination of focal population with the three remaining populations.

$\mathrm{VCFTOOLS}$[19] was used to calculate per-site F_{ST} according to the Weir and Cockerham (1984) method[15], again for each combination of each focal population with the three remaining populations. The RELATE programme[16] was used to infer genealogies across simulated gene regions, re-infer branch lengths and calculate the $\log_{10}p - \mathrm{value}$ for positive selection. The RELATE programme was also used to directly calculate SDS on the inferred genealogies[16,17].

<u>Power of methods</u>

We categorised selected SNPs as having signatures of positive selection if they fall in the extreme 5% tail of the distribution of neutral simulations. These were built from each method's output values calculated on VCFs simulated from the neutral simulations (the same demographic scenario but with no onset of positive selection in any metapopulation). We caution that whilst the accuracy calculated for the African, European, East Asian and American populations are useful for observing patterns across methods and time, these populations should not be directly compared to each other. This is because we condition on the selected allele to persist to the end of the simulation. While this is a necessary condition widely used in comparable power analyses, it does result in differential biases across populations (since differences in demography result in differences in the probability of an allele to survive to the time of sampling). Therefore, the final set of simulated genomic regions is informative but not perfectly comparable across demographic histories and populations, even within this paper.

The highest true positive rate (TPR) of identification of SNPs under positive selection is observed when using F_{ST} for cross-population methods and Relate for single-population methods[15,16], with the haplotype-based methods and remaining tree-based method SDS displaying poor accuracy in comparison except in particular cases (**Fig S2-3**). This is likely due to the fact that we simulate selection on standing genetic variation. In cases where selection is recent or ongoing and on the same haplotype background (as would be expected under selection on *de novo* mutation), we would expect the power of haplotype-based methods to be substantially higher. We also observe minimal differences in the TPR of the haplotype-based methods that use recombination distance (iHS and XPEHH[10,13]) compared to those that use number of segregating sites as a proxy for distance (nSL and XPnSL[12,14]), perhaps because power is always quite low. For all methods, and as expected, the highest accuracy is observed for the oldest simulated selection (positive selection initiated at 40 kya) and for selection in the simulated population with the largest Ne (the African population; **Figs S2-3**).

The only exception to this pattern is for recent selection which, as expected, in some populations is best identified by SDS (10,000 years ago or earlier; **Fig S2**). Nevertheless, SDS has still low TPR at those timepoints, has much lower TPR than other statistics at older timepoints, and is limited in the small sample sizes available in this study, making it a less suitable statistic overall for our purposes. Further, 40,000 years represents a particularly interesting timeframe that encompasses major human migrations and the subsequent exposure to novel selective pressures.

Further, TPR of can be as high as 69.6% and 86.9% for Relate and F_{ST}, respectively, when selection is in the simulated African population and selection coefficients between

0.04-0.05% (**Figs S4-5**), with TPR sharply increasing with derived allele frequencies higher than 50% (**Figs S6-7**). It follows that, while our simulation design does not allow to confirm this, we expect considerably higher TPR than in **Figs S2-3** with selection coefficients over ~ 0.04.and/or frequency of the derived allele higher than 60%. In this simulation design, the false negative rate (FNR) is equal to 1-TPR and therefore follows the inverse patterns as described above, being as low as 30.4% and 13.1% for Relate and $\mathrm{F_{ST}}$, respectively, when selection is in the simulated African population and selection coefficients between 0.04-0.05%.

The false positive rate (FPR) is below 8.6% for all the tested methods, with minimal differences amongst individual methods (**Figs S8-9**). The FPR of the two most promising methods ($\mathrm{F_{ST}}$ and Relate[15,16]) are as follows: the FPR of $\mathrm{F_{ST}}$ is below 7.6% across all timepoints and populations (in comparison to FPR ~5% to other cross-population methods; **Fig S9**); the FPR of Relate is below 5% and lower than compared methods for all populations and timepoints except selection at 40kya in the simulated Asian and American populations (**Fig S8**; FPR climbs to 8.5% for selection at 40kya in America). We emphasise that whilst the empirical tails are enriched for targets of positive selection, especially in the case of $\mathrm{F_{ST}}$ (**Figs S2-3**), not all SNPs in this tail are true targets.

We also evaluate the TPR and FPR when using the raw output of Relate $(\log_{10}\mathrm{p}-\mathrm{value})$ to identify SNPs under positive selection for the same significance threshold (5%, or $\mathrm{p}-\mathrm{value}=0.05$). Given that the raw output of Relate is the transformed probability of a mutation rising to its observed contemporary frequency given it's inferred coalescence history, this method may have reduced power when applied to small sample sizes of high stochasticity and / or samples that poorly represent the full coalescence history (in comparison to using neutral distributions which incorporate more information on the expected distribution under neutrality, including increased stochasticity due to small sample sizes). We hence compared the use of the tail of the neutral distribution and the raw output of Relate to identify SNPs under positive selection for two sample sizes: 25 and 50 simulated individuals. For all simulated populations, using the neutral distribution to identify SNPs under positive selection leads to a higher TPR than using the $\log_{10}\mathrm{pvalue}$ when sample sizes are decreased to 25 individuals (**Fig S10**). With sample sizes of 50 individuals, the neutral distribution also displays a higher TPR in the simulated African population but performs poorly in the simulated European and East Asian populations (with no difference in the TPR in the simulated American population; **Fig S10**). This is likely because the neutral distribution better incorporates the high variance in small-Ne populations and small samples sizes than the raw Relate output can. The patterns of FPR are less clear, but we note that the difference in FPR between SNPs identified as under positive selection according to the neutral distribution versus the $\log_{10}\mathrm{pvalue}$ is < 2% for all sample sizes, tested timepoints and populations (**Fig S11**).

We conclude that when sample sizes are small (~ 25) in real data examples, the empirical distribution should be used to identify SNPs under positive selection with Relate. With larger sample sizes, the $\log_{10}\mathrm{pvalue}$ may have slightly higher power in very specific cases, for example in populations with demographic histories similar to the

simulated European and East Asian populations. We thus select significant SNPs based on the extreme 5% tail of the neutral distribution for Relate too.

Finally, we evaluate the TPR of the gene-set enrichment method $\mathrm{SUMSTAT}$[20] to identify polygenic selection. This method extracts the most significant pvalue (in the direction of selection) of each gene within a gene set, summing across the gene set to give a final score (or **$\mathbf{SUMSTAT}$ value**). Hence, this method considers the signatures of positive selection on potentially small effect mutations across the entire gene set and has been shown to be more powerful than gene set enrichment analysis in identifying polygenic selection[21]. Gene sets with $\mathrm{SUMSTAT}$ summed values in the 5% tail of the neutral distribution (where this distribution is built from gene sets comprising gene regions simulated under neutrality, see above) are identified as those with signatures of positive selection.

We applied the $\mathrm{SUMSTAT}$ method to the two most promising methods ($\mathrm{F_{ST}}$ and Relate[15,16]). To explore the effect of gene set size and proportion of genes in the gene sets under selection, we built gene sets of various sizes (10, 20, 40, 60) and with varying proportions of genes under selection (20%, 40%, 60%, 80% and 100% gene regions under a selection) by random sampling simulated gene regions (**Fig. S12-13**). $\mathrm{SUMSTAT}$ with $\mathrm{F_{ST}}$ have high TPR for selection initiated at 40,000 years ago for gene sets of all sizes with 100% of genes under selection (TPR $> 82\%$ in the simulated African population, TPR $> 79\%$ when considering all simulated populations) and for large gene sets with proportions of genes under selection as low as 60% (e.g., TPR $> 84.9\%$ for gene sets of size 40 or larger when selection is at 40,000 years ago in the simulated African population, TPR $> 70.3\%$ when considering all simulated populations; **Fig S13**). $\mathrm{SUMSTAT}$ with $\mathrm{Relate\ pvalues}$ only has comparable TPRs for large gene sets with 80% or more genes under selection, and is more sensitive than SUMSTAT with $\mathrm{F_{ST}}$ to decreasing gene set size or proportion of genes under selection (**Fig S12**). Still, for selection initiated at 40,000 years ago, a TPR of $> 76.8\%$ can be achieved in the simulated African population if the gene sets are large (40 or 60 genes) and all genes are under selection (with TPR maintained $> 73\%$ when considering all simulated populations). If the proportion of genes under selection in these gene sets drops to 80%, TPR can vary between $54.5\% - 99.\%$, with a TPR $> 65.3\%$ for gene sets of size 60 when considering all simulated populations.

Note S3: Population Analysis

We use 929 full human genomes from the HGDP dataset (as published by[2]), which encompasses 54 populations across Africa, the Middle-east, Europe, East Asia, Central-South Asia, Oceania and the Americas and represents a significant proportion of human ethnic and cultural diversity. Since low sample sizes can significantly reduce the power to identify the genomic signatures of positive selection[22,23], we aim to merge populations with sample sizes below 20 with their geographically closest populations. In these cases, we acknowledge that the signatures of fine scale positive selection in response to extremely localised micronutrient soil levels may be lost, but we consider this a necessary step to maintain adequate power to identify positive selection that may be shared across these geographically close populations.

We carried out population analysis to verify that this criterion agreed in all cases with patterns of population differentiation. We calculated PCs for each metapopulation using plink[24], having thinned for linkage disequilibrium (pruning r^2 values above 0.2) and using windows of 50 kbp and window step size of 10 bp (see **Fig. S14-20**). This analysis confirmed that grouping by geography agrees with population differentiation, with two exceptions (see below), and we hence group according to this criterion.

When grouped, our final dataset comprised of 913 individuals from 40 populations, of which 10 are a result of merging (see **Table S16**). Two merged populations do not follow geography (Bantu-speaking population and the Xibo-Mongolian population), but instead reflect recent migrations[25–27]. Two populations were removed from our analysis (Columbian, n=7; Cambodian, n=9) since they do not group naturally geographically or genetically. Despite their small sample size, the Ju|'hoan population (n=6) was retained in our final dataset given their relatively distinct genetic variation.

Note S4: Adaptive Introgression

We briefly consider if adaptive archaic introgression has played a role in mediating genetic adaptation to micronutrients by determining which top-ranking SNPs fall in regions previously inferred as introgressed from Neandertal and Denisovan[28,29]. Whilst archaic introgression can result in genetic signatures falsely interpreted as those of local adaptation, the allele frequency of top-ranking SNPs in all non-African populations is higher than what would be expected under neutral archaic introgression (all top-ranking SNPs in non-African populations identified by F_{ST} have allele frequency > 10%; 99.47% of top-ranking SNPs in non-African populations identified by Relate have allele frequency > 10%). Hence, we propose that any archaic introgression within the MA-gene set would be adaptive in nature.

We suggest that two extreme candidate MA-genes have evidence of archaic introgression. First, the magnesium-associated *MECOM*: candidate SNPs (empirical p-value < 0.1%) of this gene in the Middle Eastern Mozabite and Central-South Asian Hazara populations fall in inferred regions of adaptive archaic introgression from Neanderthals[28,29]. Second, the zinc-associated *SLC30A9:* a previous study inferred adaptive alleles in East Asians to be introgressed from Denisovans[30] (which we do not identify with our limited analysis of archaic introgression using introgressed tracts from [28,29]).

Extreme candidate SNPs in these two genes (empirical p-value < $4.65e^{-6}$) are identified using Relate in populations of the same geographic region (**Table 2**) as those with evidence of archaic introgression as referenced above, indicating a potential role of archaic introgression in mediating dietary micronutrient adaptation.

Note S5: Inferring Age of Positive Selection

We used CLUES2[31] to infer the timing of selection on four iron-associated and five calcium-associated genes to address the hypothesis that recent changes to the diet (*i.e.,* those

surrounding the Neolithic transition) drove putative iron and calcium-associated adaptation. Due to computational constraints, we only carry out this analysis on 19 candidate SNPs (those in the 0.1% empirical tail of either the $\mathrm{F_{ST}}$ or $Relate$) across the nine iron- and calcium-associated genes. The chosen candidate SNPs all have strong evidence of positive selection in one or more populations as identified by either $\mathrm{F_{ST}}$ or $Relate$ (see **Table S6**) and, in some cases, lie within regions of the respective MA-gene with a high density of signatures of positive selection (and we hence consider them representative of those regions). The candidate SNPs referenced here (see **Table S6**) also include the top candidate SNPs in HIF1A, FTMT and ATPB2 which bypass our most stringent threshold; $\mathrm{p-value} \leq 4.65\mathrm{e}^{-6}$.

For all 19 of the candidate SNPs, and for each population, we convert the tree sequences inferred by Relate[16] to marginal trees before directly using CLUES2 to infer the evidence of positive selection at the four timepoints[31]. We note that this is only done for unfixed SNPs with $\mathrm{DAF} > 0.2$. Signatures of positive selection were identified in SNPs with $\mathrm{p} \leq 0.001$ and a cut-off of $\mathrm{p} \leq 1\mathrm{e}^{-10}$ was used to identify the SNPs with the strongest evidence of positive selection (**Fig 2**). Across all candidate iron- and calcium-associated genes, we isolate the calcium-associated *ATP2B4* and iron-associated *HIF1A* genes as those with strong evidence for having undergone recent positive selection (~14 kya) and as candidates for adaptation to the changes surrounding the emergence of the Neolithic diet (**Figs S36-44**).

Figures

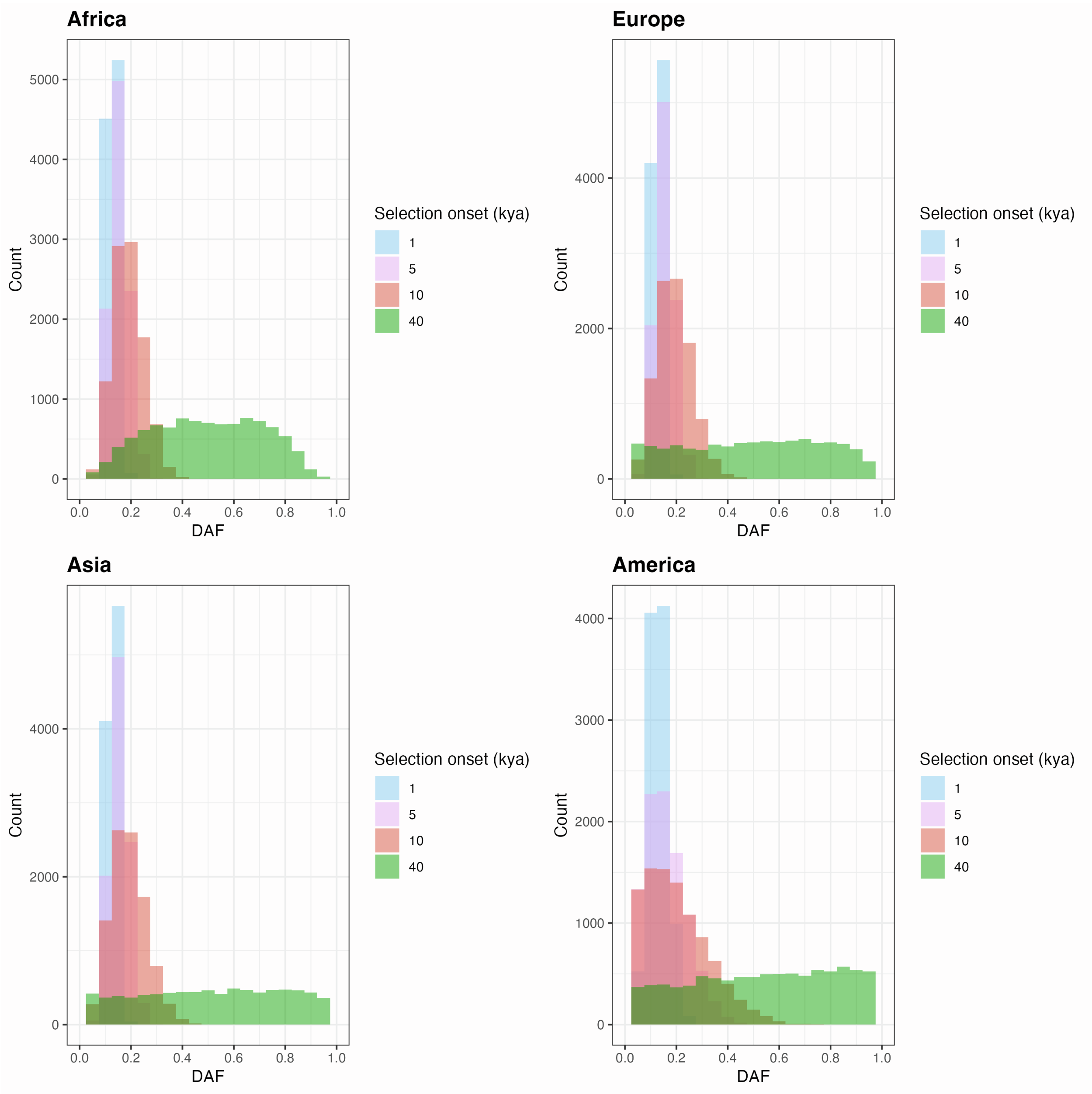

Figure S1: Distribution of derived allele frequency of selected alleles. Distribution of the derived allele frequency (DAF) of selected SNPs (y-axis) when the selection onset is 1 kya, 5 kya, 10 kya or 40 kya (indicated by legend) in four simulated populations (see **Fig. 1**).

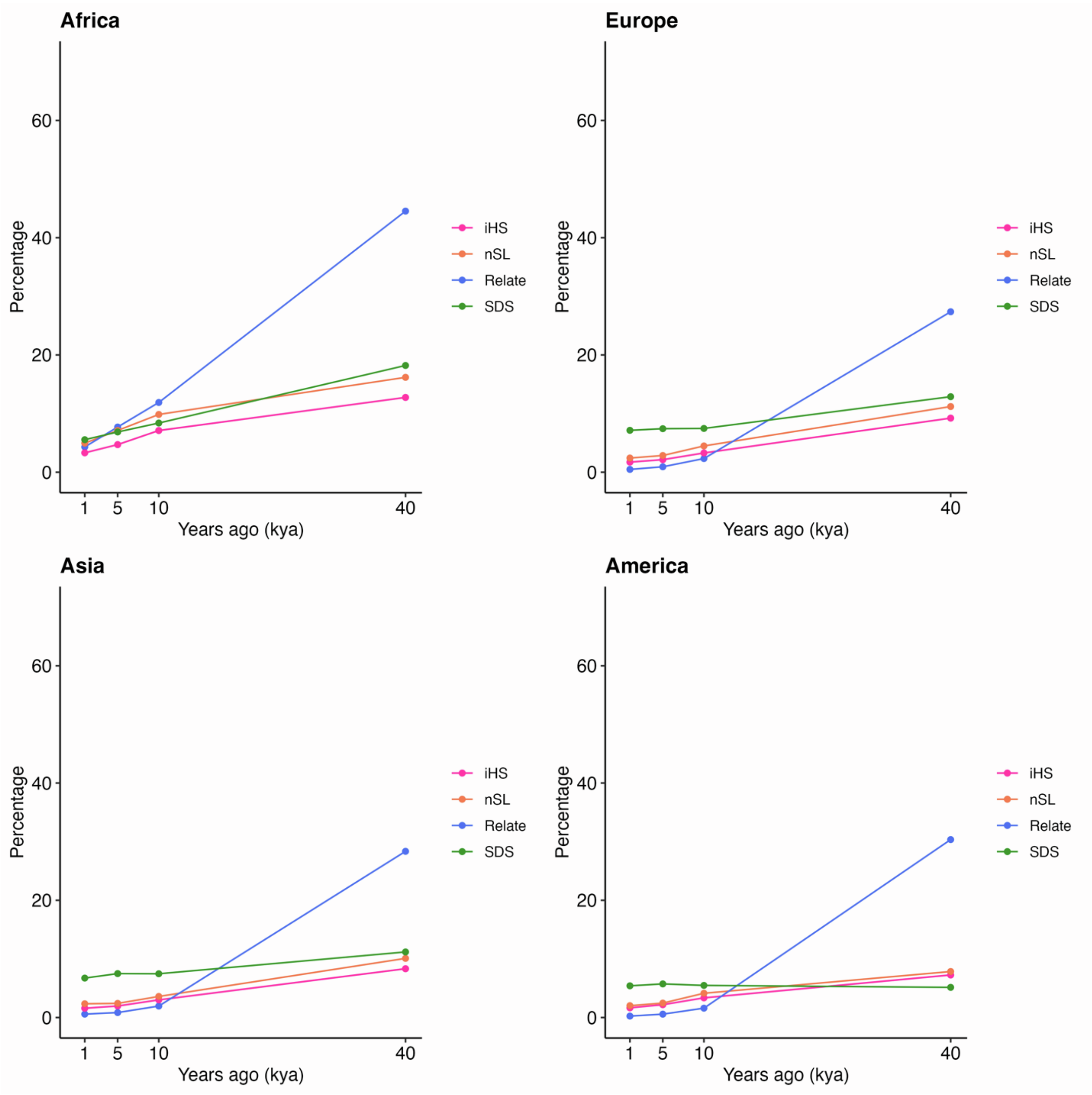

Figure S2: Percentage of selected SNPs identified as under positive selection. Percentage of selected SNPs identified as under positive selection (according to the tail of the neutral distribution; y-axis) at four different timepoints (x-axis) for methods used on focal populations. Shown for four simulated populations (see **Fig. 1**).

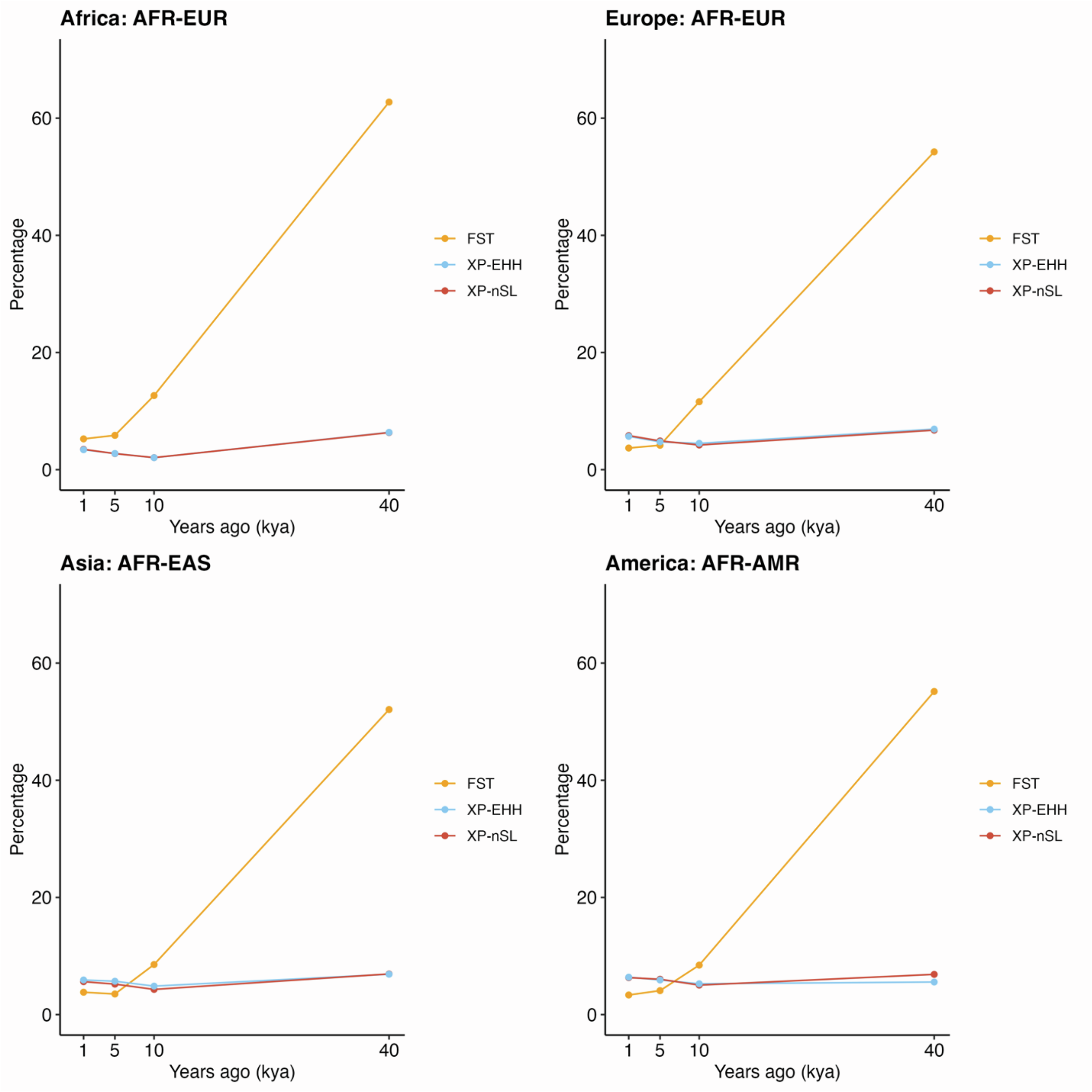

Figure S3: Percentage of selected SNPs identified as under positive selection for cross-population statistics. Percentage of selected SNPs identified as under positive selection (according to the tail of the neutral distribution; y-axis) at four different timepoints (x-axis) for cross-population methods. Shown for four simulated populations (see **Fig. 1**), with cross-population comparisons indicated.

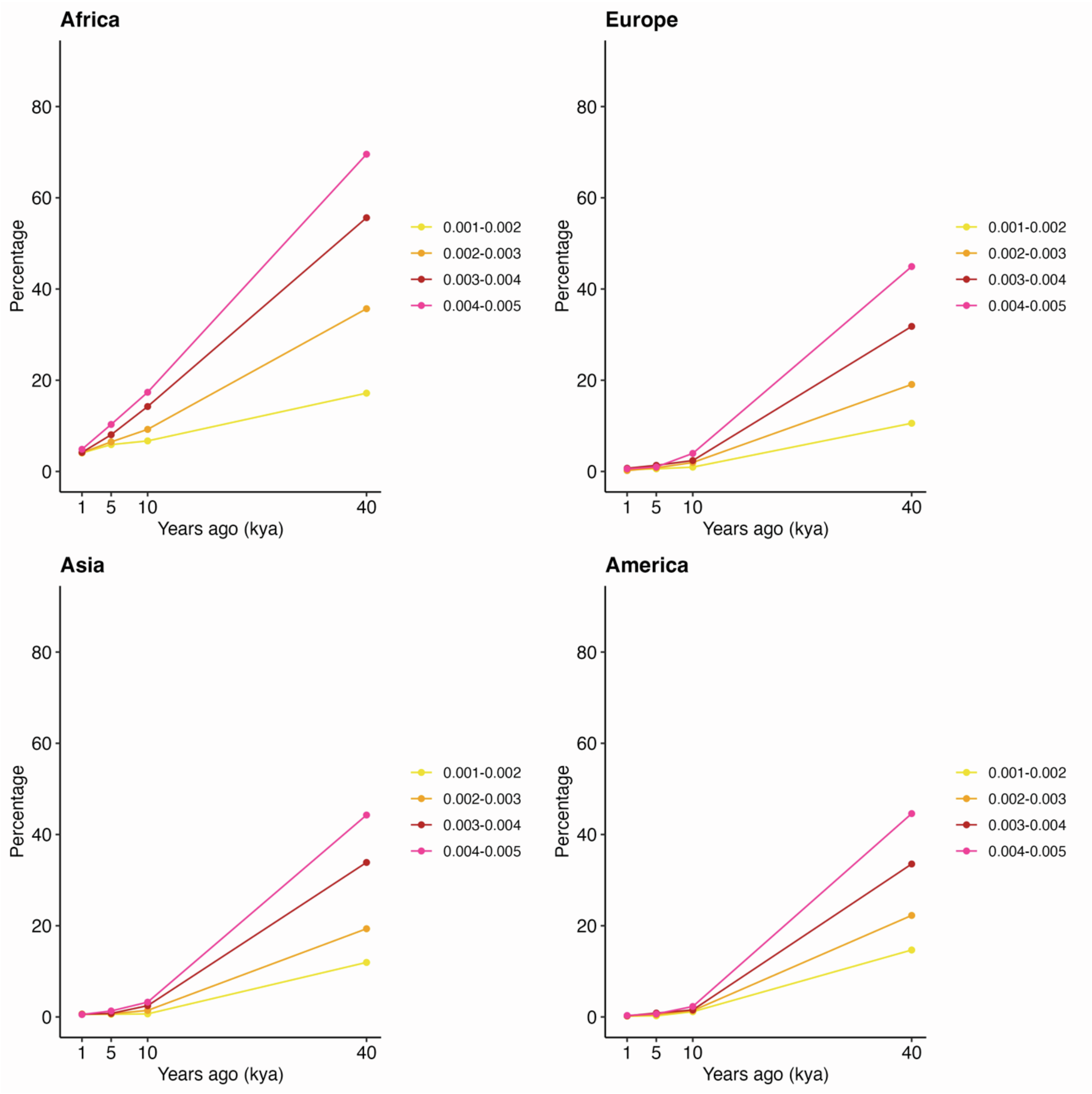

Figure S4: Percentage of selected SNPs identified as under positive selection by Relate by selection coefficient. Percentage of selected SNPs identified as under positive selection by Relate (according to the tail of the neutral distribution; y-axis) at four different timepoints (x-axis) as broken down by the simulated selection coefficient (see legend). Shown for four simulated populations (see **Fig. 1**).

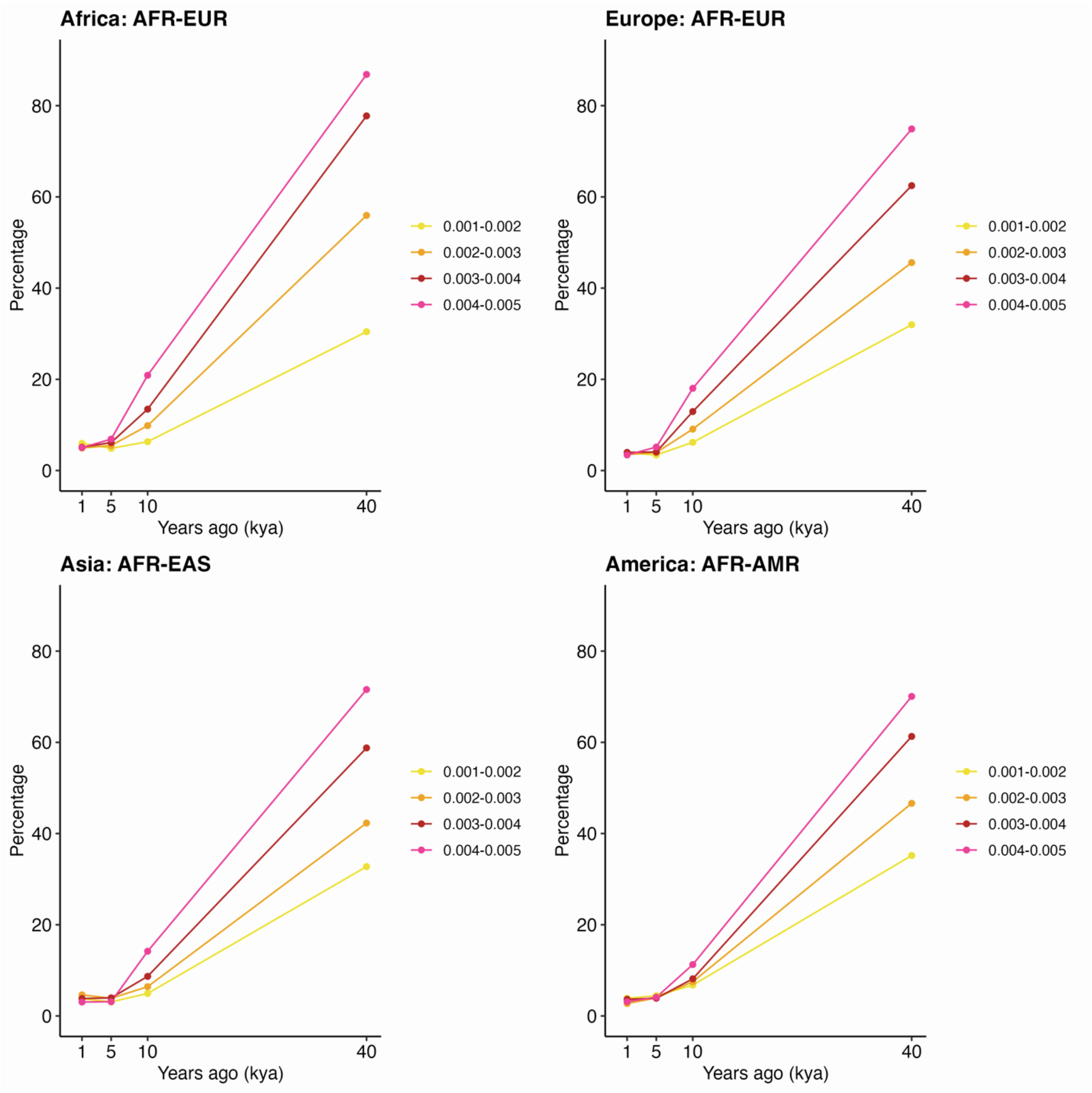

Figure S5: Percentage of selected SNPs identified as under positive selection by F_{ST} by selection coefficient. Percentage of selected SNPs identified as under positive selection by F_{ST} (according to the tail of the neutral distribution; y-axis) at four different timepoints (x-axis) as broken down by the simulated selection coefficient (see legend). Shown for four simulated populations (see **Fig. 1**), with cross-population comparisons indicated.

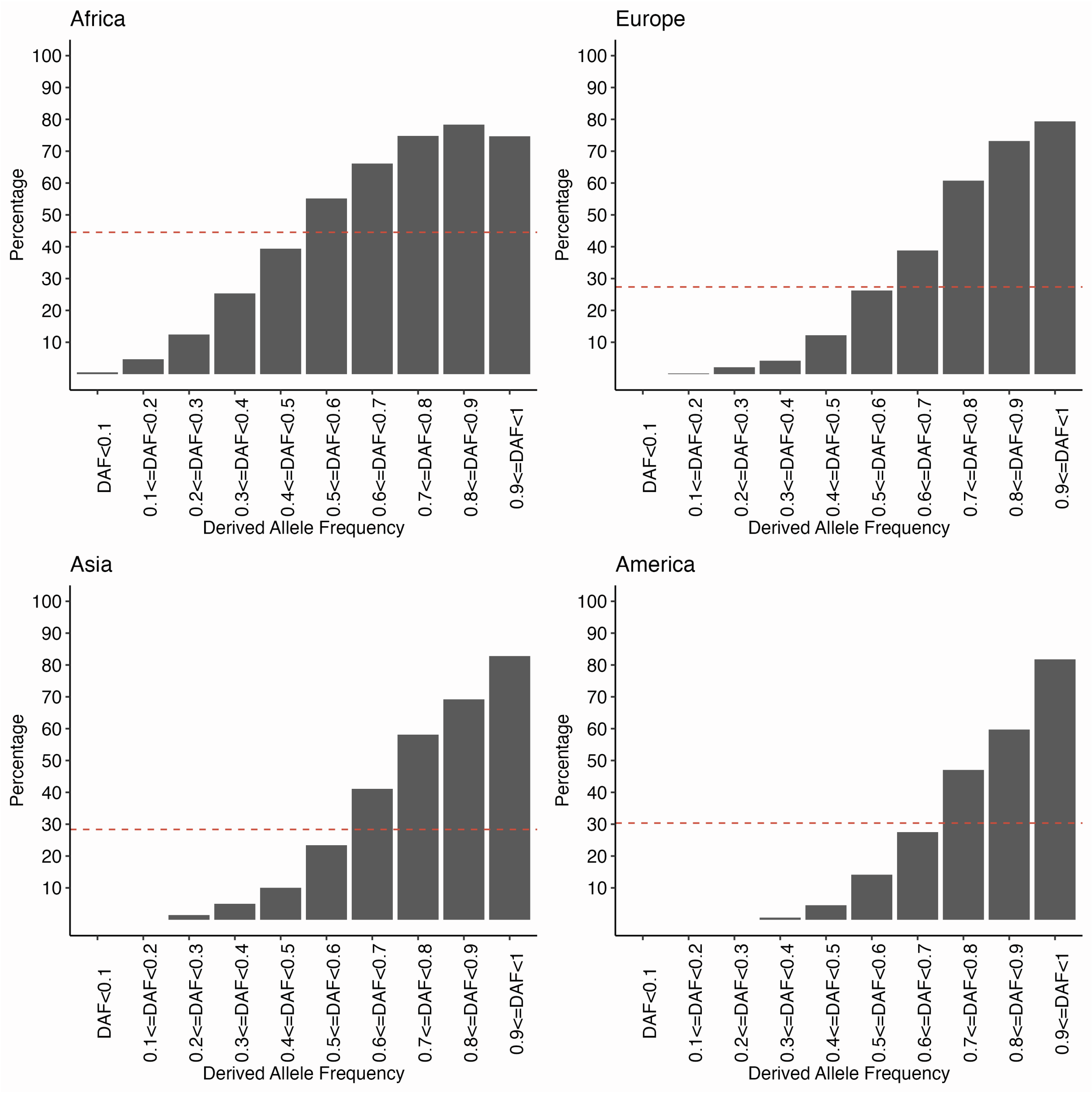

Figure S6: Percentage of selected SNPs identified as under positive selection by Relate by derived allele frequency. Percentage of selected SNPs identified as under positive selection by Relate (according to the tail of the neutral distribution; y-axis) as broken down by derived allele frequency (DAF; x-axis). Dashed line indicates the percentage of selected SNPs identified as under positive selection for all DAF values. Shown for positive selection initiated at 40 kya for four simulated populations (see **Fig. 1**).

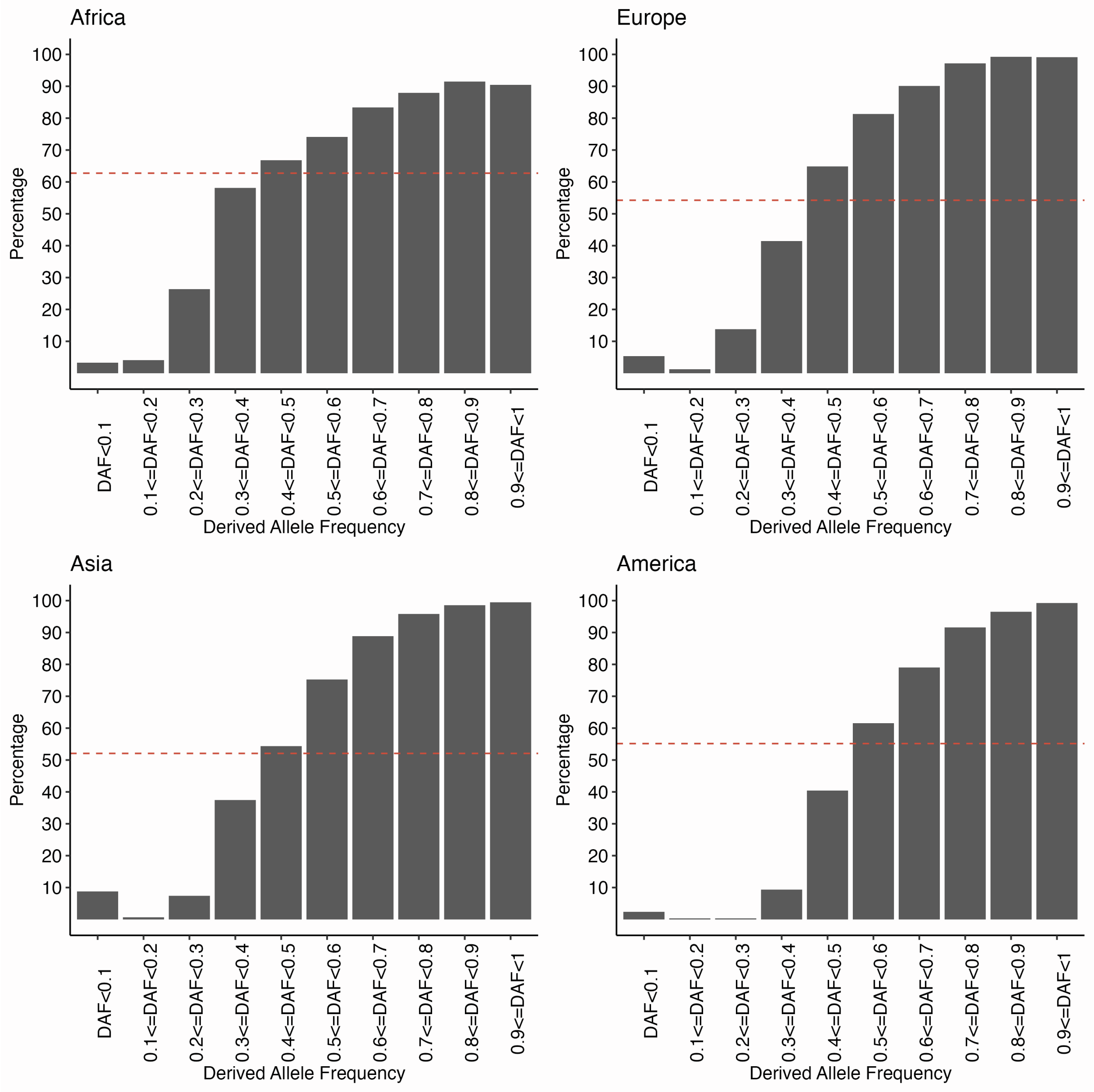

Figure S7: Percentage of selected SNPs identified as under positive selection by F_{ST} by derived allele frequency. Percentage of selected SNPs identified as under positive selection by F_{ST} (according to the tail of the neutral distribution; y-axis) as broken down by derived allele frequency (DAF; x-axis). Dashed line indicates the percentage of selected SNPs identified as under positive selection for all DAF values. Shown for positive selection initiated at 40 kya for four simulated populations (see **Fig. 1**), with cross-population comparisons indicated.

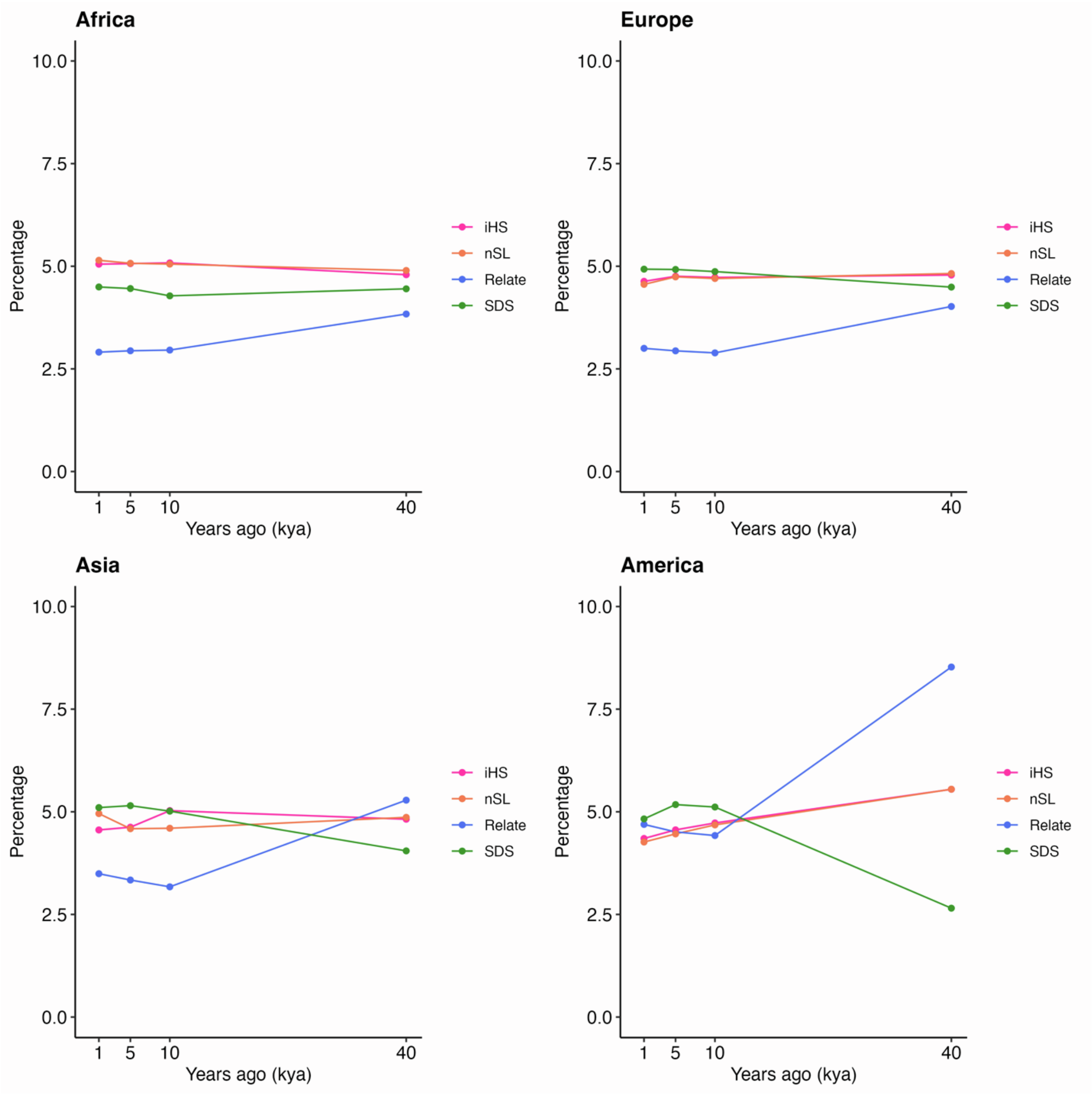

Figure S8: Percentage of non-selected SNPs identified as under positive selection. Percentage of non-selected SNPs identified as under positive selection (according to the tail of the neutral distribution; y-axis) at four different timepoints (x-axis) for methods used on focal populations. Shown for four simulated populations (see **Fig. 1**).

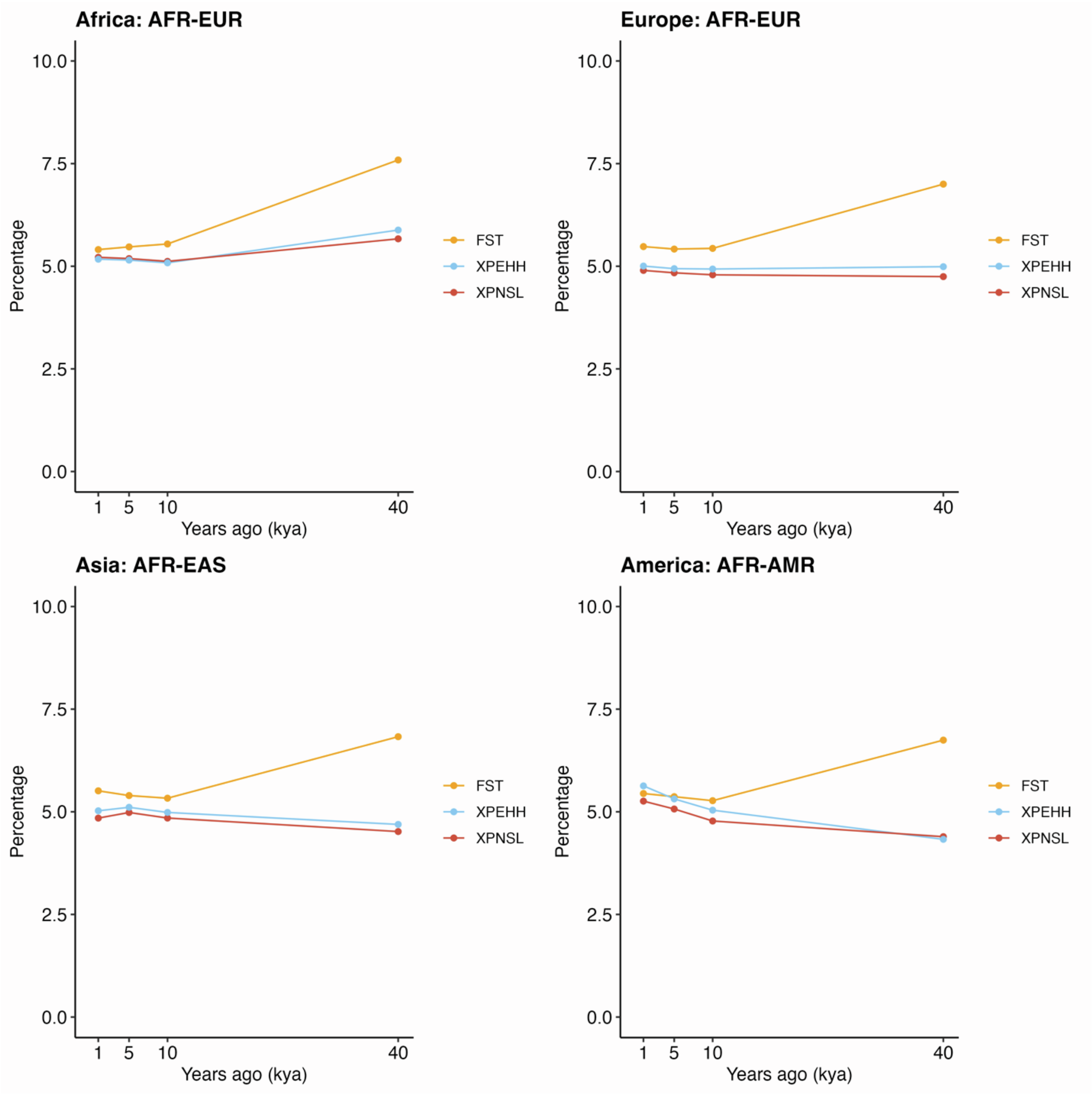

Figure S9: Percentage of non-selected SNPs identified as under positive selection for cross-population methods. Percentage of non-selected SNPs identified as under positive selection (according to the tail of the neutral distribution; y-axis) at four different timepoints (x-axis) for cross-population methods. Shown for four simulated populations (see **Fig. 1**), with cross-population comparisons indicated.

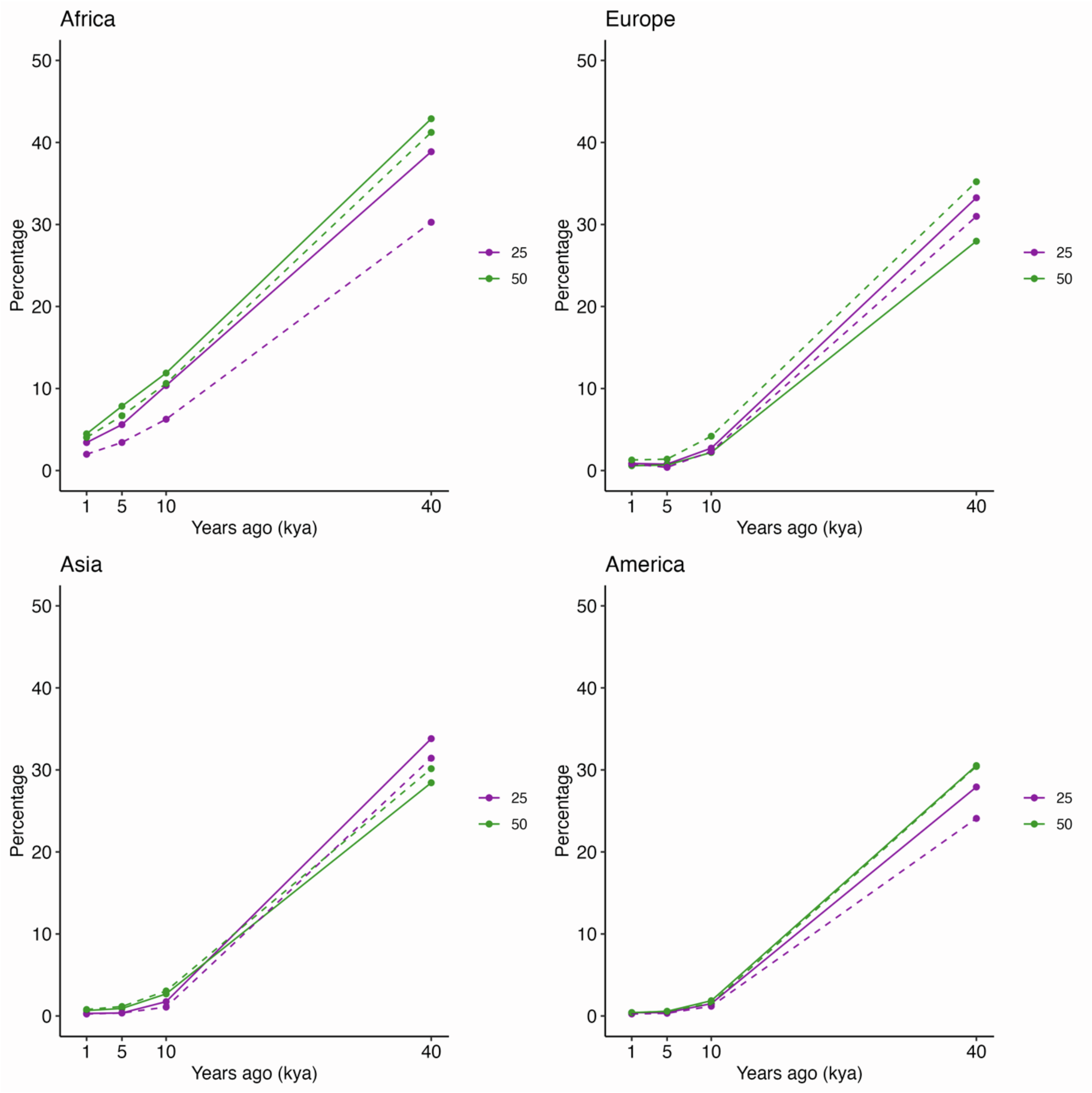

Figure S10: Percentage of selected SNPs identified as under positive selection by differing application of Relate. Percentage of selected SNPs identified as under positive selection (y-axis) by Relate according to either the tail of the neutral distribution (solid lines) or by the raw output of Relate ($-\log10p - value$; dashed lines) at four different timepoints (x-axis) and for sample sizes of 50 (green) or 25 (purple) simulated individuals. Shown for four simulated populations (see **Fig. 1**)

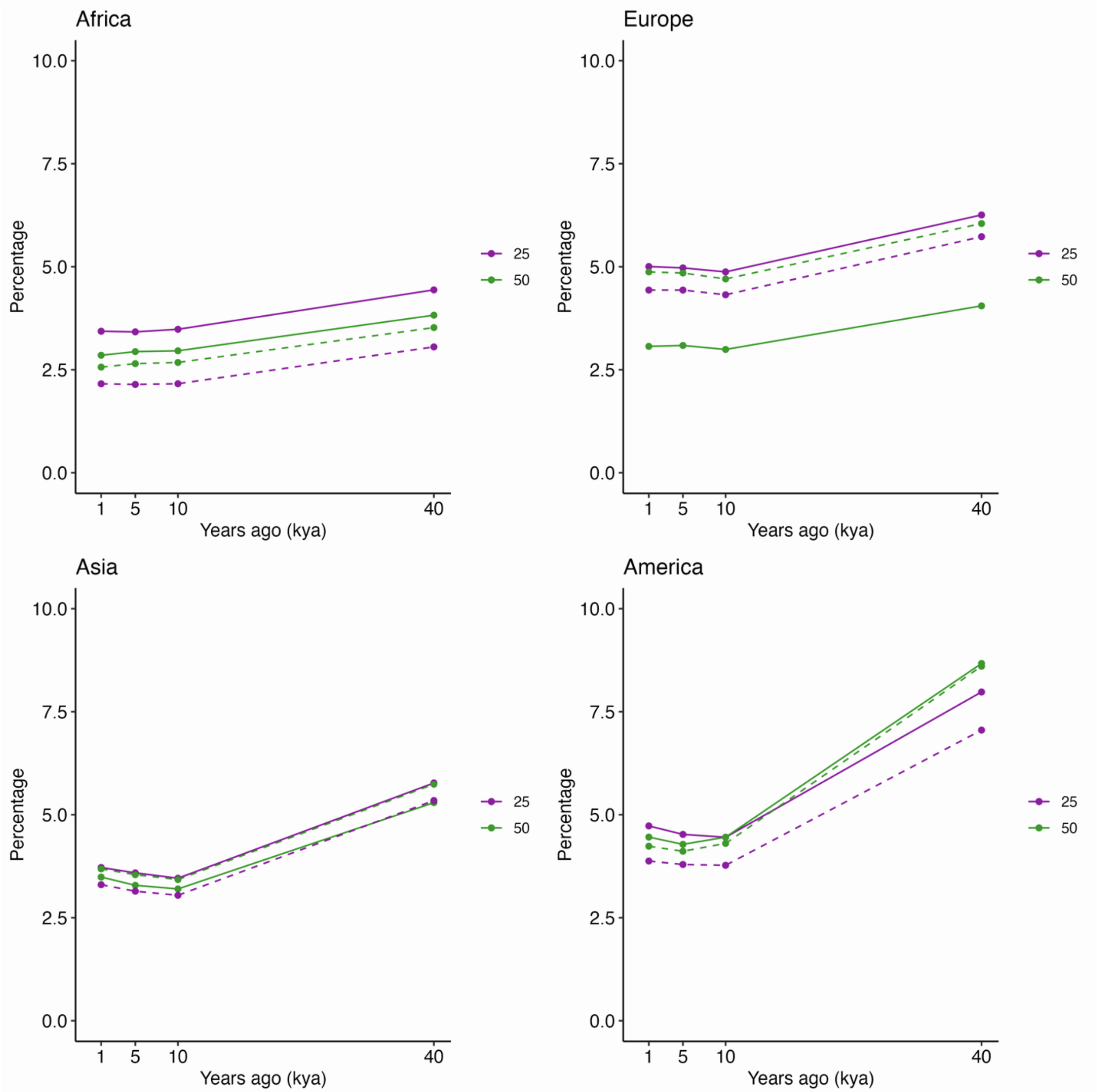

Figure S11: Percentage of non-selected SNPs identified as under positive selection by differing application of Relate. Percentage of non-selected SNPs identified as under positive selection (y-axis) by Relate according to either the tail of the neutral distribution (solid lines) or by the raw output of Relate ($-\log10\text{p} - \text{value}$; dashed lines) at four different timepoints (x-axis) and for sample sizes of 50 (green) or 25 (purple) simulated individuals. Shown for four simulated populations (see **Fig. 1**)

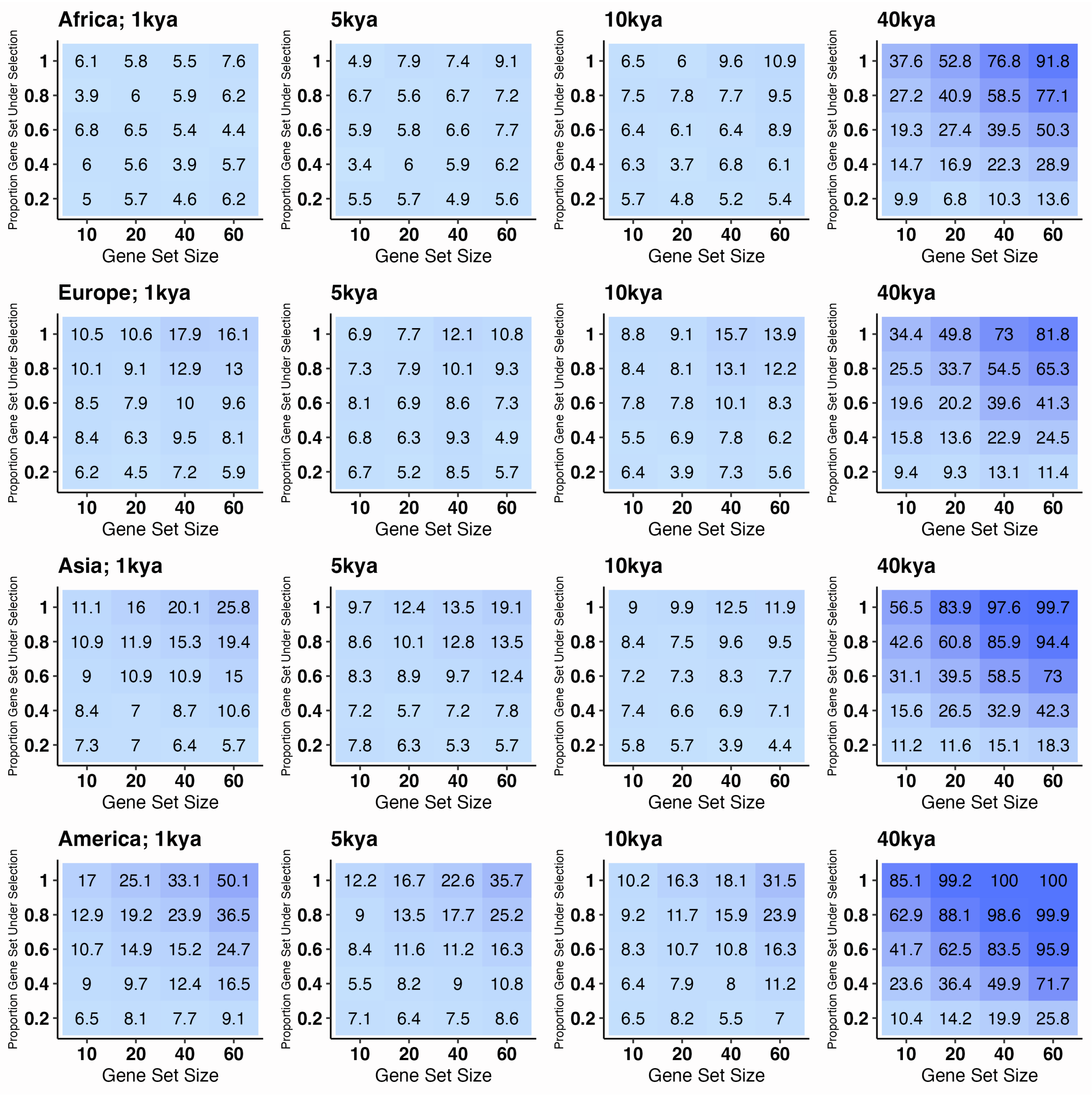

Figure S12: Percentage of selected gene sets identified as under positive selection by Relate. Percentage of selected gene sets identified as under positive selection by Relate (according to the tail of the neutral distribution; numbers inside matrix) for gene sets that vary by size (x-axis) and by proportion of gene regions under positive selection (y-axis). Shown for positive selection simulated in four simulated populations (rows; see **Fig. 1**) at four timepoints (columns).

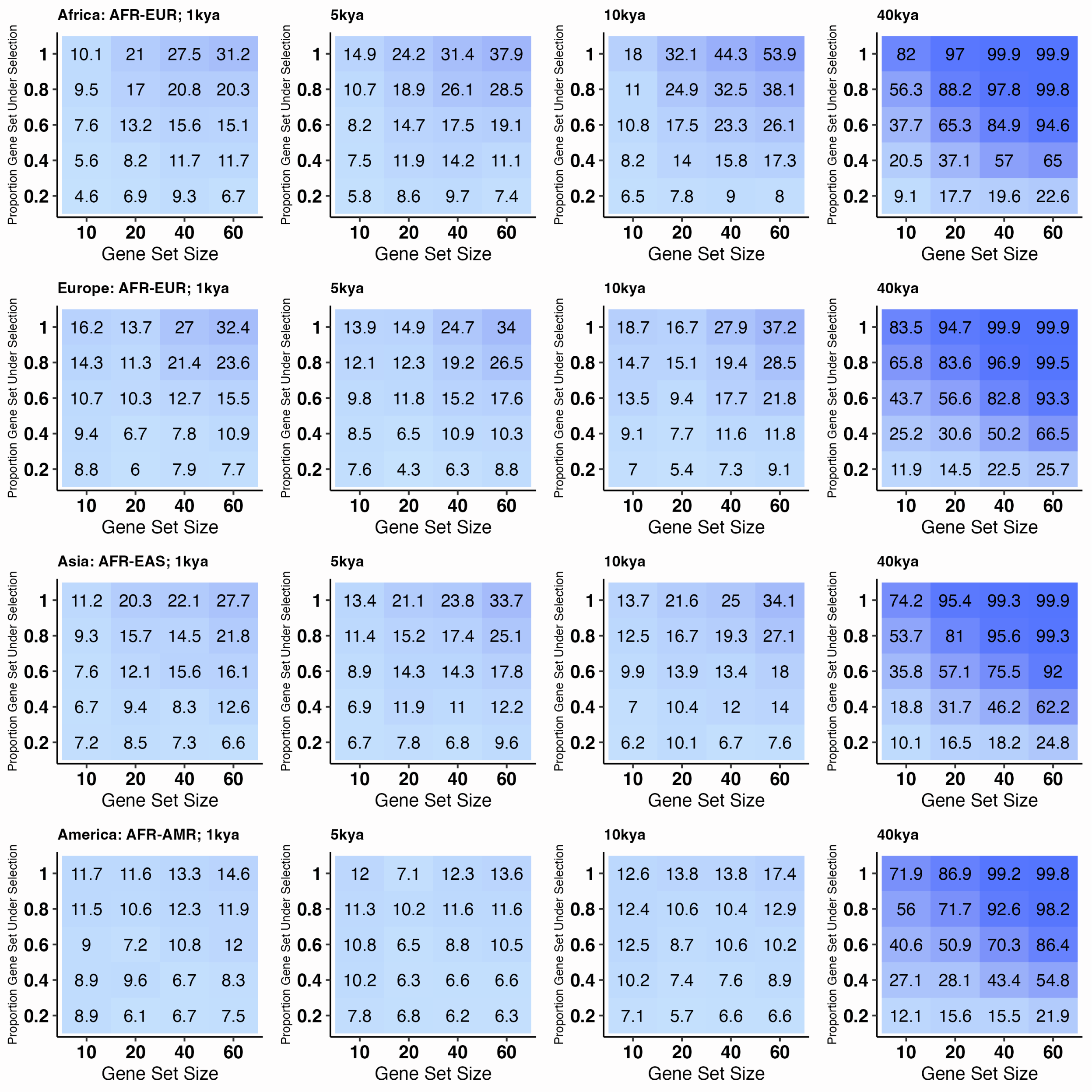

Figure S13: Percentage of selected gene sets identified as under positive selection by F_{ST}. Percentage of selected gene sets identified as under positive selection by F_{ST} (according to the tail of the neutral distribution; numbers inside matrix) for gene sets that vary by size (x-axis) and by proportion of gene regions under positive selection (y-axis). Shown for positive selection simulated in four simulated populations, with cross-population comparisons indicated (rows; see **Fig. 1**), at four timepoints (columns).

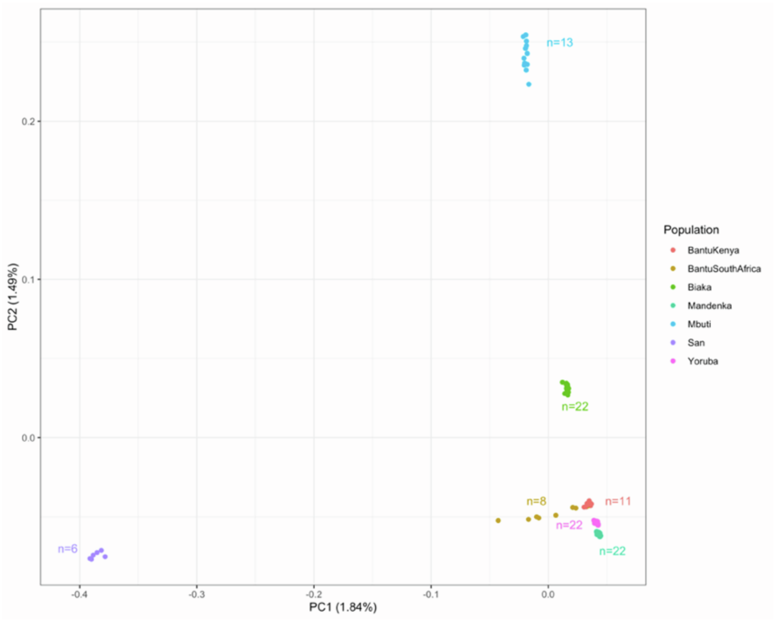

Figure S14: Principal component analysis of African individuals. Principal component analysis of African individuals from[2], showing PC1 and PC2.

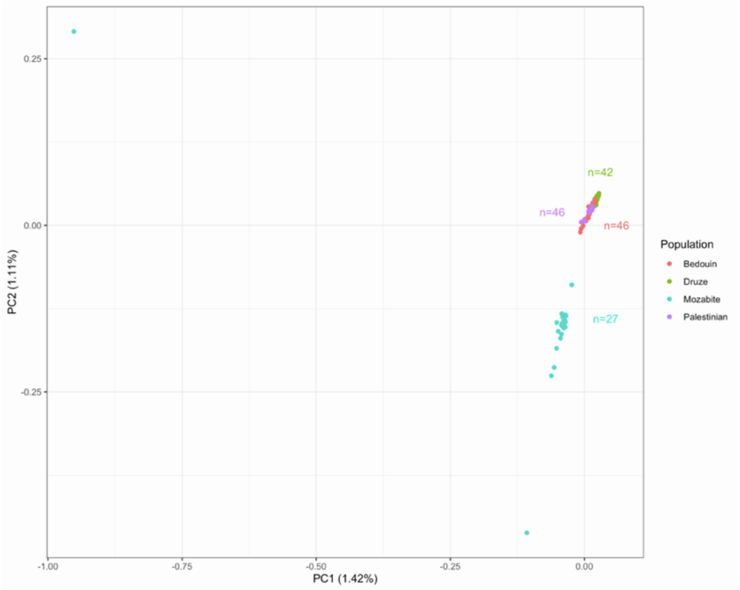

Figure S15: Principal component analysis of Middle-Eastern individuals. Principal component analysis of Middle-eastern individuals from[2], showing PC1 and PC2, having removed outlier individuals

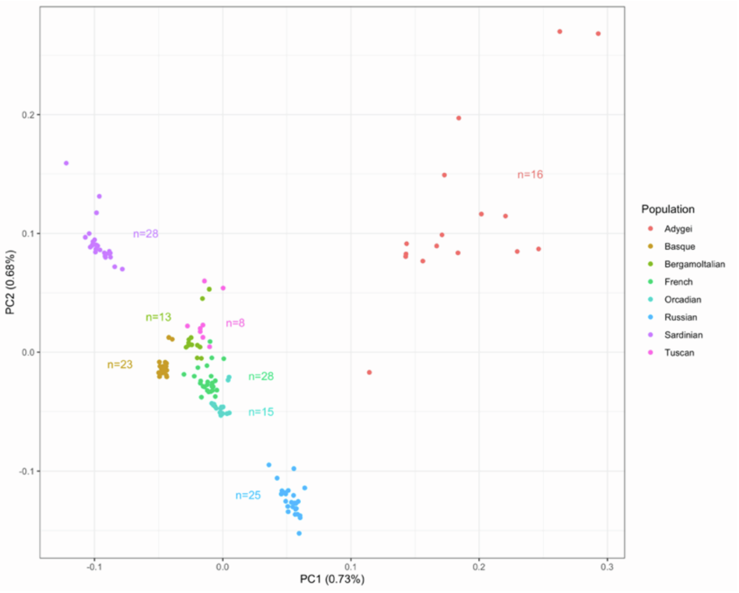

Figure S16: Principal component analysis of European individuals. Principal component analysis of European individuals from[2], showing PC1 and PC2, having removed outlier individuals.

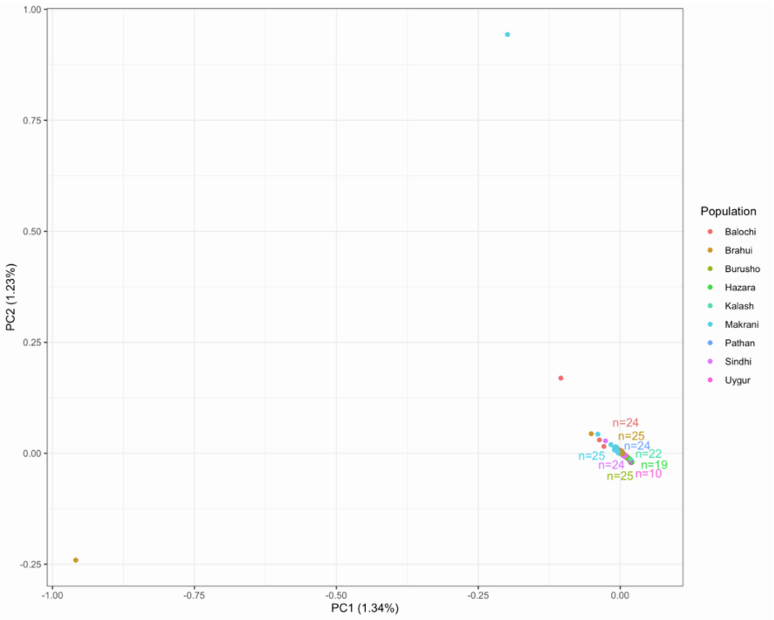

Figure S17: Principal component analysis of Central-South Asian individuals. Principal component analysis of Central-South Asian individuals from[2], showing PC1 and PC2, having removed outlier individuals

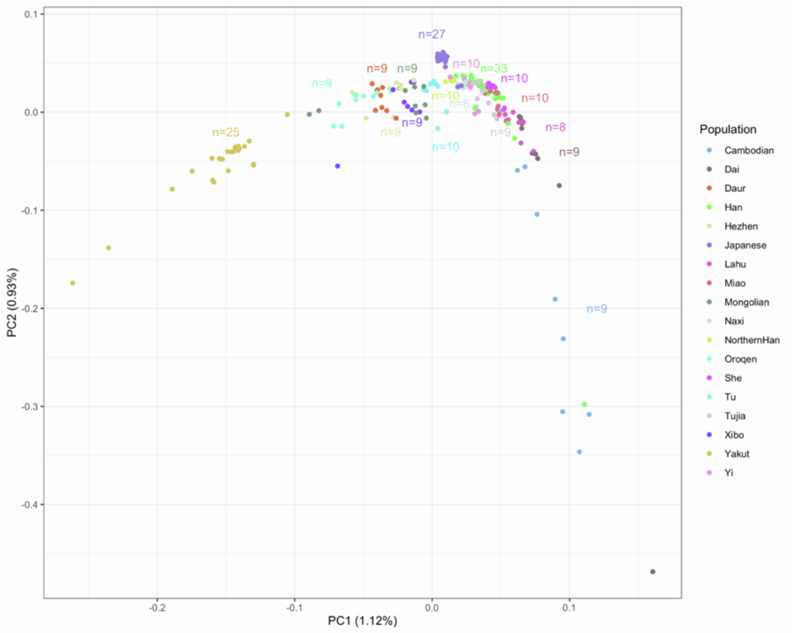

Figure S18: Principal component analysis of East Asian individuals. Principal component analysis of East Asian individuals from[2], showing PC1 and PC2

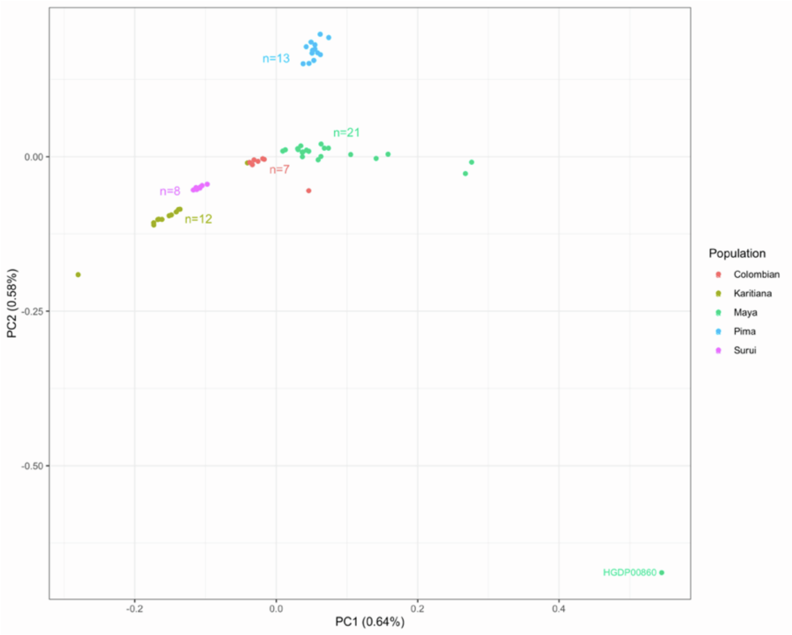

Figure S19: Principal component analysis of American individuals. Principal component analysis of American individuals from[2], showing PC1 and PC2, having removed outlier individuals

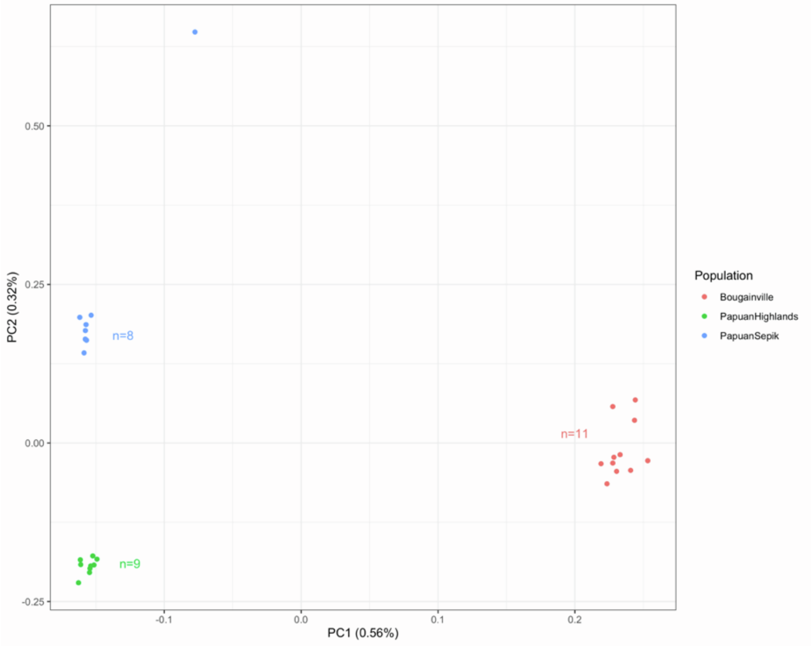

Figure S20: Principal component analysis of Oceanian individuals. Principal component analysis of Oceanic individuals from[2], showing PC1 and PC2

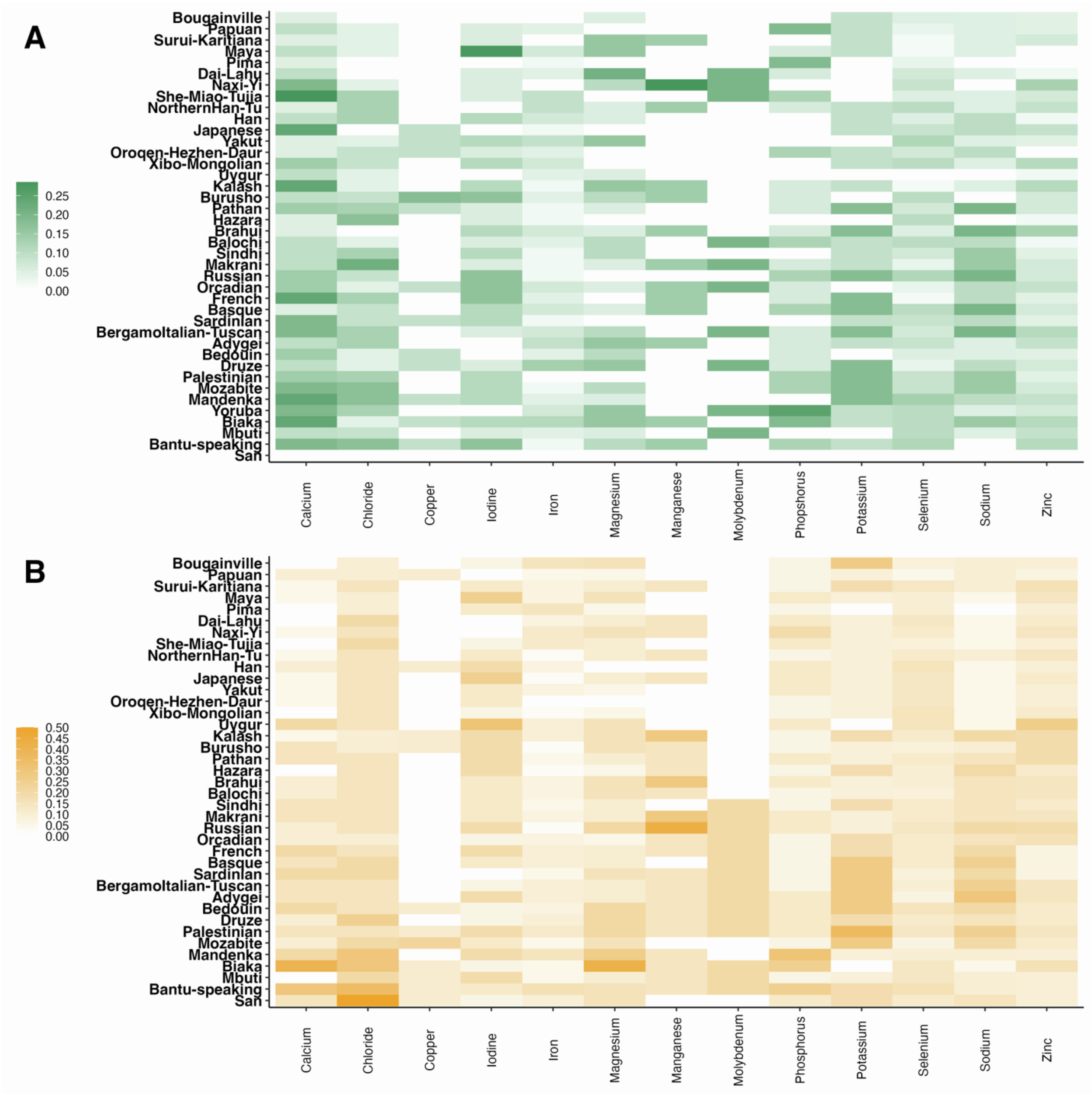

Fig. S21: Proportion of micronutrient gene sets with signatures of positive selection. A and B show the proportion of each micronutrient gene set (including overlap between sets) that have signatures in the 0.1% tail for Relate and F_{ST} selection values, respectively. Key shows the proportion of genes within a gene set with such signatures.

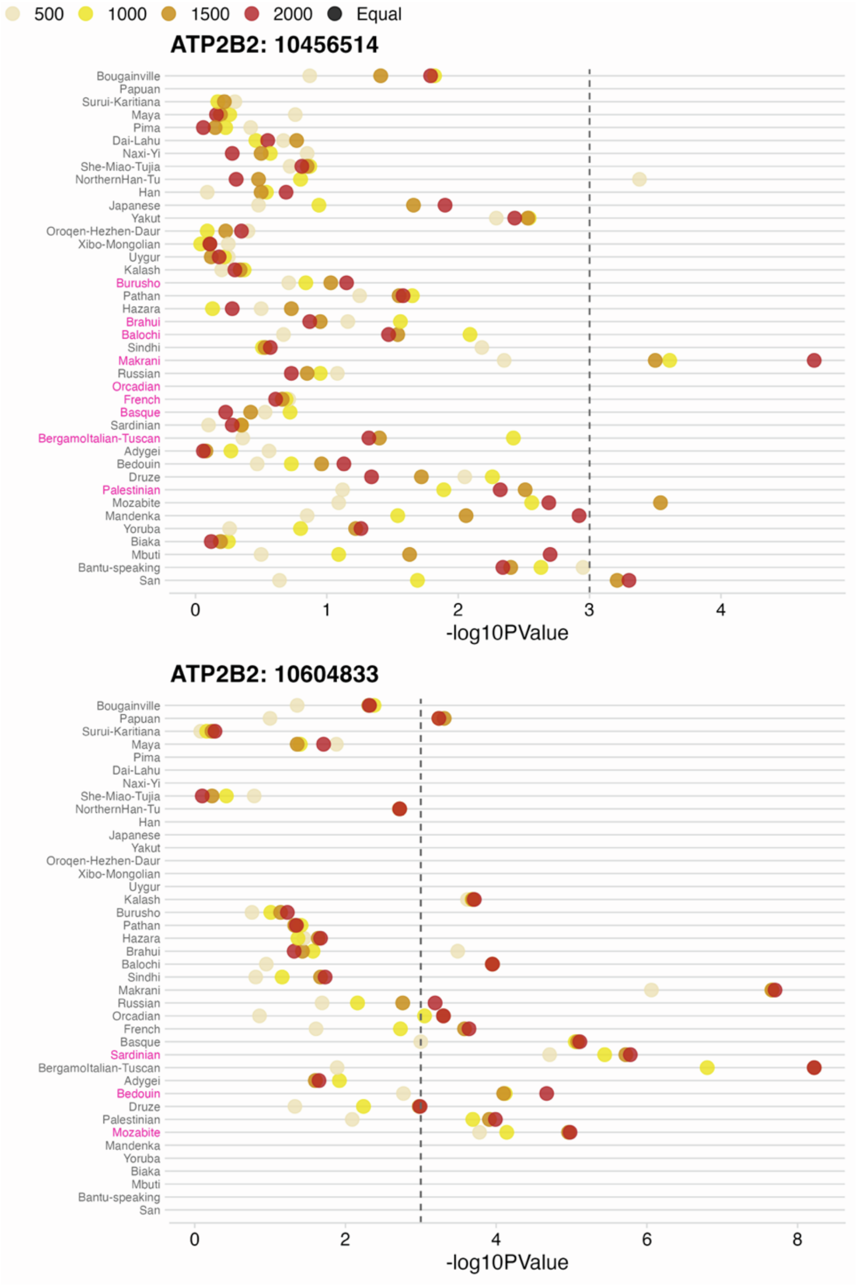

Fig. S22: Evidence for positive selection as inferred by CLUES2 for candidate SNPs in calcium-associated *ATP2B2*. Evidence for positive selection as inferred by CLUES2 for candidate SNPs (positions given) in calcium-associated *ATP2B2* (see **Supp. Note 5** and **Table S6**) across four generational timepoints (see top legend). Dashed vertical line indicates $p = 0.001$. Black points indicate that the evidence for selection is equal across all four tested timepoints. Populations in pink are those with signatures of positive selection ($p \leq 0.001$) identified by Relate or F_{ST}.

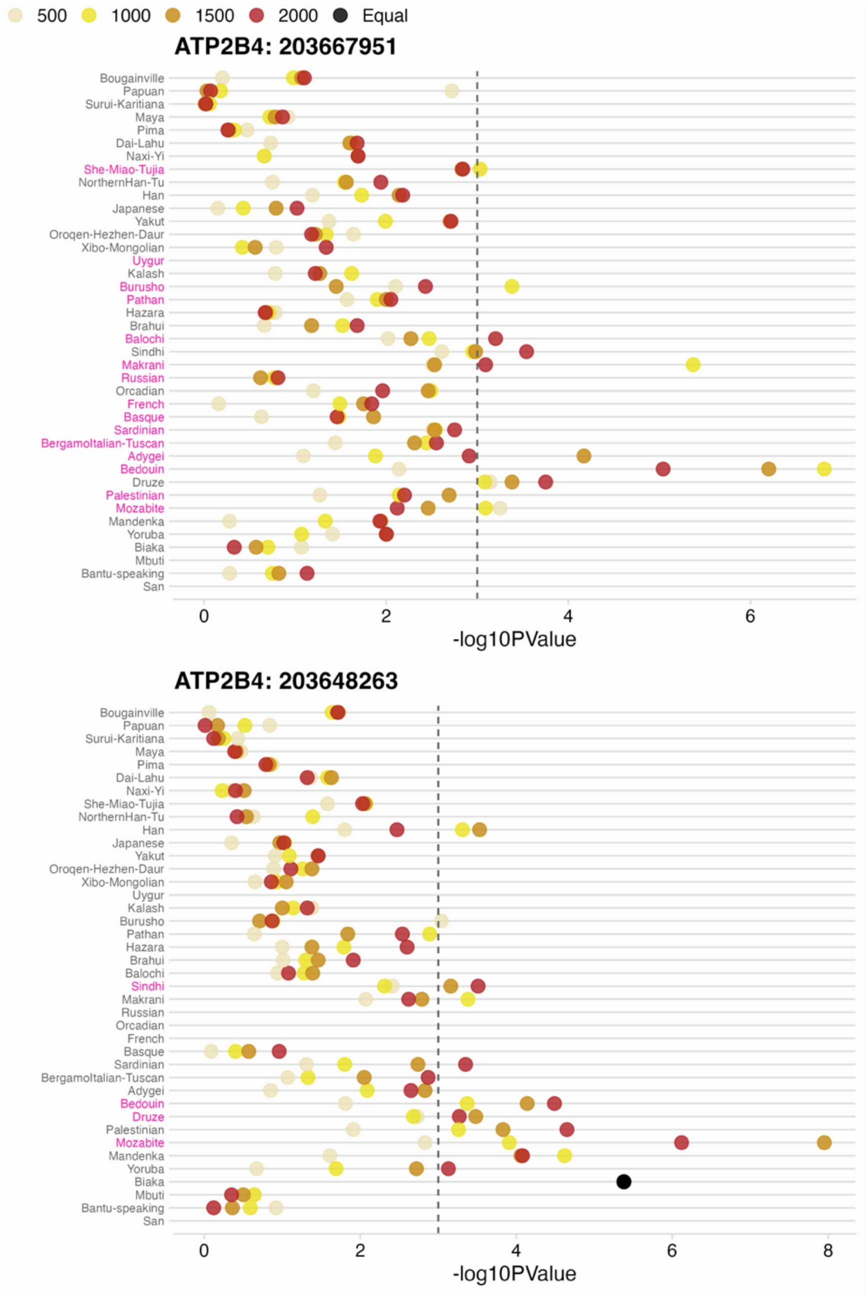

Fig. S23: Evidence for positive selection as inferred by CLUES2 for candidate SNPs in calcium-associated *ATP2B4*. Evidence for positive selection as inferred by CLUES2 for candidate SNPs (positions given) in calcium-associated *ATP2B4* (see **Supp. Note 5** and **Table S6**) across four generational timepoints (see top legend). Dashed vertical line indicates $p = 0.001$. Black points indicate that the evidence for selection is equal across all four tested timepoints. Populations in pink are those with signatures of positive selection ($p \leq 0.001$) identified by Relate or F_{ST}.

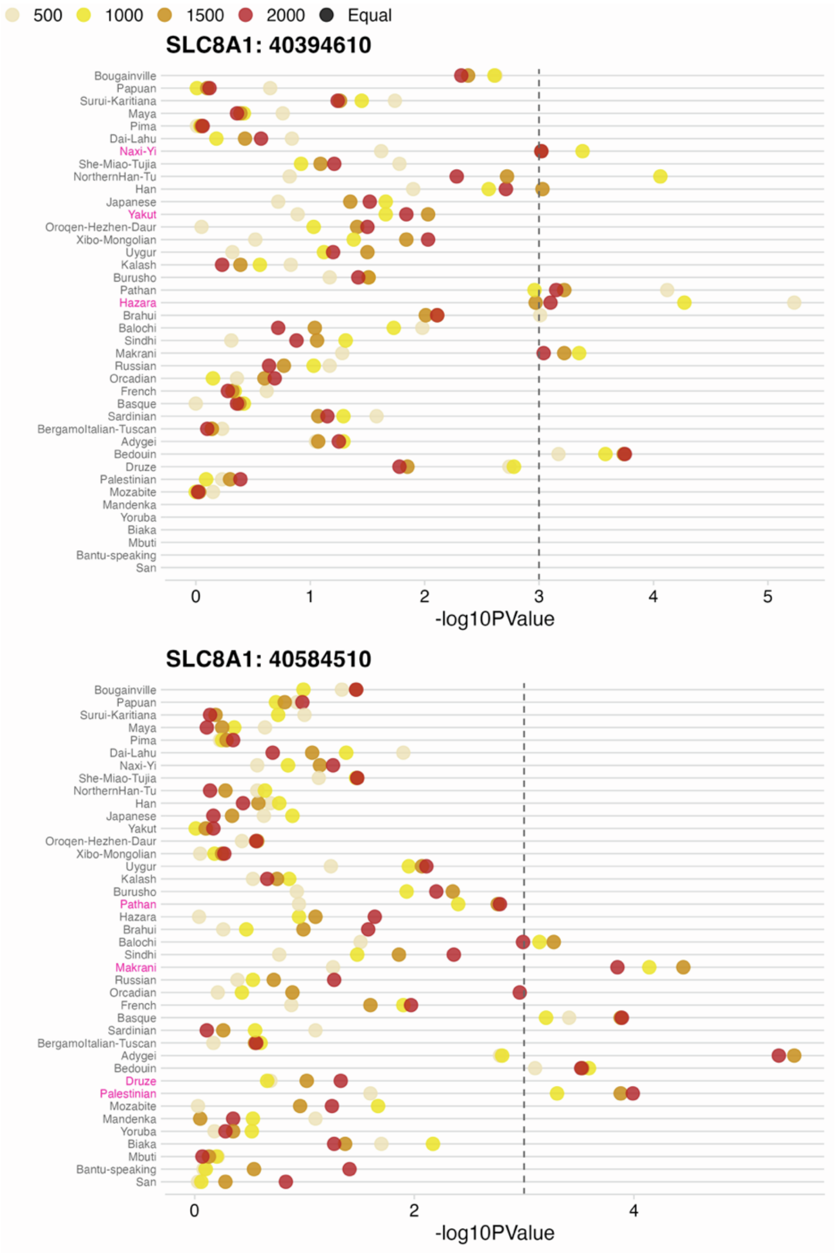

Fig. S24: Evidence for positive selection as inferred by CLUES2 for candidate SNPs in calcium-associated *SLC8A1*. Evidence for positive selection as inferred by CLUES2 for candidate SNPs (positions given) in calcium-associated *SLC8A1* (see **Supp. Note 5** and **Table S6**) across four generational timepoints (see top legend). Dashed vertical line indicates $p = 0.001$. Black points indicate that the evidence for selection is equal across all four tested timepoints. Populations in pink are those with signatures of positive selection ($p \leq 0.001$) identified by Relate or F_{ST}.

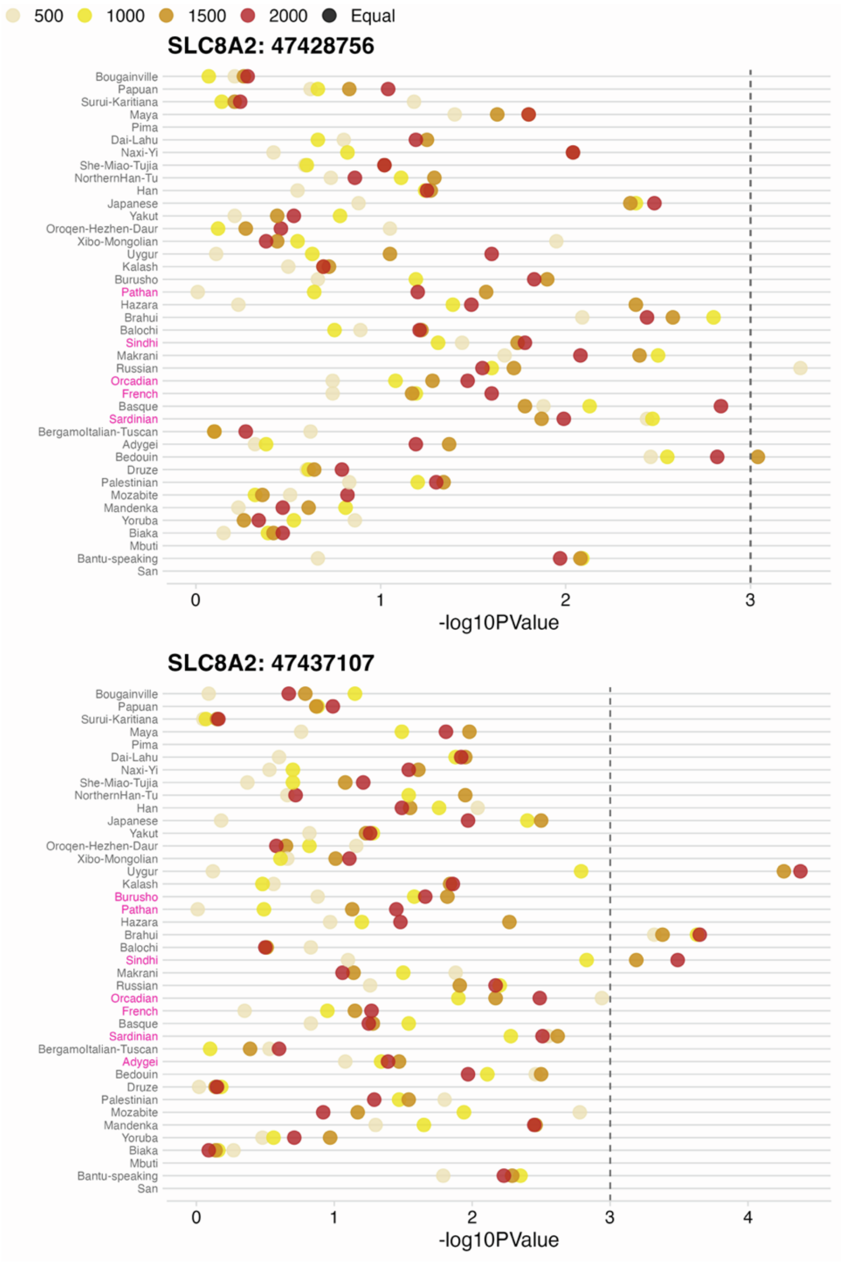

Fig. S25: Evidence for positive selection as inferred by CLUES2 for candidate SNPs in calcium-associated *SLC8A2* Evidence for positive selection as inferred by CLUES2 for candidate SNPs (positions given) in calcium-associated *SLC8A2* (see **Supp. Note 5** and **Table S6**) across four generational timepoints (see top legend). Dashed vertical line indicates $p = 0.001$. Black points indicate that the evidence for selection is equal across all four tested timepoints. Populations in pink are those with signatures of positive selection ($p \leq 0.001$) identified by Relate or F_{ST}.

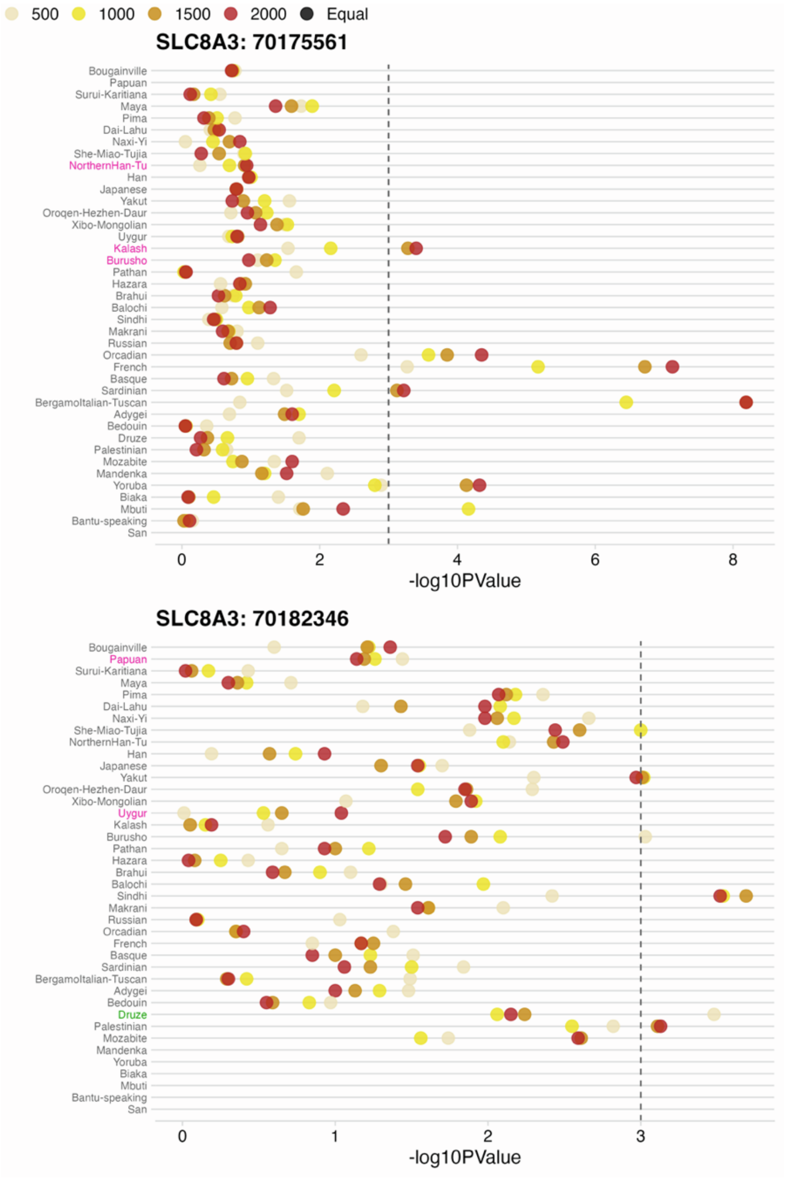

Fig. S26: Evidence for positive selection as inferred by CLUES2 for candidate SNPs in calcium-associated *SLC8A3*. Evidence for positive selection as inferred by CLUES2 for candidate SNPs (positions given) in calcium-associated *SLC8A3* (see **Supp. Note 5** and **Table S6**) across four generational timepoints (see top legend). Dashed vertical line indicates $p = 0.001$. Black points indicate that the evidence for selection is equal across all four tested timepoints. Populations in pink are those with signatures of positive selection ($p \leq 0.001$) identified by Relate or F_{ST}.

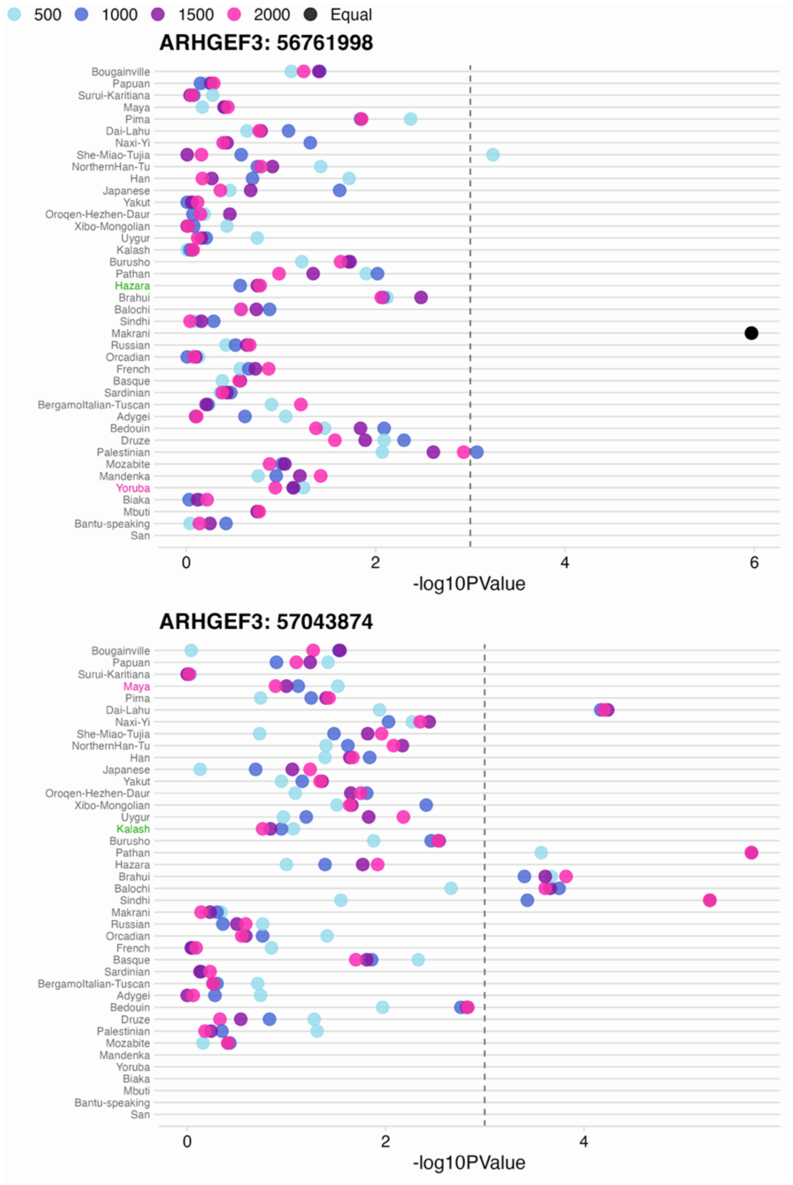

Fig. S27: Evidence for positive selection as inferred by CLUES2 for candidate SNPs in calcium-associated *ARHGEF3*. Evidence for positive selection as inferred by CLUES2 for candidate SNPs (positions given) in iron-associated *ARHGEF3* (see **Supp. Note 5** and **Table S6**) across four generational timepoints (see top legend). Dashed vertical line indicates $p = 0.001$. Black points indicate that the evidence for selection is equal across all four tested timepoints. Populations in pink are those with signatures of positive selection ($p \leq 0.001$) identified by Relate or F_{ST}.

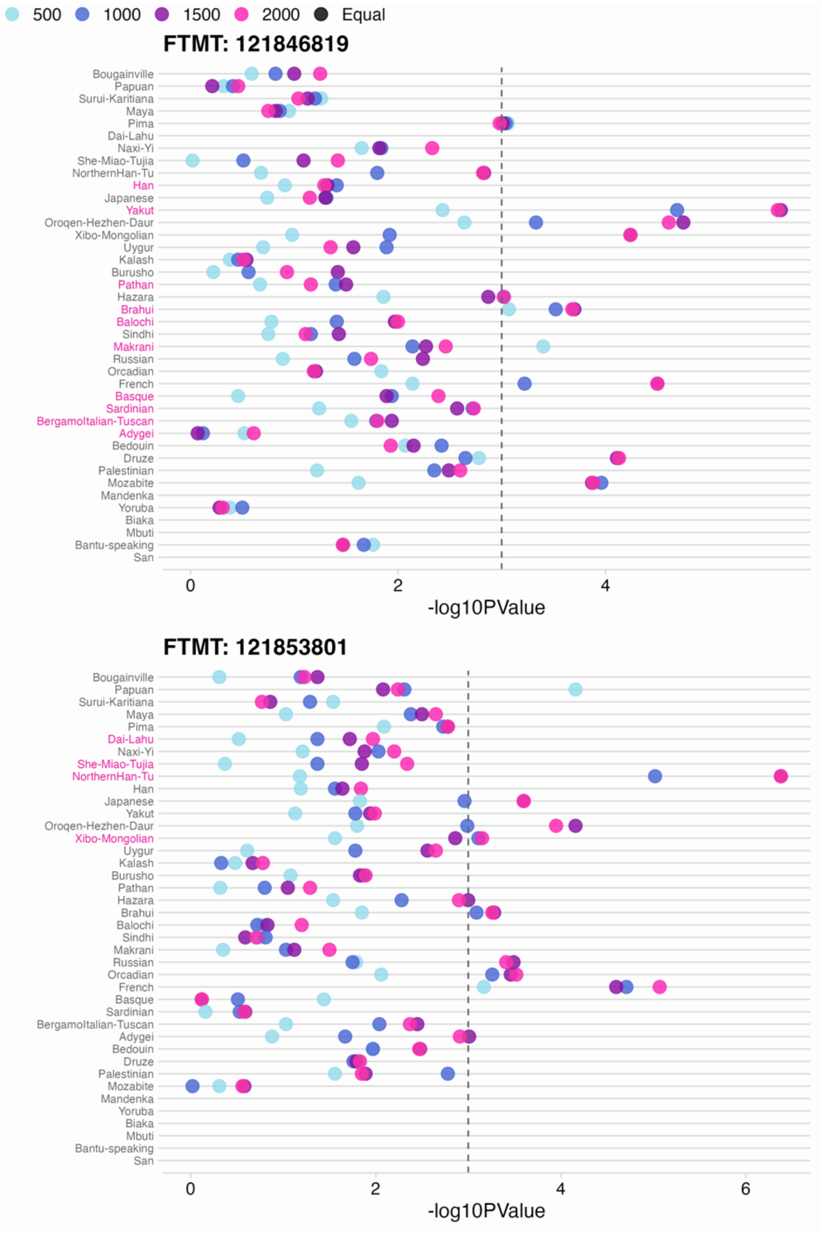

Fig. S28: Evidence for positive selection as inferred by $\mathrm{CLUES2}$ for candidate SNPs in calcium-associated *FTMT*. Evidence for positive selection as inferred by $\mathrm{CLUES2}$ for candidate SNPs (positions given) in iron-associated *FTMT* (see **Supp. Note 5** and **Table S6**) across four generational timepoints (see top legend). Dashed vertical line indicates $\mathrm{p} = 0.001$. Black points indicate that the evidence for selection is equal across all four tested timepoints. Populations in pink are those with signatures of positive selection ($\mathrm{p} \leq 0.001$) identified by Relate or $\mathrm{F_{ST}}$.

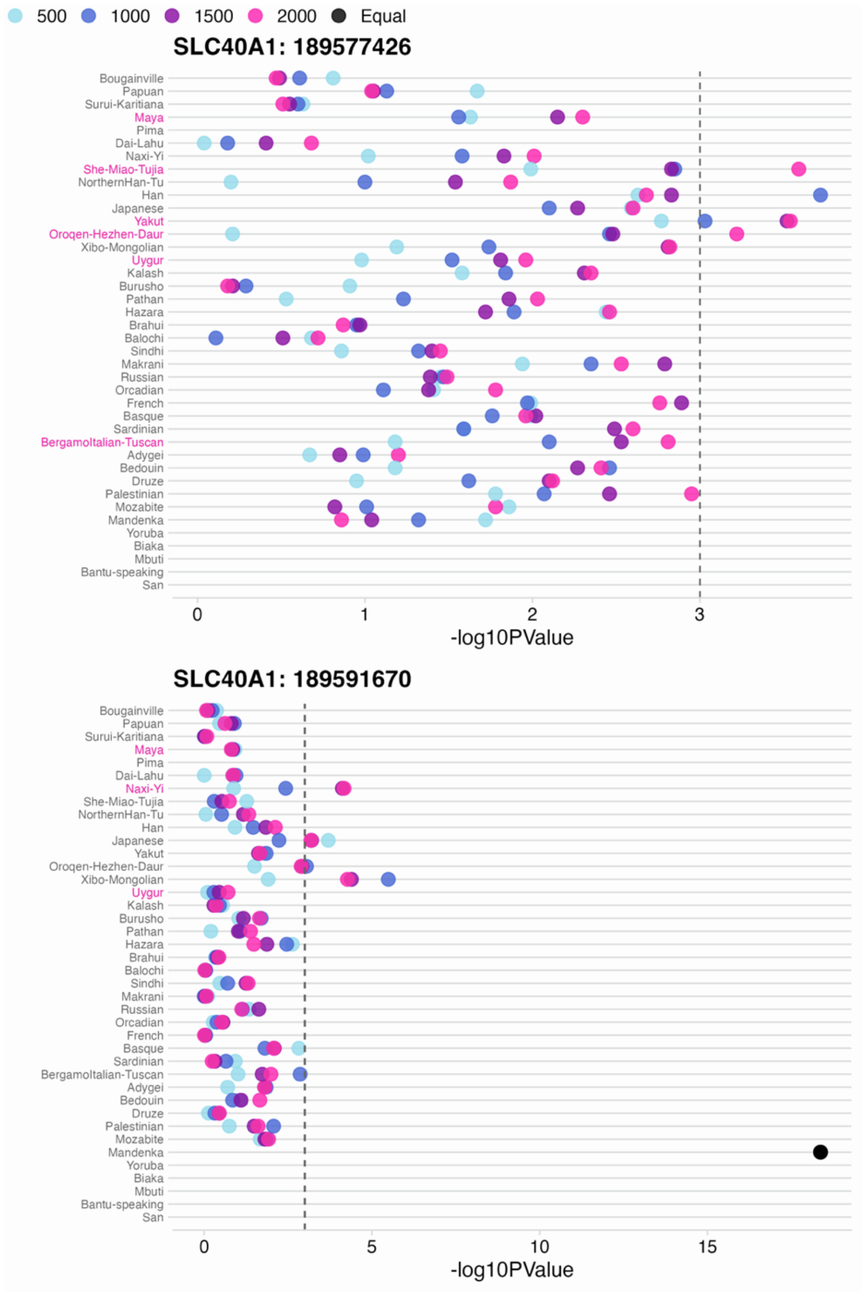

Fig. S29: Evidence for positive selection as inferred by CLUES2 for candidate SNPs in calcium-associated *SLC40A1*. Evidence for positive selection as inferred by CLUES2 for candidate SNPs (positions given) in iron-associated *SLC40A1* (see **Supp. Note 5** and **Table S6**) across four generational timepoints (see top legend). Dashed vertical line indicates $p = 0.001$. Black points indicate that the evidence for selection is equal across all four tested timepoints. Populations in pink are those with signatures of positive selection ($p \leq 0.001$) identified by Relate or F_{ST}.

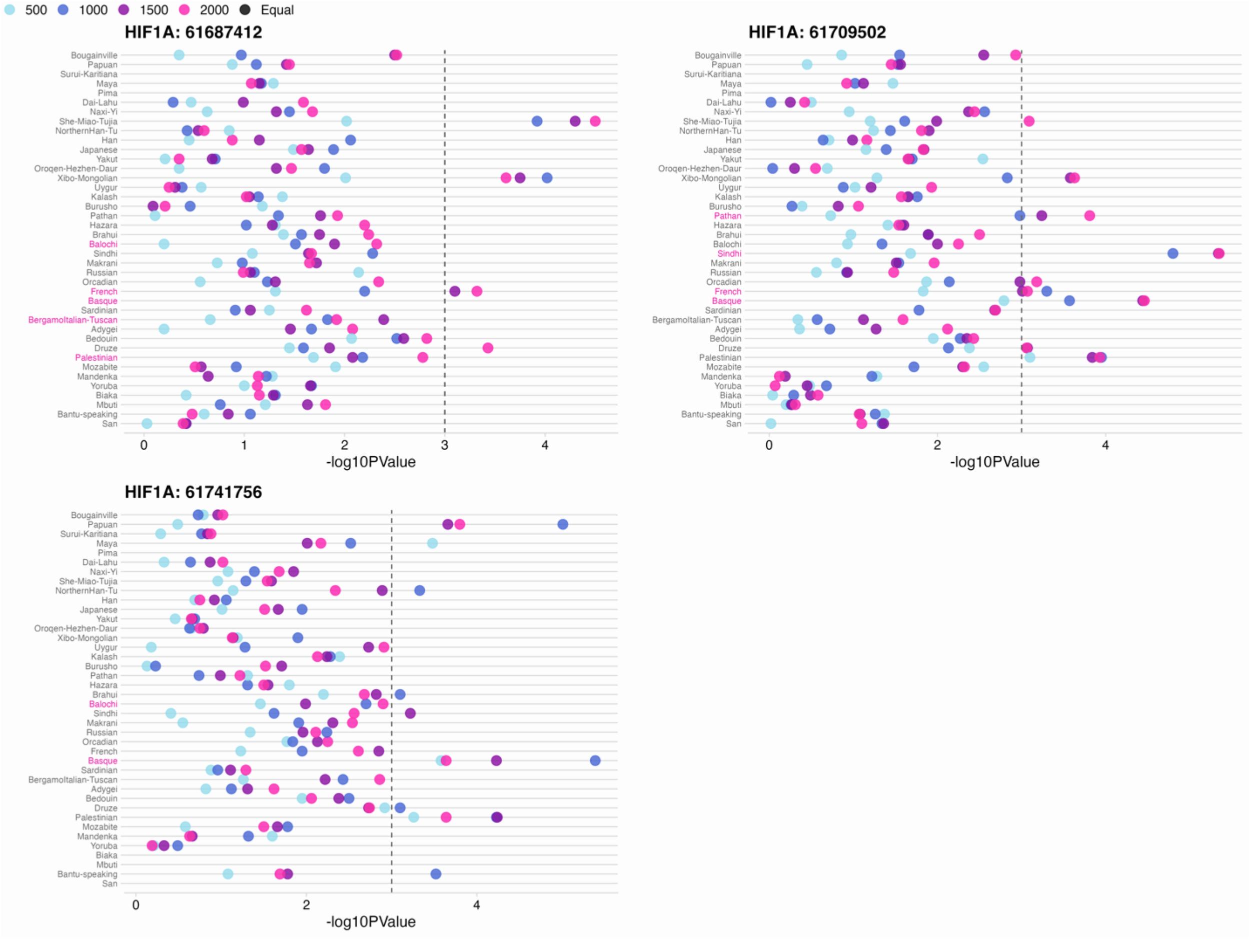

Fig. S30: Evidence for positive selection as inferred by CLUES2 for candidate SNPs in calcium-associated *HIF1A*. Evidence for positive selection as inferred by CLUES2 for candidate SNPs (positions given) in iron-associated *HIF1A* (see **Supp. Note 5** and **Table S6**) across four generational timepoints (see top legend). Dashed vertical line indicates $p = 0.001$. Black points indicate that the evidence for selection is equal across all four tested timepoints. Populations in pink are those with signatures of positive selection ($p \leq 0.001$) identified by Relate or F_{ST}.

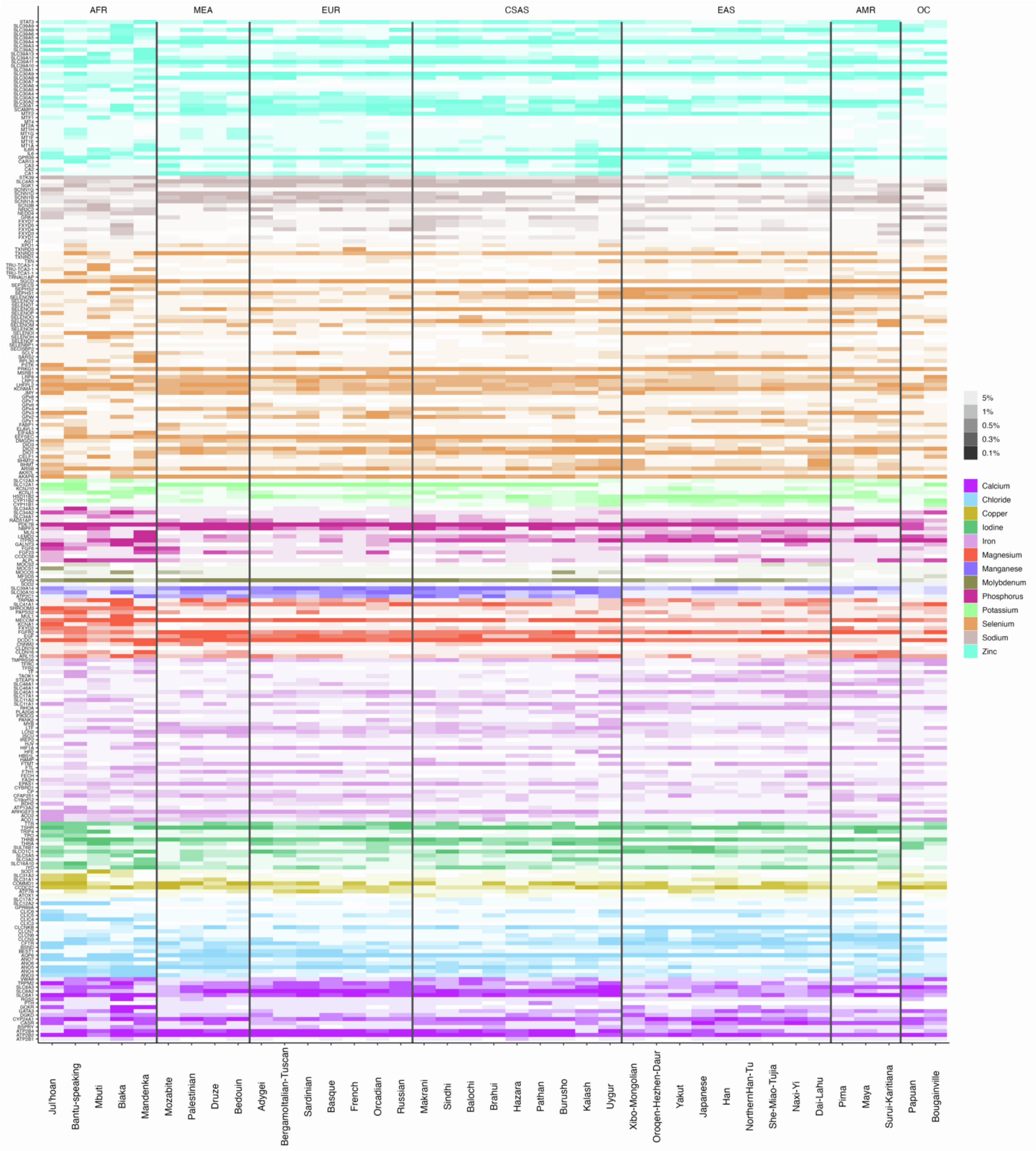

Figure S31: Signatures of positive selection as inferred by F_{ST} across all autosomal MA-genes. Signatures of positive selection as inferred by F_{ST} across all autosomal MA-genes (Y-axis, coloured by micronutrient) and all populations (X-axis, grouped by metapopulation). Darker blocks reflect lower empirical p-values (from lightest to darkest: below 5%, 1%, 0.5%, 0.3%, 0.1%, see left legend).

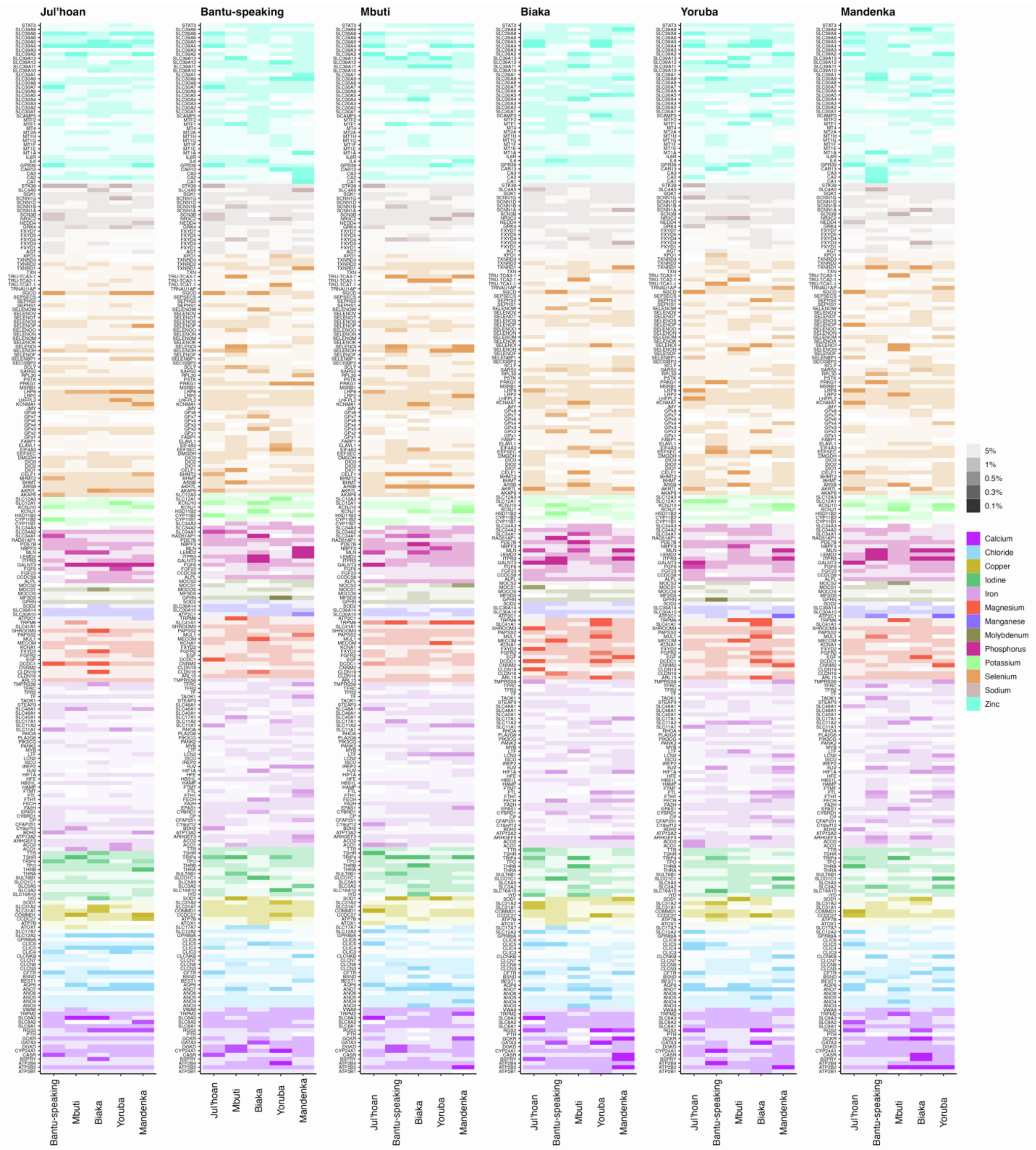

Figure S32: Signatures of positive selection as inferred by F_{ST} across all autosomal MA-genes for all within-Africa pairs. Signatures of positive selection as inferred by F_{ST} across all autosomal MA-genes (Y-axis, coloured by micronutrient) for all within-Africa pairs (each labelled panel represents one population vs. all other African populations on the X-axis). Darker blocks reflect lower empirical p-values (from lightest to darkest: below 5%, 1%, 0.5%, 0.3%, 0.1%, see left legend).

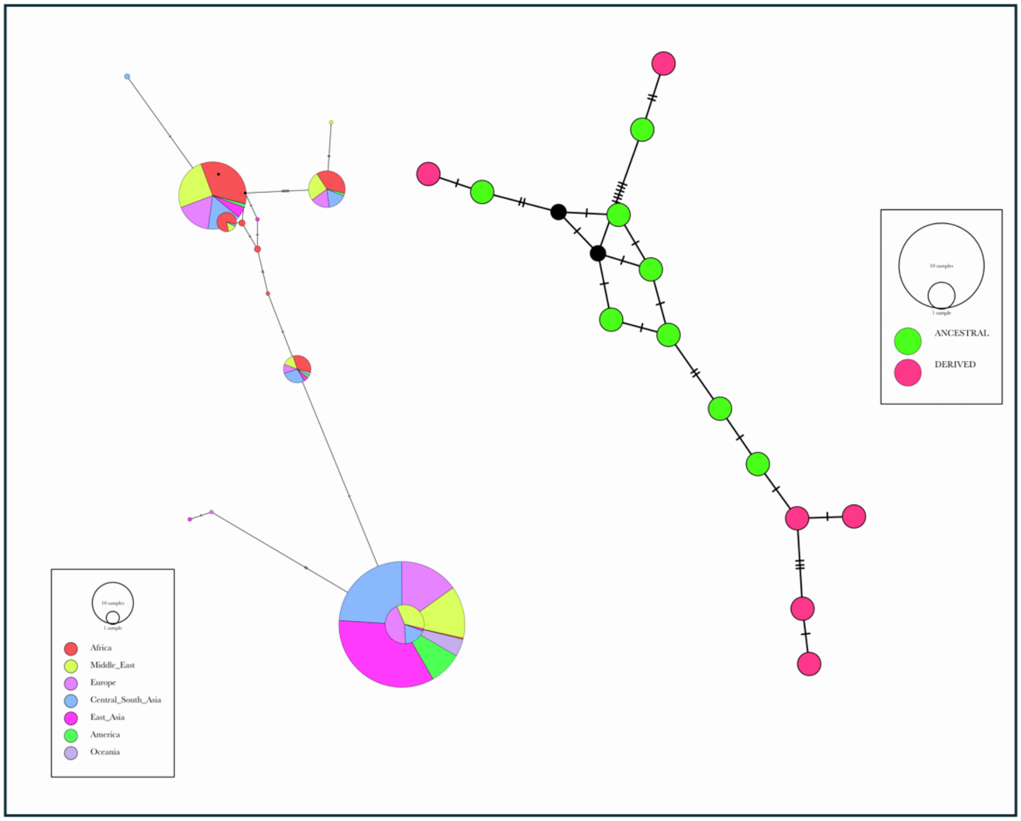

Figure S33: : Haplotype network for region surrounding chr4: 41981527. Haplotype network built from the 10kb region surrounding the chr4: 41981527 SNP (position given) of SLC30A9. Left: Red = Africa; Yellow = Middle-East; Purple = Europe; Blue = Central-South Asia; Pink = East Asia; Green= America; Lilac = Oceania. Right: Green= Ancestral, Pink = Derived.

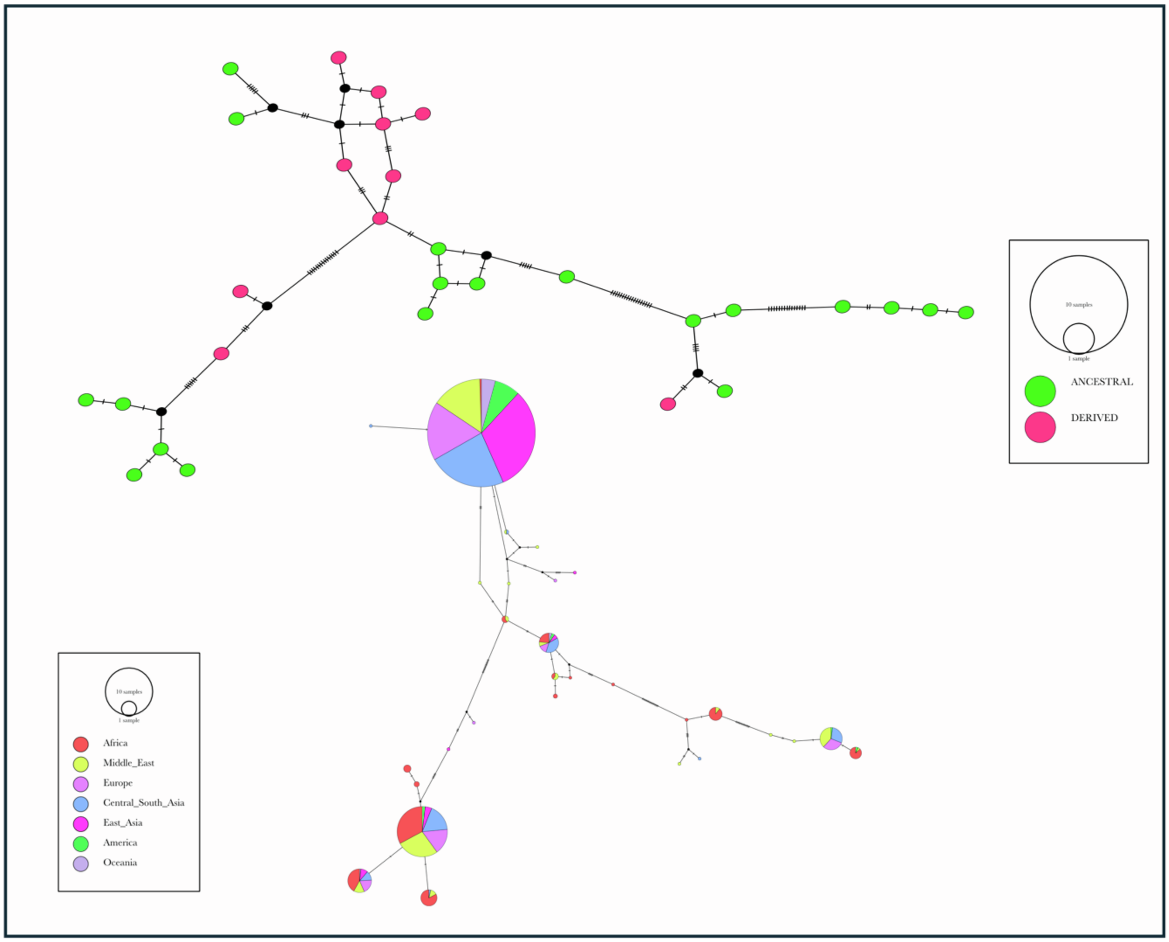

Figure S34: **Haplotype network for region surrounding chr4: 42001116.** Haplotype network built from the 10kb region surrounding the chr4: 42001116 SNP (position given) of SLC30A9. Top: Green= Ancestral, Pink = Derived. Bottom: Red = Africa; Yellow = Middle-East; Purple = Europe; Blue = Central-South Asia; Pink = East Asia; Green= America; Lilac = Oceania.

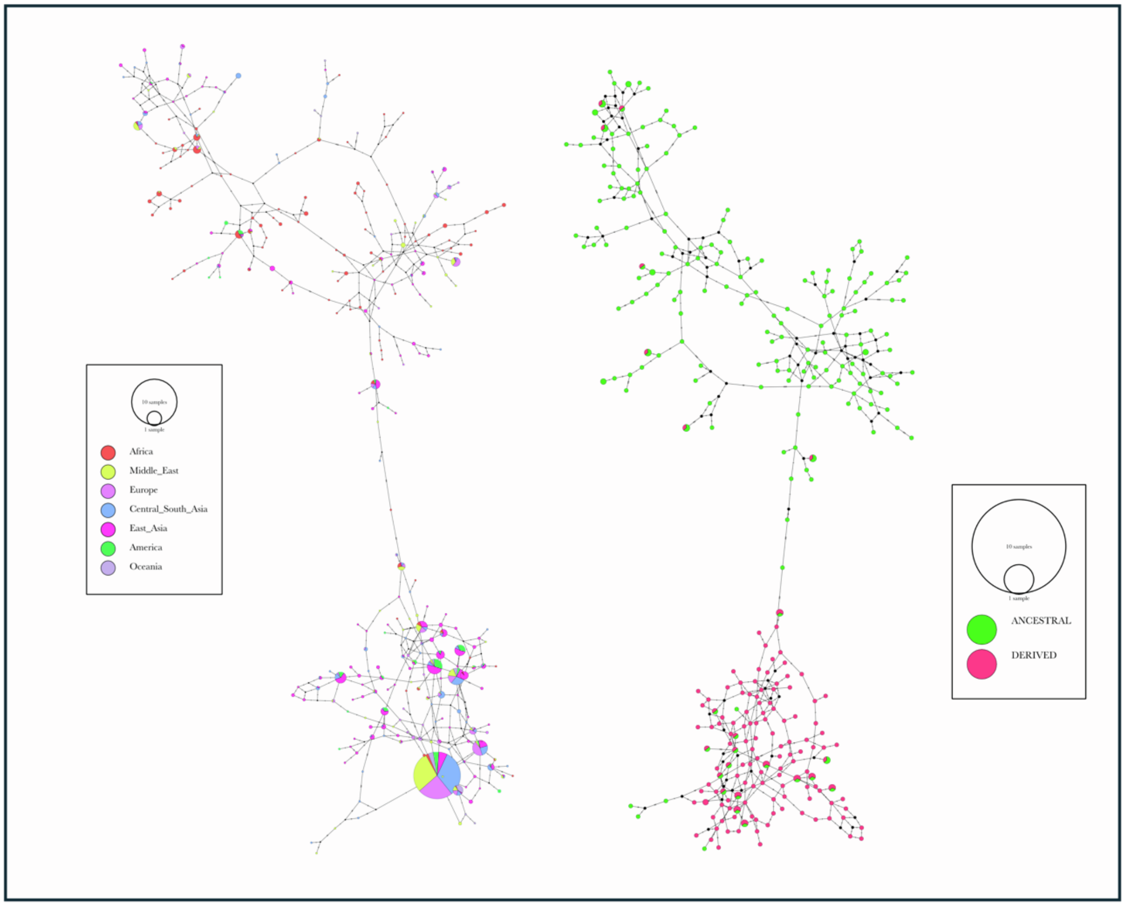

Figure S35: Haplotype network for region surrounding chr17: 73012306.
Haplotype network built from the 10kb region surrounding the chr17: 73012306 (position given) SNP of SLC39A11. Left: Red = Africa; Yellow = Middle-East; Purple = Europe; Blue = Central-South Asia; Pink = East Asia; Green= America; Lilac = Oceania. Right: Green= Ancestral, Pink = Derived.

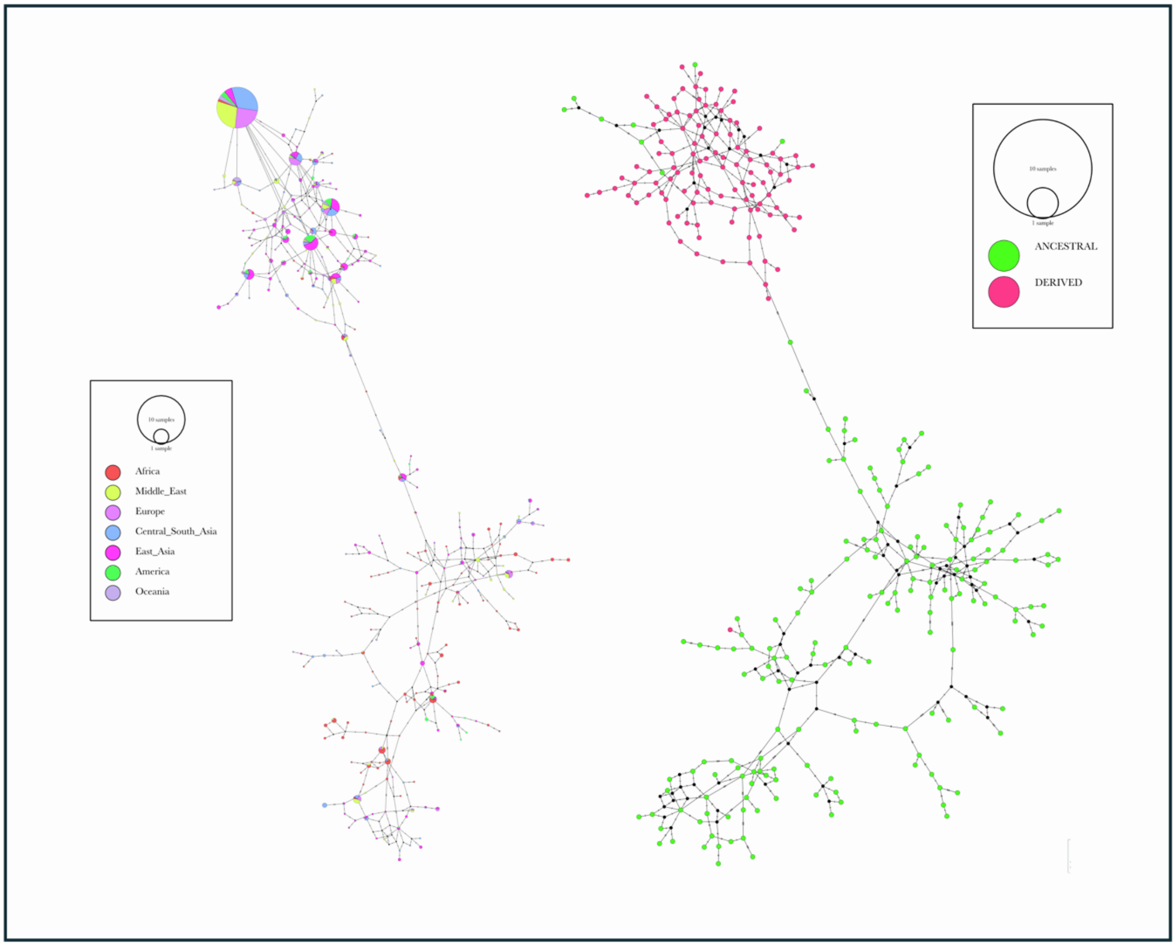

Figure S36: **Haplotype network for region surrounding chr17: 73010373.** Haplotype network built from the 10kb region surrounding the chr17: 73010373 SNP (position given) of SLC39A11. Left: Red = Africa; Yellow = Middle-East; Purple = Europe; Blue = Central-South Asia; Pink = East Asia; Green= America; Lilac = Oceania. Right: Green= Ancestral, Pink = Derived.

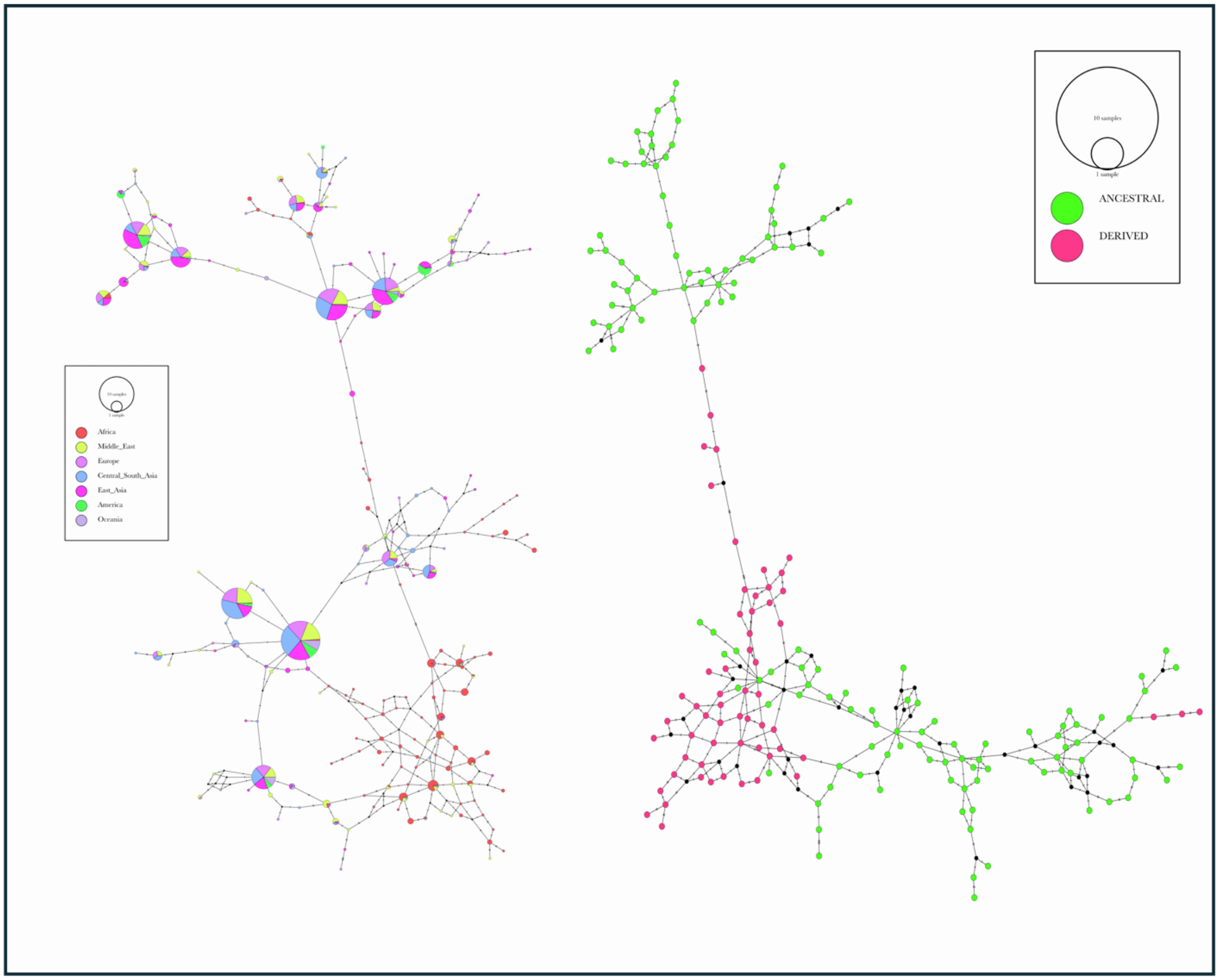

Figure S37: **Haplotype network for region surrounding chr2: 132638916.** Haplotype network built from the 10kb region surrounding the chr2: 132638916 (position given) SNP of GPR39. Left: Red = Africa; Yellow = Middle-East; Purple = Europe; Blue = Central-South Asia; Pink = East Asia; Green= America; Lilac = Oceania. Right: Green= Ancestral, Pink = Derived.

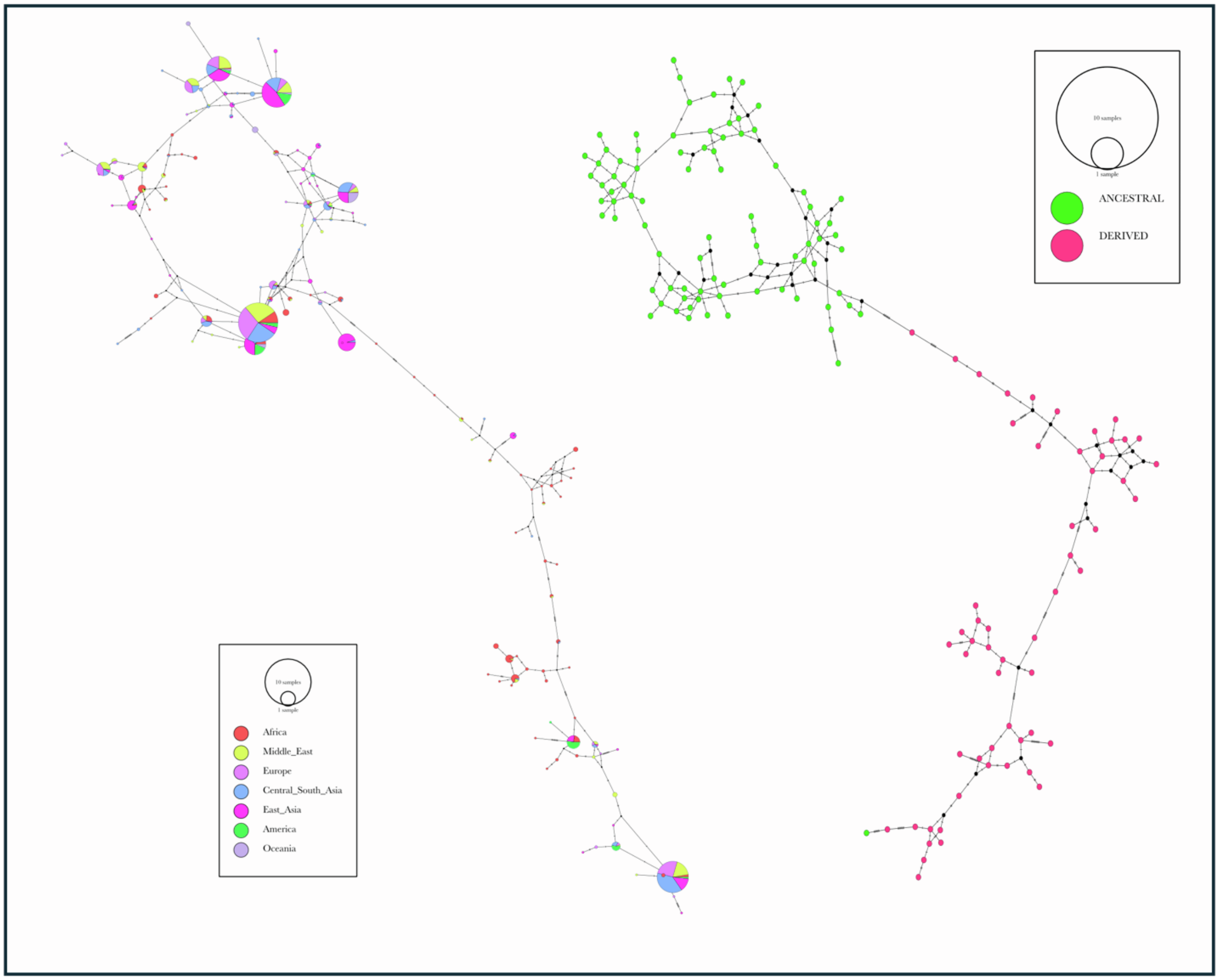

Figure S38: **Haplotype network for region surrounding chr2: 132602934**.Haplotype network built from the 10kb region surrounding the chr2: 132602934 (position given) SNP of GPR39. Left: Red = Africa; Yellow = Middle-East; Purple = Europe; Blue = Central-South Asia; Pink = East Asia; Green= America; Lilac = Oceania. Right: Green= Ancestral, Pink = Derived.

Tables

	Micronutrient	Number of Associated Genes		
		Total Set	Post-mask	Cut-down
**Trace Minerals**	Selenium	61	59	59
	Copper	11	11	9
	Iron	44	44	44
	Zinc	46	45	42
	Iodine	18	18	14
	Manganese	7	7	4
	Molybdenum	5	5	5
**Macrominerals**	Calcium	23	21	17
	Phosphorus	16	16	14
	Magnesium	19	19	15
	Sodium	20	20	17
	Chloride	25	23	22
	Potassium	11	11	7

Table S1: The total number of genes used in this study associated with the uptake, metabolism or regulation of 13 micronutrients. Number of genes for each MA-gene set given as a total (“Total Set”), the number of genes following the removal of any masked gene regions (“Post-mask”) and when cutting down gene sets to remove any overlap, assigning each gene to its most supported associated micronutrient set (“Cut-down”).

	Number of SNPs	Mean	Difference to Background Mean	Median	Difference to Background Median	Standard Deviation	Difference to Background Standard Deviation	Significance
**Selenium**	17614	0.337	-0.008	0.227	0.000	0.282	-0.008	0.057
**Copper**	**1409**	**0.315**	**-0.030**	**0.182**	**-0.045**	**0.279**	**-0.011**	**0.0008543**
**Iron**	6476	0.343	-0.002	0.227	0.000	0.292	0.002	0.6131
**Magnesium**	**7862**	**0.356**	**0.011**	**0.227**	**0.000**	**0.293**	**0.003**	**0.03418**
**Zinc**	7755	0.356	0.011	0.227	0.000	0.297	0.007	0.1861
**Sodium**	**5016**	**0.327**	**-0.018**	**0.205**	**-0.023**	**0.284**	**-0.006**	**0.001019**
**Calcium**	6978	0.344	-0.001	0.205	-0.023	0.293	0.003	0.6302
**Iodine**	4035	0.351	0.006	0.250	0.023	0.290	0.000	0.2436
**Chloride**	8514	0.337	-0.008	0.227	0.000	0.281	-0.009	0.9225
**Potassium**	1682	0.342	-0.003	0.250	0.023	0.276	-0.014	0.1838
**Phosphorus**	2662	0.344	-0.001	0.205	-0.023	0.294	0.004	0.8334
**Manganese**	2152	0.348	0.003	0.205	-0.023	0.289	-0.001	0.08396
**Molybdenum**	**1390**	**0.427**	**0.082**	**0.273**	**0.045**	**0.305**	**0.015**	**2.20E-16**

Table S2: Details on the allele frequency distribution of all micronutrient-associated gene sets. The number of SNPs over all genes in a given gene set (calculated from the Yoruba population), mean, median and standard deviation of the allele frequency distribution of all micronutrient-associated gene sets, the difference to that of the background (allele frequency distribution of chr1 of the Yoruba population; mean= 0.345, median= 0.227, standard deviation= 0.290), and the significance calculated when comparing these distributions (unpaired Wilcoxon test). Excluding the molybdenum gene set, the differences to the background mean are negligible.

Method	Micronutrient	Population	Significance	
Relate	Phosphorus	Pima	0.000013	**< 0.0001**
	**Sodium**	**Adygei**	**0.000029**	
	**Potassium**	**French**	**0.000322**	**< 0.001**
	Iodine	Maya	0.000325	
	**Sodium**	**Brahui**	**0.00115**	**<0.01**
	**Potassium**	**BergamoItalian_Tuscan**	**0.002963**	
	Sodium	Bougainville	0.003455	
	Potassium	Bougainville	0.003722	
	**Sodium**	**Russian**	**0.004935**	
	Sodium	Pathan	0.004951	
	Sodium	Ju|’hoan	0.0057	
	**Sodium**	**Orcadian**	**0.005823**	
	**Sodium**	**French**	**0.006133**	
	Iodine	Mozabite	0.006333	
	Calcium	Mozabite	0.007348	
	Iodine	Russian	0.009037	
	Sodium	NorthernHan_Tu	0.010827	**<0.05**
	**Sodium**	**BergamoItalian_Tuscan**	**0.01141**	
	**Sodium**	**Basque**	**0.011611**	
	Potassium	NorthernHan_Tu	0.014566	
	Sodium	Dai_Lahu	0.01592	
	**Potassium**	**Russian**	**0.017106**	
	Potassium	Druze	0.018052	
	Calcium	Sardinian	0.018387	
	Calcium	Pima	0.018616	
	**Sodium**	**Sindhi**	**0.021039**	
	Potassium	Xibo_Mongolian	0.021087	
	**Selenium**	**Xibo_Mongolian**	**0.02171**	
	Magnesium	Surui_Karitiana	0.023716	
	Potassium	Mandenka	0.02482	
	**Potassium**	**Sindhi**	**0.027062**	
	**Potassium**	**Palestinian**	**0.033025**	
	**Potassium**	**Mozabite**	**0.033461**	
	Manganese	Naxi_Yi	0.034193	
	Zinc	Naxi_Yi	0.03529	
	**Potassium**	**Sardinian**	**0.035508**	
	Phosphorus	Yoruba	0.036495	
	Iodine	Orcadian	0.036859	
	Copper	Sardinian	0.037657	
	Calcium	Japanese	0.038349	
	**Potassium**	**Kalash**	**0.038729**	
	Phosphorus	PapuanHighlands_PapuanSepik	0.04091	
	Calcium	Maya	0.042026	
	**Potassium**	**Pathan**	**0.044689**	
	Calcium	Pathan	0.044822	
	Potassium	Yoruba	0.045052	
	Phosphorus	Pathan	0.046086	
	Calcium	Orcadian	0.047252	
F_{ST}	Potassium	BantuSouthAfrica_BantuKenya	0.000043	**< 0.0001**
	Sodium	Makrani	0.00048	**< 0.001**
	Calcium	Mandenka	0.000912	
	Calcium	Biaka	0.001264	**< 0.01**
	Potassium	Orcadian	0.001556	
	Potassium	Surui_Karitiana	0.001698	
	**Potassium**	**Russian**	**0.002343**	

Zinc	Kalash	0.004891	
**Potassium**	**Palestinian**	**0.005466**	
Phosphorus	Mandenka	0.006715	
Sodium	Surui_Karitiana	0.0068	
**Potassium**	**Mozabite**	**0.008791**	
**Potassium**	**French**	**0.0088**	
**Potassium**	**Kalash**	**0.009572**	
**Selenium**	**Xibo_Mongolian**	**0.00993**	
Potassium	Pima	0.012051	**<0.05**
**Sodium**	**French**	**0.013281**	
Zinc	Uygur	0.016652	
**Sodium**	**Orcadian**	**0.016658**	
**Sodium**	**Russian**	**0.017819**	
Potassium	Basque	0.018501	
Potassium	Bedouin	0.01917	
**Sodium**	**BergamoItalian_Tuscan**	**0.020302**	
Potassium	Adygei	0.021671	
Iron	Mandenka	0.022027	
Potassium	Makrani	0.022717	
Potassium	Brahui	0.022756	
**Sodium**	**Sindhi**	**0.024379**	
**Sodium**	**Basque**	**0.024715**	
Selenium	Japanese	0.025141	
**Potassium**	**Sardinian**	**0.026595**	
**Sodium**	**Brahui**	**0.026743**	
Magnesium	Biaka	0.027088	
**Sodium**	**Adygei**	**0.028808**	
**Potassium**	**BergamoItalian_Tuscan**	**0.029975**	
Selenium	Pima	0.031199	
Selenium	Surui_Karitiana	0.032171	
**Potassium**	**Sindhi**	**0.032643**	
Selenium	Han	0.033762	
Selenium	She_Miao_Tujia	0.037054	
Selenium	Oroqen_Hezhen_Daur	0.038023	
Potassium	Balochi	0.038248	
Potassium	Dai_Lahu	0.042516	
Potassium	Burusho	0.042705	
Sodium	Kalash	0.043516	
**Potassium**	**Pathan**	**0.046447**	
Sodium	Biaka	0.047979	
Potassium	Ju|’hoan	0.048742	
Potassium	Maya	0.048774	

Table S3: Micronutrient-associated gene sets with significantly different summed selection values. According to the gene set method $\mathrm{SUMSTAT}$ integrating Relate or F_{ST} selection values. Bold rows are for MA-gene set and population pairs with significantly different summed selection results according to both integrating Relate and F_{ST} selection values. Partitioned by significance.

Method	Micronutrient	Population	Significance	
Relate	Phosphorus	Pima	0.005012	**< 0.01**
	**Selenium**	**Xibo_Mongolian**	**0.02171**	**< 0.05**
F_{ST}	**Selenium**	**Xibo_Mongolian**	**0.00993**	**< 0.01**
	Iron	Mandenka	0.022027	
	Selenium	Japanese	0.025141	
	Selenium	Pima	0.031199	
	Selenium	Surui_Karitiana	0.032171	
	Selenium	Han	0.033762	
	Selenium	She_Miao_Tujia	0.037054	
	Selenium	Oroqen_Hezhen_Daur	0.038023	**< 0.05**

Table S4: Micronutrient-associated gene sets, as cut down to remove overlap, with significantly different summed selection values. According to the gene set method $\mathrm{SUMSTAT}$ integrating Relate or $\mathrm{F_{ST}}$ selection values. Bold rows are for MA-gene set and population pairs with significantly different summed selection results according to both integrating Relate and $\mathrm{F_{ST}}$ selection values. Partitioned by significance.

Micronutrient	Gene	Candidate SNP Position	rsID	Ancestral/Derived	Consequence	CADD Score	No. Populations		Strongest Evidence	
							Relate	Fst	Relate	Fst
Calcium	*ATP2B2*	chr3:10456514	rs638107	G/C	Intron variant	4.955	3	9	4.97e-5 (Makrani)	0.0003 (Basque)
		chr3:10604833	rs11720808	C/A	Intron variant	0.053	3	1	2.10e-7 (Sardinian)	0.00052 (Mozabite)
	ATP2B4	chr1:203648263	rs7540131	T/A	Intron variant	15.17	4	0	0.00054 (Bedouin)	0.0049 (Biaka)
		chr1:203667951	rs12045866	A/G	Intron variant	5.235	3	14	0.00057 (Bedouin)	0.00019 (Russian)
	SLC8A1	chr2:40394610	rs2192773	A/T	Intron variant	0.567	3	0	0.000452 (Yakut)	0.0014 (Uygur)
		chr2:40584510	rs10172760	T/C	Intron variant	3.341	4	0	2.40e-5 (Makrani)	0.013 (Makrani)
	SLC8A2	chr19:47428756	rs2280692	T/C	3 prime UTR variant	4.661	0	5	0.0087 (French)	0.00013 (Sardinian)
		chr19:47437107	rs4802363	C/T	Intron variant	0.477	0	7	0.0033 (Brahui)	0.00033 (Sardinian)
	SLC8A3	chr14:70182346	rs66672884	T/G	Intron variant	3.026	0	2	0.0052 (NorthernHan-Tu)	0.00072 (Uygur)
		chr14:70175561	rs715305	A/G	Intron variant	0.878	2	0	3.90e-5 (NorthernHan-Tu)	
Iron	*ARHGEF3*	chr3:56761998	rs9825091	C/T	Intron variant	9.002	0	14		2.89e-5 (Balochi)
		chr3:57043874	rs55952750	G/A	Intron variant	3.634	1	0	0.00029 (Maya)	0.0043 (Pima)
	HIF1A	chr14:61687412	rs4899055	G/C	Intron variant	0.192	0	5	0.0033 (Balochi)	0.00024 (Basque)
		chr14:61709502	rs12232182	C/T	Intron variant	13.75	4		2.28e-5 (Sindhi)	0.0018 (Surui-Karitiana)
		chr14:61741756	rs4902081	C/G	Intron variant	6.7	1	3	2.43e-6 (Basque)	0.00031 (Pima)
	FTMT	chr5:121846819	rs6595327	A/C	Intron variant	0.277	2	11	3.37e-6 (Yakut)	4.85e-5 (Dai-Lahu)
		chr5:121853801	rs1560550	A/G	Intron variant	4.003	3	1	0.00022 (She-Miao-Tujia)	0.000203 (Dai-Lahu)
	SLC40A1	chr2:189577426	rs10188785	T/G	Intron variant	0.855	3	4	1.62e-5 (Uygur)	0.00031 (Pima)
		chr2:189591670	rs2352270	G/A	Intergenic variant	0.784	0	1	0.0012 (Naxi-Yi)	0.0001 (Pima)

Table S5: The candidate SNPs in CALCIUM- and IRON-associated genes chosen for analysis in $\mathbf{CLUES2}$. The candidate SNPs (positions given) in CALCIUM- and IRON-associated genes chosen for analysis in $\mathrm{CLUES2}$ (see **Note S1**) alongside their inferred genomic consequences (as inferred from dbSNP[52]) and CADD score[53]. "No. Populations" gives the number of populations where this SNP falls into the 0.1% empirical tail of Relate or $\mathrm{F_{ST}}$. "Strongest Evidence" gives the most extreme pvalue over our dataset and the corresponding population.

Population	Top-ranking MA-Gene	Micronutrient	Significance
Ju|'hoan	*PRKG1*	selenium	0.0011774
	AKAP6	selenium	0.0011774
	SGCD	selenium	0.0011774
	SELENOP	selenium	0.0011774
	TXNRD3	selenium	0.0011774
	PSTK	selenium	0.0011774
Mbuti	*SGK1*	selenium	6.77e-5
	SGCD	selenium	8.72e-5
	EEFSEC	selenium	0.00020261
Biaka	*SKG1*	selenium	4.81e-5
	SGCD	selenium	8.77e-5
Mandenka	*TRPM2*	calcium	2.11e-5
	SLC8A1	calcium	7.52e-5
Druze	*FGFR2*	magnesium	6.67E-05
	SHROOM3	magnesium	0.00021632
Bedouin	*EPAS1*	iron	0.00013935
	HBS1L	iron	0.0002138
Adygei	*C19orf12*	iron	2.01E-05
	CFAP251	iron	9.28E-05
	ARHGEF3	iron	0.00015855
Sardinian	*ATP2B2*	calcium	2.10E-07
	SLC8A1	calcium	0.00012589
	SECISBP2	selenium, iodine	3.27E-05
	VWA8	selenium	0.0002822
Basque	*HIF1A*	iron	2.43E-06
	ARHGEF3	iron	2.47E-05
	TXNRD3	selenium	4.79E-05
	EEFSEC	selenium	5.59E-05
French	*SCNN1D*	sodium, potassium	1.87E-06
	ANO3	chloride	4.56E-05
	SLC12A1	sodium, chloride, potassium	0.00022405
Russian	*SLC4A5*	sodium	3.83E-06
	SCNN1D	sodium, potassium	6.81E-06
	SLC30A1	zinc	1.32E-05
	KCNMA1	calcium, potassium	0.000137
	SLC39A11	zinc	0.00015104
Makrani	*SLC8A1*	calcium	2.40E-05
	ATP2B2	calcium	4.97E-05
	SLC39A11	zinc	1.40E-06
	SLC39A12	zinc	2.40E-05
	ATP2B2	calcium	4.97E-05
Sindhi	*HIF1A*	iron	2.28E-05
	HSD11B2	iron	9.68E-05
	SLC39A11	zinc	0.00015917
	SLC30A9	zinc	0.00031178
Brahui	*GPx2*	selenium	0.00010148
	SGK1	selenium	0.00010148
Hazara	*PRKG1*	selenium	5.56E-05
	ARSB	selenium	0.00017433
	SELENOP	selenium	0.00017433
	TXNRD1	selenium	0.00017433
	LRP2	selenium	0.00034417
Pathan	*ANO3*	chloride	1.76E-05
	SLC12A1	sodium, chloride, potassium	5.81E-05
Kalash	*SLC39A10*	zinc, magnesium, manganese	1.21E-05
	SLC12A3	calcium, magnesium, potassium	2.21E-05
	MECOM	magnesium	0.00014809
Uygur	*ATP2B2*	calcium	5.23E-05
	SLC8A1	calcium	0.0002744
	SLC40A1	iron	1.62E-05
	PLA2G6	iron	7.10E-05
Xibo-Mongolian	*SLC8A3*	calcium	6.74E-05
	KCNMA1	calcium, potassium	0.00016803
	PRKG1	selenium	0.0002743

	SELENOP	selenium	0.0002743
Oroqen-Hezhen-Daur	*SLC40A1*	iron	0.00023563
	ARHGEF3	iron	0.00023563
	PRKG1	selenium	0.00023563
	ARSB	selenium	0.00023563
	AKAP6	selenium	0.00023563
Yakut	*AKAP6*	selenium	7.62E-05
	GPx7	selenium	0.00013104
Japenese	*LHFPL2*	selenium	0.00015814
	ATP2B2	selenium	0.0002387
Han	*TRIP4*	iodine	0.00013754
	SLCO1C1	iodine	0.00013754
NorthernHan-Tu	*SLC8A3*	calcium	3.90E-05
	KCNMA1	calcium, potassium	3.90E-05
She-Miao-Tujia	*MLN*	phosphorus	4.27E-06
	ITPR3	phosphorus	1.93E-05
Naxi-Yi	*PRKG1*	selenium	2.17E-05
	SELENOI	selenium	0.00037186
	SLC39A11	zinc	8.43E-05
	SLC39A8	zinc, magnesium, manganese	0.00016511
	IL6	zinc	0.00037186
	SLC30A10	zinc, magnesium, manganese	0.00037186
Dai-Lahu	*SLC8A3*	calcium	4.43E-05
	SLC8A1	calcium	7.39E-05
Pima	*ITPR3*	phosphorus	8.09E-05
	MLN	phosphorus	0.00053942
	LEMD2	phosphorus	0.00053942
Maya	*SLC8A1*	calcium	4.17E-05
	ATP2B1	calcium	0.00015384
	TRPM6	magnesium	5.25E-05
	ARL15	magnesium	0.00015794
	TSHR	iodine	0.00027492
	THRA	iodine	0.00027492
Surui-Karitiana	*CLDN16*	magnesium	0.00012466
	SLC39A8	zinc, magnesium, manganese	0.00020085
	MECOM	magnesium	0.00041299
	SLC39A10	zinc	0.00014355
	SLC39A11	zinc	0.00041299
Papuan	*SLC8A1*	calcium	1.26E-05
	ATP2B2	calcium	1.97E-05
	HIF1A	iron	0.00027674
	HBS1L	iron	0.00027674
Bougainville	*SLC39A11*	zinc	0.000375
	SLC30A6	zinc	0.000375

Table S6: Populations with top-ranking MA-genes that fall within the same MA-gene set according to Relate. "Population" = populations where two or more of the top-ranking MA-genes ("Top-ranking MA-gene"; those with signatures of positive selection ranking within the top-five strongest signatures of positive selection according to Relate for that population) fall within the same micronutrient gene set ("Micronutrient"). Significance according to Relate given in "Significance" column.

Population	Gene	Micronutrient	Significance
Ju|'hoan	*LRP8*	selenium	2.15E-05
	LHFPL2	selenium	3.08E-05
Bantu-speaking	*SLC12A1*	sodium, chloride, potassium	5.06E-06
	KCNJ10	calcium, potassium	1.38E-05
	LHFPL2	selenium	4.99E-06
	PRKG1	selenium	2.11E-05
	EEFSEC	selenium	2.53E-05
Mbuti	*TRU-TCA2-1*	selenium	6.63E-05
	LHFPL2	selenium	7.44E-05
Biaka	*SLC8A1*	calcium	3.05E-05
	ATP2B4	calcium	8.39E-05
	LHFPL2	selenium	8.33E-05
	EEFSEC	selenium	0.00010422
Mandenka	*ATP2B2*	calcium	7.75E-08
	SLC8A1	calcium	3.00E-05
	FTL	iron	1.99E-05
	HJV	iron	2.91E-05
Mozabite	*EEFSEC*	calcium	1.29E-05
	ATP2B4	calcium	8.32E-05
Palestinian	*SLC39A4*	zinc	2.85E-05
	GPR39	zinc	3.72E-05
Druze	*SLC39A4*	zinc	1.02E-05
	GPR39	zinc	3.23E-05
BergamoItalian-Tuscan	*SLC39A4*	zinc	8.84E-06
	GPR39	zinc	8.94E-05
Sardinian	*SLC39A4*	zinc	1.33E-05
	GPR39	zinc	5.57E-05
Basque	*SLC39A4*	zinc	1.41E-05
	GPR39	zinc	0.00013401
French	*SLC12A1*	sodium, chloride, potassium	7.65E-07
	AQP6	chloride	3.41E-05
	SLC39A4	zinc	5.58E-06
	GPR39	zinc	5.35E-05
Orcadian	*SLC12A1*	sodium, chloride, potassium	4.80E-06
	SCNN1A	sodium, potassium	9.97E-05
	SLC39A4	zinc	3.19E-05
	SLC30A10	zinc, magnesium, manganese	9.97E-05
Russian	*SLC12A1*	sodium, chloride, potassium	1.40E-06
	SLC4A5	sodium	1.89E-05
Makrani	*SGCD*	selenium	1.18E-05
	SGK1	selenium	3.59E-05
Sindhi	*SLC39A4*	zinc	1.03E-05
	SLC39A11	zinc	2.99E-05
	GPR39	zinc	5.83E-05
Brahui	*SLC39A4*	zinc	7.98E-06
	GPR39	zinc	2.63E-05
Hazara	*SLC39A4*	zinc	7.24E-06
	SLC30A9	zinc	2.23E-05
	GPR39	zinc	9.24E-05
	MTF2	zinc	9.77E-05
Pathan	*SLC39A4*	zinc	5.47E-06
	GPR39	zinc	3.47E-05
	SLC30A9	zinc	6.08E-05
Burusho	*SLC39A4*	zinc	8.76E-06
	SLC30A9	zinc	5.38E-05
	GPR39	zinc	5.78E-05
Kalash	*SLC39A4*	zinc	1.72E-05
	SLC39A11	zinc	9.98E-05
	GPR39	zinc	0.00013799
Uygur	*SLC39A4*	zinc	5.66E-05
	CA3	zinc	0.00019682
Xibo-Mongolian	*PRKG1*	selenium	1.00E-05
	SEPHS2	selenium	2.66E-05
	SLC30A9	zinc	2.66E-05
	SLC39A4	zinc	5.49E-05
	HSD11B2	iron	6.97E-05
Oroqen-Hezhen-Daur	*SLC30A9*	zinc	1.51E-05

	SLC39A4	zinc	3.99E-05
Yakut	*SLC30A9*	zinc	1.71E-05
	SLC39A4	zinc	3.57E-05
	PRKG1	selenium	0.00010185
	DIO1	selenium, iodine	0.0002481
Japanese	*SLC39A4*	zinc	6.69E-05
	SLC30A9	zinc	0.00014582
Han	*SLC30A9*	zinc	3.55E-06
	SLC39A4	zinc	4.34E-05
NorthernHan-Tu	*SLC39A4*	zinc	5.98E-05
	SLC30A9	zinc	7.01E-05
	PRKG1	selenium	0.00011442
	SEPHS2	selenium	0.00020983
She-Miao-Tujia	*SLC30A9*	zinc	2.05E-05
	SLC39A4	zinc	4.75E-05
Naxi-Yi	*SLC39A4*	zinc	9.03E-05
	SLC30A9	zinc	0.00010709
	SEPHS2	selenium	0.00010969
	PRKG1	selenium	0.0001864
Dai-Lahu	*SLC30A9*	zinc	2.12E-05
	SLC39A4	zinc	7.82E-05
Pima	*GPx3*	selenium	0.00010471
	SELENON	selenium	0.00010471
	RHOA	iron	0.00010471
	SLC40A1	iron	0.00010471
	STAT3	zinc	0.00010471
	SLC39A11	zinc	0.00010471
	SLC30A2	zinc	0.00010471
Surui-Karitiana	*GPx3*	selenium	0.00012002
	SGCD	selenium	0.00012002
Papuan	*ACO1*	chloride	5.36E-05
	CLCN3	chloride	0.00025195
	SLC39A11	zinc	9.64E-05
	SLC30A9	zinc	0.00025195
Bougainville	*TFRC*	iron	3.80E-05
	TMPRSS6	iron	8.23E-05
	DCDC1	magnesium	3.80E-05
	SLC41A1	magnesium	8.23E-05

Table S7: Populations with top-ranking MA-genes that fall within the same MA-gene set according to $\mathbf{F_{ST}}$. "Population" = populations where two or more of the top-ranking MA-genes ("Top-ranking MA-gene"; those with signatures of positive selection ranking within the top-five strongest signatures of positive selection according to F_{ST} for that population) fall within the same micronutrient gene set ("Micronutrient"). Significance according to F_{ST} given in "Significance" column.

Gene	Focal SNP	No. Populations $Relate$	F_{ST}
SLC30A9	chr4:41981527	3	25*
	chr4:42001116	8*	21
SLC39A11	chr17:73012306	11	20*
	chr17:73010373	15*	6
GPR39	chr2:132638916	1	30*
	chr2:132598713	3*	2

Table S8: **Candidate SNPs of ZINC-associated genes with geographically widespread signatures of positive selection**. Candidate SNPs of ZINC-associated genes with geographically widespread signatures of positive selection (those identified as candidate SNPs in ten or more populations) that are identified as candidate SNPs in the highest number of populations according to either Relate and $\mathrm{F_{ST}}$ ["No. Populations" gives the number of populations where this SNP is identified as a candidate SNP for either Relate and $\mathrm{F_{ST}}$]. Asteriks indicate that this is the most commonly identified candidate SNP in this gene according to the column's respective method.

Gene	0.1% tail	
	No.	Populations
PRKG1	24	Bantu-speaking, Biaka, Yoruba, Mandenka, Palestinian, Bedouin, Adygei, Sardinian, Basque, Sindhi, Balochi, Hazara, Pathan, Burusho, Kalash, Xibo-Mongolian, Oroqen-Hezhen-Daur, Yakut, Japanese, Han, NorthernHan-Tu, She-Miao-Tujia, Naxi-Yi, Dai-Lahu
SGCD	21	Bantu-speaking, Mbuti, Biaka, Mozabite, Palestinian, Druze, Bedouin, BergamoItalian-Tuscan, Sardinian, Basque, Sindhi, Hazara, Burusho, Oroqen-Hezhen-Daur, Yakut, Han, NorthernHan-Tu, She-Miao-Tujia, Naxi-Yi, Dai-Lahu, Bougainville
AKAP6	20	Bantu-speaking, Biaka, Yoruba, Mandenka, Mozabite, Palestinian, Druze, Bedouin, Adygei, BergamoItalian-Tuscan, Sardinian, Basque, Burusho, Xibo-Mongolian, Oroqen-Hezhen-Daur, Yakut, NorthernHan-Tu, Naxi-Yi, Surui-Karitiana, Papuan
KCNMA1	16	Mbuti, Biaka, Yoruba, Mandenka, Adygei, BergamoItalian-Tuscan, Russian, Sindhi, Xibo-Mongolian, Yakut, Japanese, Han, NorthernHan-Tu, She-Miao-Tujia, Naxi-Yi, Dai-Lahu
EEFSEC	5	Bantu-speaking, Mbuti, Adygei, Basque, Balochi
ARSB	5	Hazara, Burusho, Xibo-Mongolian, Oroqen-Hezhen-Daur, Japanese
SELENOP	5	Bantu-speaking, Mandenka, Hazara, Pathan, Xibo-Mongolian
LRP8	5	Bantu-speaking, Yoruba, Mandenka, Balochi, Xibo-Mongolian
SELENOS	5	Biaka, Yoruba, Palestinian, Balochi, NorthernHan-Tu
LHFPL2	4	Biaka, Mandenka, Japanese, Maya
SCLY	4	Bantu-speaking, Mozabite, Russian
GPx2	4	BergamoItalian-Tuscan, Makrani, Balochi, Brahui
TXNRD3	3	Mozabite, Adygei, Basque
SECISBP2	3	Mandenka, Sardinian, Russian
AKR7L	1	Burusho

TXNRD2	1	Dai-Lahu
SELENOM	1	Yoruba

Table S9: **The populations with signatures of positive selection for all selenium-associated genes.** The number ("No.") and name of the populations ("Populations") with signatures of positive selection, as identified by the 0.1% tail of the empirical background distribution of Relate, for all selenium-associated genes with p-values $< 10^{-5}$ in at least one population.

Gene	Population	$Relate$ Significance	F_{ST} Significance
PRKG1	Bantu-speaking		2.11e-5
	Palestinian	6.83e-5	
	Hazara	5.56e-5	
	Han	6.54e-5	
	Naxi-Yi	2.17e-5	
	She-Miao-Tujia		2.05e-5
	Xibo-Mongolian		1e-5
SGCD	Biaka	8.77e-5	
	Mbuti	8.72e-5	
	Palestinian	9.50e-5	
	BergamoItalian-Tuscan		1.07e-5
	Makrani		1.18e-5
	Papuan		5.36e-5
AKAP6	Mozabite	4.16e-5	
	Adygei	4.62e-5	
	Yakut	7.62e-5	
	Surui-Karitiana	3.96e-5	
EEFSEC	Bantu-speaking		2.53e-5
	Bedouin		8.33e-5
	Mozabite		1.29e-5
	Basque	5.59e-5	
LHFPL2	Bantu-speaking		4.99e-6
	Biaka		8.33e-5
	Mbuti		7.44e-5
	Ju|’hoan		3.08e-5
LRP8	Bantu-speaking	8.8e-5	
	Mandenka	1.04e-5	
	Ju|’hoan	2.15e-5	
SELENOS	Russian		4.01e-5
	Brahui		8.04e-5
	Hazara		1.86e-5
KCNMA1	NorthernHan-Tu	3.9e-5	
	Yakut	1.46e-5	
SLCY	Mozabite	9.09e-5	
TXNDR3	Basque	4.79e-5	
SECISBP2	Sardinian	3.27e-5	
AKR7L	Burusho	9.83e-6	
GPx2	Makrani	9.61e-6	
TXNRD2	Mandenka		9.72e-5
TRU-TCA2-1	Mbuti		6.63e-5
ARSB	Orcadian		7.92e-5
SELENOM	Yoruba	5.87e-6	
SELENOP	Pathan	4.09e-5	
SELENOW	Japanese		6.91e-5
SEPHS2	Xibo-Mongolian		2.66e-5

Table S10: **Selenium-associated genes with p-values $< 10^{-5}$.** Selenium-associated genes with p-values $< 10^{-5}$, as calculated from the empirical distribution of either Relate or F_{ST}. P-values less than 4.65×10^{-6} (see **Section 3.4.5**) highlighted in bold.

Overlapping Genes				Overlap (bp)	Nature of Overlap
GPx1	(selenium)	RHOA	(iron)	787	$\pm 10kbp$
LHFPL2	(selenium)	ARSB	(selenium)	7188	$\pm 10kbp$
LEMD2	(phosphorus)	MLN	(phosphorus)	5543	$\pm 10kbp$
MT1F	(zinc)	MT1G	(zinc)	6032	$\pm 10kbp$
MT1G	(zinc)	MT1H	(zinc)	1749	$\pm 10kbp$
FXYD1	(sodium)	FXYD7	(sodium)	141	$\pm 10kbp$
FXYD7	(sodium)	FXYD5	(sodium)	428	$\pm 10kbp$
DMGDH	(selenium)	BHMT2	(selenium)	166271	$ensemble$
GPx5	(selenium)	GPx6	(selenium)	2334	$ensemble$
CA1	(zinc)	CA3	(zinc)	5578	$ensemble$
BEST1	(chloride)	FT1H	(zinc)	5797	$ensemble$

Table S11: **All overlapping micronutrient-associated genes**. All micronutrient-associated genes which are less than 10kbp (“Nature of Overlap” = “$\pm 10\mathrm{kbp}$”) or have overlapping gene regions as given by $\mathrm{ensemble}$ (“Nature of Overlap” = “$\mathrm{ensemble}$”)

Micronutrient	Gene	SNPs	CDF
Selenium	*SELENOO*	1083	0.99776
Iron	*EPAS1*	2829	0.97643
Zinc	*MT1A*	584	0.97112
Zinc	*MT1F*	631	0.98755
Sodium, Potassium	*SCNN1D*	807	0.95443
Calcium	*SLC8A1*	13155	0.98523
Chloride	*CLCN7*	1388	0.99038

Table S12: **Micronutrient-associated genes enriched for SNP-density** . Micronutrient genes enriched for SNP-density (over 95% quantile of the cumulative density function drawn from the distribution formed from generated neutral gene regions).

Metapopulation	Group name	Populations	Sample Size
**Africa**	Mbuti	Mbuti	13
	Biaka	Biaka	22
	Ju|'hoan	Ju|'hoan	6
	Bantu-speaking	Bantu(Kenya), Bantu(SouthAfrica)	19
	Yoruba	Yoruba	22
	Mandenka	Mandenka	22
**Middle-East**	Mozabite	Mozabite	27
	Palestinian	Palestinian	46
	Druze	Druze	42
	Bedouin	Bedouin	46
**Europe**	BergamoItalian-Tuscan	Bergamo_Italian, Tuscan	21
	Russian	Russian	25
	Adygei	Adygei	16
	Orcadian	Orcadian	15
	French	French	28
	Basque	Basque	23
	Sardinian	Sardinian	28
	Russian	Russian	25
**East-Asia**	Xibo-Mongolian	Mongolian, Xibo	18
	NorthernHan-Tu	NorthernHan, Tu	20
	Naxi-Yi	Naxi, Yi	18
	She-Miao-Tujia	She, Miao, Tujia	29
	Oroqen-Hezhen-Daur	Oroqen, Hezhen, Daur	27
	Dai-Lahu	Dai, Lahu	17
	Han	Han	33
	Japanese	Japanese	27
	Yakut	Yakut	25
**Central-South Asia**	Hazara	Hazara	19
	Uygur	Uygur	10
	Makrani	Makrani	25
	Sindhi	Sindhi	24
	Balochi	Balochi	24
	Brahui	Brahui	25
	Burusho	Barusho	25
	Kalash	Kalash	22
	Pathan	Pathan	24

**Oceania**	Papuan	Papuan (Sepik), Papuan(Highlands)	17
	Bougainville	Bougainville	11
**Americas**	Pima	Pima	13
	Maya	Maya	21
	Surui-Karitiana	Surui, Karitiana	20

Table S13: **Populations used in this study.** Populations used in this study, as defined by[2] .Note, Ju|'hoan refers to the population previously labelled San by the Human Diversity Genome Panel (HGDP).

Population	Correlation	Rsquared	Pvalue
**Ju|'hoan**	0.07659437	0.0058667	2.55E-40
**BantuSouthAfrica_BantuKenya**	0.03474197	0.001207	1.79E-16
**Mbuti**	0.14264497	0.02034759	1.30E-213
**Biaka**	0.07205673	0.00519217	6.80E-75
**Mandenka**	0.01919085	0.00036829	3.08E-06
**Mozabite**	0.16813781	0.02827032	0
**Palestinian**	0.21530503	0.04635626	0
**Druze**	0.21907057	0.04799192	0
**Bedouin**	0.1928931	0.03720775	0
**Adygei**	0.1649932	0.02722276	9.53E-243
**BergamoItalian_Tuscan**	0.20588759	0.0423897	0
**Sardinian**	0.21234659	0.04509107	0
**Basque**	0.21153356	0.04474645	0
**French**	0.22424484	0.05028575	0
**Orcadian**	0.1769971	0.03132797	8.95E-276
**Russian**	0.17270263	0.0298262	1.18E-302
**Makrani**	0.1804534	0.03256343	0
**Sindhi**	0.18332147	0.03360676	0
**Balochi**	0.18597233	0.03458571	0
**Brahui**	0.19563697	0.03827382	0
**Hazara**	0.17675899	0.03124374	7.84E-301
**Pathan**	0.18677213	0.03488383	0
**Burusho**	0.17608256	0.03100507	1.3723108091009e-317
**Kalash**	0.2503178	0.062659	0
**Uygur**	0.17682151	0.03126585	2.48E-239
**Xibo_Mongolian**	0.23409674	0.05480128	0
**Oroqen_Hezhen_Daur**	0.23733334	0.05632712	0
**Yakut**	0.26052163	0.06787152	0
**Japanese**	0.22845015	0.05218947	0
**Han**	0.25153117	0.06326793	0

**NorthernHan_Tu**	0.21461105	0.0460579	0
**She_Miao_Tujia**	0.24431667	0.05969064	0
**Naxi_Yi**	0.21039097	0.04426436	0
**Dai_Lahu**	0.23418859	0.0548443	0
**Pima**	0.28077905	0.07883687	0
**Maya**	0.32331967	0.10453561	0
**Surui_Karitiana**	0.28452489	0.08095441	0
**PapuanHighlands_PapuanSepik**	0.24935343	0.06217713	0
**Bougainville**	0.19203861	0.03687883	7.31E-265

Table S14: Correlation between empirical p-values of MA-genes calculated by F_{ST} and $Relate$. Correlation between empirical p-values of MA-genes calculated by F_{ST} (as calculated between Yoruba and each test population) and $Relate$ for all populations. Populations with positive correlations with p-value < 0.05 are given in italics. Pvalue=0 indicates pvalue below 1e-318.

Population	Correlation	Rsquared	Pvalue
**Ju|'hoan**	*0.07840859*	*0.00614791*	*0.0513838*
**BantuSouthAfrica_BantuKenya**	0.1399856	0.01959597	3.54E-08
**Mbuti**	0.11269175	0.01269943	2.51E-05
**Biaka**	0.19952424	0.03980992	6.53E-14
**Mandenka**	*0.00263365*	*6.94E-06*	*0.91958327*
**Mozabite**	0.22675485	0.05141776	2.23E-33
**Palestinian**	0.13053672	0.01703984	5.81E-12
**Druze**	0.30606577	0.09367626	4.21E-60
**Bedouin**	0.14237412	0.02027039	3.89E-15
**Adygei**	0.1376565	0.01894931	5.63E-12
**BergamoItalian_Tuscan**	0.26151534	0.06839027	6.39E-46
**Sardinian**	0.14279613	0.02039073	2.62E-14
**Basque**	0.23054803	0.0531524	1.19E-32
**French**	0.24662259	0.0608227	2.51E-37
**Orcadian**	0.20221591	0.04089127	3.75E-23
**Russian**	0.24381588	0.05944618	6.08E-38
**Makrani**	0.21591664	0.04662	2.19E-32
**Sindhi**	0.1176469	0.01384079	6.78E-10
**Balochi**	0.16826001	0.02831143	3.86E-19
**Brahui**	0.10323381	0.01065722	4.19E-08
**Hazara**	0.19161027	0.0367145	4.35E-20
**Pathan**	0.14826994	0.02198397	1.40E-14
**Burusho**	0.17353564	0.03011462	1.05E-19
**Kalash**	0.19137435	0.03662414	6.93E-21
**Uygur**	0.15219298	0.0231627	2.90E-12
**Xibo_Mongolian**	0.19156455	0.03669697	2.81E-21
**Oroqen_Hezhen_Daur**	0.25448203	0.0647611	4.29E-34
**Yakut**	0.24049998	0.05784024	3.47E-34
**Japanese**	0.20340443	0.04137336	2.21E-21
**Han**	0.25333765	0.06417997	3.36E-34
**NorthernHan_Tu**	0.16345559	0.02671773	8.62E-15
**She_Miao_Tujia**	0.27300488	0.07453166	4.03E-40
**Naxi_Yi**	0.27932745	0.07802383	3.93E-40
**Dai_Lahu**	0.25160567	0.06330541	3.83E-35
**Pima**	0.18042089	0.0325517	1.91E-13
**Maya**	0.20890365	0.04364074	3.93E-25
**Surui_Karitiana**	0.29465741	0.08682299	2.79E-35
**PapuanHighlands_PapuanSepik**	0.24589531	0.0604645	1.01E-32
**Bougainville**	0.25884529	0.06700088	2.14E-63

Table S15: Correlation between empirical p-values of MA-genes calculated by F_{ST} and $Relate$, pruned by F_{ST} p-value. Correlation between empirical p-values of MA-

genes calculated by $\mathrm{F_{ST}}$ (as calculated between Yoruba and each test population) and $Relate$ for all populations. To allow a better comparison between methods, we prune SNPs to only include those in the 5% tail of the empirical $\mathrm{F_{ST}}$ distribution (selection over the lifetime of a mutation - as captured by $Relate$ – does not necessitate the signatures of differential selection that are identified by $\mathrm{F_{ST}}$) with lower frequency in Yoruba (indicating selection in the test population). Populations with positive correlations with p-value < 0.05 are given in italics.

Supplemental References

1. Haller, B.C., and Messer, P.W. (2019). SLiM 3: Forward Genetic Simulations Beyond the Wright-Fisher Model. Mol Biol Evol. https://doi.org/10.1093/molbev/msy228.
2. Bergström, A., McCarthy, S.A., Hui, R., Almarri, M.A., Ayub, Q., Danecek, P., Chen, Y., Felkel, S., Hallast, P., Kamm, J., et al. (2020). Insights into human genetic variation and population history from 929 diverse genomes. Science (1979) *367*. https://doi.org/10.1126/SCIENCE.AAY5012/SUPPL_FILE/AAY5012-BERGSTROM-SM.PDF.
3. Gravel, S., Henn, B.M., Gutenkunst, R.N., Indap, A.R., Marth, G.T., Clark, A.G., Yu, F., Gibbs, R.A., and Bustamante, C.D. (2011). Demographic history and rare allele sharing among human populations. Proc Natl Acad Sci U S A. https://doi.org/10.1073/pnas.1019276108.

4. Gravel, S., Zakharia, F., Moreno-Estrada, A., Byrnes, J.K., Muzzio, M., Rodriguez-Flores, J.L., Kenny, E.E., Gignoux, C.R., Maples, B.K., Guiblet, W., et al. (2013). Reconstructing Native American Migrations from Whole-Genome and Whole-Exome Data. PLoS Genet. https://doi.org/10.1371/journal.pgen.1004023.
5. Schlebusch, C.M., Sjödin, P., Breton, G., Günther, T., Naidoo, T., Hollfelder, N., Sjöstrand, A.E., Xu, J., Gattepaille, L.M., Vicente, M., et al. (2020). Khoe-San Genomes Reveal Unique Variation and Confirm the Deepest Population Divergence in Homo sapiens. Mol Biol Evol *37*, 2944–2954. https://doi.org/10.1093/MOLBEV/MSAA140.
6. Boyko, A.R., Williamson, S.H., Indap, A.R., Degenhardt, J.D., Hernandez, R.D., Lohmueller, K.E., Adams, M.D., Schmidt, S., Sninsky, J.J., Sunyaev, S.R., et al. (2008). Assessing the evolutionary impact of amino acid mutations in the human genome. PLoS Genet. https://doi.org/10.1371/journal.pgen.1000083.
7. Ragsdale, A.P., Weaver, T.D., Atkinson, E.G., Hoal, E.G., Möller, M., Henn, B.M., and Gravel, S. (2023). A weakly structured stem for human origins in Africa. Nature 2023 617:7962 *617*, 755–763. https://doi.org/10.1038/s41586-023-06055-y.
8. Kim, B.Y., Huber, C.D., and Lohmueller, K.E. (2017). Inference of the distribution of selection coefficients for new nonsynonymous mutations using large samples. Genetics. https://doi.org/10.1534/genetics.116.197145.
9. Lipson, M., Sawchuk, E.A., Thompson, J.C., Oppenheimer, J., Tryon, C.A., Ranhorn, K.L., de Luna, K.M., Sirak, K.A., Olalde, I., Ambrose, S.H., et al. (2022). Ancient DNA and deep population structure in sub-Saharan African foragers. Nature 2022 603:7900 *603*, 290–296. https://doi.org/10.1038/s41586-022-04430-9.
10. Voight, B.F., Kudaravalli, S., Wen, X., and Pritchard, J.K. (2006). A Map of Recent Positive Selection in the Human Genome. https://doi.org/10.1371/journal.pbio.0040072.
11. Schlebusch, C.M., Malmström, H., Günther, T., Sjödin, P., Coutinho, A., Edlund, H., Munters, A.R., Vicente, M., Steyn, M., Soodyall, H., et al. (2017). Southern African ancient genomes estimate modern human divergence to 350,000 to 260,000 years ago. Science (1979) *358*, 652–655. https://doi.org/10.1126/SCIENCE.AAO6266/SUPPL_FILE/AAO6266_SCHLEBUSCH_SM.PDF.
12. Ferrer-Admetlla, A., Liang, M., Korneliussen, T., and Nielsen, R. (2014). On Detecting Incomplete Soft or Hard Selective Sweeps Using Haplotype Structure. Mol Biol Evol. https://doi.org/10.1093/molbev/msu077.
13. Sabeti, P.C., Varilly, P., Fry, B., Lohmueller, J., Hostetter, E., Cotsapas, C., Xie, X., Byrne, E.H., Mccarroll, S.A., Gaudet, R., et al. (2007). Genome-wide detection and characterization of positive selection in human populations. *449*. https://doi.org/10.1038/nature06250.
14. Szpiech, Z.A., Novak, T.E., Bailey, N.P., and Stevison, L.S. (2020). High-altitude adaptation in rhesus macaques. Preprint at bioRxiv, https://doi.org/10.1101/2020.05.19.104380 https://doi.org/10.1101/2020.05.19.104380.

15. Weir, B.S., and Cockerham, C.C. (1984). Estimating F-Statistics for the Analysis of Population Structure. Evolution (N Y) *38*, 1358. https://doi.org/10.2307/2408641.
16. Speidel, L., Forest, M., Shi, S., and Myers, S.R. (2019). A method for genome-wide genealogy estimation for thousands of samples. Nat Genet *51*, 1321–1329. https://doi.org/10.1038/s41588-019-0484-x.
17. Field, Y., Boyle, E.A., Telis, N., Gao, Z., Gaulton, K.J., Golan, D., Yengo, L., Rocheleau, G., Froguel, P., McCarthy, M.I., et al. (2016). Detection of human adaptation during the past 2000 years. Science (1979) *354*, 760–764. https://doi.org/10.1126/science.aag0776.
18. Szpiech, Z.A., and Hernandez, R.D. (2014). Selscan: An efficient multithreaded program to perform EHH-based scans for positive selection. Mol Biol Evol. https://doi.org/10.1093/molbev/msu211.
19. Danecek, P., Auton, A., Abecasis, G., Albers, C.A., Banks, E., DePristo, M.A., Handsaker, R.E., Lunter, G., Marth, G.T., Sherry, S.T., et al. (2011). The variant call format and VCFtools. Bioinformatics. https://doi.org/10.1093/bioinformatics/btr330.
20. Daub, J.T., Hofer, T., Cutivet, E., Dupanloup, I., Quintana-Murci, L., Robinson-Rechavi, M., and Excoffier, L. (2013). Evidence for Polygenic Adaptation to Pathogens in the Human Genome. Mol Biol Evol *30*, 1544–1558. https://doi.org/10.1093/molbev/mst080.
21. Tintle, N.L., Borchers, B., Brown, M., and Bekmetjev, A. (2009). Comparing gene set analysis methods on single-nucleotide polymorphism data from Genetic Analysis Workshop 16. BMC Proceedings 2009 3:7 *3*, 1–5. https://doi.org/10.1186/1753-6561-3-S7-S96.
22. Serdar, C.C., Cihan, M., Yücel, D., and Serdar, M.A. (2021). Sample size, power and effect size revisited: simplified and practical approaches in pre-clinical, clinical and laboratory studies. Biochem Med (Zagreb) *31*, 1–27. https://doi.org/10.11613/BM.2021.010502.
23. Subramanian, S. (2016). The effects of sample size on population genomic analyses - implications for the tests of neutrality. BMC Genomics *17*, 1–13. https://doi.org/10.1186/S12864-016-2441-8/FIGURES/7.
24. Purcell, S., Neale, B., Todd-Brown, K., Thomas, L., Ferreira, M.A.R., Bender, D., Maller, J., Sklar, P., De Bakker, P.I.W., Daly, M.J., et al. (2007). PLINK: A tool set for whole-genome association and population-based linkage analyses. Am J Hum Genet. https://doi.org/10.1086/519795.
25. Hou, X., Zhang, X., Li, X., Huang, T., Li, W., Zhang, H., Huang, H., and Wen, Y. (2022). Genomic insights into the genetic structure and population history of Mongolians in Liaoning Province. Front Genet *13*, 947758. https://doi.org/10.3389/FGENE.2022.947758/BIBTEX.
26. Patin, E., Lopez, M., Grollemund, R., Verdu, P., Harmant, C., Quach, H., Laval, G., Perry, G.H., Barreiro, L.B., Froment, A., et al. (2017). Dispersals and genetic adaptation of Bantu-speaking populations in Africa and North America. Science *356*, 543–546. https://doi.org/10.1126/SCIENCE.AAL1988.
27. Bai, H., Guo, X., Zhang, D., Narisu, N., Bu, J., Jirimutu, J., Liang, F., Zhao, X., Xing, Y., Wang, D., et al. (2014). The genome of a Mongolian individual reveals the genetic

imprints of Mongolians on modern human populations. Genome Biol Evol *6*, 3122–3136. https://doi.org/10.1093/GBE/EVU242.
28. Racimo, F., Marnetto, D., and Huerta-Sánchez, E. (2017). Signatures of Archaic Adaptive Introgression in Present-Day Human Populations. Mol Biol Evol *34*, 296–317. https://doi.org/10.1093/MOLBEV/MSW216.
29. Skov, L., Coll Macià, M., Sveinbjörnsson, G., Mafessoni, F., Lucotte, E.A., Einarsdóttir, M.S., Jonsson, H., Halldorsson, B., Gudbjartsson, D.F., Helgason, A., et al. (2020). The nature of Neanderthal introgression revealed by 27,566 Icelandic genomes. Nature 2020 582:7810 *582*, 78–83. https://doi.org/10.1038/s41586-020-2225-9.
30. Roca-Umbert, A., Garcia-Calleja, J., Vogel-González, M., Fierro-Villegas, A., Ill-Raga, G., Herrera-Fernández, V., Bosnjak, A., Muntané, G., Gutiérrez, E., Campelo, F., et al. (2023). Human genetic adaptation related to cellular zinc homeostasis. PLoS Genet *19*, e1010950. https://doi.org/10.1371/JOURNAL.PGEN.1010950.
31. Vaughn, A.H., and Nielsen, R. (2024). Fast and Accurate Estimation of Selection Coefficients and Allele Histories from Ancient and Modern DNA. Mol Biol Evol *41*. https://doi.org/10.1093/MOLBEV/MSAE156.
32. Yates, B., Braschi, B., Gray, K.A., Seal, R.L., Tweedie, S., and Bruford, E.A. (2017). Genenames.org: The HGNC and VGNC resources in 2017. Nucleic Acids Res. https://doi.org/10.1093/nar/gkw1033.
33. White, L., Romagné, F., Müller, E., Erlebach, E., Weihmann, A., Parra, G., Andrés, A.M., and Castellano, S. (2015). Genetic adaptation to levels of dietary selenium in recent human history. Mol Biol Evol *32*, 1507–1518. https://doi.org/10.1093/molbev/msv043.
34. Engelken, J., Espadas, G., Mancuso, F.M., Bonet, N., Scherr, A.L., Jímenez-Álvarez, V., Codina-Solà, M., Medina-Stacey, D., Spataro, N., Stoneking, M., et al. (2016). Signatures of evolutionary adaptation in quantitative trait loci influencing trace element homeostasis in liver. Mol Biol Evol *33*, 738–754. https://doi.org/10.1093/molbev/msv267.
35. Wishart, D.S., Tzur, D., Knox, C., Eisner, R., Guo, A.C., Young, N., Cheng, D., Jewell, K., Arndt, D., Sawhney, S., et al. (2007). HMDB: The human metabolome database. Nucleic Acids Res. https://doi.org/10.1093/nar/gkl923.
36. Dib, M.J., Elliott, R., and Ahmadi, K.R. (2019). A critical evaluation of results from genome-wide association studies of micronutrient status and their utility in the practice of precision nutrition. British Journal of Nutrition. https://doi.org/10.1017/S0007114519001119.
37. Kovacs, G., Montalbetti, N., Franz, M.C., Graeter, S., Simonin, A., and Hediger, M.A. (2013). Human TRPV5 and TRPV6: Key players in cadmium and zinc toxicity. Cell Calcium *54*, 276–286. https://doi.org/10.1016/j.ceca.2013.07.003.
38. López Herráez, D., Bauchet, M., Tang, K., Theunert, C., Pugach, I., Li, J., Nandineni, M.R., Gross, A., Scholz, M., and Stoneking, M. (2009). Genetic Variation and Recent Positive Selection in Worldwide Human Populations: Evidence from Nearly 1 Million SNPs. PLoS One *4*, e7888. https://doi.org/10.1371/journal.pone.0007888.

39. Hughes, D.A., Tang, K., Strotmann, R., Schöneberg, T., Prenen, J., Nilius, B., and Stoneking, M. (2008). Parallel selection on TRPV6 in human populations. PLoS One *3*. https://doi.org/10.1371/journal.pone.0001686.
40. Muckenthaler, M.U., Galy, B., and Hentze, M.W. (2008). Systemic iron homeostasis and the iron-responsive element/iron-regulatory protein (IRE/IRP) regulatory network. Annu Rev Nutr *28*, 197–213. https://doi.org/10.1146/ANNUREV.NUTR.28.061807.155521.
41. Khanal, R.C., and Nemere, I. (2008). Regulation of intestinal calcium transport. Annu Rev Nutr *28*, 179–196. https://doi.org/10.1146/ANNUREV.NUTR.010308.161202.
42. Stauber, T., and Jentsch, T.J. (2013). Chloride in vesicular trafficking and function. Annu Rev Physiol *75*, 453–477. https://doi.org/10.1146/ANNUREV-PHYSIOL-030212-183702.
43. Chang, A.R., and Anderson, C. (2017). Dietary Phosphorus Intake and the Kidney. Annu Rev Nutr *37*, 321–346. https://doi.org/10.1146/ANNUREV-NUTR-071816-064607.
44. Jain, G., Ong, S., and Warnock, D.G. (2013). Genetic Disorders of Potassium Homeostasis. Semin Nephrol *33*, 300–309. https://doi.org/10.1016/J.SEMNEPHROL.2013.04.010.
45. Reiss, J., and Hahnewald, R. (2011). Molybdenum cofactor deficiency: Mutations in GPHN, MOCS1, and MOCS2. Hum Mutat *32*, 10–18. https://doi.org/10.1002/HUMU.21390.
46. Horning, K.J., Caito, S.W., Tipps, K.G., Bowman, A.B., and Aschner, M. (2015). Manganese Is Essential for Neuronal Health. Annu Rev Nutr *35*, 71. https://doi.org/10.1146/ANNUREV-NUTR-071714-034419.
47. Bateman, A., Martin, M.J., Orchard, S., Magrane, M., Ahmad, S., Alpi, E., Bowler-Barnett, E.H., Britto, R., Bye-A-Jee, H., Cukura, A., et al. (2023). UniProt: the Universal Protein Knowledgebase in 2023. Nucleic Acids Res *51*, D523–D531. https://doi.org/10.1093/NAR/GKAC1052.
48. Freitas, S.R.S. (2018). Molecular Genetics of Salt-Sensitivity and Hypertension: Role of Renal Epithelial Sodium Channel Genes. Am J Hypertens *31*, 172–174. https://doi.org/10.1093/AJH/HPX184.
49. Rossier, B.C., Pradervand, S., Schild, L., and Hummler, E. (2002). Epithelial sodium channel and the control of sodium balance: Interaction between genetic and environmental factors. Annu Rev Physiol *64*, 877–897. https://doi.org/10.1146/ANNUREV.PHYSIOL.64.082101.143243/CITE/REFWORKS.
50. Houillier, P. (2014). Mechanisms and regulation of renal magnesium transport. Annu Rev Physiol *76*, 411–430. https://doi.org/10.1146/ANNUREV-PHYSIOL-021113-170336/CITE/REFWORKS.
51. Fishilevich, S., Nudel, R., Rappaport, N., Hadar, R., Plaschkes, I., Stein, T.I., Rosen, N., Kohn, A., Twik, M., Safran, M., et al. (2017). GeneHancer: genome-wide integration of enhancers and target genes in GeneCards. Database *2017*, 1–17. https://doi.org/10.1093/DATABASE/BAX028.

52. Phan, L., Zhang, H., Wang, Q., Villamarin, R., Hefferon, T., Ramanathan, A., and Kattman, B. (2025). The evolution of dbSNP: 25 years of impact in genomic research. Nucleic Acids Res *53*, D925–D931. https://doi.org/10.1093/NAR/GKAE977.
53. Rentzsch, P., Witten, D., Cooper, G.M., Shendure, J., and Kircher, M. (2019). CADD: predicting the deleteriousness of variants throughout the human genome. Nucleic Acids Res *47*, D886–D894. https://doi.org/10.1093/NAR/GKY1016.
